# Uneven distribution of enamel, dentine and cementum in cheek teeth of domestic horses (*Equus caballus*): A micro computed tomography study

**DOI:** 10.1371/journal.pone.0183220

**Published:** 2017-08-16

**Authors:** Lauritz Martin Englisch, Kathrin Kostrzewa, Susan Kopke, Klaus Failing, Carsten Staszyk

**Affiliations:** 1 Institute of Veterinary-Anatomy, -Histology and -Embryology, Faculty of Veterinary Medicine, Justus Liebig University Giessen, Giessen, Germany; 2 Clinical Skills Lab, University of Veterinary Medicine Hannover Foundation, Hannover, Germany; 3 Unit for Biomathematics and Data Processing, Justus Liebig University Giessen, Giessen, Germany; Ecole Normale Supérieure de Lyon, FRANCE

## Abstract

**Background:**

Hypsodont equine cheek teeth possess large dental crowns, resting partly in the bony alveolus. Over a horse’s life cheek teeth erupt continuously to compensate for occlusal wear of 3–4 mm per year. Parts of the crown initially resting in the bony alveolus become progressively exposed at the occlusal surface with time. Hitherto, it is unclear whether the typical structure of the equine occlusal surface, composed of a complex arrangement of enamel, dentin and cementum, remains constant or undergoes structural changes with age. Therefore, we tested the hypothesis that the occlusal surface composition does not remain constant by a quantitative analysis of the dental substances at multiple levels along the dental crown of equine cheek teeth.

**Methods:**

Micro-computed tomography scans of 20 upper cheek teeth and 16 lower cheek teeth from 19 domestic horses were morphologically analysed using imaging and measurement software. Area for individual dental substances was measured at different levels from the apex to the occlusal surface. The data was statistically analysed to detect changes in the area of individual substance along the dental crown. The area of peripheral cementum was measured separately for levels inside and outside the bony alveolus.

**Results:**

In both, upper and lower cheek teeth, enamel area decreased in an apical direction, while dentine area increased. Peripheral Cementum increased dramatically in the occlusal/coronal extra-alveolar position.

**Conclusion:**

With increasing age the occlusal surface content of dentine increases while the content of enamel decreases. These changes are considered relevant for the detailed explanation of forage disruption in horses as well as for the recommendation of concepts in equine dentistry.

## Introduction

Over the millennia, the horse adapted to changes in feeds available from a foraging animal to the modern grazing creature [1]. As a result of this evolutionary progress the teeth of the today’s horse changed from a short crowned, brachydont form to the long crowned, hypsodont form to withstand their highly abrasive feed. Hypsodonty is defined as a large tooth crown height compared to tooth length [2,3].The crown of equine cheek teeth (CT) can be divided in a clinical crown (visible in the oral cavity) and a reserve crown resting within the bony alveolus. The tooth becomes completed by the development of a dental root which is composed of dentin and cementum but lacks enamel. To compensate for occlusal wear (3–4 mm / year) [4] caused by attrition (tooth to tooth contact) and abrasion (tooth to food contact) [5], CT erupt continuously during a horse’s life [6]. Consequently parts of the tooth crown initially resting in the bony alveolus become revealed at the occlusal surface with time.

The dental substances (enamel, dentine and cementum) are forming characteristic patterns on the occlusal surface for upper cheek teeth (UCT) and lower cheek teeth (LCT). Dentine and cementum wear out faster than enamel which results in the formation of dentine cups and enamel ridges. This arrangement is lost, when the tooth crown is worn down and parts of the dental root—without a complete layer of enamel—are exposed at the occlusal surface. From an embryological conception cementum is not part of the tooth unlike dentine and enamel, rather it belongs to the periodontium [7,8]. Therefore we refer in this study to the tooth substances as dentine and enamel without peripheral cementum. It remains unclear if the distribution of enamel and dentine changes along the anatomical crown and therefore the ratio of dentine, enamel and cementum changes at the occlusal surface with age. Findings in another equidae species (Plains Zebra, *Equus quagga*) documented an uneven distribution of relative enamel content along the dental crown with higher enamel contents near the apex. This result implies that the content of enamel at the occlusal surface increases with time [2].

Hence we propose a difference in the amount and ratio of the substances enamel, dentine and cementum along the dental crown in domestic horses (*Equus caballus)*. To test this hypothesis, we examined the amount of these substances at defined levels along the tooth axis using micro-computed tomography (microCT) scans and picture analysis software to detect differences in their distribution.

## Material and methods

### Material

A total of 36 CT (20 UCT and 16 LCT, Triadan positions 7–10) were included in this study (Table 1). CT from Triadan position 6 and 11 were excluded due to their very particular shape. Furthermore only CT with a minumum crown height of 20 mm were taken into account. The CT were dissected along with their bony alveoli from the heads of 19 domestic horses of different age, sex and breed *(Equus caballus)*. Horses were euthanized by licensed veterinarians on humane grounds for non-dental reasons. The cadaveric heads were provided either by the Institute for Pathology of the University of Veterinary Medicine Hannover or directly from the Clinic for Horses of the University of Veterinary Medicine Hannover.

**Table 1:** Upper and lower cheek teeth used in this study.

group	teeth [n]	age range [years]	reserve crown height [mm]
UCT	20	1.5–20	21.91–62.49
LCT	16	1.5–16	21.57–75.77

UCT, Upper cheek teeth; LCT, Lower cheek teeth

The dental ages were determined as published by Dixon and Kopke[9,10]. CT were prepared for microCT scans using a saber bone saw (Schmid & Wezel GmbH & Co KG, Maulbronn, Germany) steel band saw (Kolbe GmbH, Elchingen, Germany) and diamond coated band saw (Proxxon GmbH, Föhren, Germany). Each CT resting within its bony alveolus was scanned using a microCT (Scanco Medical AG, Brüttisellen, Switzerland), resulting in 1000–2000 two dimensional DICOM (Digital Imaging and Communications in Medicine) per tooth with a minimum resolution 0.082 mm. For further details please see Kopke et al. [10]

### Methods

#### Defining the horizontal slice position along the dental crown

**Fig 1:**
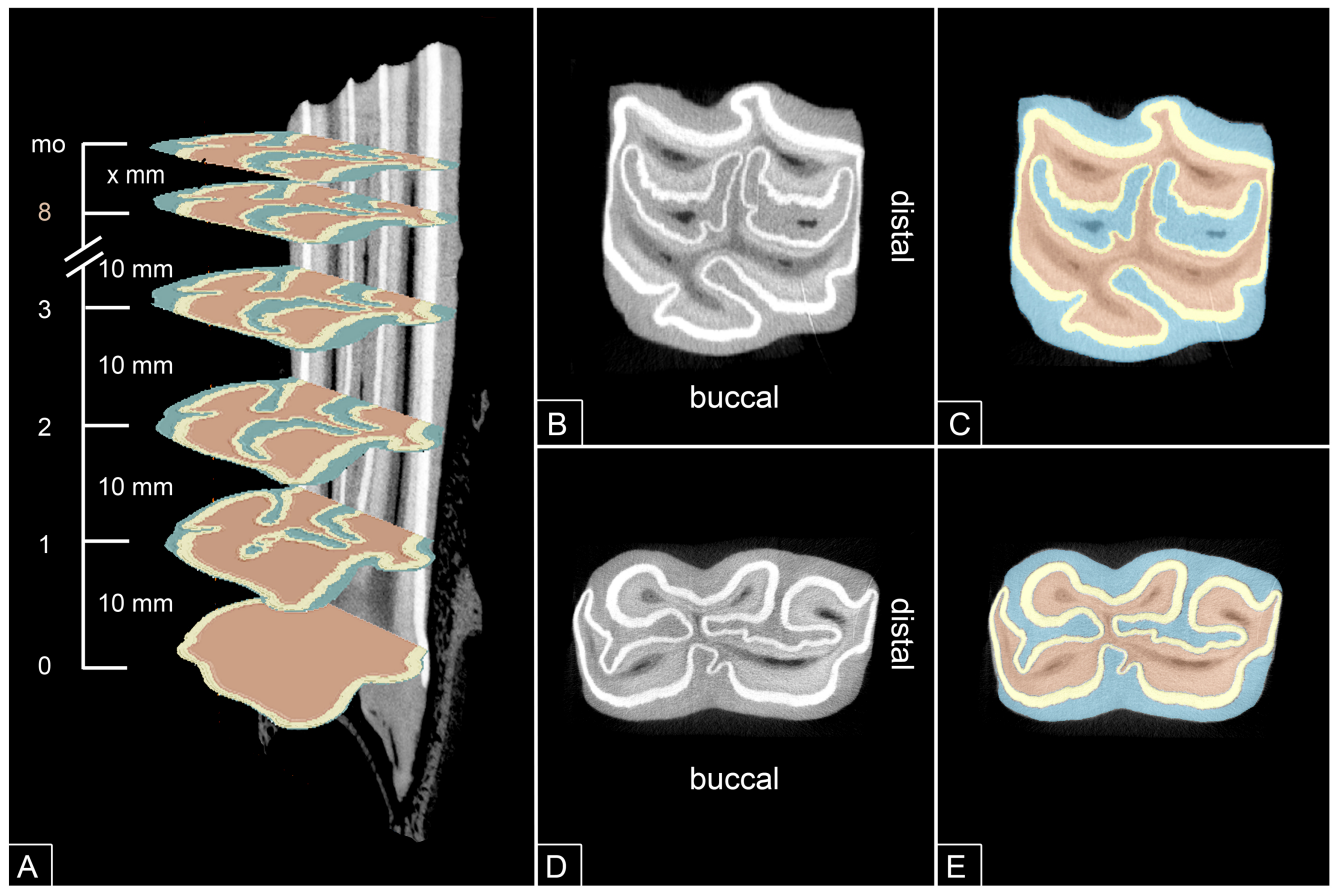
MicroCT images of a 109. (A)MicroCT image of a 109, longitudinal section. Depending on the tooth height up to nine equal spaced (10 mm) horizontal sections presenting a complete layer of peripheral enamel were selected for measurements. Additionally the most occlusal section (mo) of the crown showing a complete layer of peripheral enamel was selected. (B,D) MicroCT cross section showing the typical arrangement of the dental substances of an upper (B) and lower (D) cheek tooth. (C,E) MicroCt cross section as in B,D with selected enamel (yellow), dentine (light red) and cementum (blue) to define areas for measurement.

The DICOM images of each tooth were transferred to the software Osirix (Pixmeo, Geneva, Switzerland), to determine the absolute position of the individual slides. Each tooth was aligned to its longitudinal axis and 2D microCT images were visualized. The most apical image displaying a complete layer of peripheral enamel was assigned as level 0 being the most apical level of the dental crown. Further microCT images were selected every 10 mm along the longitudinal axis of the tooth (Fig 1). The most occlusal image showing a complete layer of peripheral enamel was selected last to be analysed (closest to occlusal surface). By calculating the distance between the most apical and most occlusal image, the individual dental crown height was determined.

#### Amount and ratio of the dental substances

Dicom images were transferred to the software Amira (Visualization Sciences Group, Merignac Cedesx, France) for further processing. The substances enamel, dentine and cementum were identified on 2D microCT images (Fig 1) and were selected using automatic segmentation tools (Fig 1). Each segmentation was reviewed visually and if necessary corrected by manual segmentation. For UCT, the area of peripheral and infundibular cementum as well as of peripheral and infundibular enamel were recorded separately. The area per slice in mm² of each selected substance was calculated by the software. When a selected slice still showed pulp cavities, these areas were added to the dentine. Relative area for the measured substances was calculated by dividing each substance by the absolute area of all substances excluding peripheral cementum in a single slide. Furthermore the area of peripheral cementum was calculated separately for slides associated with an intra-alveolar position and for slides associated with an extra-alveolar position for comparison of mean area of peripheral cementum inside and outside of the bony alveolus.

### Statistical analysis

Statistical analyses were performed with the BMDP program package (Dixon, BMDP Statistical Software Manual, University of California Press, Berkeley, Los Angeles). UCT and LCT were analyzed separately due to the morphological difference of the teeth.

For the majority of the variables, data descriptions represent the arithmetic mean ± standard deviation (SD). To detect a change in the absolute and relative area of the tooth substances along the crown, the regression coefficient (slope) of each measured variable dependent from the position of selected 2D microCT images in mm within each tooth was calculated by available data transformation in form of the linear regression coefficient. Only teeth with at least two measuring points were taken in account. Subsequently, by one-sample t-test the mean slope value was tested for deviation from the hypothetical value zero to test for a statistical significant trend.

According to the design of the study the comparison of the area of peripheral cementum intra- versus extra-alveolar the matched-pairs-t-test was applied.

For each variable, the level of statistical significance was set at *p* ≤ 0.05.

Furthermore, for each measured variable the intra-class correlation coefficent (ICC) was calculated being based on the one-way analysis of variance with the horse as the class defining variable. Hereby the total variance of the data is decomposed in the part explained by horses and the part between teeth within the horses (intra-class variance) in order to quantify the amount of correlation within an individual horse with multiple teeth for the variable enamel, infundibula and dentine respectively distinguishing between intra and extra alveolar positions for peripheral cementum.

## Results

### Upper cheek teeth

**Fig 2:**
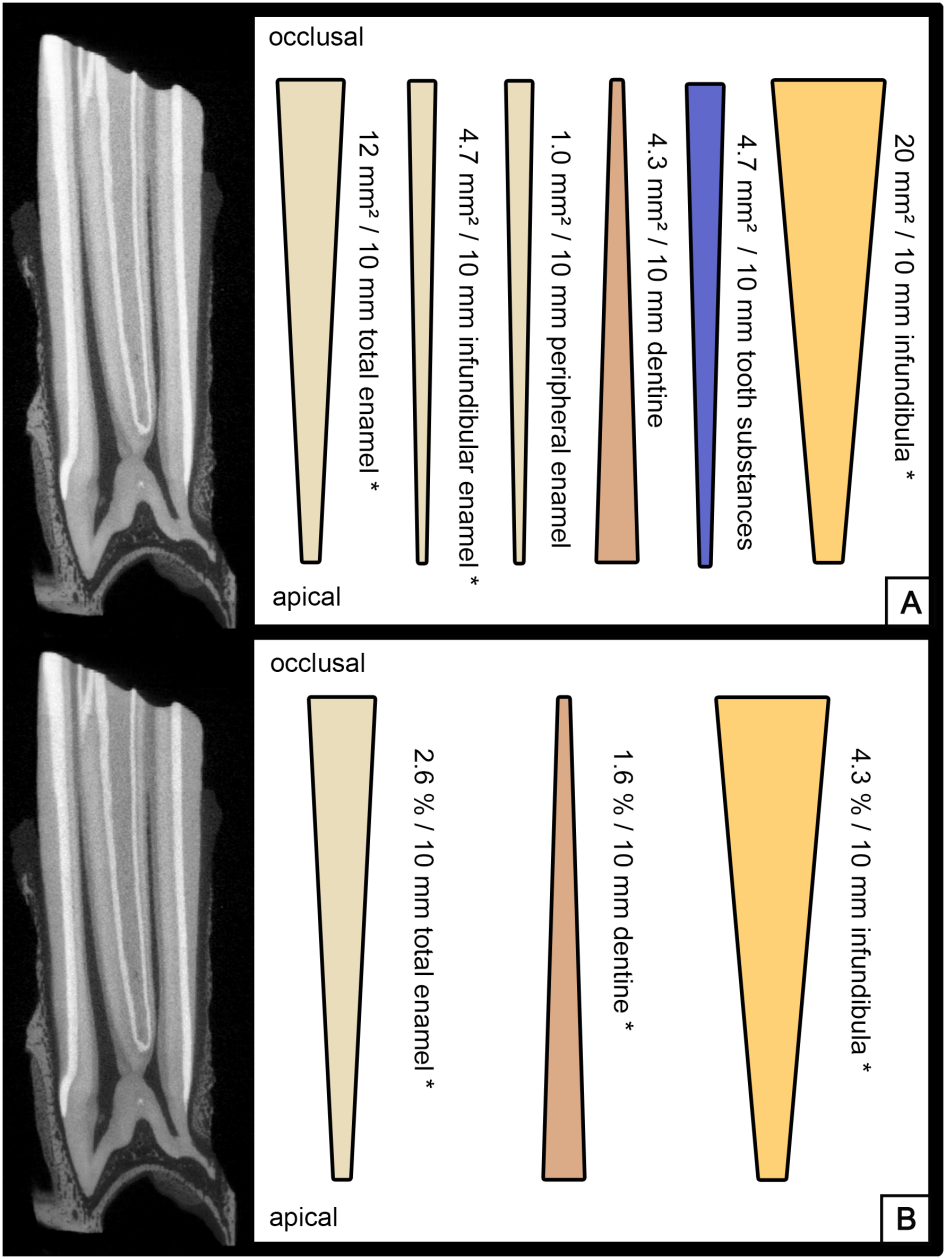
MicroCT of an UCT, longitudinal section. Bars indicating decrease / increase of dental substances along the dental crown. P values < 0.05 are marked with *. (A) Calculated mean absolute changes per 10 mm crown height. (B) Calculated mean relative changes per 10 mm crown height.

From occlusal to apical the absolute area of enamel (peripheral enamel and infundibular enamel), the absolute area of infundibular enamel as well as the complete area of the infundibula (infundibular enamel and infundibular cementum) decreased (all p < 0.01). The absolute area of peripheral enamel as well as the absolute area of the tooth (peripheral enamel + complete infundibula + dentine) decreased, however these changes were not statistically significant (p = 0.31 and p = 0.33). The absolute area of dentine increased, however this increase was not statistically significant (p = 0.09) (Fig 2).

**Fig 3:**
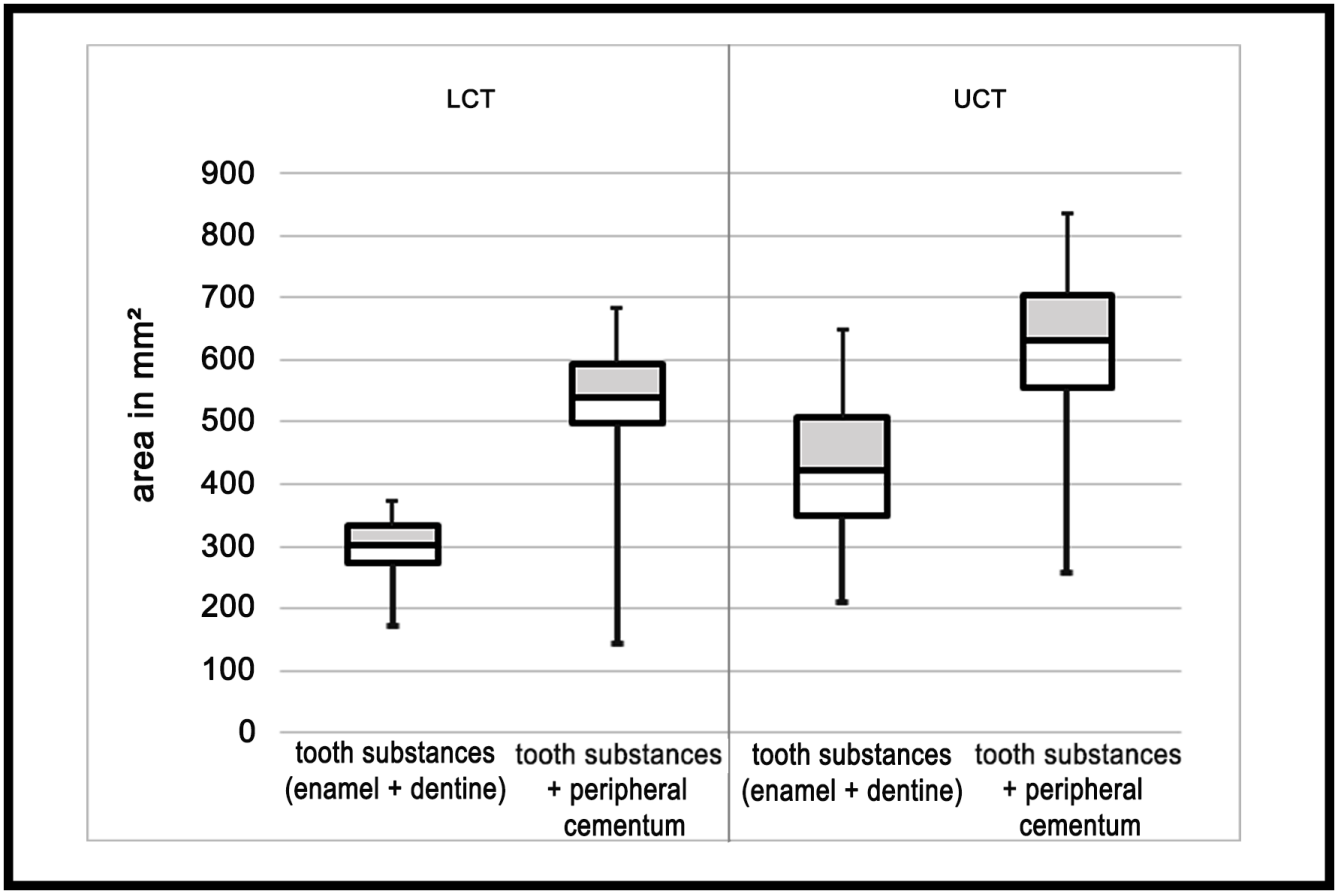
Boxplots representing the absolute area of tooth substances (dentine + enamel) and the absolute area of tooth substances plus peripheral cementum for UCT and LCT. The boxes represent the interquartile range (25%, 75%), the horizontal line the median and the whiskers the range.

The relative area of the complete enamel (peripheral and infundibular enamel) in relation to the complete tooth area (excluding peripheral cementum) decreased (p < 0.001), while the relative area of dentine increased from occlusal to apical (p < 0.01). The relative area of the infundibula (infundibula enamel and infundibular cementum) decreased (p < 0.001) (Fig 2).The absolute area of tooth substances (enamel and dentine) including peripheral cementum ranged from 256.1 to 837.1 mm² (mean 621.9 mm² ± 115.5 mm²). The absolute area of tooth substances (dentine and enamel) excluding peripheral cementum ranged from 144.7 to 682.4 mm² (mean 529.7 mm² ± 95.1 mm²) (Fig 3).

**Fig 4:**
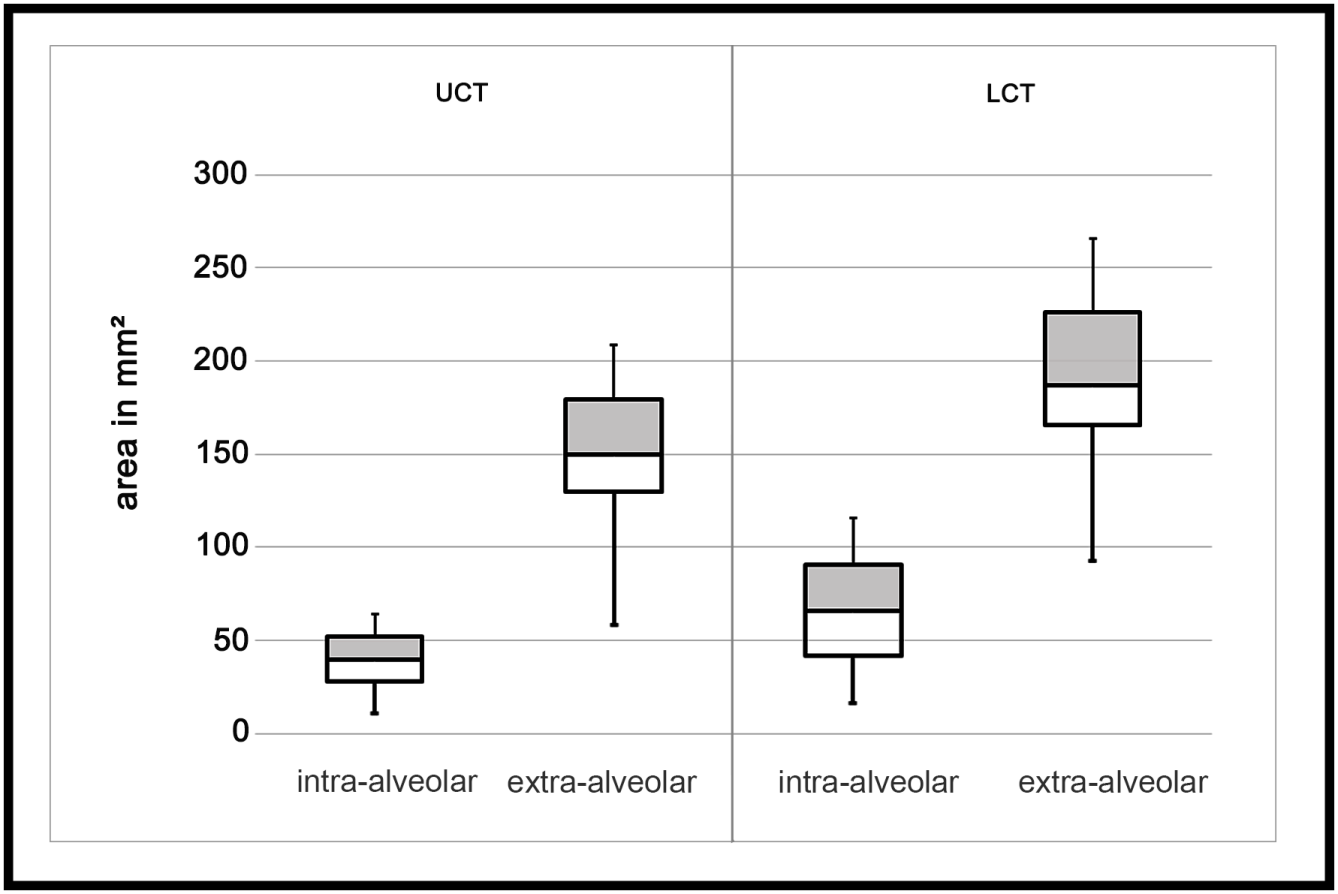
Boxplots representing the area of peripheral cementum in intra-alveolar and extra-alveolar position for LCT and UCT. The boxes represent the interquartile range (25%, 75%), the horizontal line the median and the whiskers the range.

The area of peripheral cementum was approximately 3.4 times higher in extra-alveolar positions (mean 148.8 mm² ± 32.5 mm²) than in intra-alveolar positions (mean 43.8 mm² ± 11.8 mm²) (p < 0.01) (Fig 4). Results of the intra-class correlation analysis can be found in Table 2.

**Table 2:** Results of the intra-class correlation analysis for the slopes concerning UCT.

	tooth substance	variance component between		population ICC	p-value
		horses	teeth within individual horse		
**absolute data**	total enamel	0.385	0.744	0.341	0.725
	infundibular enamel	0.002	0.001	0.711	0.170
	peripheral enamel	0.0451	0.300	0.129	0.958
	dentine	0.456	1.004	0.313	0.766
	tooth substances	2.152	7.877	0.215	0.887
	infundibula	5.380	1.965	0.733	0.105
	peripheral cementum	1465.533	377.828	0.795	0.096
**relative data**	total enamel	0.141	0.144	0.494	0.471
	infundibular enamel	0.272	0.123	0.677	0.212
	peripheral enamel	0.024	0.054	0.308	0.773
	dentine	0.018	0.029	0.378	0.671
	infundibula	0.413	0.279	0.596	0.295
	peripheral cementum	37.046	4.929	0.882	0.031

ICC, intra-class correlation

### Lower cheek teeth

**Fig 5:**
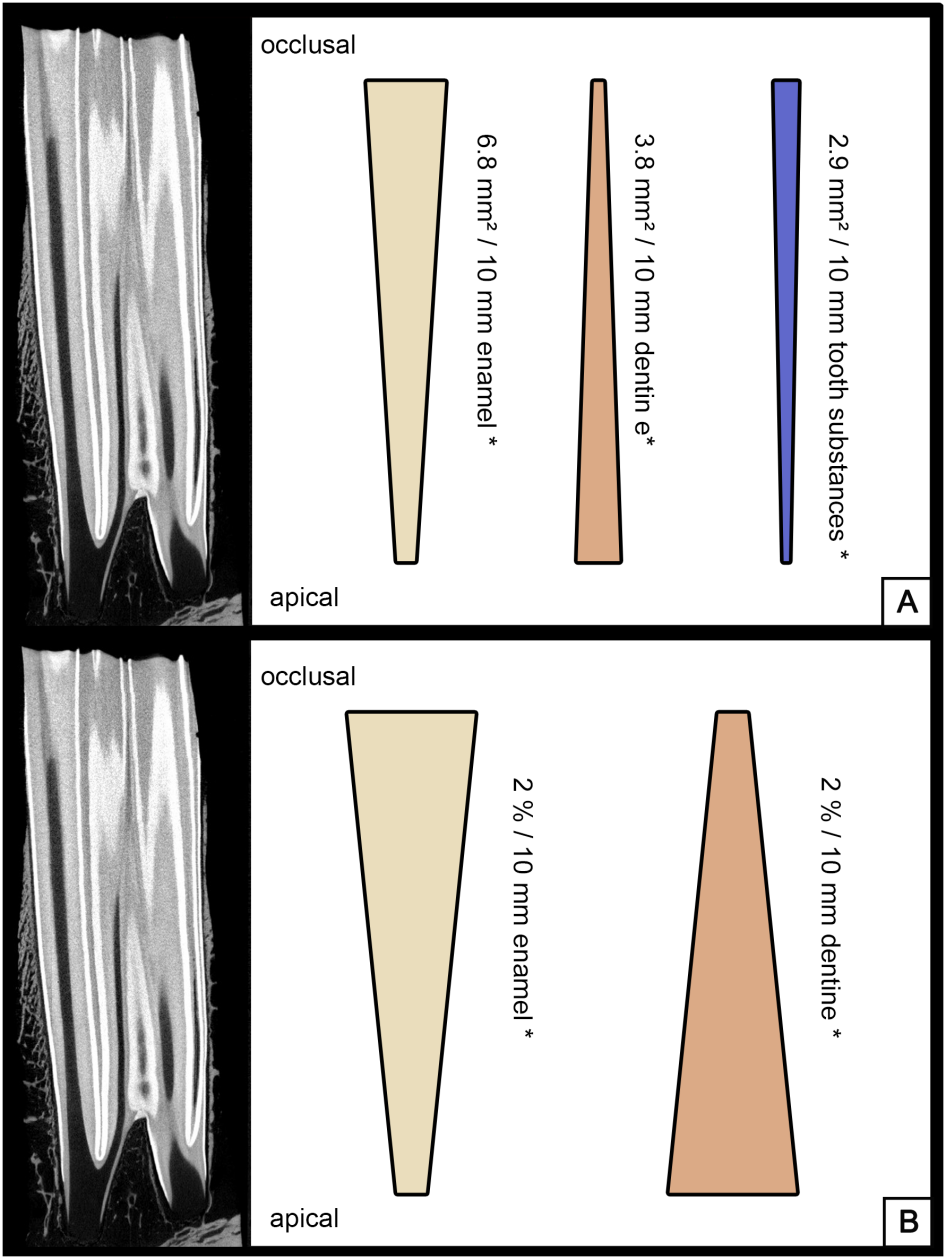
MicroCT of an LCT, longitudinal section. Bars indicating decrease / increase of dental substances along the dental crown. P values < 0.05 are marked with *. (A) Calculated mean absolute changes per 10 mm crown height. (B) Calculated mean relative changes per 10 mm crown height.

From occlusal to apical the absolute area of enamel and the complete tooth area (enamel and dentine, excluding peripheral cementum) decreased (p < 0.001 and p = 0.03), while the absolute area of dentine increased (p = 0.02) (Fig 5).

The relative area of enamel in relation to the complete tooth area excluding peripheral cementum decreased (p < 0.001), while the relative dentine area increased (p < 0.001) (Fig 5).The absolute area of tooth substances (enamel and dentine) including peripheral cementum ranged from 209.5 to 650.1 mm² (mean 422.3 mm² ± 97.8 mm²). The absolute area of tooth substances (dentine and enamel) excluding peripheral cementum ranged from 170.9 to 370.5 mm² (mean 293.8 mm² ± 45.1 mm²) (Fig 3).

The area of peripheral cementum was approximately 2.7 times higher in extra-alveolar positions (mean 192.9 mm² ± 38.7 mm²) than in intra-alveolar positions (mean 71.7 mm² ± 23.8 mm ²) (p < 0.01) (Fig 4). Results of the intra-class correlation analysis can be found in Table 3.

**Table 3:** Results of the intra-class correlation analysis for the slopes concerning LCT.

	tooth substance	variance component between		population ICC	p-value
		horses	teeth within individual horse		
**absolute data**	enamel	0.546	0.126	0.812	0.051
	dentine	0.088	0.116	0.439	0.540
	tooth substances	0.436	0.185	0.708	0.157
	peripheral cementum	3078.914	516.706	0.856	0.024
**relative data**	enamel	0.029	0.019	0.7133	0.144
	dentine	0.029	0.019	0.7133	0.145
	peripheral cementum	21.515	25.905	0.458	0.518

ICC, intra-class correlation

## Discussion

Our results show an uneven distribution of enamel and dentine along the dental crown in both UCT and LCT. Therefore the ratio of enamel and dentine changes at the occlusal surface with age. We documented a decrease of enamel in an apical direction. These findings are different from those of Winkler et al. [2], who reported highest relative enamel content in the most apical quarter of the dental crown in UCT of a Plains Zebra *(Equus quagga)* in relation to the tooth substances (enamel, dentine and the infundibular area). Furthermore, Winkler et al. reported lowest relative enamel content in the most upper (most occlusal) quarter in UCT. However, the calculations of enamel content we performed were different from those performed by Winkler et al. [2]. We measured a change in enamel content along the tooth crown by selecting a certain number of slides rather than cutting each individual tooth in 4 sections and measuring enamel content in relation to the volume of each individual sections. The zebra specimen examined by Winkler et al. featured newly erupted CT that were hardly worn down by attrition. Equine CT in such a young state feature very distinct structural differences compared to fully erupted and functional CT. The clinical crowns of newly erupted CT possess enamel cusps covered by cementum rather than featuring a typical occlusal surface composed of enamel ridges and dentin cups. At the apical end of newly erupted CT a vital enamel organ is still present and the development of enamel and dentin is still in progress. These distinct structural features might have had influenced of the results of Winkler et al. For our examinations measurements were performed exclusively in sections with already completed enamel formation. This was confirmed by the presence of peripheral cementum, which can only form after degeneration of the enamel organ. Once the enamel organ is degenerated, the formation of new enamel layers is impossible.

For all teeth included in our study we chose an identical starting point for measurements in the apical region (level 0), that represented the most apical level of the dental crown. We then defined levels up to the occlusal surface for measuring a change of the tooth substances in relation to the level 0. This approach guaranteed to collect morphometric data which were not influenced by the dental age and therefore by occlusal wear. Because changes of area occupied by dental substances were calculated in relation to the most apical section of the dental crown, we could compare a large group of CT and detect general trends in the distribution, even if the CT were from different dental ages and therefore from different crown heights. Enamel in CT features a characteristic arrangement with many invaginations into the adherent dentine. In UCT the infundibula further increase the enamel area of the occlusal surface with multiple invaginations. In LCT deep invaginations of the peripheral enamel appear to compensate for the absence of the two infundibula [11].

These enamel invaginations were also described by Suske et al. and Kilic et al. [11,12] and become less deep and complex in apical direction. This morphological observation is consistent with our morphometric findings of a decreasing amount of enamel from occlusal to apical. The decrease of enamel folds in an apical direction in CT was also described by du Toit et al. [7,13]. With enamel being the hardest substance in the body [14–16] one can expect that it plays a major role in the wear resistance against occlusal wear by attrition and abrasion. In our study absolute and relative enamel area decreased in both LCT and UCT in apical direction. Thus with increasing age, as deeper parts of the reserve crown become revealed at the occlusal surface, the amount of enamel decreases at the occlusal surface. A decrease of enamel should lead to a decrease in wear resistance with age, because less enamel should logically lead to less wear resistance. Furthermore we observed a shift from lower relative enamel area towards higher relative dentine area in an apical direction in both UCT and LCT. Consequently, overall dentine area increases with age at the occlusal surface. These changes in the quantitative composition of the occlusal surface might lead to an overall decrease of wear resistance with age in domestic horses. However, according to clinical observations [17,18], teeth of young horses appear to be softer and less resistant to floating (i.e. corrective dental procedure performed with manual rasps or motorized floats) than CT of older horses. This suggests an increase of wear resistance with age. Though this increase is contradictory to our morphological findings when referring to the mere amounts of enamel and dentine. Thus, we assume that the decreasing enamel content at the occlusal surface is compensated by distinct processes which ensure sufficient resistance and therefore sufficient function of the tooth during mastication. Studies of equine incisor teeth documented that secondary dentine was harder on mid tooth levels than on a level near the occlusal surface [13,19]. Thus, dentine hardness at the occlusal surface increases with age. These findings are explained by a reported lifelong addition of highly mineralized peritubular dentine within the dentinal tubules [8]. Another characteristic of hypsodont CT is the ongoing mineralization of enamel after eruption compared to brachydont teeth [20]. Enamel mineralization takes place in two main phases: first matrix production and second enamel maturation [20,21]. Most of the mineralization takes place in the second phase. After these two phases the growth in thickness of enamel layers has been completed. Derived from this observation enamel layers near the apex in the dental crown have a longer maturation period. The higher mineral content of enamel layers near the apex may compensate for the lower amounts of enamel at the occlusal surface and therefore additionally protect from progressive occlusal wear. However to test the mineralization assumption further studies are required to measure overall mineral content in enamel layers at different levels along the tooth crown in CT. Furthermore the clinical observation of softer teeth in young horses and therefore our proposed increase of wear resistance with age has not yet been confirmed by structured and reliable studies. The thickness and area of enamel in CT cannot change after the enamel organ has been destroyed and cementum has been deposited on its peripheral surface within the alveolus. The same applies for the area of dentine which is completely enclosed by the peripheral enamel. At subocclusal levels pulp cavities are present surrounded by dentine. Prior to exposure at the occlusal surface these pulp cavities become completely filled with secondary dentine [11]. Consequently these pulp areas were added to dentine during measurements. Therefore all measurements of dentine and enamel on horizontal levels, even when taken at levels in the reserve crown, reflect the amount of these substances when the respective levels become exposed at the occlusal surface. This does not apply for the peripheral cementum because peripheral cementum is described as a dynamic tissue and is continuously produced throughout the life of a CT [8]. Measured amounts of peripheral cementum at the reserve crown within the bony alveolus do not represent the amounts of peripheral cementum exposed at the occlusal surface by the time the respective levels erupt. Therefore measuring a change in amounts of peripheral cementum with the same statistical calculations we performed on amounts of dentine and enamel would have led to biased results. So we chose to compare amounts of peripheral cementum with a one group t-test between levels inside and outside the bony alveolus. A thin layer of peripheral cementum within in the bony alveolus provides anchorage for the periodontal ligament [4,22].

This is the typical function of cementum located at the intra-alveolar portion of the tooth. In this regard, equine hypsodont teeth resemble brachyodont teeth [23]. However, in contrast to brachyodont teeth, equine hypsodont teeth feature cementum also in an extra-alveolar position at the occlusal surface and at the clinical crown. In this extra-alveolar position the thickness of peripheral cementum increases dramatically and cementum is then considered as a major structural component of the clinical crown and occlusal surface [22,24]. This increase suggests a pronounced cementogenesis in a periodontal zone just below the alveolar crest. This assumption is supported by results of Warhonowicz et al., that documented highest cementoblast cells density in a subgingival periodontal position directly adjacent to the equine tooth [25]. In such a periodontal zone in hypsodont cheek teeth, cementoblast cells produce several millimetres of cementum per year. This is a remarkable high production rate compared to cementum production rate in brachydont teeth of only a few micrometres [26].

The documented high production rate of cementum in the periodontium of equine cheek teeth further supports studies that suppose very high regenerative capacities of the equine periodontal tissue [25,27,28]. In our study we examined a group of individual teeth from horses of different breeds. Teeth from different breeds might differ in size, i.e. pony vs draft horse. Accordingly we analysed absolute and relative data. Relative data are independent of size and confirmed our findings using absolute data. Both data sets showed a clear shift towards higher dentine content and lower enamel content in an apical direction. However, our data were not suitable to analyse the influence of breed and sex on the composition of dental hard substances, because the specimen number per breed was to low. Calculated absolute and relative values for an increase or decrease of each tooth substance along the tooth axis might be influenced by individual particularities of single horses or teeth. However, the results of the ICC analysis showed, that the influence of the single individuals are not significant regarding almost all tooth substances. Therefore overall results have not been strongly influenced by a single specimen. Further studies with a higher number of CT should be conducted to analyse breed influence or tooth position. Another limitation of our study was, that due to technical limitations, only selected CT of one arcade were examined using microCT and picture analysis software. Recent morphological findings in mammalian herbivores documented that enamel content varies between tooth positions. The third molar showed highest enamel content independent of phylogeny [14]. Further studies with a higher numbers of CT from one individual horse are required to elucidate whether those intraindividual differences apply for domestic horses as well.

## Conclusion

Dentine and enamel are not evenly distributed along the crown in equine CT. Consequently, the complex composition of the occlusal surface changes with time, i.e. the content of dentine increases while the content of enamel decreases with age. Further studies need to evaluate the biomechanical implications (resistance against of occlusal wear) and clinical consequences (corrective dental procedures) of these findings.

## Supporting information

S1 Table Complete list of upper cheek teeth used for this study. (DOCX)

S2 Table Complete list of lower cheek teeth used for this study. (DOCX)

S1 File Excel data sheet. Measured area in mm² of the different substances for UCT and LCT used in this study. Each variable was taken at a specific level along the tooth axis. Furthermore for each level peripheral cementum was assigned to an extra- or intra-alveolar position.
(XLSX)

S2 File Results of the t-test. All measured dental substance (area in mm²) were statistical analysed. The regression coefficient (slope) of each measured variable dependent from the position of selected 2D microCT images in mm within each tooth was calculated by available data transformation in form of the linear regression coefficient.
(DOCX)

S3 File Results of the matched pair t test for peripheral cementum. Mean value of peripheral cementum of intra-alveolar positioned selected slides = 1 compared to mean value of peripheral cementum of extra-alveolar positioned selected slide = 3.
(DOCX)

S4 File Results of the intra-class correlation coefficient test. For each measured variable the intra-class correlation coefficent (ICC) was calculated being based on the one-way analysis of variance with the horse as the class defining variable.
(DOCX)
