# Supplementary material for: Uneven distribution of enamel, dentine and cementum in cheek teeth of domestic horses (Equus caballus): A micro computed tomography study
Source: PLoS One. 2017 Aug 16;12(8):e0183220. doi: 10.1371/journal.pone.0183220 (PMC5558931; doi:10.1371/journal.pone.0183220)
Supplement: S1 Table — (DOCX) [file pone.0183220.s001.docx]

**Table 2**

| tooth # | triadan position | age of tooth  [years] | breed | reserve crown length  [mm] |
| --- | --- | --- | --- | --- |
| 1 | 107 | 5 | unkw. | 49,44 |
| 2 | 107 | 8 | unkw. | 47,97 |
| 3 | 107 | 13 | unkw. | 24,44 |
| 4 | 107 | 12 | draft horse | 21,91 |
| 5 | 108 | 1 | draft horse | 51,46 |
| 6 | 109 | 20 | throughbr. | 21,98 |
| 7 | 109 | 3 | draft horse | 62,49 |
| 8 | 109 | 15 | unkw. | 47,15 |
| 9 | 109 | 12 | unkw. | 28,12 |
| 10 | 109 | 9 | throughbr. | 41,49 |
| 11 | 110 | 3,5 | warmbl. | 62,4 |
| 12 | 110 | 15 | warmbl. | 29,23 |
| 13 | 207 | 2,5 | warmbl. | 53,57 |
| 14 | 207 | 14 | warmbl. | 25,99 |
| 15 | 208 | 11 | draft horse | 27,56 |
| 16 | 209 | 13 | pony | 29,35 |
| 17 | 209 | 4,5 | warmbl. | 54,36 |
| 18 | 209 | 16 | warmbl. | 27,39 |
| 19 | 210 | 20 | throughbr. | 45,34 |
| 20 | 210 | 13 | draft horse | 27,46 |
