# Supplementary material for: Uneven distribution of enamel, dentine and cementum in cheek teeth of domestic horses (Equus caballus): A micro computed tomography study
Source: PLoS One. 2017 Aug 16;12(8):e0183220. doi: 10.1371/journal.pone.0183220 (PMC5558931; doi:10.1371/journal.pone.0183220)
Supplement: S2 Table — (DOCX) [file pone.0183220.s002.docx]

**Table 3**

| tooth # | triadan position | age of tooth  [years] | breed | reserve crown length  [mm] |
| --- | --- | --- | --- | --- |
| 1 | 307 | 2,5 | warmbl. | 60,84 |
| 2 | 307 | 14 | warmbl. | 22,14 |
| 3 | 308 | 11 | draft horse | 27,06 |
| 4 | 309 | 4,5 | warmbl. | 67,16 |
| 5 | 309 | 16 | warmbl. | 24,85 |
| 6 | 407 | 5 | unkw. | 59,53 |
| 7 | 407 | 10 | unkw. | 24,85 |
| 8 | 407 | 8 | unkw. | 39,94 |
| 9 | 407 | 11 | unkw. | 21,57 |
| 10 | 407 | 14 | unkw. | 31,82 |
| 11 | 407 | 12 | draft horse | 24,68 |
| 12 | 408 | 1,5 | warmbl. | 75,77 |
| 13 | 409 | 15 | unkw. | 45,68 |
| 14 | 409 | 16 | unkw. | 37,88 |
| 15 | 410 | 3,5 | warmbl. | 69,27 |
| 16 | 410 | 15 | warmbl. | 27,63 |
