# Supplementary material for: Uneven distribution of enamel, dentine and cementum in cheek teeth of domestic horses (Equus caballus): A micro computed tomography study
Source: PLoS One. 2017 Aug 16;12(8):e0183220. doi: 10.1371/journal.pone.0183220 (PMC5558931; doi:10.1371/journal.pone.0183220)
Supplement: S2 File — All measured dental substance (area in mm²) were statistical analysed. The regression coefficient (slope) of each measured variable dependent from the position of selected 2D microCT images in mm within each tooth was calculated by available data transformation in form of the linear regression coefficient. (DOCX) [file pone.0183220.s004.docx]

UPPER CHEEK TEETH

BMDP3D - T-TESTS

Copyright 1977, 1979, 1981, 1982, 1983, 1985, 1987, 1988, 1990, 1993

by BMDP Statistical Software, Inc.

Statistical Solutions Ltd. | Statistical Solutions

Unit 1A, South Ring Business Park | Stonehill Corporate Center, Suite 104

Kinsale Road, Cork, Ireland | 999 Broadway, Saugus, MA 01906, USA

Phone: + 353 21 4319629 | Phone: 781.231.7680

Fax: + 353 21 4319630 | Fax: 781.231.7684

e-mail: sales@statsol.ie | e-mail: info@statsolusa.com

Website: http://www.statsol.ie | Website: http://www.statsolusa.com

Release: 8.1 (Windows 9x, 2000, Me, Xp) Date: 04/27/16 at 12:57:39

Manual: BMDP Manual Volumes 1, 2, and 3.

Digest: BMDP User's Digest.

IBM PC: BMDP PC Supplement -- Installation and Special Features.

PROGRAM INSTRUCTIONS

/prob title = 'Herr Lauritz Englisch: A3D2.inp *** Quantitative Studien

zum Schmelzgehalt in Pferdebackenzaehnen.

- 1 Fall = 1 Zahn = 10 Lokalisationen = 10 Zeilen

- Hier: Oberkiefer, Zahn 7 - 10

- Umrechnung der relativen Werte in Prozentwerte

- Berechnung der Flächenanteile ohne aeusseren Zement

- mit Berechnung der Steigung "ST" ueber die Schnittebenen

- Einstichproben-t-Test gegen H0: ST = 0

***'.

/inp var = 276.

file = a.

format = '32f10,9(/50x,27f10), /20x,1f10'. ## Mit Abstandsangabe für die Ebene

/var names = zahnid,zp,qu,znr,za,

for lo = 0 to 9.% ## Lokalisation

for va = lok,statu,ps,pz,isd,izd,ism,izm,idz,id,ges,sges,dz_sb,i_zb,

inf,ps_rel,s_rel,pz_r,isd_r,izd_r,ism_r,izm_r,idz_r,

id_r,sges_r,i_zb_r,inf_r.%

|va||lo|,%%

abst99. ## Mit Abstandsangabe für die Ebene 99

use = zp,qu,znr,za,

for va = lok,statu,ps,pz,isd,izd,ism,izm,idz,id,ges,sges,dz_sb,i_zb,

inf,ps_rel,s_rel,pz_r,isd_r,izd_r,ism_r,izm_r,idz_r,

id_r,sges_r,i_zb_r,inf_r,dges.%

for lo = 0 to 9.% ## Lokalisation

|va||lo|,%%

for va = ps,pz,isd,izd,ism,izm,idz,id,ges,sges,dz_sb,i_zb,

inf,ps_rel,s_rel,pz_r,isd_r,izd_r,ism_r,izm_r,idz_r,

id_r,sges_r,i_zb_r,inf_r,dges.%

ST|va|,%

for va = ps,id,isd,ism,sges,inf.%

ST|va|r2,%

.

/trans use = ((znr ge 7) AND (znr le 10)).

lok9 = abst99. ## Abstandsangabe für die Ebene 99

# Umrechnung der relativen Werte in Prozentwerte

for va = ps_rel,s_rel,pz_r,isd_r,izd_r,ism_r,izm_r,idz_r,

id_r,sges_r,i_zb_r,inf_r.%

for lo = 0 to 9.%

|va||lo| = |va||lo| * 100.%%

# Berechnung der Flächenanteile ohne aeusseren Zement

for va = ps,id,isd,ism,sges,inf.%

for lo = 0 to 9.%

|va|_r2|lo| = |va||lo|/(ges|lo|-pz|lo|) * 100.%%

# Berechnung der Gesamtflaeche abzüglich peripherem Zement

for lo = 0 to 9.%

dges|lo| = ges|lo|-pz|lo|.%

# Berechnung der Steigung "ST" ueber die Schnittebenen

for va = ps,pz,isd,izd,ism,izm,idz,id,ges,sges,dz_sb,i_zb,

inf,ps_rel,s_rel,pz_r,isd_r,izd_r,ism_r,izm_r,idz_r,

id_r,sges_r,i_zb_r,inf_r,dges.%

ST|va| = b(for lo = 0 to 9.% lok|lo|,|va||lo|,%).

%

for va = ps,id,isd,ism,sges,inf.%

ST|va|r2 = b(for lo = 0 to 9.% lok|lo|,|va|_r2|lo|,%).

%

/onegroup

var = for va = ps,pz,isd,izd,ism,izm,idz,id,ges,sges,dz_sb,i_zb,

inf,ps_rel,s_rel,pz_r,isd_r,izd_r,ism_r,izm_r,idz_r,

id_r,sges_r,i_zb_r,inf_r,dges.%

ST|va|,%

for va = ps,id,isd,ism,sges,inf.%

ST|va|r2,%.

/print level = min.

case = 0.

/end

--- PROGRAM INSTRUCTIONS AFTER "FOR %" EXPANSION ---

/prob title = 'Herr Lauritz Englisch: A3D2.inp *** Quantitative Studien zum

Schmelzgehalt in Pferdebackenzaehnen.

- 1 Fall = 1 Zahn = 10 Lokalisationen =

10 Zeilen - Hier: Oberkiefer,

Zahn 7 - 10 - Umrechnung der relativen Werte in Prozentwerte -

Berechnung der Flächenanteile ohne aeusseren Zement - mit Berechnung

der Steigung "ST" ueber die Schnittebenen - Einstichproben-t-Test

gegen H0: ST = 0 ***'.

/inp var = 276. file = a. format = '32f10,9(/50x,27f10),

/20x,1f10'.

/var names = zahnid,zp,qu,znr,za, lok0, statu0, ps0, pz0, isd0, izd0, ism0,

izm0, idz0, id0, ges0, sges0, dz_sb0, i_zb0, inf0, ps_rel0, s_rel0,

pz_r0, isd_r0, izd_r0, ism_r0, izm_r0, idz_r0, id_r0, sges_r0,

i_zb_r0, inf_r0, lok1, statu1, ps1, pz1, isd1, izd1, ism1, izm1,

idz1, id1, ges1, sges1, dz_sb1, i_zb1, inf1, ps_rel1, s_rel1, pz_r1,

isd_r1, izd_r1, ism_r1, izm_r1, idz_r1, id_r1, sges_r1, i_zb_r1,

inf_r1, lok2, statu2, ps2, pz2, isd2, izd2, ism2, izm2, idz2, id2,

ges2, sges2, dz_sb2, i_zb2, inf2, ps_rel2, s_rel2, pz_r2, isd_r2,

izd_r2, ism_r2, izm_r2, idz_r2, id_r2, sges_r2, i_zb_r2, inf_r2,

lok3, statu3, ps3, pz3, isd3, izd3, ism3, izm3, idz3, id3, ges3,

sges3, dz_sb3, i_zb3, inf3, ps_rel3, s_rel3, pz_r3, isd_r3, izd_r3,

ism_r3, izm_r3, idz_r3, id_r3, sges_r3, i_zb_r3, inf_r3, lok4,

statu4, ps4, pz4, isd4, izd4, ism4, izm4, idz4, id4, ges4, sges4,

dz_sb4, i_zb4, inf4, ps_rel4, s_rel4, pz_r4, isd_r4, izd_r4, ism_r4,

izm_r4, idz_r4, id_r4, sges_r4, i_zb_r4, inf_r4, lok5, statu5,

ps5, pz5, isd5, izd5, ism5, izm5, idz5, id5, ges5, sges5, dz_sb5,

i_zb5, inf5, ps_rel5, s_rel5, pz_r5, isd_r5, izd_r5, ism_r5, izm_r5,

idz_r5, id_r5, sges_r5, i_zb_r5, inf_r5, lok6, statu6, ps6, pz6,

isd6, izd6, ism6, izm6, idz6, id6, ges6, sges6, dz_sb6, i_zb6, inf6,

ps_rel6, s_rel6, pz_r6, isd_r6, izd_r6, ism_r6, izm_r6, idz_r6,

id_r6, sges_r6, i_zb_r6, inf_r6, lok7, statu7, ps7, pz7, isd7,

izd7, ism7, izm7, idz7, id7, ges7, sges7, dz_sb7, i_zb7, inf7,

ps_rel7, s_rel7, pz_r7, isd_r7, izd_r7, ism_r7, izm_r7, idz_r7,

id_r7, sges_r7, i_zb_r7, inf_r7, lok8, statu8, ps8, pz8, isd8,

izd8, ism8, izm8, idz8, id8, ges8, sges8, dz_sb8, i_zb8, inf8,

ps_rel8, s_rel8, pz_r8, isd_r8, izd_r8, ism_r8, izm_r8, idz_r8,

id_r8, sges_r8, i_zb_r8, inf_r8, lok9, statu9, ps9, pz9, isd9,

izd9, ism9, izm9, idz9, id9, ges9, sges9, dz_sb9, i_zb9, inf9,

ps_rel9, s_rel9, pz_r9, isd_r9, izd_r9, ism_r9, izm_r9, idz_r9,

id_r9, sges_r9, i_zb_r9, inf_r9, abst99.

use = zp,qu,znr,za, lok0, lok1, lok2, lok3, lok4, lok5, lok6,

lok7, lok8, lok9, statu0, statu1, statu2, statu3, statu4,

statu5, statu6, statu7, statu8, statu9, ps0, ps1, ps2,

ps3, ps4, ps5, ps6, ps7, ps8, ps9, pz0, pz1, pz2, pz3,

pz4, pz5, pz6, pz7, pz8, pz9, isd0, isd1, isd2, isd3,

isd4, isd5, isd6, isd7, isd8, isd9, izd0, izd1, izd2,

izd3, izd4, izd5, izd6, izd7, izd8, izd9, ism0, ism1,

ism2, ism3, ism4, ism5, ism6, ism7, ism8, ism9, izm0,

izm1, izm2, izm3, izm4, izm5, izm6, izm7, izm8, izm9,

idz0, idz1, idz2, idz3, idz4, idz5, idz6, idz7, idz8,

idz9, id0, id1, id2, id3, id4, id5, id6, id7, id8, id9,

ges0, ges1, ges2, ges3, ges4, ges5, ges6, ges7, ges8,

ges9, sges0, sges1, sges2, sges3, sges4, sges5, sges6,

sges7, sges8, sges9, dz_sb0, dz_sb1, dz_sb2, dz_sb3,

dz_sb4, dz_sb5, dz_sb6, dz_sb7, dz_sb8, dz_sb9, i_zb0,

i_zb1, i_zb2, i_zb3, i_zb4, i_zb5, i_zb6, i_zb7, i_zb8,

i_zb9, inf0, inf1, inf2, inf3, inf4, inf5, inf6, inf7,

inf8, inf9, ps_rel0, ps_rel1, ps_rel2, ps_rel3, ps_rel4,

ps_rel5, ps_rel6, ps_rel7, ps_rel8, ps_rel9, s_rel0, s_rel1,

s_rel2, s_rel3, s_rel4, s_rel5, s_rel6, s_rel7, s_rel8,

s_rel9, pz_r0, pz_r1, pz_r2, pz_r3, pz_r4, pz_r5, pz_r6,

pz_r7, pz_r8, pz_r9, isd_r0, isd_r1, isd_r2, isd_r3,

isd_r4, isd_r5, isd_r6, isd_r7, isd_r8, isd_r9, izd_r0,

izd_r1, izd_r2, izd_r3, izd_r4, izd_r5, izd_r6, izd_r7,

izd_r8, izd_r9, ism_r0, ism_r1, ism_r2, ism_r3, ism_r4,

ism_r5, ism_r6, ism_r7, ism_r8, ism_r9, izm_r0, izm_r1,

izm_r2, izm_r3, izm_r4, izm_r5, izm_r6, izm_r7, izm_r8,

izm_r9, idz_r0, idz_r1, idz_r2, idz_r3, idz_r4, idz_r5,

idz_r6, idz_r7, idz_r8, idz_r9, id_r0, id_r1, id_r2, id_r3,

id_r4, id_r5, id_r6, id_r7, id_r8, id_r9, sges_r0, sges_r1,

sges_r2, sges_r3, sges_r4, sges_r5, sges_r6, sges_r7,

sges_r8, sges_r9, i_zb_r0, i_zb_r1, i_zb_r2, i_zb_r3,

i_zb_r4, i_zb_r5, i_zb_r6, i_zb_r7, i_zb_r8, i_zb_r9,

inf_r0, inf_r1, inf_r2, inf_r3, inf_r4, inf_r5, inf_r6,

inf_r7, inf_r8, inf_r9, dges0, dges1, dges2, dges3, dges4,

dges5, dges6, dges7, dges8, dges9, STps, STpz, STisd, STizd,

STism, STizm, STidz, STid, STges, STsges, STdz_sb, STi_zb, STinf,

STps_rel, STs_rel, STpz_r, STisd_r, STizd_r, STism_r, STizm_r,

STidz_r, STid_r, STsges_r, STi_zb_r, STinf_r, STdges, STpsr2,

STidr2, STisdr2, STismr2, STsgesr2, STinfr2 .

/trans use = ((znr ge 7) AND (znr le 10)). lok9 = abst99.

ps_rel0 = ps_rel0 * 100. ps_rel1 = ps_rel1 * 100.

ps_rel2 = ps_rel2 * 100. ps_rel3 = ps_rel3 * 100.

ps_rel4 = ps_rel4 * 100. ps_rel5 = ps_rel5 * 100.

ps_rel6 = ps_rel6 * 100. ps_rel7 = ps_rel7 * 100.

ps_rel8 = ps_rel8 * 100. ps_rel9 = ps_rel9 * 100. s_rel0 = s_rel0 * 100.

s_rel1 = s_rel1 * 100. s_rel2 = s_rel2 * 100. s_rel3 = s_rel3 * 100.

s_rel4 = s_rel4 * 100. s_rel5 = s_rel5 * 100. s_rel6 = s_rel6 * 100.

s_rel7 = s_rel7 * 100. s_rel8 = s_rel8 * 100. s_rel9 = s_rel9 * 100.

pz_r0 = pz_r0 * 100. pz_r1 = pz_r1 * 100. pz_r2 = pz_r2 * 100.

pz_r3 = pz_r3 * 100. pz_r4 = pz_r4 * 100. pz_r5 = pz_r5 * 100.

pz_r6 = pz_r6 * 100. pz_r7 = pz_r7 * 100. pz_r8 = pz_r8 * 100.

pz_r9 = pz_r9 * 100. isd_r0 = isd_r0 * 100. isd_r1 = isd_r1 * 100.

isd_r2 = isd_r2 * 100. isd_r3 = isd_r3 * 100. isd_r4 = isd_r4 * 100.

isd_r5 = isd_r5 * 100. isd_r6 = isd_r6 * 100. isd_r7 = isd_r7 * 100.

isd_r8 = isd_r8 * 100. isd_r9 = isd_r9 * 100. izd_r0 = izd_r0 * 100.

izd_r1 = izd_r1 * 100. izd_r2 = izd_r2 * 100. izd_r3 = izd_r3 * 100.

izd_r4 = izd_r4 * 100. izd_r5 = izd_r5 * 100. izd_r6 = izd_r6 * 100.

izd_r7 = izd_r7 * 100. izd_r8 = izd_r8 * 100. izd_r9 = izd_r9 * 100.

ism_r0 = ism_r0 * 100. ism_r1 = ism_r1 * 100. ism_r2 = ism_r2 * 100.

ism_r3 = ism_r3 * 100. ism_r4 = ism_r4 * 100. ism_r5 = ism_r5 * 100.

ism_r6 = ism_r6 * 100. ism_r7 = ism_r7 * 100. ism_r8 = ism_r8 * 100.

ism_r9 = ism_r9 * 100. izm_r0 = izm_r0 * 100. izm_r1 = izm_r1 * 100.

izm_r2 = izm_r2 * 100. izm_r3 = izm_r3 * 100. izm_r4 = izm_r4 * 100.

izm_r5 = izm_r5 * 100. izm_r6 = izm_r6 * 100. izm_r7 = izm_r7 * 100.

izm_r8 = izm_r8 * 100. izm_r9 = izm_r9 * 100. idz_r0 = idz_r0 * 100.

idz_r1 = idz_r1 * 100. idz_r2 = idz_r2 * 100. idz_r3 = idz_r3 * 100.

idz_r4 = idz_r4 * 100. idz_r5 = idz_r5 * 100. idz_r6 = idz_r6 * 100.

idz_r7 = idz_r7 * 100. idz_r8 = idz_r8 * 100. idz_r9 = idz_r9 * 100.

id_r0 = id_r0 * 100. id_r1 = id_r1 * 100. id_r2 = id_r2 * 100.

id_r3 = id_r3 * 100. id_r4 = id_r4 * 100. id_r5 = id_r5 * 100.

id_r6 = id_r6 * 100. id_r7 = id_r7 * 100. id_r8 = id_r8 * 100.

id_r9 = id_r9 * 100. sges_r0 = sges_r0 * 100. sges_r1 = sges_r1 * 100.

sges_r2 = sges_r2 * 100. sges_r3 = sges_r3 * 100.

sges_r4 = sges_r4 * 100. sges_r5 = sges_r5 * 100.

sges_r6 = sges_r6 * 100. sges_r7 = sges_r7 * 100.

sges_r8 = sges_r8 * 100. sges_r9 = sges_r9 * 100.

i_zb_r0 = i_zb_r0 * 100. i_zb_r1 = i_zb_r1 * 100.

i_zb_r2 = i_zb_r2 * 100. i_zb_r3 = i_zb_r3 * 100.

i_zb_r4 = i_zb_r4 * 100. i_zb_r5 = i_zb_r5 * 100.

i_zb_r6 = i_zb_r6 * 100. i_zb_r7 = i_zb_r7 * 100.

i_zb_r8 = i_zb_r8 * 100. i_zb_r9 = i_zb_r9 * 100. inf_r0 = inf_r0 * 100.

inf_r1 = inf_r1 * 100. inf_r2 = inf_r2 * 100. inf_r3 = inf_r3 * 100.

inf_r4 = inf_r4 * 100. inf_r5 = inf_r5 * 100. inf_r6 = inf_r6 * 100.

inf_r7 = inf_r7 * 100. inf_r8 = inf_r8 * 100. inf_r9 = inf_r9 * 100.

ps_r20 = ps0/(ges0-pz0) * 100. ps_r21 = ps1/(ges1-pz1) * 100.

ps_r22 = ps2/(ges2-pz2) * 100. ps_r23 = ps3/(ges3-pz3) * 100.

ps_r24 = ps4/(ges4-pz4) * 100. ps_r25 = ps5/(ges5-pz5) * 100.

ps_r26 = ps6/(ges6-pz6) * 100. ps_r27 = ps7/(ges7-pz7) * 100.

ps_r28 = ps8/(ges8-pz8) * 100. ps_r29 = ps9/(ges9-pz9) * 100.

id_r20 = id0/(ges0-pz0) * 100. id_r21 = id1/(ges1-pz1) * 100.

id_r22 = id2/(ges2-pz2) * 100. id_r23 = id3/(ges3-pz3) * 100.

id_r24 = id4/(ges4-pz4) * 100. id_r25 = id5/(ges5-pz5) * 100.

id_r26 = id6/(ges6-pz6) * 100. id_r27 = id7/(ges7-pz7) * 100.

id_r28 = id8/(ges8-pz8) * 100. id_r29 = id9/(ges9-pz9) * 100.

isd_r20 = isd0/(ges0-pz0) * 100. isd_r21 = isd1/(ges1-pz1) * 100.

isd_r22 = isd2/(ges2-pz2) * 100. isd_r23 = isd3/(ges3-pz3) * 100.

isd_r24 = isd4/(ges4-pz4) * 100. isd_r25 = isd5/(ges5-pz5) * 100.

isd_r26 = isd6/(ges6-pz6) * 100. isd_r27 = isd7/(ges7-pz7) * 100.

isd_r28 = isd8/(ges8-pz8) * 100. isd_r29 = isd9/(ges9-pz9) * 100.

ism_r20 = ism0/(ges0-pz0) * 100. ism_r21 = ism1/(ges1-pz1) * 100.

ism_r22 = ism2/(ges2-pz2) * 100. ism_r23 = ism3/(ges3-pz3) * 100.

ism_r24 = ism4/(ges4-pz4) * 100. ism_r25 = ism5/(ges5-pz5) * 100.

ism_r26 = ism6/(ges6-pz6) * 100. ism_r27 = ism7/(ges7-pz7) * 100.

ism_r28 = ism8/(ges8-pz8) * 100. ism_r29 = ism9/(ges9-pz9) * 100.

sges_r20 = sges0/(ges0-pz0) * 100. sges_r21 = sges1/(ges1-pz1) * 100.

sges_r22 = sges2/(ges2-pz2) * 100. sges_r23 = sges3/(ges3-pz3) * 100.

sges_r24 = sges4/(ges4-pz4) * 100. sges_r25 = sges5/(ges5-pz5) * 100.

sges_r26 = sges6/(ges6-pz6) * 100. sges_r27 = sges7/(ges7-pz7) * 100.

sges_r28 = sges8/(ges8-pz8) * 100. sges_r29 = sges9/(ges9-pz9) * 100.

inf_r20 = inf0/(ges0-pz0) * 100. inf_r21 = inf1/(ges1-pz1) * 100.

inf_r22 = inf2/(ges2-pz2) * 100. inf_r23 = inf3/(ges3-pz3) * 100.

inf_r24 = inf4/(ges4-pz4) * 100. inf_r25 = inf5/(ges5-pz5) * 100.

inf_r26 = inf6/(ges6-pz6) * 100. inf_r27 = inf7/(ges7-pz7) * 100.

inf_r28 = inf8/(ges8-pz8) * 100. inf_r29 = inf9/(ges9-pz9) * 100.

dges0 = ges0-pz0. dges1 = ges1-pz1. dges2 = ges2-pz2. dges3 = ges3-pz3.

dges4 = ges4-pz4. dges5 = ges5-pz5. dges6 = ges6-pz6. dges7 = ges7-pz7.

dges8 = ges8-pz8. dges9 = ges9-pz9.

STps = b( lok0,ps0, lok1,ps1, lok2,ps2, lok3,ps3, lok4,ps4, lok5,ps5,

lok6,ps6, lok7,ps7, lok8,ps8, lok9,ps9).

STpz = b( lok0,pz0, lok1,pz1, lok2,pz2, lok3,pz3, lok4,pz4, lok5,pz5,

lok6,pz6, lok7,pz7, lok8,pz8, lok9,pz9).

STisd = b( lok0,isd0, lok1,isd1, lok2,isd2, lok3,isd3, lok4,isd4, lok5,

isd5, lok6,isd6, lok7,isd7, lok8,isd8, lok9,isd9).

STizd = b( lok0,izd0, lok1,izd1, lok2,izd2, lok3,izd3, lok4,izd4, lok5,

izd5, lok6,izd6, lok7,izd7, lok8,izd8, lok9,izd9).

STism = b( lok0,ism0, lok1,ism1, lok2,ism2, lok3,ism3, lok4,ism4, lok5,

ism5, lok6,ism6, lok7,ism7, lok8,ism8, lok9,ism9).

STizm = b( lok0,izm0, lok1,izm1, lok2,izm2, lok3,izm3, lok4,izm4, lok5,

izm5, lok6,izm6, lok7,izm7, lok8,izm8, lok9,izm9).

STidz = b( lok0,idz0, lok1,idz1, lok2,idz2, lok3,idz3, lok4,idz4, lok5,

idz5, lok6,idz6, lok7,idz7, lok8,idz8, lok9,idz9).

STid = b( lok0,id0, lok1,id1, lok2,id2, lok3,id3, lok4,id4, lok5,id5,

lok6,id6, lok7,id7, lok8,id8, lok9,id9).

STges = b( lok0,ges0, lok1,ges1, lok2,ges2, lok3,ges3, lok4,ges4, lok5,

ges5, lok6,ges6, lok7,ges7, lok8,ges8, lok9,ges9).

STsges = b( lok0,sges0, lok1,sges1, lok2,sges2, lok3,sges3, lok4,sges4,

lok5,sges5, lok6,sges6, lok7,sges7, lok8,sges8, lok9,sges9).

STdz_sb = b( lok0,dz_sb0, lok1,dz_sb1, lok2,dz_sb2, lok3,dz_sb3, lok4,

dz_sb4, lok5,dz_sb5, lok6,dz_sb6, lok7,dz_sb7, lok8,dz_sb8, lok9,

dz_sb9). STi_zb = b( lok0,i_zb0, lok1,i_zb1, lok2,i_zb2, lok3,i_zb3,

lok4,i_zb4, lok5,i_zb5, lok6,i_zb6, lok7,i_zb7, lok8,i_zb8, lok9,

i_zb9). STinf = b( lok0,inf0, lok1,inf1, lok2,inf2, lok3,inf3, lok4,

inf4, lok5,inf5, lok6,inf6, lok7,inf7, lok8,inf8, lok9,inf9).

STps_rel = b( lok0,ps_rel0, lok1,ps_rel1, lok2,ps_rel2, lok3,ps_rel3,

lok4,ps_rel4, lok5,ps_rel5, lok6,ps_rel6, lok7,ps_rel7, lok8,

ps_rel8, lok9,ps_rel9).

STs_rel = b( lok0,s_rel0, lok1,s_rel1, lok2,s_rel2, lok3,s_rel3, lok4,

s_rel4, lok5,s_rel5, lok6,s_rel6, lok7,s_rel7, lok8,s_rel8, lok9,

s_rel9). STpz_r = b( lok0,pz_r0, lok1,pz_r1, lok2,pz_r2, lok3,pz_r3,

lok4,pz_r4, lok5,pz_r5, lok6,pz_r6, lok7,pz_r7, lok8,pz_r8, lok9,

pz_r9). STisd_r = b( lok0,isd_r0, lok1,isd_r1, lok2,isd_r2, lok3,

isd_r3, lok4,isd_r4, lok5,isd_r5, lok6,isd_r6, lok7,isd_r7, lok8,

isd_r8, lok9,isd_r9).

STizd_r = b( lok0,izd_r0, lok1,izd_r1, lok2,izd_r2, lok3,izd_r3, lok4,

izd_r4, lok5,izd_r5, lok6,izd_r6, lok7,izd_r7, lok8,izd_r8, lok9,

izd_r9). STism_r = b( lok0,ism_r0, lok1,ism_r1, lok2,ism_r2, lok3,

ism_r3, lok4,ism_r4, lok5,ism_r5, lok6,ism_r6, lok7,ism_r7, lok8,

ism_r8, lok9,ism_r9).

STizm_r = b( lok0,izm_r0, lok1,izm_r1, lok2,izm_r2, lok3,izm_r3, lok4,

izm_r4, lok5,izm_r5, lok6,izm_r6, lok7,izm_r7, lok8,izm_r8, lok9,

izm_r9). STidz_r = b( lok0,idz_r0, lok1,idz_r1, lok2,idz_r2, lok3,

idz_r3, lok4,idz_r4, lok5,idz_r5, lok6,idz_r6, lok7,idz_r7, lok8,

idz_r8, lok9,idz_r9).

STid_r = b( lok0,id_r0, lok1,id_r1, lok2,id_r2, lok3,id_r3, lok4,id_r4,

lok5,id_r5, lok6,id_r6, lok7,id_r7, lok8,id_r8, lok9,id_r9).

STsges_r = b( lok0,sges_r0, lok1,sges_r1, lok2,sges_r2, lok3,sges_r3,

lok4,sges_r4, lok5,sges_r5, lok6,sges_r6, lok7,sges_r7, lok8,

sges_r8, lok9,sges_r9).

STi_zb_r = b( lok0,i_zb_r0, lok1,i_zb_r1, lok2,i_zb_r2, lok3,i_zb_r3,

lok4,i_zb_r4, lok5,i_zb_r5, lok6,i_zb_r6, lok7,i_zb_r7, lok8,

i_zb_r8, lok9,i_zb_r9).

STinf_r = b( lok0,inf_r0, lok1,inf_r1, lok2,inf_r2, lok3,inf_r3, lok4,

inf_r4, lok5,inf_r5, lok6,inf_r6, lok7,inf_r7, lok8,inf_r8, lok9,

inf_r9). STdges = b( lok0,dges0, lok1,dges1, lok2,dges2, lok3,dges3,

lok4,dges4, lok5,dges5, lok6,dges6, lok7,dges7, lok8,dges8, lok9,

dges9). STpsr2 = b( lok0,ps_r20, lok1,ps_r21, lok2,ps_r22, lok3,

ps_r23, lok4,ps_r24, lok5,ps_r25, lok6,ps_r26, lok7,ps_r27, lok8,

ps_r28, lok9,ps_r29).

STidr2 = b( lok0,id_r20, lok1,id_r21, lok2,id_r22, lok3,id_r23, lok4,

id_r24, lok5,id_r25, lok6,id_r26, lok7,id_r27, lok8,id_r28, lok9,

id_r29). STisdr2 = b( lok0,isd_r20, lok1,isd_r21, lok2,isd_r22,

lok3,isd_r23, lok4,isd_r24, lok5,isd_r25, lok6,isd_r26, lok7,

isd_r27, lok8,isd_r28, lok9,isd_r29).

STismr2 = b( lok0,ism_r20, lok1,ism_r21, lok2,ism_r22, lok3,ism_r23,

lok4,ism_r24, lok5,ism_r25, lok6,ism_r26, lok7,ism_r27, lok8,

ism_r28, lok9,ism_r29).

STsgesr2 = b( lok0,sges_r20, lok1,sges_r21, lok2,sges_r22, lok3,sges_r23,

lok4,sges_r24, lok5,sges_r25, lok6,sges_r26, lok7,sges_r27, lok8,

sges_r28, lok9,sges_r29).

STinfr2 = b( lok0,inf_r20, lok1,inf_r21, lok2,inf_r22, lok3,inf_r23,

lok4,inf_r24, lok5,inf_r25, lok6,inf_r26, lok7,inf_r27, lok8,

inf_r28, lok9,inf_r29).

/onegroup var = STps, STpz, STisd, STizd, STism, STizm, STidz, STid, STges,

STsges, STdz_sb, STi_zb, STinf, STps_rel, STs_rel, STpz_r, STisd_r,

STizd_r, STism_r, STizm_r, STidz_r, STid_r, STsges_r, STi_zb_r,

STinf_r, STdges, STpsr2, STidr2, STisdr2, STismr2, STsgesr2,

STinfr2.

/print level = min. case = 0.

/end/

NUMBER OF CASES READ. . . . . . . . . . . . . . 28

CASES WITH USE SET TO ZERO . . . . . . . . . 8

REMAINING NUMBER OF CASES . . . . . . . . 20

DESCRIPTIVE STATISTICS OF DATA

----------- ---------- -- ----

VARIABLE TOTAL STANDARD ST.ERR COEFF S M A L L E S T L A R G E S T

NO. NAME FREQ. MEAN DEV. OF MEAN OF VAR VALUE Z-SCR CASE VALUE Z-SCR CASE RANGE

2 zp 20 148.50 50.380 11.265 .33926 107.00 -0.82 6 210.00 1.22 2 103.00

3 qu 20 1.4000 .50262 .11239 .35902 1.0000 -0.80 1 2.0000 1.19 2 1.0000

4 znr 20 8.5000 1.1471 .25649 .13495 7.0000 -1.31 6 10.000 1.31 2 3.0000

5 za 20 10.525 5.7181 1.2786 .54329 1.0000 -1.67 4 20.000 1.66 1 19.000

6 lok0 20 0.0000 0.0000 0.0000 0.0000 1 0.0000 1 0.0000

33 lok1 20 10.000 0.0000 0.0000 0.0000 10.000 1 10.000 1 0.0000

60 lok2 20 20.000 0.0000 0.0000 0.0000 20.000 1 20.000 1 0.0000

87 lok3 20 30.000 0.0000 0.0000 0.0000 30.000 1 30.000 1 0.0000

114 lok4 20 40.000 0.0000 0.0000 0.0000 40.000 1 40.000 1 0.0000

141 lok5 20 50.000 0.0000 0.0000 0.0000 50.000 1 50.000 1 0.0000

168 lok6 20 60.000 0.0000 0.0000 0.0000 60.000 1 60.000 1 0.0000

195 lok7 20 70.000 0.0000 0.0000 0.0000 70.000 1 70.000 1 0.0000

222 lok8 20 80.000 0.0000 0.0000 0.0000 80.000 1 80.000 1 0.0000

249 lok9 20 38.955 13.905 3.1093 .35696 21.910 -1.23 16 62.490 1.69 5 40.580

7 statu0 20 1.0000 0.0000 0.0000 0.0000 1.0000 1 1.0000 1 0.0000

34 statu1 20 1.3500 .48936 .10942 .36249 1.0000 -0.72 3 2.0000 1.33 1 1.0000

61 statu2 20 1.9500 .94451 .21120 .48437 1.0000 -1.01 4 3.0000 1.11 1 2.0000

88 statu3 20 2.2000 .95145 .21275 .43248 1.0000 -1.26 4 3.0000 0.84 1 2.0000

115 statu4 20 2.5500 .75915 .16975 .29771 1.0000 -2.04 4 3.0000 0.59 1 2.0000

142 statu5 20 2.8500 .48936 .10942 .17171 1.0000 -3.78 5 3.0000 0.31 1 2.0000

169 statu6 20 3.0000 0.0000 0.0000 0.0000 3.0000 1 3.0000 1 0.0000

196 statu7 20 3.0000 0.0000 0.0000 0.0000 3.0000 1 3.0000 1 0.0000

223 statu8 20 3.0000 0.0000 0.0000 0.0000 3.0000 1 3.0000 1 0.0000

250 statu9 20 3.0000 0.0000 0.0000 0.0000 3.0000 1 3.0000 1 0.0000

8 ps0 19 91.440 19.876 4.5600 .21737 61.020 -1.53 2 126.63 1.77 10 65.610

35 ps1 19 109.67 17.667 4.0530 .16108 80.020 -1.68 28 140.16 1.73 10 60.140

62 ps2 19 105.02 14.384 3.2999 .13697 81.630 -1.63 28 130.06 1.74 9 48.430

89 ps3 9 107.33 14.584 4.8612 .13588 88.040 -1.32 7 139.81 2.23 9 51.770

116 ps4 9 104.03 13.034 4.3446 .12528 86.670 -1.33 11 125.62 1.66 9 38.950

143 ps5 5 99.996 6.7903 3.0367 .06791 93.090 -1.02 19 109.45 1.39 4 16.360

170 ps6 2 110.14 1.1950 .84500 .01085 109.29 -0.71 5 110.98 0.71 18 1.6900

197 ps7 0

224 ps8 0

251 ps9 20 100.38 14.910 3.3340 .14853 72.580 -1.86 28 123.85 1.57 9 51.270

9 pz0 19 27.856 12.064 2.7676 .43307 10.680 -1.42 5 57.830 2.48 10 47.150

36 pz1 19 76.448 40.825 9.3659 .53402 30.350 -1.13 18 165.71 2.19 10 135.36

63 pz2 19 104.01 59.192 13.580 .56912 33.970 -1.18 18 201.47 1.65 10 167.50

90 pz3 9 62.862 29.859 9.9528 .47498 36.950 -0.87 19 133.22 2.36 11 96.270

117 pz4 9 107.63 49.818 16.606 .46284 43.970 -1.28 18 180.19 1.46 7 136.22

144 pz5 5 133.81 61.615 27.555 .46047 67.240 -1.08 18 206.04 1.17 25 138.80

171 pz6 2 144.55 8.2095 5.8050 .05679 138.75 -0.71 18 150.36 0.71 5 11.610

198 pz7 0

225 pz8 0

252 pz9 20 143.61 33.160 7.4148 .23090 58.230 -2.57 8 185.52 1.26 17 127.29

10 isd0 2 17.975 11.646 8.2350 .64790 9.7400 -0.71 10 26.210 0.71 25 16.470

37 isd1 19 19.391 5.5500 1.2732 .28621 9.0600 -1.86 3 28.860 1.71 9 19.800

64 isd2 19 23.437 4.1787 .95867 .17830 16.330 -1.70 27 30.330 1.65 9 14.000

91 isd3 9 26.367 4.5894 1.5298 .17406 20.620 -1.25 5 34.060 1.68 9 13.440

118 isd4 9 25.148 5.3570 1.7857 .21302 18.380 -1.26 5 32.790 1.43 6 14.410

145 isd5 5 25.024 4.2302 1.8918 .16904 22.010 -0.71 5 32.170 1.69 25 10.160

172 isd6 2 27.405 1.0394 .73500 .03793 26.670 -0.71 5 28.140 0.71 18 1.4700

199 isd7 0

226 isd8 0

253 isd9 20 25.475 5.8267 1.3029 .22873 12.740 -2.19 8 38.880 2.30 9 26.140

11 izd0 2 17.375 8.6479 6.1150 .49772 11.260 -0.71 10 23.490 0.71 25 12.230

38 izd1 18 32.674 11.566 2.7262 .35398 18.500 -1.23 27 57.470 2.14 5 38.970

65 izd2 18 39.324 11.196 2.6390 .28472 27.020 -1.10 8 67.690 2.53 4 40.670

92 izd3 9 45.081 7.1701 2.3900 .15905 35.300 -1.36 11 56.420 1.58 5 21.120

119 izd4 9 48.486 8.0586 2.6862 .16621 36.650 -1.47 11 60.170 1.45 5 23.520

146 izd5 5 51.936 7.3663 3.2943 .14183 42.930 -1.22 18 60.070 1.10 5 17.140

173 izd6 2 55.440 12.756 9.0200 .23009 46.420 -0.71 18 64.460 0.71 5 18.040

200 izd7 0

227 izd8 0

254 izd9 19 43.121 11.548 2.6492 .26779 18.890 -2.10 8 67.430 2.11 5 48.540

12 ism0 2 25.075 9.9207 7.0150 .39564 18.060 -0.71 10 32.090 0.71 25 14.030

39 ism1 16 19.428 7.0617 1.7654 .36348 7.0200 -1.76 22 30.200 1.53 25 23.180

66 ism2 18 23.761 5.7375 1.3524 .24147 12.340 -1.99 3 35.950 2.12 9 23.610

93 ism3 9 27.048 5.4905 1.8302 .20299 19.820 -1.32 4 35.990 1.63 25 16.170

120 ism4 9 28.280 5.1347 1.7116 .18157 22.980 -1.03 5 37.670 1.83 9 14.690

147 ism5 5 29.516 3.9994 1.7886 .13550 25.220 -1.07 5 36.090 1.64 25 10.870

174 ism6 2 28.525 2.0577 1.4550 .07214 27.070 -0.71 5 29.980 0.71 18 2.9100

201 ism7 0

228 ism8 0

255 ism9 19 27.346 6.9131 1.5860 .25280 8.1300 -2.78 8 35.520 1.18 6 27.390

13 izm0 2 29.445 14.545 10.285 .49398 19.160 -0.71 10 39.730 0.71 25 20.570

40 izm1 16 34.914 16.757 4.1892 .47994 11.600 -1.39 11 70.430 2.12 18 58.830

67 izm2 17 42.056 15.164 3.6778 .36056 24.110 -1.18 8 87.670 3.01 5 63.560

94 izm3 9 47.191 13.019 4.3396 .27588 30.040 -1.32 11 77.700 2.34 5 47.660

121 izm4 9 50.382 9.9108 3.3036 .19671 34.730 -1.58 11 65.400 1.52 5 30.670

148 izm5 5 53.678 9.5213 4.2580 .17738 44.420 -0.97 19 67.150 1.41 5 22.730

175 izm6 2 67.620 17.706 12.520 .26184 55.100 -0.71 18 80.140 0.71 5 25.040

202 izm7 0

229 izm8 0

256 izm9 19 45.087 15.460 3.5468 .34290 10.740 -2.22 8 79.630 2.23 5 68.890

14 idz0 16 408.09 64.338 16.085 .15766 268.83 -2.16 27 532.84 1.94 1 264.01

41 idz1 3 428.54 64.107 37.012 .14959 374.97 -0.84 19 499.57 1.11 1 124.60

68 idz2 2 419.08 62.282 44.040 .14862 375.04 -0.71 3 463.12 0.71 1 88.080

95 idz3 0

122 idz4 0

149 idz5 0

176 idz6 0

203 idz7 0

230 idz8 0

257 idz9 1 464.16 0.0000 0.0000 0.0000 464.16 1 464.16 1 0.0000

15 id0 3 371.88 34.456 19.893 .09265 332.15 -1.15 8 393.62 0.63 25 61.470

42 id1 15 307.15 42.389 10.945 .13801 217.86 -2.11 27 361.91 1.29 25 144.05

69 id2 16 296.72 37.677 9.4194 .12698 221.12 -2.01 27 347.10 1.34 10 125.98

96 id3 9 306.56 37.375 12.458 .12192 236.68 -1.87 11 352.81 1.24 9 116.13

123 id4 9 311.45 42.988 14.329 .13803 237.23 -1.73 11 376.13 1.50 9 138.90

150 id5 5 318.26 31.445 14.063 .09880 281.96 -1.15 18 347.51 0.93 5 65.550

177 id6 2 302.71 45.806 32.390 .15132 270.32 -0.71 18 335.10 0.71 5 64.780

204 id7 0

231 id8 0

258 id9 18 303.18 42.772 10.082 .14108 219.02 -1.97 27 381.43 1.83 9 162.41

16 ges0 19 531.13 84.211 19.319 .15855 364.46 -1.98 27 698.46 1.99 1 334.00

43 ges1 19 592.38 112.61 25.835 .19010 256.10 -2.99 28 769.83 1.58 10 513.73

70 ges2 19 623.84 108.77 24.953 .17435 310.19 -2.88 28 816.44 1.77 10 506.25

97 ges3 9 622.43 50.465 16.822 .08108 558.93 -1.26 18 713.23 1.80 9 154.30

124 ges4 9 675.41 95.434 31.811 .14130 557.10 -1.24 18 820.81 1.52 9 263.71

151 ges5 5 712.23 82.969 37.105 .11649 585.75 -1.52 18 811.21 1.19 25 225.46

178 ges6 2 736.38 80.186 56.700 .10889 679.68 -0.71 18 793.08 0.71 5 113.40

205 ges7 0

232 ges8 0

259 ges9 20 675.32 134.03 29.971 .19848 274.95 -2.99 28 837.13 1.21 9 562.18

17 sges0 19 95.972 30.246 6.9388 .31515 61.020 -1.16 2 180.76 2.80 25 119.74

44 sges1 19 145.43 26.976 6.1887 .18550 104.59 -1.51 28 191.73 1.72 10 87.140

71 sges2 19 150.97 22.395 5.1377 .14834 116.70 -1.53 28 196.33 2.03 9 79.630

98 sges3 9 160.74 22.288 7.4294 .13866 136.65 -1.08 5 204.78 1.98 9 68.130

125 sges4 9 157.46 21.960 7.3200 .13946 136.68 -0.95 5 195.15 1.72 9 58.470

152 sges5 5 154.54 12.362 5.5286 .08000 143.28 -0.91 5 172.88 1.48 25 29.600

179 sges6 2 166.07 4.2921 3.0350 .02585 163.03 -0.71 5 169.10 0.71 18 6.0700

206 sges7 0

233 sges8 0

260 sges9 20 151.84 24.166 5.4036 .15916 109.22 -1.76 8 197.56 1.89 9 88.340

18 dz_sb0 19 411.84 66.938 15.357 .16254 268.83 -2.14 27 532.84 1.81 1 264.01

45 dz_sb1 19 406.26 102.02 23.406 .25113 64.550 -3.35 28 509.58 1.01 1 445.03

72 dz_sb2 19 414.81 95.007 21.796 .22904 90.360 -3.42 28 498.70 0.88 25 408.34

99 dz_sb3 9 452.24 53.317 17.772 .11789 351.08 -1.90 11 510.37 1.09 9 159.29

126 dz_sb4 9 463.74 60.647 20.216 .13078 358.62 -1.73 11 539.28 1.25 9 180.66

153 dz_sb5 5 478.42 47.633 21.302 .09956 421.74 -1.19 18 521.97 0.91 5 100.23

180 dz_sb6 2 481.70 73.178 51.745 .15192 429.95 -0.71 18 533.44 0.71 5 103.49

207 dz_sb7 0

234 dz_sb8 0

261 dz_sb9 20 431.32 99.738 22.302 .23124 105.38 -3.27 28 558.56 1.28 9 453.18

19 i_zb0 6 15.607 26.309 10.740 1.6857 0.0000 -0.59 16 63.220 1.81 25 63.220

46 i_zb1 18 63.708 25.984 6.1245 .40786 20.620 -1.66 3 115.16 1.98 18 94.540

73 i_zb2 18 79.044 27.321 6.4396 .34564 27.670 -1.88 3 150.00 2.60 5 122.33

100 i_zb3 9 92.272 19.559 6.5196 .21197 65.340 -1.38 11 134.12 2.14 5 68.780

127 i_zb4 9 98.867 17.898 5.9659 .18103 71.380 -1.54 11 125.57 1.49 5 54.190

154 i_zb5 5 105.61 16.684 7.4611 .15797 88.320 -1.04 18 127.22 1.30 5 38.900

181 i_zb6 2 123.06 30.462 21.540 .24754 101.52 -0.71 18 144.60 0.71 5 43.080

208 i_zb7 0

235 i_zb8 0

262 i_zb9 19 88.209 26.547 6.0904 .30096 29.630 -2.21 8 147.06 2.22 5 117.43

20 inf0 7 25.679 47.510 17.957 1.8502 0.0000 -0.54 16 121.53 2.02 25 121.53

47 inf1 19 96.108 40.188 9.2197 .41815 10.010 -2.14 1 166.57 1.75 18 156.56

74 inf2 19 120.83 38.757 8.8915 .32075 18.340 -2.64 1 192.82 1.86 5 174.48

101 inf3 9 145.69 19.430 6.4767 .13337 114.40 -1.61 11 177.40 1.63 5 63.000

128 inf4 9 152.30 20.014 6.6714 .13142 121.40 -1.54 11 173.17 1.04 6 51.770

155 inf5 5 160.16 17.008 7.6064 .10620 139.78 -1.20 18 174.63 0.85 25 34.850

182 inf6 2 178.99 27.372 19.355 .15293 159.63 -0.71 18 198.34 0.71 5 38.710

209 inf7 0

236 inf8 0

263 inf9 20 135.25 44.370 9.9213 .32805 20.340 -2.59 1 203.33 1.53 5 182.99

21 ps_rel0 18 17.235 2.3821 .56147 .13821 11.363 -2.47 2 20.457 1.35 9 9.0941

48 ps_rel1 19 18.911 3.2957 .75608 .17427 15.228 -1.12 26 31.246 3.74 28 16.017

75 ps_rel2 19 17.173 2.8624 .65668 .16668 14.413 -0.96 3 26.316 3.19 28 11.903

102 ps_rel3 9 17.250 1.8453 .61512 .10698 14.672 -1.40 7 19.603 1.28 9 4.9309

129 ps_rel4 9 15.552 2.0834 .69445 .13396 12.433 -1.50 7 19.228 1.76 18 6.7956

156 ps_rel5 5 14.153 1.4748 .65956 .10421 12.897 -0.85 25 16.521 1.61 18 3.6242

183 ps_rel6 2 15.054 1.8020 1.2742 .11970 13.780 -0.71 5 16.328 0.71 18 2.5484

210 ps_rel7 0

237 ps_rel8 0

264 ps_rel9 20 15.291 2.9558 .66094 .19331 12.785 -0.85 6 26.398 3.76 28 13.613

22 s_rel0 18 22.366 3.9169 .92322 .17513 13.500 -2.26 2 28.261 1.51 10 14.762

49 s_rel1 19 31.444 22.554 5.1743 .71730 20.629 -0.48 4 123.97 4.10 28 103.34

76 s_rel2 19 28.192 15.316 3.5137 .54327 19.147 -0.59 5 90.339 4.06 28 71.191

103 s_rel3 9 23.933 3.5218 1.1739 .14715 19.073 -1.38 5 29.790 1.66 11 10.716

130 s_rel4 9 22.596 2.6601 .88670 .11772 19.078 -1.32 5 26.384 1.42 18 7.3056

157 s_rel5 5 21.002 1.6496 .73773 .07855 18.401 -1.58 5 22.946 1.18 18 4.5451

184 s_rel6 2 23.149 3.7655 2.6626 .16266 20.487 -0.71 5 25.812 0.71 18 5.3252

211 s_rel7 0

238 s_rel8 0

265 s_rel9 20 25.135 10.496 2.3469 .41757 19.720 -0.52 6 68.875 4.17 28 49.155

23 pz_r0 18 5.1635 2.0452 .48205 .39608 1.8497 -1.62 5 9.2853 2.02 26 7.4356

50 pz_r1 19 13.779 9.3213 2.1384 .67648 4.8429 -0.96 25 43.549 3.19 28 38.707

77 pz_r2 19 17.055 10.365 2.3778 .60773 5.3265 -1.13 9 44.553 2.65 28 39.227

104 pz_r3 9 10.176 5.1604 1.7201 .50713 6.5927 -0.69 19 22.622 2.41 11 16.029

131 pz_r4 9 15.560 6.2185 2.0728 .39964 7.8924 -1.23 18 25.340 1.57 7 17.448

158 pz_r5 5 18.340 7.1584 3.2013 .39031 11.479 -0.96 18 26.208 1.10 19 14.729

185 pz_r6 2 19.686 1.0292 .72779 .05228 18.958 -0.71 5 20.414 0.71 18 1.4556

212 pz_r7 0

239 pz_r8 0

266 pz_r9 20 21.576 4.5885 1.0260 .21267 12.054 -2.08 8 35.276 2.99 28 23.222

24 isd_r0 2 2.7414 1.7001 1.2022 .62017 1.5393 -0.71 10 3.9436 0.71 25 2.4044

51 isd_r1 19 3.3935 1.0730 .24616 .31620 1.3463 -1.91 1 5.5994 2.06 28 4.2530

78 isd_r2 19 3.8459 .82103 .18836 .21348 2.4771 -1.67 1 5.8996 2.50 28 3.4225

105 isd_r3 9 4.2309 .58326 .19442 .13786 3.2526 -1.68 5 4.7995 0.97 6 1.5469

132 isd_r4 9 3.7211 .54329 .18110 .14600 2.7823 -1.73 5 4.2655 1.00 19 1.4832

159 isd_r5 5 3.5156 .39930 .17857 .11358 3.0639 -1.13 4 3.9658 1.13 25 .90189

186 isd_r6 2 3.7518 .54929 .38841 .14641 3.3633 -0.71 5 4.1402 0.71 18 .77681

213 isd_r7 0

240 isd_r8 0

267 isd_r9 20 3.8701 1.0192 .22790 .26335 2.6377 -1.21 8 7.4486 3.51 28 4.8110

25 izd_r0 2 2.6574 1.2400 .87681 .46663 1.7806 -0.71 10 3.5342 0.71 25 1.7536

52 izd_r1 18 5.6955 1.9160 .45162 .33641 3.2422 -1.28 10 9.1092 1.78 5 5.8670

79 izd_r2 18 6.5196 1.9324 .45547 .29640 3.8515 -1.38 3 10.720 2.17 4 6.8688

106 izd_r3 9 7.2446 1.0117 .33725 .13965 5.9946 -1.24 11 8.8987 1.63 5 2.9041

133 izd_r4 9 7.2095 .99035 .33012 .13737 5.6950 -1.53 9 9.1072 1.92 5 3.4121

160 izd_r5 5 7.3131 .86338 .38611 .11806 6.4226 -1.03 19 8.5835 1.47 5 2.1609

187 izd_r6 2 7.4791 .91792 .64907 .12273 6.8300 -0.71 18 8.1282 0.71 5 1.2981

214 izd_r7 0

241 izd_r8 0

268 izd_r9 19 6.5553 1.7683 .40566 .26974 3.9113 -1.50 8 11.999 3.08 28 8.0873

26 ism_r0 2 3.8416 1.3949 .98636 .36311 2.8553 -0.71 10 4.8280 0.71 25 1.9727

53 ism_r1 16 3.3451 .93142 .23286 .27845 1.2011 -2.30 22 4.5147 1.26 25 3.3136

80 ism_r2 18 3.9205 .91366 .21535 .23304 1.7175 -2.41 3 5.4562 1.68 9 3.7387

107 ism_r3 9 4.3414 .76784 .25595 .17687 3.1345 -1.57 4 5.4828 1.49 25 2.3484

134 ism_r4 9 4.1975 .54331 .18110 .12943 3.4784 -1.32 5 4.9788 1.44 18 1.5003

161 ism_r5 5 4.1668 .54467 .24358 .13072 3.6038 -1.03 5 4.9716 1.48 18 1.3678

188 ism_r6 2 3.9118 .70489 .49844 .18020 3.4134 -0.71 5 4.4102 0.71 18 .99687

215 ism_r7 0

242 ism_r8 0

269 ism_r9 19 4.1423 1.0894 .24992 .26300 1.6828 -2.26 8 7.4123 3.00 28 5.7295

27 izm_r0 2 4.5030 2.0852 1.4744 .46306 3.0285 -0.71 10 5.9774 0.71 25 2.9488

54 izm_r1 16 6.0086 2.5302 .63255 .42110 2.3230 -1.46 11 11.848 2.31 18 9.5254

81 izm_r2 17 6.9379 2.2802 .55302 .32865 4.4626 -1.09 8 13.901 3.05 5 9.4386

108 izm_r3 9 7.5686 1.9458 .64859 .25709 5.1005 -1.27 11 12.255 2.41 5 7.1546

135 izm_r4 9 7.4888 1.3050 .43499 .17426 5.7122 -1.36 9 9.8979 1.85 5 4.1857

162 izm_r5 5 7.5774 1.3348 .59693 .17615 6.2366 -1.00 19 9.5951 1.51 5 3.3585

189 izm_r6 2 9.1053 1.4130 .99912 .15518 8.1062 -0.71 18 10.104 0.71 5 1.9982

216 izm_r7 0

243 izm_r8 0

270 izm_r9 19 6.7762 2.1116 .48444 .31162 2.2229 -2.16 8 11.468 2.22 28 9.2447

28 idz_r0 16 78.036 3.3612 .84029 .04307 72.178 -1.74 16 84.171 1.83 2 11.993

55 idz_r1 3 65.544 1.4224 .82120 .02170 64.583 -0.68 19 67.178 1.15 1 2.5950

82 idz_r2 2 57.385 7.3266 5.1807 .12768 52.204 -0.71 3 62.566 0.71 1 10.361

109 idz_r3 0

136 idz_r4 0

163 idz_r5 0

190 idz_r6 0

217 idz_r7 0

244 idz_r8 0

271 idz_r9 1 62.200 0.0000 0.0000 0.0000 62.200 1 62.200 1 0.0000

29 id_r0 3 65.682 9.1836 5.3021 .13982 59.216 -0.70 25 76.193 1.14 8 16.977

56 id_r1 15 51.040 3.7470 .96747 .07341 44.829 -1.66 16 56.947 1.58 4 12.119

83 id_r2 16 47.248 4.6451 1.1613 .09831 40.087 -1.54 22 53.826 1.42 19 13.738

110 id_r3 9 49.189 3.7591 1.2530 .07642 40.192 -2.39 11 53.226 1.07 4 13.034

137 id_r4 9 46.270 3.9831 1.3277 .08608 40.315 -1.50 7 51.445 1.30 4 11.130

164 id_r5 5 44.934 4.3640 1.9516 .09712 40.178 -1.09 25 49.654 1.08 5 9.4761

191 id_r6 2 41.012 1.7545 1.2406 .04278 39.771 -0.71 18 42.253 0.71 5 2.4812

218 id_r7 0

245 id_r8 0

272 id_r9 17 43.703 4.5509 1.1037 .10413 38.699 -1.10 7 59.202 3.41 8 20.503

30 sges_r0 19 17.900 3.5740 .81993 .19967 11.363 -1.83 2 27.194 2.60 25 15.832

57 sges_r1 19 25.122 4.9465 1.1348 .19690 17.917 -1.46 1 40.840 3.18 28 22.923

84 sges_r2 19 24.733 4.4974 1.0318 .18184 17.579 -1.59 1 37.622 2.87 28 20.043

111 sges_r3 9 25.822 2.7339 .91131 .10588 21.553 -1.56 5 28.878 1.12 18 7.3256

138 sges_r4 9 23.471 2.7650 .92166 .11780 19.345 -1.49 7 28.044 1.65 18 8.6991

165 sges_r5 5 21.835 1.9877 .88893 .09103 20.473 -0.69 5 25.306 1.75 18 4.8330

192 sges_r6 2 22.718 3.0561 2.1610 .13453 20.557 -0.71 5 24.879 0.71 18 4.3220

219 sges_r7 0

246 sges_r8 0

273 sges_r9 20 23.096 4.5919 1.0268 .19882 17.310 -1.26 1 41.258 3.96 28 23.948

31 i_zb_r0 3 4.7735 4.7559 2.7458 .99630 0.0000 -1.00 28 9.5116 1.00 25 9.5116

58 i_zb_r1 18 11.036 4.0861 .96310 .37023 3.2543 -1.90 3 19.373 2.04 18 16.119

85 i_zb_r2 18 13.072 4.4220 1.0423 .33828 3.8515 -2.09 3 23.785 2.42 5 19.934

112 i_zb_r3 9 14.813 2.8507 .95024 .19245 11.095 -1.30 11 21.154 2.22 5 10.059

139 i_zb_r4 9 14.698 2.2853 .76176 .15548 11.407 -1.44 9 19.005 1.88 5 7.5978

166 i_zb_r5 5 14.890 2.1952 .98173 .14742 12.659 -1.02 19 18.179 1.50 5 5.5194

193 i_zb_r6 2 16.584 2.3309 1.6482 .14055 14.936 -0.71 18 18.233 0.71 5 3.2964

220 i_zb_r7 0

247 i_zb_r8 0

274 i_zb_r9 19 13.331 3.7776 .86663 .28336 6.1341 -1.91 8 23.466 2.68 28 17.332

32 inf_r0 3 9.1622 9.1416 5.2779 .99775 0.0000 -1.00 28 18.283 1.00 25 18.283

59 inf_r1 19 16.666 6.5257 1.4971 .39156 1.3463 -2.35 1 28.023 1.74 18 26.676

86 inf_r2 19 19.944 6.5318 1.4985 .32751 2.4771 -2.67 1 30.575 1.63 5 28.098

113 inf_r3 9 23.386 2.3177 .77257 .09911 19.426 -1.71 11 27.979 1.98 5 8.5533

140 inf_r4 9 22.617 1.5852 .52839 .07009 19.878 -1.73 9 25.266 1.67 5 5.3881

167 inf_r5 5 22.573 1.8701 .83632 .08285 20.200 -1.27 19 24.928 1.26 5 4.7284

194 inf_r6 2 24.248 1.0767 .76135 .04440 23.487 -0.71 18 25.009 0.71 5 1.5227

221 inf_r7 0

248 inf_r8 0

275 inf_r9 20 20.470 6.7460 1.5085 .32955 2.7257 -2.63 1 38.327 2.65 28 35.601

337 dges0 19 503.28 80.411 18.448 .15978 339.24 -2.04 27 649.47 1.82 1 310.23

338 dges1 19 515.93 115.56 26.511 .22398 144.57 -3.21 28 636.55 1.04 25 491.98

339 dges2 19 519.83 104.45 23.963 .20093 171.99 -3.33 28 623.73 0.99 9 451.74

340 dges3 9 559.57 60.374 20.125 .10789 455.66 -1.72 11 650.19 1.50 9 194.53

341 dges4 9 567.77 69.301 23.100 .12206 445.29 -1.77 11 664.91 1.40 9 219.62

342 dges5 5 578.42 52.007 23.258 .08991 518.51 -1.15 18 624.82 0.89 4 106.31

343 dges6 2 591.83 71.976 50.895 .12162 540.93 -0.71 18 642.72 0.71 5 101.79

344 dges7 0

345 dges8 0

346 dges9 20 531.71 111.96 25.036 .21058 177.96 -3.16 28 682.40 1.35 9 504.44

347 STps 20 .10244 .44074 .09855 4.3023 -.72635 -1.88 16 .94844 1.92 3 1.6748

348 STpz 20 3.5175 1.5044 .33639 .42769 1.1825 -1.55 8 6.1917 1.78 17 5.0092

349 STisd 20 .24767 .29963 .06700 1.2098 -.36397 -2.04 8 .85278 2.02 1 1.2168

350 STizd 19 .41392 .47767 .10959 1.1540 -.72621 -2.39 22 1.1859 1.62 10 1.9121

351 STism 19 .31048 .36442 .08360 1.1737 -.40273 -1.96 8 1.2963 2.71 3 1.6990

352 STizm 18 .44786 .50214 .11836 1.1212 -.45150 -1.79 8 1.6454 2.38 22 2.0969

353 STidz 3 -3.8165 .56340 .32528 -.14762 -4.3950 -1.03 19 -3.2695 0.97 1 1.1255

354 STid 17 -.43881 1.0030 .24327 -2.2858 -2.6450 -2.20 22 1.4015 1.83 9 4.0465

355 STges 20 3.9876 2.7644 .61814 .69326 -4.2186 -2.97 28 7.5714 1.30 17 11.790

356 STsges 20 1.2304 .77398 .17307 .62903 -.28135 -1.95 25 2.7688 1.99 3 3.0502

357 STdz_sb 20 .36759 2.0736 .46367 5.6411 -6.9899 -3.55 28 2.4539 1.01 9 9.4438

358 STi_zb 19 1.2475 1.0851 .24893 .86980 -.71585 -1.81 8 3.2989 1.89 22 4.0147

359 STinf 20 2.0636 1.7793 .39786 .86223 -1.4825 -1.99 8 5.1589 1.74 16 6.6414

360 STps_rel 20 -.08023 .11444 .02559 -1.4265 -.28924 -1.83 16 .26502 3.02 28 .55426

361 STs_rel 20 .04039 .31892 .07131 7.8965 -.27108 -0.98 16 1.2871 3.91 28 1.5582

362 STpz_r 20 .48786 .21698 .04852 .44477 .23418 -1.17 5 .99384 2.33 28 .75966

363 STisd_r 20 .02146 .04762 .01065 2.2192 -.04506 -1.40 8 .11447 1.95 1 .15954

364 STizd_r 19 .03995 .08885 .02038 2.2239 -.18255 -2.50 22 .20404 1.85 28 .38659

365 STism_r 19 .03412 .06733 .01545 1.9731 -.05901 -1.38 8 .19337 2.37 28 .25239

366 STizm_r 18 .04332 .08869 .02090 2.0471 -.06623 -1.24 9 .23450 2.16 28 .30072

367 STidz_r 3 -1.1389 .46445 .26815 -.40782 -1.5502 -0.89 3 -.63518 1.08 1 .91502

368 STid_r 17 -.36461 .21140 .05127 -.57982 -.86967 -2.39 22 -.03916 1.54 16 .83051

369 STsges_r 20 .09107 .19685 .04402 2.1616 -.23723 -1.67 6 .77115 3.45 28 1.0084

370 STi_zb_r 19 .11567 .20403 .04681 1.7638 -.08151 -0.97 8 .79413 3.33 28 .87563

371 STinf_r 20 .17689 .31973 .07149 1.8075 -.18558 -1.13 8 1.3003 3.51 28 1.4858

372 STdges 20 .47005 2.1068 .47110 4.4822 -6.9082 -3.50 28 2.5680 1.00 9 9.4762

373 STpsr2 20 .02583 .17782 .03976 6.8854 -.17178 -1.11 16 .69706 3.77 28 .86884

374 STidr2 17 -.16024 .17480 .04239 -1.0909 -.45418 -1.68 10 .16972 1.89 16 .62390

375 STisdr2 20 .04122 .05515 .01233 1.3379 -.07829 -2.17 8 .15309 2.03 1 .23138

376 STismr2 19 .06007 .08277 .01899 1.3778 -.08885 -1.80 8 .25253 2.33 28 .34138

377 STsgesr2 20 .26166 .32271 .07216 1.2333 -.07710 -1.05 6 1.4956 3.82 28 1.5727

378 STinfr2 20 .43071 .51130 .11433 1.1871 -.31938 -1.47 8 2.0505 3.17 28 2.3699

NUMBER OF CASES READ. . . . . . . . . . . . . . 28

CASES WITH USE SET TO ZERO . . . . . . . . . 8

REMAINING NUMBER OF CASES . . . . . . . . 20

STps VAR. 347 VS. MEAN= 0.0000

*******************************************

TEST STATISTICS P-VALUE DF

------------------- --------------------------------

MEAN 0.1024 1-SAMPLE T 1.04 0.3116 19

H STD DEV 0.4407

HH S.E.M. 0.0986

H H HH H SAMPLE SIZE 20

H HHH HHHHH HH H MAXIMUM 0.9484

M--------------------M MINIMUM -0.7263

I AN H= 1 CASES A Z MAX 1.92

N (N= 20) X Z MIN -1.88

CASE (MAX) 3

CASE (MIN) 16

STpz VAR. 348 VS. MEAN= 0.0000

*******************************************

TEST STATISTICS P-VALUE DF

------------------- --------------------------------

MEAN 3.5175 1-SAMPLE T 10.46 0.0000 19

STD DEV 1.5044

H H S.E.M. 0.3364

H HH H SAMPLE SIZE 20

HHHHHHHHH HHH HH MAXIMUM 6.1917

M--------------------M MINIMUM 1.1825

I AN H= 1 CASES A Z MAX 1.78

N (N= 20) X Z MIN -1.55

CASE (MAX) 17

CASE (MIN) 8

STid VAR. 354 VS. MEAN= 0.0000

*******************************************

TEST STATISTICS P-VALUE DF

------------------- --------------------------------

MEAN -0.4388 1-SAMPLE T -1.80 0.0901 16

STD DEV 1.0030

H S.E.M. 0.2433

H H H SAMPLE SIZE 17

H HHHHHHHHHHH H MAXIMUM 1.4015

M--------------------M MINIMUM -2.6450

I AN H= 1 CASES A Z MAX 1.83

N (N= 17) X Z MIN -2.20

CASE (MAX) 9

CASE (MIN) 22

STges VAR. 355 VS. MEAN= 0.0000

*******************************************

TEST STATISTICS P-VALUE DF

------------------- --------------------------------

MEAN 3.9876 1-SAMPLE T 6.45 0.0000 19

H

H STD DEV 2.7644

H H S.E.M. 0.6181

HHH HHHH SAMPLE SIZE 20

H HHH HHHHH MAXIMUM 7.5714

M--------------------M MINIMUM -4.2186

I AN H= 1 CASES A Z MAX 1.30

N (N= 20) X Z MIN -2.97

CASE (MAX) 17

CASE (MIN) 28

STsges VAR. 356 VS. MEAN= 0.0000

*******************************************

TEST STATISTICS P-VALUE DF

------------------- --------------------------------

MEAN 1.2304 1-SAMPLE T 7.11 0.0000 19

STD DEV 0.7740

H HH S.E.M. 0.1731

H HH H SAMPLE SIZE 20

HH HHHHHHHH HHH MAXIMUM 2.7688

M--------------------M MINIMUM -0.2813

I AN H= 1 CASES A Z MAX 1.99

N (N= 20) X Z MIN -1.95

CASE (MAX) 3

CASE (MIN) 25

STinf VAR. 359 VS. MEAN= 0.0000

*******************************************

TEST STATISTICS P-VALUE DF

------------------- --------------------------------

H MEAN 2.0636 1-SAMPLE T 5.19 0.0001 19

H

H STD DEV 1.7793

H H S.E.M. 0.3979

H H H H H SAMPLE SIZE 20

H H H HHH H HHH MAXIMUM 5.1589

M--------------------M MINIMUM -1.4825

I AN H= 1 CASES A Z MAX 1.74

N (N= 20) X Z MIN -1.99

CASE (MAX) 16

CASE (MIN) 8

STpsr2 VAR. 373 VS. MEAN= 0.0000

*******************************************

TEST STATISTICS P-VALUE DF

------------------- --------------------------------

MEAN 0.0258 1-SAMPLE T 0.65 0.5238 19

H STD DEV 0.1778

H S.E.M. 0.0398

HH SAMPLE SIZE 20

HHHHHHH H MAXIMUM 0.6971

M--------------------M MINIMUM -0.1718

I AN H= 2 CASES A Z MAX 3.77

N (N= 20) X Z MIN -1.11

CASE (MAX) 28

CASE (MIN) 16

STidr2 VAR. 374 VS. MEAN= 0.0000

*******************************************

TEST STATISTICS P-VALUE DF

------------------- --------------------------------

MEAN -0.1602 1-SAMPLE T -3.78 0.0016 16

H STD DEV 0.1748

H S.E.M. 0.0424

H H HH H SAMPLE SIZE 17

HH H H HHHH H H MAXIMUM 0.1697

M--------------------M MINIMUM -0.4542

I AN H= 1 CASES A Z MAX 1.89

N (N= 17) X Z MIN -1.68

CASE (MAX) 16

CASE (MIN) 10

STsgesr2 VAR. 377 VS. MEAN= 0.0000

*******************************************

TEST STATISTICS P-VALUE DF

------------------- --------------------------------

MEAN 0.2617 1-SAMPLE T 3.63 0.0018 19

H STD DEV 0.3227

H S.E.M. 0.0722

HH SAMPLE SIZE 20

HHHHHHH H MAXIMUM 1.4956

M--------------------M MINIMUM -0.0771

I AN H= 2 CASES A Z MAX 3.82

N (N= 20) X Z MIN -1.05

CASE (MAX) 28

CASE (MIN) 6

STinfr2 VAR. 378 VS. MEAN= 0.0000

*******************************************

TEST STATISTICS P-VALUE DF

------------------- --------------------------------

MEAN 0.4307 1-SAMPLE T 3.77 0.0013 19

H

H H STD DEV 0.5113

HH H S.E.M. 0.1143

HH H H H SAMPLE SIZE 20

H HHHHHH H H MAXIMUM 2.0505

M--------------------M MINIMUM -0.3194

I AN H= 1 CASES A Z MAX 3.17

N (N= 20) X Z MIN -1.47

CASE (MAX) 28

CASE (MIN) 8

NUMBER OF INTEGER WORDS USED IN PRECEDING SUBPROBLEM 24021

/ FINISH

PROGRAM TERMINATED

LOWER CHEEK TEETH

MDP3D - T-TESTS

Copyright 1977, 1979, 1981, 1982, 1983, 1985, 1987, 1988, 1990, 1993

by BMDP Statistical Software, Inc.

Statistical Solutions Ltd. | Statistical Solutions

Unit 1A, South Ring Business Park | Stonehill Corporate Center, Suite 104

Kinsale Road, Cork, Ireland | 999 Broadway, Saugus, MA 01906, USA

Phone: + 353 21 4319629 | Phone: 781.231.7680

Fax: + 353 21 4319630 | Fax: 781.231.7684

e-mail: sales@statsol.ie | e-mail: info@statsolusa.com

Website: http://www.statsol.ie | Website: http://www.statsolusa.com

Release: 8.1 (Windows 9x, 2000, Me, Xp) Date: 04/29/16 at 10:36:10

Manual: BMDP Manual Volumes 1, 2, and 3.

Digest: BMDP User's Digest.

IBM PC: BMDP PC Supplement -- Installation and Special Features.

PROGRAM INSTRUCTIONS

/prob title = 'Herr Lauritz Englisch: B3D2.inp *** Quantitative Studien

zum Schmelzgehalt in Pferdebackenzaehnen.

- 1 Fall = 1 Zahn = 10 Lokalisationen = 10 Zeilen

- Hier: Unterkiefer, Zahn 7 - 10

- Umrechnung der relativen Werte in Prozentwerte

- Berechnung der Flächenanteile ohne aeusseren Zement

- mit Berechnung der Steigung "ST" ueber die Schnittebenen

- Einstichproben-t-Test gegen H0: ST = 0

***'.

/inp var = 87.

file = b.

format = '14f10,8(/50x,9f10), /20x,1f10'. ## Mit Abstandsangabe für die Ebene 9

/var names = zahnid,zp,qu,znr,za,

for lo = 0 to 8.% ## Lokalisation

for va = lok,statu,ps,pz,id,ges,s_rel,z_rel,d_rel.%

|va||lo|,%%

abst99. ## Mit Abstandsangabe für die Ebene 99

use = zp,qu,znr,za,

for va = lok,ps,pz,id,ges,s_rel,z_rel,d_rel,dges.%

for lo = 0 to 8.% ## Lokalisation

|va||lo|,%%

for va = ps,id.%

for lo = 0 to 8.%

|va|_r2|lo|,%%

for va = ps,pz,id,ges,s_rel,z_rel,d_rel,dges.%

ST|va|,%

for va = ps,id.%

ST|va|r2,%

.

/trans use = ((znr ge 7) AND (znr le 10)).

lok8 = abst99. ## Abstandsangabe für die Ebene 99

# Umrechnung der relativen Werte in Prozentwerte

for va = s_rel,z_rel,d_rel.%

for lo = 0 to 8.%

|va||lo| = |va||lo| * 100.%%

# Berechnung der Flächenanteile ohne aeusseren Zement

for va = ps,id.%

for lo = 0 to 8.%

|va|_r2|lo| = |va||lo|/(ges|lo|-pz|lo|) * 100.%%

# Berechnung der Gesamtflaeche abzüglich peripherem Zement

for lo = 0 to 8.%

dges|lo| = ges|lo|-pz|lo|.%

# Berechnung der Steigung "ST" ueber die Schnittebenen

for va = ps,pz,id,ges,s_rel,z_rel,d_rel,dges.%

ST|va| = b(for lo = 0 to 8.% lok|lo|,|va||lo|,%).

%

for va = ps,id.%

ST|va|r2 = b(for lo = 0 to 8.% lok|lo|,|va|_r2|lo|,%).

%

/onegroup

var = for va = ps,pz,id,ges,s_rel,z_rel,d_rel,dges.%

ST|va|,%

for va = ps,id.%

ST|va|r2,%.

/print level = min.

case = 0.

/end

--- PROGRAM INSTRUCTIONS AFTER "FOR %" EXPANSION ---

/prob title = 'Herr Lauritz Englisch: B3D2.inp *** Quantitative Studien zum

Schmelzgehalt in Pferdebackenzaehnen.

- 1 Fall = 1 Zahn = 10 Lokalisationen =

10 Zeilen - Hier: Unterkiefer,

Zahn 7 - 10 - Umrechnung der relativen Werte in Prozentwerte -

Berechnung der Flächenanteile ohne aeusseren Zement - mit Berechnung

der Steigung "ST" ueber die Schnittebenen - Einstichproben-t-Test

gegen H0: ST = 0 ***'.

/inp var = 87. file = b. format = '14f10,8(/50x,9f10),

/20x,1f10'.

/var names = zahnid,zp,qu,znr,za, lok0, statu0, ps0, pz0, id0, ges0, s_rel0,

z_rel0, d_rel0, lok1, statu1, ps1, pz1, id1, ges1, s_rel1, z_rel1,

d_rel1, lok2, statu2, ps2, pz2, id2, ges2, s_rel2, z_rel2, d_rel2,

lok3, statu3, ps3, pz3, id3, ges3, s_rel3, z_rel3, d_rel3, lok4,

statu4, ps4, pz4, id4, ges4, s_rel4, z_rel4, d_rel4, lok5, statu5,

ps5, pz5, id5, ges5, s_rel5, z_rel5, d_rel5, lok6, statu6, ps6,

pz6, id6, ges6, s_rel6, z_rel6, d_rel6, lok7, statu7, ps7, pz7,

id7, ges7, s_rel7, z_rel7, d_rel7, lok8, statu8, ps8, pz8, id8,

ges8, s_rel8, z_rel8, d_rel8, abst99.

use = zp,qu,znr,za, lok0, lok1, lok2, lok3, lok4, lok5, lok6,

lok7, lok8, ps0, ps1, ps2, ps3, ps4, ps5, ps6, ps7, ps8,

pz0, pz1, pz2, pz3, pz4, pz5, pz6, pz7, pz8, id0, id1,

id2, id3, id4, id5, id6, id7, id8, ges0, ges1, ges2,

ges3, ges4, ges5, ges6, ges7, ges8, s_rel0, s_rel1,

s_rel2, s_rel3, s_rel4, s_rel5, s_rel6, s_rel7, s_rel8,

z_rel0, z_rel1, z_rel2, z_rel3, z_rel4, z_rel5, z_rel6,

z_rel7, z_rel8, d_rel0, d_rel1, d_rel2, d_rel3, d_rel4,

d_rel5, d_rel6, d_rel7, d_rel8, dges0, dges1, dges2, dges3,

dges4, dges5, dges6, dges7, dges8, ps_r20, ps_r21, ps_r22,

ps_r23, ps_r24, ps_r25, ps_r26, ps_r27, ps_r28, id_r20, id_r21,

id_r22, id_r23, id_r24, id_r25, id_r26, id_r27, id_r28, STps, STpz,

STid, STges, STs_rel, STz_rel, STd_rel, STdges, STpsr2, STidr2 .

/trans use = ((znr ge 7) AND (znr le 10)). lok8 = abst99.

s_rel0 = s_rel0 * 100. s_rel1 = s_rel1 * 100. s_rel2 = s_rel2 * 100.

s_rel3 = s_rel3 * 100. s_rel4 = s_rel4 * 100. s_rel5 = s_rel5 * 100.

s_rel6 = s_rel6 * 100. s_rel7 = s_rel7 * 100. s_rel8 = s_rel8 * 100.

z_rel0 = z_rel0 * 100. z_rel1 = z_rel1 * 100. z_rel2 = z_rel2 * 100.

z_rel3 = z_rel3 * 100. z_rel4 = z_rel4 * 100. z_rel5 = z_rel5 * 100.

z_rel6 = z_rel6 * 100. z_rel7 = z_rel7 * 100. z_rel8 = z_rel8 * 100.

d_rel0 = d_rel0 * 100. d_rel1 = d_rel1 * 100. d_rel2 = d_rel2 * 100.

d_rel3 = d_rel3 * 100. d_rel4 = d_rel4 * 100. d_rel5 = d_rel5 * 100.

d_rel6 = d_rel6 * 100. d_rel7 = d_rel7 * 100. d_rel8 = d_rel8 * 100.

ps_r20 = ps0/(ges0-pz0) * 100. ps_r21 = ps1/(ges1-pz1) * 100.

ps_r22 = ps2/(ges2-pz2) * 100. ps_r23 = ps3/(ges3-pz3) * 100.

ps_r24 = ps4/(ges4-pz4) * 100. ps_r25 = ps5/(ges5-pz5) * 100.

ps_r26 = ps6/(ges6-pz6) * 100. ps_r27 = ps7/(ges7-pz7) * 100.

ps_r28 = ps8/(ges8-pz8) * 100. id_r20 = id0/(ges0-pz0) * 100.

id_r21 = id1/(ges1-pz1) * 100. id_r22 = id2/(ges2-pz2) * 100.

id_r23 = id3/(ges3-pz3) * 100. id_r24 = id4/(ges4-pz4) * 100.

id_r25 = id5/(ges5-pz5) * 100. id_r26 = id6/(ges6-pz6) * 100.

id_r27 = id7/(ges7-pz7) * 100. id_r28 = id8/(ges8-pz8) * 100.

dges0 = ges0-pz0. dges1 = ges1-pz1. dges2 = ges2-pz2. dges3 = ges3-pz3.

dges4 = ges4-pz4. dges5 = ges5-pz5. dges6 = ges6-pz6. dges7 = ges7-pz7.

dges8 = ges8-pz8. STps =

b( lok0,ps0, lok1,ps1, lok2,ps2, lok3,ps3, lok4,ps4, lok5,ps5, lok6,

ps6, lok7,ps7, lok8,ps8).

STpz = b( lok0,pz0, lok1,pz1, lok2,pz2, lok3,pz3, lok4,pz4, lok5,pz5,

lok6,pz6, lok7,pz7, lok8,pz8).

STid = b( lok0,id0, lok1,id1, lok2,id2, lok3,id3, lok4,id4, lok5,id5,

lok6,id6, lok7,id7, lok8,id8).

STges = b( lok0,ges0, lok1,ges1, lok2,ges2, lok3,ges3, lok4,ges4, lok5,

ges5, lok6,ges6, lok7,ges7, lok8,ges8).

STs_rel = b( lok0,s_rel0, lok1,s_rel1, lok2,s_rel2, lok3,s_rel3, lok4,

s_rel4, lok5,s_rel5, lok6,s_rel6, lok7,s_rel7, lok8,s_rel8).

STz_rel = b( lok0,z_rel0, lok1,z_rel1, lok2,z_rel2, lok3,z_rel3, lok4,

z_rel4, lok5,z_rel5, lok6,z_rel6, lok7,z_rel7, lok8,z_rel8).

STd_rel = b( lok0,d_rel0, lok1,d_rel1, lok2,d_rel2, lok3,d_rel3, lok4,

d_rel4, lok5,d_rel5, lok6,d_rel6, lok7,d_rel7, lok8,d_rel8).

STdges = b( lok0,dges0, lok1,dges1, lok2,dges2, lok3,dges3, lok4,dges4,

lok5,dges5, lok6,dges6, lok7,dges7, lok8,dges8).

STpsr2 = b( lok0,ps_r20, lok1,ps_r21, lok2,ps_r22, lok3,ps_r23, lok4,

ps_r24, lok5,ps_r25, lok6,ps_r26, lok7,ps_r27, lok8,ps_r28).

STidr2 = b( lok0,id_r20, lok1,id_r21, lok2,id_r22, lok3,id_r23, lok4,

id_r24, lok5,id_r25, lok6,id_r26, lok7,id_r27, lok8,id_r28).

/onegroup var = STps, STpz, STid, STges, STs_rel, STz_rel, STd_rel, STdges,

STpsr2, STidr2.

/print level = min. case = 0.

/end/

NUMBER OF CASES READ. . . . . . . . . . . . . . 26

CASES WITH USE SET TO ZERO . . . . . . . . . 10

REMAINING NUMBER OF CASES . . . . . . . . 16

DESCRIPTIVE STATISTICS OF DATA

----------- ---------- -- ----

VARIABLE TOTAL STANDARD ST.ERR COEFF S M A L L E S T L A R G E S T

NO. NAME FREQ. MEAN DEV. OF MEAN OF VAR VALUE Z-SCR CASE VALUE Z-SCR CASE RANGE

2 zp 16 376.75 47.885 11.971 .12710 307.00 -1.46 22 410.00 0.69 16 103.00

3 qu 16 3.6875 .47871 .11968 .12982 3.0000 -1.44 13 4.0000 0.65 1 1.0000

4 znr 16 8.0000 1.1547 .28868 .14434 7.0000 -0.87 1 10.000 1.73 16 3.0000

5 za 16 9.9375 5.0986 1.2747 .51307 1.5000 -1.65 20 16.000 1.19 6 14.500

6 lok0 16 0.0000 0.0000 0.0000 0.0000 1 0.0000 1 0.0000

15 lok1 16 10.000 0.0000 0.0000 0.0000 10.000 1 10.000 1 0.0000

24 lok2 16 20.000 0.0000 0.0000 0.0000 20.000 1 20.000 1 0.0000

33 lok3 16 30.000 0.0000 0.0000 0.0000 30.000 1 30.000 1 0.0000

42 lok4 16 40.000 0.0000 0.0000 0.0000 40.000 1 40.000 1 0.0000

51 lok5 16 50.000 0.0000 0.0000 0.0000 50.000 1 50.000 1 0.0000

60 lok6 16 60.000 0.0000 0.0000 0.0000 60.000 1 60.000 1 0.0000

69 lok7 16 70.000 0.0000 0.0000 0.0000 70.000 1 70.000 1 0.0000

78 lok8 16 41.292 19.043 4.7607 .46118 21.570 -1.04 5 75.770 1.81 20 54.200

8 ps0 16 89.754 19.127 4.7819 .21311 62.530 -1.42 24 134.11 2.32 4 71.580

17 ps1 16 120.94 20.451 5.1127 .16910 78.390 -2.08 24 161.60 1.99 4 83.210

26 ps2 16 123.72 22.112 5.5280 .17872 82.190 -1.88 24 156.03 1.46 4 73.840

35 ps3 9 127.59 16.927 5.6424 .13267 104.87 -1.34 15 154.31 1.58 22 49.440

44 ps4 6 128.68 16.916 6.9061 .13146 104.75 -1.41 15 146.52 1.05 22 41.770

53 ps5 5 124.15 12.314 5.5069 .09918 105.90 -1.48 15 139.93 1.28 22 34.030

62 ps6 4 128.48 4.2764 2.1382 .03329 124.47 -0.94 16 133.20 1.10 22 8.7300

71 ps7 1 132.77 0.0000 0.0000 0.0000 132.77 20 132.77 20 0.0000

80 ps8 16 117.98 18.737 4.6842 .15881 81.150 -1.97 25 144.73 1.43 16 63.580

9 pz0 16 50.933 35.626 8.9066 .69947 16.270 -0.97 20 123.60 2.04 4 107.33

18 pz1 16 109.45 64.351 16.088 .58795 32.440 -1.20 16 208.98 1.55 5 176.54

27 pz2 16 159.88 83.986 20.996 .52530 40.650 -1.42 16 292.54 1.58 13 251.89

36 pz3 9 142.12 84.246 28.082 .59277 51.940 -1.07 16 279.64 1.63 4 227.70

45 pz4 5 96.750 35.789 16.006 .36992 55.760 -1.15 16 137.65 1.14 1 81.890

54 pz5 5 162.12 89.864 40.188 .55431 65.850 -1.07 16 267.20 1.17 22 201.35

63 pz6 4 208.12 27.576 13.788 .13250 187.00 -0.77 16 247.63 1.43 15 60.630

72 pz7 1 253.18 0.0000 0.0000 0.0000 253.18 20 253.18 20 0.0000

81 pz8 16 165.91 42.773 10.693 .25781 92.850 -1.71 24 257.61 2.14 13 164.76

10 id0 16 192.71 32.884 8.2211 .17064 108.33 -2.57 24 232.01 1.20 5 123.68

19 id1 16 169.97 30.189 7.5473 .17761 104.42 -2.17 24 229.04 1.96 7 124.62

28 id2 16 170.20 28.516 7.1290 .16755 102.30 -2.38 24 218.55 1.70 4 116.25

37 id3 9 193.51 24.499 8.1665 .12661 156.56 -1.51 2 222.95 1.20 4 66.390

46 id4 6 183.51 27.205 11.106 .14824 152.21 -1.15 16 216.25 1.20 22 64.040

55 id5 5 189.13 31.456 14.067 .16631 150.48 -1.23 16 219.37 0.96 22 68.890

64 id6 4 178.41 32.670 16.335 .18312 147.75 -0.94 15 207.04 0.88 20 59.290

73 id7 1 193.95 0.0000 0.0000 0.0000 193.95 20 193.95 20 0.0000

82 id8 16 172.95 32.968 8.2419 .19062 102.53 -2.14 24 225.12 1.58 7 122.59

11 ges0 16 333.40 61.417 15.354 .18422 209.51 -2.02 24 461.13 2.08 4 251.62

20 ges1 16 400.36 82.318 20.579 .20561 271.44 -1.57 24 519.47 1.45 5 248.03

29 ges2 16 453.80 97.804 24.451 .21552 311.87 -1.45 16 605.64 1.55 4 293.77

38 ges3 9 463.21 105.96 35.321 .22876 332.43 -1.23 16 650.18 1.76 4 317.75

47 ges4 5 417.42 66.830 29.887 .16010 345.03 -1.08 16 493.87 1.14 22 148.84

56 ges5 5 475.41 123.61 55.278 .26000 337.48 -1.12 16 626.51 1.22 22 289.03

65 ges6 4 515.00 35.492 17.746 .06892 464.06 -1.44 16 545.60 0.86 22 81.540

74 ges7 1 579.90 0.0000 0.0000 0.0000 579.90 20 579.90 20 0.0000

83 ges8 16 456.83 82.501 20.625 .18059 281.42 -2.13 24 569.58 1.37 4 288.16

12 s_rel0 16 27.038 3.4510 .86274 .12764 21.600 -1.58 5 35.500 2.45 22 13.900

21 s_rel1 16 30.725 4.6540 1.1635 .15147 23.500 -1.55 7 38.700 1.71 16 15.200

30 s_rel2 16 27.969 5.8900 1.4725 .21059 21.900 -1.03 25 37.700 1.65 15 15.800

39 s_rel3 9 28.344 5.0683 1.6894 .17881 22.200 -1.21 7 34.800 1.27 16 12.600

48 s_rel4 5 32.000 4.9031 2.1927 .15322 27.300 -0.96 1 39.700 1.57 16 12.400

57 s_rel5 5 27.160 5.6487 2.5262 .20798 22.000 -0.91 1 35.900 1.55 16 13.900

66 s_rel6 4 25.000 1.2247 .61237 .04899 24.100 -0.73 15 26.800 1.47 16 2.7000

75 s_rel7 1 22.900 0.0000 0.0000 0.0000 22.900 20 22.900 20 0.0000

84 s_rel8 16 26.131 3.3420 .83550 .12789 21.100 -1.51 13 35.000 2.65 16 13.900

13 z_rel0 16 14.681 8.4979 2.1245 .57883 4.9000 -1.15 20 27.000 1.45 23 22.100

22 z_rel1 16 25.912 11.793 2.9481 .45509 10.600 -1.30 16 42.300 1.39 23 31.700

31 z_rel2 16 33.500 13.764 3.4410 .41086 12.600 -1.52 15 49.900 1.19 13 37.300

40 z_rel3 9 28.722 11.480 3.8266 .39969 15.600 -1.14 16 43.000 1.24 4 27.400

49 z_rel4 5 22.640 5.3984 2.4142 .23845 16.200 -1.19 16 29.200 1.22 1 13.000

58 z_rel5 5 32.080 10.560 4.7224 .32917 19.500 -1.19 16 43.000 1.03 1 23.500

67 z_rel6 4 40.475 5.0408 2.5204 .12454 36.200 -0.85 20 47.600 1.41 15 11.400

76 z_rel7 1 43.700 0.0000 0.0000 0.0000 43.700 20 43.700 20 0.0000

85 z_rel8 16 35.956 4.2417 1.0604 .11797 26.600 -2.21 23 46.800 2.56 13 20.200

14 d_rel0 16 58.275 7.5841 1.8960 .13014 44.100 -1.87 4 70.300 1.59 2 26.200

23 d_rel1 16 43.369 8.0354 2.0088 .18528 32.100 -1.40 14 53.600 1.27 20 21.500

32 d_rel2 16 38.531 8.0440 2.0110 .20877 27.200 -1.41 13 50.900 1.54 16 23.700

41 d_rel3 9 42.900 6.8218 2.2739 .15902 34.300 -1.26 4 50.700 1.14 15 16.400

50 d_rel4 5 45.340 2.1686 .96985 .04783 43.400 -0.89 1 47.700 1.09 15 4.3000

59 d_rel5 5 40.740 5.5891 2.4995 .13719 35.000 -1.03 1 47.300 1.17 20 12.300

68 d_rel6 4 34.550 4.8925 2.4463 .14161 28.400 -1.26 15 39.100 0.93 20 10.700

77 d_rel7 1 33.400 0.0000 0.0000 0.0000 33.400 20 33.400 20 0.0000

86 d_rel8 16 37.925 3.3670 .84175 .08878 32.100 -1.73 13 45.800 2.34 23 13.700

106 dges0 16 282.46 45.841 11.460 .16229 170.86 -2.43 24 337.53 1.20 4 166.67

107 dges1 16 290.91 45.745 11.436 .15725 182.80 -2.36 24 361.64 1.55 4 178.84

108 dges2 16 293.92 49.666 12.416 .16898 184.49 -2.20 24 374.58 1.62 4 190.09

109 dges3 9 321.09 39.081 13.027 .12171 267.09 -1.38 2 370.54 1.27 4 103.45

110 dges4 5 320.67 37.651 16.838 .11741 273.83 -1.24 15 362.77 1.12 22 88.940

111 dges5 5 313.30 41.784 18.686 .13337 266.62 -1.12 15 359.31 1.10 22 92.690

112 dges6 4 306.89 36.813 18.407 .11996 273.02 -0.92 15 339.45 0.88 22 66.430

113 dges7 1 326.72 0.0000 0.0000 0.0000 326.72 20 326.72 20 0.0000

114 dges8 16 290.93 46.895 11.724 .16119 188.57 -2.18 24 360.36 1.48 7 171.79

88 ps_r20 16 31.819 3.8969 .97422 .12247 25.378 -1.65 2 39.733 2.03 4 14.355

89 ps_r21 16 41.663 3.3050 .82625 .07933 32.431 -2.79 7 46.323 1.41 14 13.893

90 ps_r22 16 42.066 1.7951 .44878 .04267 39.175 -1.61 23 45.722 2.04 13 6.5471

91 ps_r23 9 39.743 2.0723 .69077 .05214 36.751 -1.44 1 42.341 1.25 22 5.5891

92 ps_r24 5 41.221 3.6860 1.6484 .08942 38.254 -0.81 15 47.381 1.67 16 9.1277

93 ps_r25 5 39.833 2.8098 1.2566 .07054 37.243 -0.92 20 44.597 1.70 16 7.3542

94 ps_r26 4 42.199 3.7271 1.8636 .08832 38.747 -0.93 20 45.883 0.99 15 7.1357

95 ps_r27 1 40.637 0.0000 0.0000 0.0000 40.637 20 40.637 20 0.0000

96 ps_r28 16 40.740 3.9343 .98358 .09657 37.127 -0.92 1 51.268 2.68 16 14.141

97 id_r20 16 68.181 3.8975 .97438 .05716 60.267 -2.03 4 74.626 1.65 2 14.358

98 id_r21 16 58.337 3.3050 .82625 .05665 53.677 -1.41 14 67.569 2.79 7 13.893

99 id_r22 16 57.934 1.7950 .44875 .03098 54.274 -2.04 13 60.825 1.61 23 6.5505

100 id_r23 9 60.257 2.0728 .69094 .03440 57.659 -1.25 22 63.249 1.44 1 5.5891

101 id_r24 5 58.778 3.6853 1.6481 .06270 52.619 -1.67 16 61.743 0.80 15 9.1240

102 id_r25 5 60.164 2.8101 1.2567 .04671 55.399 -1.70 16 62.754 0.92 20 7.3550

103 id_r26 4 57.802 3.7279 1.8640 .06450 54.117 -0.99 15 61.253 0.93 20 7.1357

104 id_r27 1 59.363 0.0000 0.0000 0.0000 59.363 20 59.363 20 0.0000

105 id_r28 16 59.261 3.9346 .98366 .06640 48.728 -2.68 16 62.870 0.92 1 14.142

115 STps 16 .68289 .52635 .13159 .77077 -.11643 -1.52 22 1.7245 1.98 5 1.8409

116 STpz 16 3.7048 1.4794 .36985 .39932 1.4930 -1.50 23 7.5728 2.61 13 6.0798

117 STid 16 -.38397 .60354 .15089 -1.5718 -2.1609 -2.94 5 .45713 1.39 4 2.6180

118 STges 16 3.9974 1.7050 .42625 .42652 2.1687 -1.07 16 8.2274 2.48 13 6.0587

119 STs_rel 16 -.07953 .11042 .02760 -1.3884 -.23804 -1.44 22 .24584 2.95 5 .48388

120 STz_rel 16 .61927 .21218 .05305 .34264 .17742 -2.08 23 1.0073 1.83 13 .82991

121 STd_rel 16 -.53972 .21815 .05454 -.40419 -.87326 -1.53 13 -.16331 1.73 23 .70996

122 STdges 16 .29265 .50220 .12555 1.7160 -.43653 -1.45 5 1.3783 2.16 7 1.8148

123 STpsr2 16 .20630 .16401 .04100 .79501 -.03888 -1.49 4 .59045 2.34 5 .62933

124 STidr2 16 -.20628 .16400 .04100 -.79505 -.59042 -2.34 5 .03885 1.49 4 .62927

NUMBER OF CASES READ. . . . . . . . . . . . . . 26

CASES WITH USE SET TO ZERO . . . . . . . . . 10

REMAINING NUMBER OF CASES . . . . . . . . 16

STps VAR. 115 VS. MEAN= 0.0000

*******************************************

TEST STATISTICS P-VALUE DF

------------------- --------------------------------

MEAN 0.6829 1-SAMPLE T 5.19 0.0001 15

H STD DEV 0.5264

H S.E.M. 0.1316

H H H H SAMPLE SIZE 16

H HHH HHH H HH MAXIMUM 1.7245

M--------------------M MINIMUM -0.1164

I AN H= 1 CASES A Z MAX 1.98

N (N= 16) X Z MIN -1.52

CASE (MAX) 5

CASE (MIN) 22

STpz VAR. 116 VS. MEAN= 0.0000

*******************************************

TEST STATISTICS P-VALUE DF

------------------- --------------------------------

MEAN 3.7048 1-SAMPLE T 10.02 0.0000 15

H STD DEV 1.4794

HH S.E.M. 0.3698

HH H SAMPLE SIZE 16

HH HHHHHHH H MAXIMUM 7.5728

M--------------------M MINIMUM 1.4930

I AN H= 1 CASES A Z MAX 2.61

N (N= 16) X Z MIN -1.50

CASE (MAX) 13

CASE (MIN) 23

STid VAR. 117 VS. MEAN= 0.0000

*******************************************

TEST STATISTICS P-VALUE DF

------------------- --------------------------------

MEAN -0.3840 1-SAMPLE T -2.54 0.0224 15

STD DEV 0.6035

H H S.E.M. 0.1509

HHHH H SAMPLE SIZE 16

H HHHHHHH H MAXIMUM 0.4571

M--------------------M MINIMUM -2.1609

I AN H= 1 CASES A Z MAX 1.39

N (N= 16) X Z MIN -2.94

CASE (MAX) 4

CASE (MIN) 5

STges VAR. 118 VS. MEAN= 0.0000

*******************************************

TEST STATISTICS P-VALUE DF

------------------- --------------------------------

MEAN 3.9974 1-SAMPLE T 9.38 0.0000 15

STD DEV 1.7050

H S.E.M. 0.4263

HH HH H SAMPLE SIZE 16

HHHHH HH HH H MAXIMUM 8.2274

M--------------------M MINIMUM 2.1687

I AN H= 1 CASES A Z MAX 2.48

N (N= 16) X Z MIN -1.07

CASE (MAX) 13

CASE (MIN) 16

STdges VAR. 122 VS. MEAN= 0.0000

*******************************************

TEST STATISTICS P-VALUE DF

------------------- --------------------------------

MEAN 0.2927 1-SAMPLE T 2.33 0.0341 15

STD DEV 0.5022

S.E.M. 0.1256

H HHH H SAMPLE SIZE 16

HH HHHH HHH H H MAXIMUM 1.3783

M--------------------M MINIMUM -0.4365

I AN H= 1 CASES A Z MAX 2.16

N (N= 16) X Z MIN -1.45

CASE (MAX) 7

CASE (MIN) 5

STpsr2 VAR. 123 VS. MEAN= 0.0000

*******************************************

TEST STATISTICS P-VALUE DF

------------------- --------------------------------

MEAN 0.2063 1-SAMPLE T 5.03 0.0001 15

STD DEV 0.1640

S.E.M. 0.0410

H H H H H SAMPLE SIZE 16

H HH HHHHHHH H MAXIMUM 0.5905

M--------------------M MINIMUM -0.0389

I AN H= 1 CASES A Z MAX 2.34

N (N= 16) X Z MIN -1.49

CASE (MAX) 5

CASE (MIN) 4

STidr2 VAR. 124 VS. MEAN= 0.0000

*******************************************

TEST STATISTICS P-VALUE DF

------------------- --------------------------------

MEAN -0.2063 1-SAMPLE T -5.03 0.0001 15

STD DEV 0.1640

S.E.M. 0.0410

H H H H H SAMPLE SIZE 16

H HHHHHHH HH H MAXIMUM 0.0388

M--------------------M MINIMUM -0.5904

I AN H= 1 CASES A Z MAX 1.49

N (N= 16) X Z MIN -2.34

CASE (MAX) 4

CASE (MIN) 5

NUMBER OF INTEGER WORDS USED IN PRECEDING SUBPROBLEM 7967

/ FINISH

PROGRAM TERMINATED
