# Supplementary material for: Uneven distribution of enamel, dentine and cementum in cheek teeth of domestic horses (Equus caballus): A micro computed tomography study
Source: PLoS One. 2017 Aug 16;12(8):e0183220. doi: 10.1371/journal.pone.0183220 (PMC5558931; doi:10.1371/journal.pone.0183220)
Supplement: S3 File — Mean value of peripheral cementum of intra-alveolar positioned selected slides = 1 compared to mean value of peripheral cementum of extra-alveolar positioned selected slide = 3. (DOCX) [file pone.0183220.s005.docx]

UPPER CHEEK TEETH

BMDP3D - T-TESTS

Copyright 1977, 1979, 1981, 1982, 1983, 1985, 1987, 1988, 1990, 1993

by BMDP Statistical Software, Inc.

Statistical Solutions Ltd. | Statistical Solutions

Unit 1A, South Ring Business Park | Stonehill Corporate Center, Suite 104

Kinsale Road, Cork, Ireland | 999 Broadway, Saugus, MA 01906, USA

Phone: + 353 21 4319629 | Phone: 781.231.7680

Fax: + 353 21 4319630 | Fax: 781.231.7684

e-mail: sales@statsol.ie | e-mail: info@statsolusa.com

Website: http://www.statsol.ie | Website: http://www.statsolusa.com

Release: 8.1 (Windows 9x, 2000, Me, Xp) Date: 04/27/16 at 13:43:19

Manual: BMDP Manual Volumes 1, 2, and 3.

Digest: BMDP User's Digest.

IBM PC: BMDP PC Supplement -- Installation and Special Features.

PROGRAM INSTRUCTIONS

for s1 = 1, 1, 2. # Status bzgl. der Lokalisation im Zahnfach

s2 = 2, 3, 3.%

/prob title = 'Herr Lauritz Englisch: A3D5.inp *** Quantitative Studien

zum Schmelzgehalt in Pferdebackenzaehnen.

- 1 Fall = 1 Zahn = 10 Lokalisationen = 10 Zeilen

- Hier: Oberkiefer, Zahn 7 - 10

- Vergleich der Mittelwerte des Zahnzements

bzgl. der Lokalisation im Zahnfach

- Umrechnung der relativen Werte in Prozentwerte

- Berechnung der Mittelwerte "M" ueber die Schnittebenen

getrennt fuer innerhalb und ausserhalb des Zahnfachs

***'.

/inp var = 276.

file = a.

format = '32f10,9(/50x,27f10), /20x,1f10'. ## Mit Abstandsangabe für die Ebene

/var names = zahnid,zp,qu,znr,za,

for lo = 0 to 9.% ## Lokalisation

for va = lok,statu,ps,pz,isd,izd,ism,izm,idz,id,ges,sges,dz_sb,i_zb,

inf,ps_rel,s_rel,pz_r,isd_r,izd_r,ism_r,izm_r,idz_r,

id_r,sges_r,i_zb_r,inf_r.%

|va||lo|,%%

abst99. ## Mit Abstandsangabe für die Ebene 99

use = znr,

for va = pz,pz_r.%

for lo = 0 to 9.% ## Lokalisation

|va||lo|,%%

for va = pz,pz_r.%

M|s1||va|,M|s2||va|,%.

/trans use = ((znr ge 7) AND (znr le 10)).

# Umrechnung der relativen Werte in Prozentwerte

for lo = 0 to 9.%

pz_r|lo| = pz_r|lo| * 100.%

# Selektion der Schnittebenen nach Position bzgl. des Zahnfachs

for va = pz,pz_r.%

# status = s1

for lo = 0 to 9.%

tmp|lo| = XMIS. ## vorbesetzen

if (statu|lo| eq |s1| ) then tmp|lo| = |va||lo|.

%

M|s1||va| = mean(for lo = 0 to 9.% tmp|lo|,%).

# status = s2

for lo = 0 to 9.%

tmp|lo| = XMIS. ## vorbesetzen

if (statu|lo| eq |s2| ) then tmp|lo| = |va||lo|.

%

M|s2||va| = mean(for lo = 0 to 9.% tmp|lo|,%).

%

/matched

first = for va = pz,pz_r.% M|s1||va|,%.

second = for va = pz,pz_r.% M|s2||va|,%.

pair.

/print level = min.

case = 0.

/end

*** N O T E *** A FOR-%-LOOP ENDS AT THE END OF THE INSTRUCTIONS LISTED ABOVE.

IF THIS IS NOT WHAT YOU INTENDED, CHECK FOR A MISSING % SIGN.

--- PROGRAM INSTRUCTIONS AFTER "FOR %" EXPANSION ---

/prob title = 'Herr Lauritz Englisch: A3D5.inp *** Quantitative Studien

zum Schmelzgehalt in Pferdebackenzaehnen.

- 1 Fall = 1 Zahn = 10 Lokalisationen =

10 Zeilen - Hier: Oberkiefer,

Zahn 7 - 10 - Vergleich der Mittelwerte des Zahnzements bzgl.

der Lokalisation im Zahnfach - Umrechnung der relativen Werte in

Prozentwerte - Berechnung der Mittelwerte "M" ueber die

Schnittebenen getrennt fuer innerhalb und ausserhalb des

Zahnfachs ***'.

/inp var = 276. file = a. format = '32f10,9(/50x,27f10),

/20x,1f10'.

/var names = zahnid,zp,qu,znr,za, lok0, statu0, ps0, pz0, isd0, izd0,

ism0, izm0, idz0, id0, ges0, sges0, dz_sb0, i_zb0, inf0,

ps_rel0, s_rel0, pz_r0, isd_r0, izd_r0, ism_r0, izm_r0,

idz_r0, id_r0, sges_r0, i_zb_r0, inf_r0, lok1, statu1, ps1,

pz1, isd1, izd1, ism1, izm1, idz1, id1, ges1, sges1, dz_sb1,

i_zb1, inf1, ps_rel1, s_rel1, pz_r1, isd_r1, izd_r1, ism_r1,

izm_r1, idz_r1, id_r1, sges_r1, i_zb_r1, inf_r1, lok2,

statu2, ps2, pz2, isd2, izd2, ism2, izm2, idz2, id2, ges2,

sges2, dz_sb2, i_zb2, inf2, ps_rel2, s_rel2, pz_r2, isd_r2,

izd_r2, ism_r2, izm_r2, idz_r2, id_r2, sges_r2, i_zb_r2,

inf_r2, lok3, statu3, ps3, pz3, isd3, izd3, ism3, izm3,

idz3, id3, ges3, sges3, dz_sb3, i_zb3, inf3, ps_rel3, s_rel3,

pz_r3, isd_r3, izd_r3, ism_r3, izm_r3, idz_r3, id_r3,

sges_r3, i_zb_r3, inf_r3, lok4, statu4, ps4, pz4, isd4,

izd4, ism4, izm4, idz4, id4, ges4, sges4, dz_sb4, i_zb4,

inf4, ps_rel4, s_rel4, pz_r4, isd_r4, izd_r4, ism_r4, izm_r4,

idz_r4, id_r4, sges_r4, i_zb_r4, inf_r4, lok5, statu5, ps5,

pz5, isd5, izd5, ism5, izm5, idz5, id5, ges5, sges5, dz_sb5,

i_zb5, inf5, ps_rel5, s_rel5, pz_r5, isd_r5, izd_r5, ism_r5,

izm_r5, idz_r5, id_r5, sges_r5, i_zb_r5, inf_r5, lok6,

statu6, ps6, pz6, isd6, izd6, ism6, izm6, idz6, id6, ges6,

sges6, dz_sb6, i_zb6, inf6, ps_rel6, s_rel6, pz_r6, isd_r6,

izd_r6, ism_r6, izm_r6, idz_r6, id_r6, sges_r6, i_zb_r6,

inf_r6, lok7, statu7, ps7, pz7, isd7, izd7, ism7, izm7,

idz7, id7, ges7, sges7, dz_sb7, i_zb7, inf7, ps_rel7, s_rel7,

pz_r7, isd_r7, izd_r7, ism_r7, izm_r7, idz_r7, id_r7,

sges_r7, i_zb_r7, inf_r7, lok8, statu8, ps8, pz8, isd8,

izd8, ism8, izm8, idz8, id8, ges8, sges8, dz_sb8, i_zb8,

inf8, ps_rel8, s_rel8, pz_r8, isd_r8, izd_r8, ism_r8, izm_r8,

idz_r8, id_r8, sges_r8, i_zb_r8, inf_r8, lok9, statu9, ps9,

pz9, isd9, izd9, ism9, izm9, idz9, id9, ges9, sges9, dz_sb9,

i_zb9, inf9, ps_rel9, s_rel9, pz_r9, isd_r9, izd_r9, ism_r9,

izm_r9, idz_r9, id_r9, sges_r9, i_zb_r9, inf_r9, abst99.

use = znr, pz0, pz1, pz2, pz3, pz4, pz5, pz6, pz7,

pz8, pz9, pz_r0, pz_r1, pz_r2, pz_r3, pz_r4, pz_r5,

pz_r6, pz_r7, pz_r8, pz_r9, M1pz,M2pz, M1pz_r,M2pz_r.

/trans use = ((znr ge 7) AND (znr le 10)). pz_r0 = pz_r0 * 100.

pz_r1 = pz_r1 * 100. pz_r2 = pz_r2 * 100. pz_r3 = pz_r3 * 100.

pz_r4 = pz_r4 * 100. pz_r5 = pz_r5 * 100. pz_r6 = pz_r6 * 100.

pz_r7 = pz_r7 * 100. pz_r8 = pz_r8 * 100. pz_r9 = pz_r9 * 100.

tmp0 = XMIS. if (statu0 eq 1 ) then tmp0 = pz0. tmp1 = XMIS.

if (statu1 eq 1 ) then tmp1 = pz1. tmp2 = XMIS.

if (statu2 eq 1 ) then tmp2 = pz2. tmp3 = XMIS.

if (statu3 eq 1 ) then tmp3 = pz3. tmp4 = XMIS.

if (statu4 eq 1 ) then tmp4 = pz4. tmp5 = XMIS.

if (statu5 eq 1 ) then tmp5 = pz5. tmp6 = XMIS.

if (statu6 eq 1 ) then tmp6 = pz6. tmp7 = XMIS.

if (statu7 eq 1 ) then tmp7 = pz7. tmp8 = XMIS.

if (statu8 eq 1 ) then tmp8 = pz8. tmp9 = XMIS.

if (statu9 eq 1 ) then tmp9 = pz9.

M1pz = mean( tmp0, tmp1, tmp2, tmp3, tmp4, tmp5, tmp6, tmp7, tmp8,

tmp9). tmp0 = XMIS. if (statu0 eq 2 ) then tmp0 = pz0.

tmp1 = XMIS. if (statu1 eq 2 ) then tmp1 = pz1. tmp2 = XMIS.

if (statu2 eq 2 ) then tmp2 = pz2. tmp3 = XMIS.

if (statu3 eq 2 ) then tmp3 = pz3. tmp4 = XMIS.

if (statu4 eq 2 ) then tmp4 = pz4. tmp5 = XMIS.

if (statu5 eq 2 ) then tmp5 = pz5. tmp6 = XMIS.

if (statu6 eq 2 ) then tmp6 = pz6. tmp7 = XMIS.

if (statu7 eq 2 ) then tmp7 = pz7. tmp8 = XMIS.

if (statu8 eq 2 ) then tmp8 = pz8. tmp9 = XMIS.

if (statu9 eq 2 ) then tmp9 = pz9.

M2pz = mean( tmp0, tmp1, tmp2, tmp3, tmp4, tmp5, tmp6, tmp7, tmp8,

tmp9). tmp0 = XMIS. if (statu0 eq 1 ) then tmp0 = pz_r0.

tmp1 = XMIS. if (statu1 eq 1 ) then tmp1 = pz_r1. tmp2 = XMIS.

if (statu2 eq 1 ) then tmp2 = pz_r2. tmp3 = XMIS.

if (statu3 eq 1 ) then tmp3 = pz_r3. tmp4 = XMIS.

if (statu4 eq 1 ) then tmp4 = pz_r4. tmp5 = XMIS.

if (statu5 eq 1 ) then tmp5 = pz_r5. tmp6 = XMIS.

if (statu6 eq 1 ) then tmp6 = pz_r6. tmp7 = XMIS.

if (statu7 eq 1 ) then tmp7 = pz_r7. tmp8 = XMIS.

if (statu8 eq 1 ) then tmp8 = pz_r8. tmp9 = XMIS.

if (statu9 eq 1 ) then tmp9 = pz_r9.

M1pz_r = mean( tmp0, tmp1, tmp2, tmp3, tmp4, tmp5, tmp6, tmp7,

tmp8, tmp9). tmp0 = XMIS.

if (statu0 eq 2 ) then tmp0 = pz_r0. tmp1 = XMIS.

if (statu1 eq 2 ) then tmp1 = pz_r1. tmp2 = XMIS.

if (statu2 eq 2 ) then tmp2 = pz_r2. tmp3 = XMIS.

if (statu3 eq 2 ) then tmp3 = pz_r3. tmp4 = XMIS.

if (statu4 eq 2 ) then tmp4 = pz_r4. tmp5 = XMIS.

if (statu5 eq 2 ) then tmp5 = pz_r5. tmp6 = XMIS.

if (statu6 eq 2 ) then tmp6 = pz_r6. tmp7 = XMIS.

if (statu7 eq 2 ) then tmp7 = pz_r7. tmp8 = XMIS.

if (statu8 eq 2 ) then tmp8 = pz_r8. tmp9 = XMIS.

if (statu9 eq 2 ) then tmp9 = pz_r9.

M2pz_r = mean( tmp0, tmp1, tmp2, tmp3, tmp4, tmp5, tmp6, tmp7,

tmp8, tmp9).

/matched first = M1pz, M1pz_r. second = M2pz, M2pz_r. pair.

/print level = min. case = 0.

/end/

NUMBER OF CASES READ. . . . . . . . . . . . . . 28

CASES WITH USE SET TO ZERO . . . . . . . . . 8

REMAINING NUMBER OF CASES . . . . . . . . 20

DESCRIPTIVE STATISTICS OF DATA

----------- ---------- -- ----

VARIABLE TOTAL STANDARD ST.ERR COEFF S M A L L E S T L A R G E S T

NO. NAME FREQ. MEAN DEV. OF MEAN OF VAR VALUE Z-SCR CASE VALUE Z-SCR CASE RANGE

4 znr 20 8.5000 1.1471 .25649 .13495 7.0000 -1.31 6 10.000 1.31 2 3.0000

9 pz0 19 27.856 12.064 2.7676 .43307 10.680 -1.42 5 57.830 2.48 10 47.150

36 pz1 19 76.448 40.825 9.3659 .53402 30.350 -1.13 18 165.71 2.19 10 135.36

63 pz2 19 104.01 59.192 13.580 .56912 33.970 -1.18 18 201.47 1.65 10 167.50

90 pz3 9 62.862 29.859 9.9528 .47498 36.950 -0.87 19 133.22 2.36 11 96.270

117 pz4 9 107.63 49.818 16.606 .46284 43.970 -1.28 18 180.19 1.46 7 136.22

144 pz5 5 133.81 61.615 27.555 .46047 67.240 -1.08 18 206.04 1.17 25 138.80

171 pz6 2 144.55 8.2095 5.8050 .05679 138.75 -0.71 18 150.36 0.71 5 11.610

198 pz7 0

225 pz8 0

252 pz9 20 143.61 33.160 7.4148 .23090 58.230 -2.57 8 185.52 1.26 17 127.29

23 pz_r0 18 5.1635 2.0452 .48205 .39608 1.8497 -1.62 5 9.2853 2.02 26 7.4356

50 pz_r1 19 13.779 9.3213 2.1384 .67648 4.8429 -0.96 25 43.549 3.19 28 38.707

77 pz_r2 19 17.055 10.365 2.3778 .60773 5.3265 -1.13 9 44.553 2.65 28 39.227

104 pz_r3 9 10.176 5.1604 1.7201 .50713 6.5927 -0.69 19 22.622 2.41 11 16.029

131 pz_r4 9 15.560 6.2185 2.0728 .39964 7.8924 -1.23 18 25.340 1.57 7 17.448

158 pz_r5 5 18.340 7.1584 3.2013 .39031 11.479 -0.96 18 26.208 1.10 19 14.729

185 pz_r6 2 19.686 1.0292 .72779 .05228 18.958 -0.71 5 20.414 0.71 18 1.4556

212 pz_r7 0

239 pz_r8 0

266 pz_r9 20 21.576 4.5885 1.0260 .21267 12.054 -2.08 8 35.276 2.99 28 23.222

287 M1pz 20 43.847 11.759 2.6294 .26818 23.980 -1.69 2 71.495 2.35 8 47.515

288 M2pz 16 114.69 36.185 9.0462 .31551 58.550 -1.55 19 173.96 1.64 3 115.41

289 M1pz_r 19 7.8440 2.3470 .53843 .29921 4.4662 -1.44 2 13.273 2.31 8 8.8068

290 M2pz_r 16 18.918 8.1449 2.0362 .43054 8.8393 -1.24 9 43.549 3.02 28 34.710

NUMBER OF CASES READ. . . . . . . . . . . . . . 28

CASES WITH USE SET TO ZERO . . . . . . . . . 8

REMAINING NUMBER OF CASES . . . . . . . . 20

NUMBER OF INTEGER WORDS USED IN PRECEDING SUBPROBLEM 8841

/prob title = 'Herr Lauritz Englisch: A3D5.inp *** Quantitative Studien

zum Schmelzgehalt in Pferdebackenzaehnen.

- 1 Fall = 1 Zahn = 10 Lokalisationen =

10 Zeilen - Hier: Oberkiefer,

Zahn 7 - 10 - Vergleich der Mittelwerte des Zahnzements bzgl.

der Lokalisation im Zahnfach - Umrechnung der relativen Werte in

Prozentwerte - Berechnung der Mittelwerte "M" ueber die

Schnittebenen getrennt fuer innerhalb und ausserhalb des

Zahnfachs ***'.

/inp var = 276. file = a. format = '32f10,9(/50x,27f10),

/20x,1f10'.

*** N O T E *** THE ABOVE INSTRUCTIONS ARE ASSUMED TO BE THE START OF A NEW PROBLEM. IF THIS IS NOT YOUR INTENT, PLACE A

POUND-SIGN (#) AFTER THE SLASH OR DOLLAR-SIGN WHICH ENDS THE PARAGRAPH.

NUMBER OF INTEGER WORDS USED IN PRECEDING PROBLEM 4891

BMDP3D - T-TESTS

VARIABLE TOTAL STANDARD ST.ERR COEFF S M A L L E S T L A R G E S T

NO. NAME FREQ. MEAN DEV. OF MEAN OF VAR VALUE Z-SCR CASE VALUE Z-SCR CASE RANGE

4 znr 20 8.5000 1.1471 .25649 .13495 7.0000 -1.31 6 10.000 1.31 2 3.0000

9 pz0 19 27.856 12.064 2.7676 .43307 10.680 -1.42 5 57.830 2.48 10 47.150

36 pz1 19 76.448 40.825 9.3659 .53402 30.350 -1.13 18 165.71 2.19 10 135.36

63 pz2 19 104.01 59.192 13.580 .56912 33.970 -1.18 18 201.47 1.65 10 167.50

90 pz3 9 62.862 29.859 9.9528 .47498 36.950 -0.87 19 133.22 2.36 11 96.270

117 pz4 9 107.63 49.818 16.606 .46284 43.970 -1.28 18 180.19 1.46 7 136.22

144 pz5 5 133.81 61.615 27.555 .46047 67.240 -1.08 18 206.04 1.17 25 138.80

171 pz6 2 144.55 8.2095 5.8050 .05679 138.75 -0.71 18 150.36 0.71 5 11.610

198 pz7 0

225 pz8 0

252 pz9 20 143.61 33.160 7.4148 .23090 58.230 -2.57 8 185.52 1.26 17 127.29

23 pz_r0 18 5.1635 2.0452 .48205 .39608 1.8497 -1.62 5 9.2853 2.02 26 7.4356

50 pz_r1 19 13.779 9.3213 2.1384 .67648 4.8429 -0.96 25 43.549 3.19 28 38.707

77 pz_r2 19 17.055 10.365 2.3778 .60773 5.3265 -1.13 9 44.553 2.65 28 39.227

104 pz_r3 9 10.176 5.1604 1.7201 .50713 6.5927 -0.69 19 22.622 2.41 11 16.029

131 pz_r4 9 15.560 6.2185 2.0728 .39964 7.8924 -1.23 18 25.340 1.57 7 17.448

158 pz_r5 5 18.340 7.1584 3.2013 .39031 11.479 -0.96 18 26.208 1.10 19 14.729

185 pz_r6 2 19.686 1.0292 .72779 .05228 18.958 -0.71 5 20.414 0.71 18 1.4556

212 pz_r7 0

239 pz_r8 0

266 pz_r9 20 21.576 4.5885 1.0260 .21267 12.054 -2.08 8 35.276 2.99 28 23.222

287 M1pz 20 43.847 11.759 2.6294 .26818 23.980 -1.69 2 71.495 2.35 8 47.515

288 M3pz 20 148.80 32.527 7.2733 .21860 58.230 -2.78 8 185.48 1.13 6 127.25

289 M1pz_r 19 7.8440 2.3470 .53843 .29921 4.4662 -1.44 2 13.273 2.31 8 8.8068

290 M3pz_r 20 22.298 5.3232 1.1903 .23873 12.054 -1.92 8 39.914 3.31 28 27.861

NUMBER OF CASES READ. . . . . . . . . . . . . . 28

CASES WITH USE SET TO ZERO . . . . . . . . . 8

REMAINING NUMBER OF CASES . . . . . . . . 20

**********************************************************

M1pz VS. M3pz (VAR. NO. 287 VS. 288)

********************************************

M1pz M3pz M1pz M3pz

-------------------------------

HH MEAN 43.8474 148.7978

HH X

HHH X STD DEV 11.7590 32.5273

HHH XX X S.E.M. 2.6294 7.2733

HHHH XXXXX X SAMPLE SIZE 20 20

HHHHHH X XXXXXXXX MAXIMUM 71.4950 185.4800

M--------------------M M--------------------M MINIMUM 23.9800 58.2300

I AN H= 1 CASES A I AN X= 1 CASES A Z MAX 2.35 1.13

N (N= 20) X N (N= 20) X Z MIN -1.69 -2.78

CASE (MAX) 8 6

CASE (MIN) 2 8

M1pz - M3pz (VAR. NO. 287 - 288)

***************************************

M1pz - M3pz TEST STATISTICS P-VALUE DF

------------------- --------------------------------

MEAN -104.9503 MATCHED T -12.17 0.0000 19

H STD DEV 38.5602

H HH S.E.M. 8.6223

HH HHHH SAMPLE SIZE 20

HHHHHHH H H H MAXIMUM 13.2650 CORRELATION -0.3799 0.0903 18

M--------------------M MINIMUM -151.7700

I AN H= 1 CASES A Z MAX 3.07

N (N= 20) X Z MIN -1.21

CASE (MAX) 8

CASE (MIN) 17

**********************************************************

M1pz_r VS. M3pz_r (VAR. NO. 289 VS. 290)

********************************************

M1pz_r M3pz_r M1pz_r M3pz_r

-------------------------------

X MEAN 7.8440 22.4098

XX

H XX STD DEV 2.3470 5.4448

HH XXXX S.E.M. 0.5384 1.2491

HH H XXXX SAMPLE SIZE 19 19

HHHH X XXXX X MAXIMUM 13.2731 39.9144

M--------------------M M--------------------M MINIMUM 4.4662 12.0539

I AN H= 2 CASES A I AN X= 1 CASES A Z MAX 2.31 3.21

N (N= 19) X N (N= 19) X Z MIN -1.44 -1.90

CASE (MAX) 8 28

CASE (MIN) 2 8

M1pz_r - M3pz_r (VAR. NO. 289 - 290)

***************************************

M1pz_r - M3pz_r TEST STATISTICS P-VALUE DF

------------------- --------------------------------

MEAN -14.5658 MATCHED T -9.76 0.0000 18

HH STD DEV 6.5074

HH S.E.M. 1.4929

HHHHHH SAMPLE SIZE 19

H HHHHHHH H MAXIMUM 1.2192 CORRELATION -0.2814 0.2304 17

M--------------------M MINIMUM -33.0555

I AN H= 1 CASES A Z MAX 2.43

N (N= 19) X Z MIN -2.84

CASE (MAX) 8

CASE (MIN) 28

LOWER CHEEK TEETH

MDP3D - T-TESTS

Copyright 1977, 1979, 1981, 1982, 1983, 1985, 1987, 1988, 1990, 1993

by BMDP Statistical Software, Inc.

Statistical Solutions Ltd. | Statistical Solutions

Unit 1A, South Ring Business Park | Stonehill Corporate Center, Suite 104

Kinsale Road, Cork, Ireland | 999 Broadway, Saugus, MA 01906, USA

Phone: + 353 21 4319629 | Phone: 781.231.7680

Fax: + 353 21 4319630 | Fax: 781.231.7684

e-mail: sales@statsol.ie | e-mail: info@statsolusa.com

Website: http://www.statsol.ie | Website: http://www.statsolusa.com

Release: 8.1 (Windows 9x, 2000, Me, Xp) Date: 04/28/16 at 12:28:22

Manual: BMDP Manual Volumes 1, 2, and 3.

Digest: BMDP User's Digest.

IBM PC: BMDP PC Supplement -- Installation and Special Features.

PROGRAM INSTRUCTIONS

for s1 = 1, 1, 2. # Status bzgl. der Lokalisation im Zahnfach

s2 = 2, 3, 3.%

/prob title = 'Herr Lauritz Englisch: B3D5.inp *** Quantitative Studien

zum Schmelzgehalt in Pferdebackenzaehnen.

- 1 Fall = 1 Zahn = 10 Lokalisationen = 10 Zeilen

- Hier: Oberkiefer, Zahn 7 - 10

- Vergleich der Mittelwerte des Zahnzements

bzgl. der Lokalisation im Zahnfach

- Umrechnung der relativen Werte in Prozentwerte

- Berechnung der Mittelwerte "M" ueber die Schnittebenen

getrennt fuer innerhalb und ausserhalb des Zahnfachs

***'.

/inp var = 87.

file = b.

format = '14f10,8(/50x,9f10), /20x,1f10'. ## Mit Abstandsangabe für die Ebene 9

/var names = zahnid,zp,qu,znr,za,

for lo = 0 to 8.% ## Lokalisation

for va = lok,statu,ps,pz,id,ges,s_rel,z_rel,d_rel.%

|va||lo|,%%

abst99. ## Mit Abstandsangabe für die Ebene 99

use = znr,

for va = pz,z_rel.%

for lo = 0 to 8.% ## Lokalisation

|va||lo|,%%

for va = pz,z_rel.%

M|s1||va|,M|s2||va|,%.

/trans use = ((znr ge 7) AND (znr le 10)).

# Umrechnung der relativen Werte in Prozentwerte

for lo = 0 to 8.%

z_rel|lo| = z_rel|lo| * 100.%

# Selektion der Schnittebenen nach Position bzgl. des Zahnfachs

for va = pz,z_rel.%

# status = s1

for lo = 0 to 8.%

tmp|lo| = XMIS. ## vorbesetzen

if (statu|lo| eq |s1| ) then tmp|lo| = |va||lo|.

%

M|s1||va| = mean(for lo = 0 to 8.% tmp|lo|,%).

# status = s2

for lo = 0 to 8.%

tmp|lo| = XMIS. ## vorbesetzen

if (statu|lo| eq |s2| ) then tmp|lo| = |va||lo|.

%

M|s2||va| = mean(for lo = 0 to 8.% tmp|lo|,%).

%

/matched

first = for va = pz,z_rel.% M|s1||va|,%.

second = for va = pz,z_rel.% M|s2||va|,%.

pair.

/print level = min.

case = 0.

/end

*** N O T E *** A FOR-%-LOOP ENDS AT THE END OF THE INSTRUCTIONS LISTED ABOVE.

IF THIS IS NOT WHAT YOU INTENDED, CHECK FOR A MISSING % SIGN.

--- PROGRAM INSTRUCTIONS AFTER "FOR %" EXPANSION ---

/prob title = 'Herr Lauritz Englisch: B3D5.inp *** Quantitative Studien

zum Schmelzgehalt in Pferdebackenzaehnen.

- 1 Fall = 1 Zahn = 10 Lokalisationen =

10 Zeilen - Hier: Oberkiefer,

Zahn 7 - 10 - Vergleich der Mittelwerte des Zahnzements bzgl.

der Lokalisation im Zahnfach - Umrechnung der relativen Werte in

Prozentwerte - Berechnung der Mittelwerte "M" ueber die

Schnittebenen getrennt fuer innerhalb und ausserhalb des

Zahnfachs ***'.

/inp var = 87. file = b. format = '14f10,8(/50x,9f10),

/20x,1f10'.

/var names = zahnid,zp,qu,znr,za, lok0, statu0, ps0, pz0, id0, ges0,

s_rel0, z_rel0, d_rel0, lok1, statu1, ps1, pz1, id1, ges1,

s_rel1, z_rel1, d_rel1, lok2, statu2, ps2, pz2, id2, ges2,

s_rel2, z_rel2, d_rel2, lok3, statu3, ps3, pz3, id3, ges3,

s_rel3, z_rel3, d_rel3, lok4, statu4, ps4, pz4, id4, ges4,

s_rel4, z_rel4, d_rel4, lok5, statu5, ps5, pz5, id5, ges5,

s_rel5, z_rel5, d_rel5, lok6, statu6, ps6, pz6, id6, ges6,

s_rel6, z_rel6, d_rel6, lok7, statu7, ps7, pz7, id7, ges7,

s_rel7, z_rel7, d_rel7, lok8, statu8, ps8, pz8, id8, ges8,

s_rel8, z_rel8, d_rel8, abst99.

use = znr, pz0, pz1, pz2, pz3, pz4, pz5, pz6, pz7,

pz8, z_rel0, z_rel1, z_rel2, z_rel3, z_rel4, z_rel5,

z_rel6, z_rel7, z_rel8, M1pz,M2pz, M1z_rel,M2z_rel.

/trans use = ((znr ge 7) AND (znr le 10)). z_rel0 = z_rel0 * 100.

z_rel1 = z_rel1 * 100. z_rel2 = z_rel2 * 100.

z_rel3 = z_rel3 * 100. z_rel4 = z_rel4 * 100.

z_rel5 = z_rel5 * 100. z_rel6 = z_rel6 * 100.

z_rel7 = z_rel7 * 100. z_rel8 = z_rel8 * 100. tmp0 = XMIS.

if (statu0 eq 1 ) then tmp0 = pz0. tmp1 = XMIS.

if (statu1 eq 1 ) then tmp1 = pz1. tmp2 = XMIS.

if (statu2 eq 1 ) then tmp2 = pz2. tmp3 = XMIS.

if (statu3 eq 1 ) then tmp3 = pz3. tmp4 = XMIS.

if (statu4 eq 1 ) then tmp4 = pz4. tmp5 = XMIS.

if (statu5 eq 1 ) then tmp5 = pz5. tmp6 = XMIS.

if (statu6 eq 1 ) then tmp6 = pz6. tmp7 = XMIS.

if (statu7 eq 1 ) then tmp7 = pz7. tmp8 = XMIS.

if (statu8 eq 1 ) then tmp8 = pz8.

M1pz = mean( tmp0, tmp1, tmp2, tmp3, tmp4, tmp5, tmp6, tmp7,

tmp8). tmp0 = XMIS. if (statu0 eq 2 ) then tmp0 = pz0.

tmp1 = XMIS. if (statu1 eq 2 ) then tmp1 = pz1. tmp2 = XMIS.

if (statu2 eq 2 ) then tmp2 = pz2. tmp3 = XMIS.

if (statu3 eq 2 ) then tmp3 = pz3. tmp4 = XMIS.

if (statu4 eq 2 ) then tmp4 = pz4. tmp5 = XMIS.

if (statu5 eq 2 ) then tmp5 = pz5. tmp6 = XMIS.

if (statu6 eq 2 ) then tmp6 = pz6. tmp7 = XMIS.

if (statu7 eq 2 ) then tmp7 = pz7. tmp8 = XMIS.

if (statu8 eq 2 ) then tmp8 = pz8.

M2pz = mean( tmp0, tmp1, tmp2, tmp3, tmp4, tmp5, tmp6, tmp7,

tmp8). tmp0 = XMIS. if (statu0 eq 1 ) then tmp0 = z_rel0.

tmp1 = XMIS. if (statu1 eq 1 ) then tmp1 = z_rel1. tmp2 = XMIS.

if (statu2 eq 1 ) then tmp2 = z_rel2. tmp3 = XMIS.

if (statu3 eq 1 ) then tmp3 = z_rel3. tmp4 = XMIS.

if (statu4 eq 1 ) then tmp4 = z_rel4. tmp5 = XMIS.

if (statu5 eq 1 ) then tmp5 = z_rel5. tmp6 = XMIS.

if (statu6 eq 1 ) then tmp6 = z_rel6. tmp7 = XMIS.

if (statu7 eq 1 ) then tmp7 = z_rel7. tmp8 = XMIS.

if (statu8 eq 1 ) then tmp8 = z_rel8.

M1z_rel = mean( tmp0, tmp1, tmp2, tmp3, tmp4, tmp5, tmp6, tmp7,

tmp8). tmp0 = XMIS. if (statu0 eq 2 ) then tmp0 = z_rel0.

tmp1 = XMIS. if (statu1 eq 2 ) then tmp1 = z_rel1. tmp2 = XMIS.

if (statu2 eq 2 ) then tmp2 = z_rel2. tmp3 = XMIS.

if (statu3 eq 2 ) then tmp3 = z_rel3. tmp4 = XMIS.

if (statu4 eq 2 ) then tmp4 = z_rel4. tmp5 = XMIS.

if (statu5 eq 2 ) then tmp5 = z_rel5. tmp6 = XMIS.

if (statu6 eq 2 ) then tmp6 = z_rel6. tmp7 = XMIS.

if (statu7 eq 2 ) then tmp7 = z_rel7. tmp8 = XMIS.

if (statu8 eq 2 ) then tmp8 = z_rel8.

M2z_rel = mean( tmp0, tmp1, tmp2, tmp3, tmp4, tmp5, tmp6, tmp7,

tmp8).

/matched first = M1pz, M1z_rel. second = M2pz, M2z_rel. pair.

NUMBER OF CASES READ. . . . . . . . . . . . . . 26

CASES WITH USE SET TO ZERO . . . . . . . . . 10

REMAINING NUMBER OF CASES . . . . . . . . 16

/prob title = 'Herr Lauritz Englisch: B3D5.inp *** Quantitative Studien

zum Schmelzgehalt in Pferdebackenzaehnen.

- 1 Fall = 1 Zahn = 10 Lokalisationen =

10 Zeilen - Hier: Oberkiefer,

Zahn 7 - 10 - Vergleich der Mittelwerte des Zahnzements bzgl.

der Lokalisation im Zahnfach - Umrechnung der relativen Werte in

Prozentwerte - Berechnung der Mittelwerte "M" ueber die

Schnittebenen getrennt fuer innerhalb und ausserhalb des

Zahnfachs ***'.

/inp var = 87. file = b. format = '14f10,8(/50x,9f10),

/20x,1f10'.

*** N O T E *** THE ABOVE INSTRUCTIONS ARE ASSUMED TO BE THE START OF A NEW PROBLEM. IF THIS IS NOT YOUR INTENT, PLACE A

POUND-SIGN (#) AFTER THE SLASH OR DOLLAR-SIGN WHICH ENDS THE PARAGRAPH.

NUMBER OF INTEGER WORDS USED IN PRECEDING PROBLEM 2833

BMDP3D - T-TESTS

Release: 8.1 (Windows 9x, 2000, Me, Xp) Date: 04/28/16 at 12:28:22

(CONTINUED FROM ABOVE)

/var names = zahnid,zp,qu,znr,za, lok0, statu0, ps0, pz0, id0, ges0,

s_rel0, z_rel0, d_rel0, lok1, statu1, ps1, pz1, id1, ges1,

s_rel1, z_rel1, d_rel1, lok2, statu2, ps2, pz2, id2, ges2,

s_rel2, z_rel2, d_rel2, lok3, statu3, ps3, pz3, id3, ges3,

s_rel3, z_rel3, d_rel3, lok4, statu4, ps4, pz4, id4, ges4,

s_rel4, z_rel4, d_rel4, lok5, statu5, ps5, pz5, id5, ges5,

s_rel5, z_rel5, d_rel5, lok6, statu6, ps6, pz6, id6, ges6,

s_rel6, z_rel6, d_rel6, lok7, statu7, ps7, pz7, id7, ges7,

s_rel7, z_rel7, d_rel7, lok8, statu8, ps8, pz8, id8, ges8,

s_rel8, z_rel8, d_rel8, abst99.

use = znr, pz0, pz1, pz2, pz3, pz4, pz5, pz6, pz7,

pz8, z_rel0, z_rel1, z_rel2, z_rel3, z_rel4, z_rel5,

z_rel6, z_rel7, z_rel8, M1pz,M3pz, M1z_rel,M3z_rel.

/trans use = ((znr ge 7) AND (znr le 10)). z_rel0 = z_rel0 * 100.

z_rel1 = z_rel1 * 100. z_rel2 = z_rel2 * 100.

z_rel3 = z_rel3 * 100. z_rel4 = z_rel4 * 100.

z_rel5 = z_rel5 * 100. z_rel6 = z_rel6 * 100.

z_rel7 = z_rel7 * 100. z_rel8 = z_rel8 * 100. tmp0 = XMIS.

if (statu0 eq 1 ) then tmp0 = pz0. tmp1 = XMIS.

if (statu1 eq 1 ) then tmp1 = pz1. tmp2 = XMIS.

if (statu2 eq 1 ) then tmp2 = pz2. tmp3 = XMIS.

if (statu3 eq 1 ) then tmp3 = pz3. tmp4 = XMIS.

if (statu4 eq 1 ) then tmp4 = pz4. tmp5 = XMIS.

if (statu5 eq 1 ) then tmp5 = pz5. tmp6 = XMIS.

if (statu6 eq 1 ) then tmp6 = pz6. tmp7 = XMIS.

if (statu7 eq 1 ) then tmp7 = pz7. tmp8 = XMIS.

if (statu8 eq 1 ) then tmp8 = pz8.

M1pz = mean( tmp0, tmp1, tmp2, tmp3, tmp4, tmp5, tmp6, tmp7,

tmp8). tmp0 = XMIS. if (statu0 eq 3 ) then tmp0 = pz0.

tmp1 = XMIS. if (statu1 eq 3 ) then tmp1 = pz1. tmp2 = XMIS.

if (statu2 eq 3 ) then tmp2 = pz2. tmp3 = XMIS.

if (statu3 eq 3 ) then tmp3 = pz3. tmp4 = XMIS.

if (statu4 eq 3 ) then tmp4 = pz4. tmp5 = XMIS.

if (statu5 eq 3 ) then tmp5 = pz5. tmp6 = XMIS.

if (statu6 eq 3 ) then tmp6 = pz6. tmp7 = XMIS.

if (statu7 eq 3 ) then tmp7 = pz7. tmp8 = XMIS.

if (statu8 eq 3 ) then tmp8 = pz8.

M3pz = mean( tmp0, tmp1, tmp2, tmp3, tmp4, tmp5, tmp6, tmp7,

tmp8). tmp0 = XMIS. if (statu0 eq 1 ) then tmp0 = z_rel0.

tmp1 = XMIS. if (statu1 eq 1 ) then tmp1 = z_rel1. tmp2 = XMIS.

if (statu2 eq 1 ) then tmp2 = z_rel2. tmp3 = XMIS.

if (statu3 eq 1 ) then tmp3 = z_rel3. tmp4 = XMIS.

if (statu4 eq 1 ) then tmp4 = z_rel4. tmp5 = XMIS.

if (statu5 eq 1 ) then tmp5 = z_rel5. tmp6 = XMIS.

if (statu6 eq 1 ) then tmp6 = z_rel6. tmp7 = XMIS.

if (statu7 eq 1 ) then tmp7 = z_rel7. tmp8 = XMIS.

if (statu8 eq 1 ) then tmp8 = z_rel8.

M1z_rel = mean( tmp0, tmp1, tmp2, tmp3, tmp4, tmp5, tmp6, tmp7,

tmp8). tmp0 = XMIS. if (statu0 eq 3 ) then tmp0 = z_rel0.

tmp1 = XMIS. if (statu1 eq 3 ) then tmp1 = z_rel1. tmp2 = XMIS.

if (statu2 eq 3 ) then tmp2 = z_rel2. tmp3 = XMIS.

if (statu3 eq 3 ) then tmp3 = z_rel3. tmp4 = XMIS.

if (statu4 eq 3 ) then tmp4 = z_rel4. tmp5 = XMIS.

if (statu5 eq 3 ) then tmp5 = z_rel5. tmp6 = XMIS.

if (statu6 eq 3 ) then tmp6 = z_rel6. tmp7 = XMIS.

if (statu7 eq 3 ) then tmp7 = z_rel7. tmp8 = XMIS.

if (statu8 eq 3 ) then tmp8 = z_rel8.

M3z_rel = mean( tmp0, tmp1, tmp2, tmp3, tmp4, tmp5, tmp6, tmp7,

tmp8).

/matched first = M1pz, M1z_rel. second = M3pz, M3z_rel. pair.

/print level = min. case = 0.

/end/

NUMBER OF CASES READ. . . . . . . . . . . . . . 26

CASES WITH USE SET TO ZERO . . . . . . . . . 10

REMAINING NUMBER OF CASES . . . . . . . . 16

DESCRIPTIVE STATISTICS OF DATA

----------- ---------- -- ----

VARIABLE TOTAL STANDARD ST.ERR COEFF S M A L L E S T L A R G E S T

NO. NAME FREQ. MEAN DEV. OF MEAN OF VAR VALUE Z-SCR CASE VALUE Z-SCR CASE RANGE

4 znr 16 8.0000 1.1547 .28868 .14434 7.0000 -0.87 1 10.000 1.73 16 3.0000

9 pz0 16 50.933 35.626 8.9066 .69947 16.270 -0.97 20 123.60 2.04 4 107.33

18 pz1 16 109.45 64.351 16.088 .58795 32.440 -1.20 16 208.98 1.55 5 176.54

27 pz2 16 159.88 83.986 20.996 .52530 40.650 -1.42 16 292.54 1.58 13 251.89

36 pz3 9 142.12 84.246 28.082 .59277 51.940 -1.07 16 279.64 1.63 4 227.70

45 pz4 5 96.750 35.789 16.006 .36992 55.760 -1.15 16 137.65 1.14 1 81.890

54 pz5 5 162.12 89.864 40.188 .55431 65.850 -1.07 16 267.20 1.17 22 201.35

63 pz6 4 208.12 27.576 13.788 .13250 187.00 -0.77 16 247.63 1.43 15 60.630

72 pz7 1 253.18 0.0000 0.0000 0.0000 253.18 20 253.18 20 0.0000

81 pz8 16 165.91 42.773 10.693 .25781 92.850 -1.71 24 257.61 2.14 13 164.76

13 z_rel0 16 14.681 8.4979 2.1245 .57883 4.9000 -1.15 20 27.000 1.45 23 22.100

22 z_rel1 16 25.912 11.793 2.9481 .45509 10.600 -1.30 16 42.300 1.39 23 31.700

31 z_rel2 16 33.500 13.764 3.4410 .41086 12.600 -1.52 15 49.900 1.19 13 37.300

40 z_rel3 9 28.722 11.480 3.8266 .39969 15.600 -1.14 16 43.000 1.24 4 27.400

49 z_rel4 5 22.640 5.3984 2.4142 .23845 16.200 -1.19 16 29.200 1.22 1 13.000

58 z_rel5 5 32.080 10.560 4.7224 .32917 19.500 -1.19 16 43.000 1.03 1 23.500

67 z_rel6 4 40.475 5.0408 2.5204 .12454 36.200 -0.85 20 47.600 1.41 15 11.400

76 z_rel7 1 43.700 0.0000 0.0000 0.0000 43.700 20 43.700 20 0.0000

85 z_rel8 16 35.956 4.2417 1.0604 .11797 26.600 -2.21 23 46.800 2.56 13 20.200

97 M1pz 16 71.677 23.757 5.9392 .33144 38.650 -1.39 24 126.72 2.32 4 88.070

98 M3pz 16 192.95 38.738 9.6845 .20077 129.62 -1.63 24 275.08 2.12 13 145.46

99 M1z_rel 16 19.361 4.1758 1.0440 .21568 13.567 -1.39 16 27.000 1.83 23 13.433

100 M3z_rel 16 39.456 3.2734 .81836 .08296 36.000 -1.06 5 48.350 2.72 13 12.350

NUMBER OF CASES READ. . . . . . . . . . . . . . 26

CASES WITH USE SET TO ZERO . . . . . . . . . 10

REMAINING NUMBER OF CASES . . . . . . . . 16

**********************************************************

M1pz VS. M3pz (VAR. NO. 97 VS. 98)

********************************************

M1pz M3pz M1pz M3pz

-------------------------------

MEAN 71.6771 192.9478

H STD DEV 23.7570 38.7379

HHHH X X X S.E.M. 5.9392 9.6845

HHHHH X X X SAMPLE SIZE 16 16

HHHHH H XXXXXXXXX X MAXIMUM 126.7200 275.0750

M--------------------M M--------------------M MINIMUM 38.6500 129.6150

I AN H= 1 CASES A I AN X= 1 CASES A Z MAX 2.32 2.12

N (N= 16) X N (N= 16) X Z MIN -1.39 -1.63

CASE (MAX) 4 13

CASE (MIN) 24 24

M1pz - M3pz (VAR. NO. 97 - 98)

***************************************

M1pz - M3pz TEST STATISTICS P-VALUE DF

------------------- --------------------------------

MEAN -121.2707 MATCHED T -14.95 0.0000 15

STD DEV 32.4385

H S.E.M. 8.1096

H HHH SAMPLE SIZE 16

H HH HHHHHH HH MAXIMUM -66.5400 CORRELATION 0.5502 0.0230 14

M--------------------M MINIMUM -200.7150

I AN H= 1 CASES A Z MAX 1.69

N (N= 16) X Z MIN -2.45

CASE (MAX) 23

CASE (MIN) 13

**********************************************************

M1z_rel VS. M3z_rel (VAR. NO. 99 VS. 100)

********************************************

M1z_rel M3z_rel M1z_rel M3z_rel

-------------------------------

MEAN 19.3610 39.4563

H XX

H XX STD DEV 4.1758 3.2734

HHH XXX S.E.M. 1.0440 0.8184

HHH H XXXX SAMPLE SIZE 16 16

HHHHHHH XXXX X MAXIMUM 27.0000 48.3500

M--------------------M M--------------------M MINIMUM 13.5667 36.0000

I AN H= 1 CASES A I AN X= 1 CASES A Z MAX 1.83 2.72

N (N= 16) X N (N= 16) X Z MIN -1.39 -1.06

CASE (MAX) 23 13

CASE (MIN) 16 5

M1z_rel - M3z_rel (VAR. NO. 99 - 100)

***************************************

M1z_rel - M3z_rel TEST STATISTICS P-VALUE DF

------------------- --------------------------------

MEAN -20.0952 MATCHED T -15.92 0.0000 15

STD DEV 5.0483

S.E.M. 1.2621

H H H H SAMPLE SIZE 16

H HHHHHHHH HH H MAXIMUM -10.0000 CORRELATION 0.0976 0.7098 14

M--------------------M MINIMUM -27.5700

I AN H= 1 CASES A Z MAX 2.00

N (N= 16) X Z MIN -1.48

CASE (MAX) 23

CASE (MIN) 15

NUMBER OF INTEGER WORDS USED IN PRECEDING SUBPROBLEM 5479

/prob title = 'Herr Lauritz Englisch: B3D5.inp *** Quantitative Studien

zum Schmelzgehalt in Pferdebackenzaehnen.

- 1 Fall = 1 Zahn = 10 Lokalisationen =

10 Zeilen - Hier: Oberkiefer,

Zahn 7 - 10 - Vergleich der Mittelwerte des Zahnzements bzgl.

der Lokalisation im Zahnfach - Umrechnung der relativen Werte in

Prozentwerte - Berechnung der Mittelwerte "M" ueber die

Schnittebenen getrennt fuer innerhalb und ausserhalb des

Zahnfachs ***'.

/inp var = 87. file = b. format = '14f10,8(/50x,9f10),

/20x,1f10'.

*** N O T E *** THE ABOVE INSTRUCTIONS ARE ASSUMED TO BE THE START OF A NEW PROBLEM. IF THIS IS NOT YOUR INTENT, PLACE A

POUND-SIGN (#) AFTER THE SLASH OR DOLLAR-SIGN WHICH ENDS THE PARAGRAPH.

NUMBER OF INTEGER WORDS USED IN PRECEDING PROBLEM 2833
