# Supplementary material for: Uneven distribution of enamel, dentine and cementum in cheek teeth of domestic horses (Equus caballus): A micro computed tomography study
Source: PLoS One. 2017 Aug 16;12(8):e0183220. doi: 10.1371/journal.pone.0183220 (PMC5558931; doi:10.1371/journal.pone.0183220)
Supplement: S4 File — For each measured variable the intra-class correlation coefficent (ICC) was calculated being based on the one-way analysis of variance with the horse as the class defining variable. (DOCX) [file pone.0183220.s006.docx]

BMDP7D - ONE- AND TWO-WAY ANALYSIS OF VARIANCE WITH DATA SCREENING

Copyright 1977, 1979, 1981, 1982, 1983, 1985, 1987, 1988, 1990, 1993

by BMDP Statistical Software, Inc.

Statistical Solutions Ltd. | Statistical Solutions

Unit 1A, South Ring Business Park | Stonehill Corporate Center, Suite 104

Kinsale Road, Cork, Ireland | 999 Broadway, Saugus, MA 01906, USA

Phone: + 353 21 4319629 | Phone: 781.231.7680

Fax: + 353 21 4319630 | Fax: 781.231.7684

e-mail: sales@statsol.ie | e-mail: info@statsolusa.com

Website: http://www.statsol.ie | Website: http://www.statsolusa.com

Release: 8.1 (Windows 9x, 2000, Me, Xp) Date: 05/30/17 at 15:13:18

Manual: BMDP Manual Volumes 1, 2, and 3.

Digest: BMDP User's Digest.

IBM PC: BMDP PC Supplement -- Installation and Special Features.

PROGRAM INSTRUCTIONS

/prob title = 'Herr Lauritz Englisch: D7D1.inp *** Quantitative Studien

zum Schmelzgehalt in Pferdebackenzaehnen.

- 1 Fall = 1 Zahn = 10 Lokalisationen = 10 Zeilen

- Hier: Unterkiefer, Zahn 7 - 10

- Umrechnung der relativen Werte in Prozentwerte

- Berechnung der Flächenanteile ohne aeusseren Zement

- mit Berechnung der Steigung "ST" ueber die Schnittebenen

- einfaktorielle Varianzanalyse

***'.

/inp var = 89.

file = d. ## analog zu Datensatz B.Dat

format = '16f10,8(/70x,9f10), /40x,1f10'. ## Mit Abstandsangabe für die Ebene 9

/var names = zahnid,pferd,rasse,zp,qu,znr,za,

for lo = 0 to 8.% ## Lokalisation

for va = lok,statu,ps,pz,id,ges,s_rel,z_rel,d_rel.%

|va||lo|,%%

abst99. ## Mit Abstandsangabe für die Ebene 99

use = pferd,zp,qu,znr,za,

for va = lok,ps,pz,id,ges,s_rel,z_rel,d_rel,dges.%

for lo = 0 to 8.% ## Lokalisation

|va||lo|,%%

for va = ps,id.%

for lo = 0 to 8.%

|va|_r2|lo|,%%

for va = ps,pz,id,ges,s_rel,z_rel,d_rel,dges.%

ST|va|,%

for va = ps,id.%

ST|va|r2,%

.

/group codes(pferd) = 7 to 10. ## diese Pferde haben mehr als 1 Zahn in der Au

/trans use = ((znr ge 7) AND (znr le 10)).

lok8 = abst99. ## Abstandsangabe für die Ebene 99

# Umrechnung der relativen Werte in Prozentwerte

for va = s_rel,z_rel,d_rel.%

for lo = 0 to 8.%

|va||lo| = |va||lo| * 100.%%

# Berechnung der Flächenanteile ohne aeusseren Zement

for va = ps,id.%

for lo = 0 to 8.%

|va|_r2|lo| = |va||lo|/(ges|lo|-pz|lo|) * 100.%%

# Berechnung der Gesamtflaeche abzüglich peripherem Zement

for lo = 0 to 8.%

dges|lo| = ges|lo|-pz|lo|.%

# Berechnung der Steigung "ST" ueber die Schnittebenen

for va = ps,pz,id,ges,s_rel,z_rel,d_rel,dges.%

ST|va| = b(for lo = 0 to 8.% lok|lo|,|va||lo|,%).

%

for va = ps,id.%

ST|va|r2 = b(for lo = 0 to 8.% lok|lo|,|va|_r2|lo|,%).

%

/hist group = Pferd.

var = for va = ps,pz,id,ges,s_rel,z_rel,d_rel,dges.%

ST|va|,%

for va = ps,id.%

ST|va|r2,%.

/print level = min.

case = 0.

/end

--- PROGRAM INSTRUCTIONS AFTER "FOR %" EXPANSION ---

/prob title = 'Herr Lauritz Englisch: D7D1.inp *** Quantitative Studien zum

Schmelzgehalt in Pferdebackenzaehnen.

- 1 Fall = 1 Zahn = 10 Lokalisationen =

10 Zeilen - Hier: Unterkiefer,

Zahn 7 - 10 - Umrechnung der relativen Werte in Prozentwerte -

Berechnung der Flächenanteile ohne aeusseren Zement - mit Berechnung

der Steigung "ST" ueber die Schnittebenen - einfaktorielle

Varianzanalyse ***'.

/inp var = 89. file = d. format = '16f10,8(/70x,9f10),

/40x,1f10'.

/var names = zahnid,pferd,rasse,zp,qu,znr,za, lok0, statu0, ps0, pz0, id0,

ges0, s_rel0, z_rel0, d_rel0, lok1, statu1, ps1, pz1, id1, ges1,

s_rel1, z_rel1, d_rel1, lok2, statu2, ps2, pz2, id2, ges2, s_rel2,

z_rel2, d_rel2, lok3, statu3, ps3, pz3, id3, ges3, s_rel3, z_rel3,

d_rel3, lok4, statu4, ps4, pz4, id4, ges4, s_rel4, z_rel4, d_rel4,

lok5, statu5, ps5, pz5, id5, ges5, s_rel5, z_rel5, d_rel5, lok6,

statu6, ps6, pz6, id6, ges6, s_rel6, z_rel6, d_rel6, lok7, statu7,

ps7, pz7, id7, ges7, s_rel7, z_rel7, d_rel7, lok8, statu8, ps8,

pz8, id8, ges8, s_rel8, z_rel8, d_rel8, abst99.

use = pferd,zp,qu,znr,za, lok0, lok1, lok2, lok3, lok4, lok5,

lok6, lok7, lok8, ps0, ps1, ps2, ps3, ps4, ps5, ps6,

ps7, ps8, pz0, pz1, pz2, pz3, pz4, pz5, pz6, pz7, pz8,

id0, id1, id2, id3, id4, id5, id6, id7, id8, ges0,

ges1, ges2, ges3, ges4, ges5, ges6, ges7, ges8, s_rel0,

s_rel1, s_rel2, s_rel3, s_rel4, s_rel5, s_rel6, s_rel7,

s_rel8, z_rel0, z_rel1, z_rel2, z_rel3, z_rel4, z_rel5,

z_rel6, z_rel7, z_rel8, d_rel0, d_rel1, d_rel2, d_rel3,

d_rel4, d_rel5, d_rel6, d_rel7, d_rel8, dges0, dges1,

dges2, dges3, dges4, dges5, dges6, dges7, dges8, ps_r20,

ps_r21, ps_r22, ps_r23, ps_r24, ps_r25, ps_r26, ps_r27, ps_r28,

id_r20, id_r21, id_r22, id_r23, id_r24, id_r25, id_r26, id_r27,

id_r28, STps, STpz, STid, STges, STs_rel, STz_rel, STd_rel, STdges,

STpsr2, STidr2 .

/group codes(pferd) = 7 to 10.

/trans use = ((znr ge 7) AND (znr le 10)). lok8 = abst99.

s_rel0 = s_rel0 * 100. s_rel1 = s_rel1 * 100. s_rel2 = s_rel2 * 100.

s_rel3 = s_rel3 * 100. s_rel4 = s_rel4 * 100. s_rel5 = s_rel5 * 100.

s_rel6 = s_rel6 * 100. s_rel7 = s_rel7 * 100. s_rel8 = s_rel8 * 100.

z_rel0 = z_rel0 * 100. z_rel1 = z_rel1 * 100. z_rel2 = z_rel2 * 100.

z_rel3 = z_rel3 * 100. z_rel4 = z_rel4 * 100. z_rel5 = z_rel5 * 100.

z_rel6 = z_rel6 * 100. z_rel7 = z_rel7 * 100. z_rel8 = z_rel8 * 100.

d_rel0 = d_rel0 * 100. d_rel1 = d_rel1 * 100. d_rel2 = d_rel2 * 100.

d_rel3 = d_rel3 * 100. d_rel4 = d_rel4 * 100. d_rel5 = d_rel5 * 100.

d_rel6 = d_rel6 * 100. d_rel7 = d_rel7 * 100. d_rel8 = d_rel8 * 100.

ps_r20 = ps0/(ges0-pz0) * 100. ps_r21 = ps1/(ges1-pz1) * 100.

ps_r22 = ps2/(ges2-pz2) * 100. ps_r23 = ps3/(ges3-pz3) * 100.

ps_r24 = ps4/(ges4-pz4) * 100. ps_r25 = ps5/(ges5-pz5) * 100.

ps_r26 = ps6/(ges6-pz6) * 100. ps_r27 = ps7/(ges7-pz7) * 100.

ps_r28 = ps8/(ges8-pz8) * 100. id_r20 = id0/(ges0-pz0) * 100.

id_r21 = id1/(ges1-pz1) * 100. id_r22 = id2/(ges2-pz2) * 100.

id_r23 = id3/(ges3-pz3) * 100. id_r24 = id4/(ges4-pz4) * 100.

id_r25 = id5/(ges5-pz5) * 100. id_r26 = id6/(ges6-pz6) * 100.

id_r27 = id7/(ges7-pz7) * 100. id_r28 = id8/(ges8-pz8) * 100.

dges0 = ges0-pz0. dges1 = ges1-pz1. dges2 = ges2-pz2. dges3 = ges3-pz3.

dges4 = ges4-pz4. dges5 = ges5-pz5. dges6 = ges6-pz6. dges7 = ges7-pz7.

dges8 = ges8-pz8. STps =

b( lok0,ps0, lok1,ps1, lok2,ps2, lok3,ps3, lok4,ps4, lok5,ps5, lok6,

ps6, lok7,ps7, lok8,ps8).

STpz = b( lok0,pz0, lok1,pz1, lok2,pz2, lok3,pz3, lok4,pz4, lok5,pz5,

lok6,pz6, lok7,pz7, lok8,pz8).

STid = b( lok0,id0, lok1,id1, lok2,id2, lok3,id3, lok4,id4, lok5,id5,

lok6,id6, lok7,id7, lok8,id8).

STges = b( lok0,ges0, lok1,ges1, lok2,ges2, lok3,ges3, lok4,ges4, lok5,

ges5, lok6,ges6, lok7,ges7, lok8,ges8).

STs_rel = b( lok0,s_rel0, lok1,s_rel1, lok2,s_rel2, lok3,s_rel3, lok4,

s_rel4, lok5,s_rel5, lok6,s_rel6, lok7,s_rel7, lok8,s_rel8).

STz_rel = b( lok0,z_rel0, lok1,z_rel1, lok2,z_rel2, lok3,z_rel3, lok4,

z_rel4, lok5,z_rel5, lok6,z_rel6, lok7,z_rel7, lok8,z_rel8).

STd_rel = b( lok0,d_rel0, lok1,d_rel1, lok2,d_rel2, lok3,d_rel3, lok4,

d_rel4, lok5,d_rel5, lok6,d_rel6, lok7,d_rel7, lok8,d_rel8).

STdges = b( lok0,dges0, lok1,dges1, lok2,dges2, lok3,dges3, lok4,dges4,

lok5,dges5, lok6,dges6, lok7,dges7, lok8,dges8).

STpsr2 = b( lok0,ps_r20, lok1,ps_r21, lok2,ps_r22, lok3,ps_r23, lok4,

ps_r24, lok5,ps_r25, lok6,ps_r26, lok7,ps_r27, lok8,ps_r28).

STidr2 = b( lok0,id_r20, lok1,id_r21, lok2,id_r22, lok3,id_r23, lok4,

id_r24, lok5,id_r25, lok6,id_r26, lok7,id_r27, lok8,id_r28).

/hist group = Pferd. var = STps, STpz, STid, STges, STs_rel, STz_rel, STd_rel,

STdges, STpsr2, STidr2.

/print level = min. case = 0.

/end/

NUMBER OF CASES READ. . . . . . . . . . . . . . 26

CASES WITH USE SET TO ZERO . . . . . . . . . 10

REMAINING NUMBER OF CASES . . . . . . . . 16

DESCRIPTIVE STATISTICS OF DATA

----------- ---------- -- ----

VARIABLE TOTAL STANDARD ST.ERR COEFF SMALLEST LARGEST

NO. NAME FREQ. MEAN DEV. OF MEAN OF VAR VALUE VALUE RANGE

2 pferd 16 7.0625 2.9090 .72726 .41190 1.0000 10.000 9.0000

4 zp 16 376.75 47.885 11.971 .12710 307.00 410.00 103.00

5 qu 16 3.6875 .47871 .11968 .12982 3.0000 4.0000 1.0000

6 znr 16 8.0000 1.1547 .28868 .14434 7.0000 10.000 3.0000

7 za 16 9.9375 5.0986 1.2747 .51307 1.5000 16.000 14.500

8 lok0 16 0.0000 0.0000 0.0000 0.0000 0.0000 0.0000

17 lok1 16 10.000 0.0000 0.0000 0.0000 10.000 10.000 0.0000

26 lok2 16 20.000 0.0000 0.0000 0.0000 20.000 20.000 0.0000

35 lok3 16 30.000 0.0000 0.0000 0.0000 30.000 30.000 0.0000

44 lok4 16 40.000 0.0000 0.0000 0.0000 40.000 40.000 0.0000

53 lok5 16 50.000 0.0000 0.0000 0.0000 50.000 50.000 0.0000

62 lok6 16 60.000 0.0000 0.0000 0.0000 60.000 60.000 0.0000

71 lok7 16 70.000 0.0000 0.0000 0.0000 70.000 70.000 0.0000

80 lok8 16 41.292 19.043 4.7607 .46118 21.570 75.770 54.200

10 ps0 16 89.754 19.127 4.7819 .21311 62.530 134.11 71.580

19 ps1 16 120.94 20.451 5.1127 .16910 78.390 161.60 83.210

28 ps2 16 123.72 22.112 5.5280 .17872 82.190 156.03 73.840

37 ps3 9 127.59 16.927 5.6424 .13267 104.87 154.31 49.440

46 ps4 6 128.68 16.916 6.9061 .13146 104.75 146.52 41.770

55 ps5 5 124.15 12.314 5.5069 .09918 105.90 139.93 34.030

64 ps6 4 128.48 4.2764 2.1382 .03329 124.47 133.20 8.7300

73 ps7 1 132.77 0.0000 0.0000 0.0000 132.77 132.77 0.0000

82 ps8 16 117.98 18.737 4.6842 .15881 81.150 144.73 63.580

11 pz0 16 50.933 35.626 8.9066 .69947 16.270 123.60 107.33

20 pz1 16 109.45 64.351 16.088 .58795 32.440 208.98 176.54

29 pz2 16 159.88 83.986 20.996 .52530 40.650 292.54 251.89

38 pz3 9 142.12 84.246 28.082 .59277 51.940 279.64 227.70

47 pz4 5 96.750 35.789 16.006 .36992 55.760 137.65 81.890

56 pz5 5 162.12 89.864 40.188 .55431 65.850 267.20 201.35

65 pz6 4 208.12 27.576 13.788 .13250 187.00 247.63 60.630

74 pz7 1 253.18 0.0000 0.0000 0.0000 253.18 253.18 0.0000

83 pz8 16 165.91 42.773 10.693 .25781 92.850 257.61 164.76

12 id0 16 192.71 32.884 8.2211 .17064 108.33 232.01 123.68

21 id1 16 169.97 30.189 7.5473 .17761 104.42 229.04 124.62

30 id2 16 170.20 28.516 7.1290 .16755 102.30 218.55 116.25

39 id3 9 193.51 24.499 8.1665 .12661 156.56 222.95 66.390

48 id4 6 183.51 27.205 11.106 .14824 152.21 216.25 64.040

57 id5 5 189.13 31.456 14.067 .16631 150.48 219.37 68.890

66 id6 4 178.41 32.670 16.335 .18312 147.75 207.04 59.290

75 id7 1 193.95 0.0000 0.0000 0.0000 193.95 193.95 0.0000

84 id8 16 172.95 32.968 8.2419 .19062 102.53 225.12 122.59

13 ges0 16 333.40 61.417 15.354 .18422 209.51 461.13 251.62

22 ges1 16 400.36 82.318 20.579 .20561 271.44 519.47 248.03

31 ges2 16 453.80 97.804 24.451 .21552 311.87 605.64 293.77

40 ges3 9 463.21 105.96 35.321 .22876 332.43 650.18 317.75

49 ges4 5 417.42 66.830 29.887 .16010 345.03 493.87 148.84

58 ges5 5 475.41 123.61 55.278 .26000 337.48 626.51 289.03

67 ges6 4 515.00 35.492 17.746 .06892 464.06 545.60 81.540

76 ges7 1 579.90 0.0000 0.0000 0.0000 579.90 579.90 0.0000

85 ges8 16 456.83 82.501 20.625 .18059 281.42 569.58 288.16

14 s_rel0 16 27.038 3.4510 .86274 .12764 21.600 35.500 13.900

23 s_rel1 16 30.725 4.6540 1.1635 .15147 23.500 38.700 15.200

32 s_rel2 16 27.969 5.8900 1.4725 .21059 21.900 37.700 15.800

41 s_rel3 9 28.344 5.0683 1.6894 .17881 22.200 34.800 12.600

50 s_rel4 5 32.000 4.9031 2.1927 .15322 27.300 39.700 12.400

59 s_rel5 5 27.160 5.6487 2.5262 .20798 22.000 35.900 13.900

68 s_rel6 4 25.000 1.2247 .61237 .04899 24.100 26.800 2.7000

77 s_rel7 1 22.900 0.0000 0.0000 0.0000 22.900 22.900 0.0000

86 s_rel8 16 26.131 3.3420 .83550 .12789 21.100 35.000 13.900

15 z_rel0 16 14.681 8.4979 2.1245 .57883 4.9000 27.000 22.100

24 z_rel1 16 25.912 11.793 2.9481 .45509 10.600 42.300 31.700

33 z_rel2 16 33.500 13.764 3.4410 .41086 12.600 49.900 37.300

42 z_rel3 9 28.722 11.480 3.8266 .39969 15.600 43.000 27.400

51 z_rel4 5 22.640 5.3984 2.4142 .23845 16.200 29.200 13.000

60 z_rel5 5 32.080 10.560 4.7224 .32917 19.500 43.000 23.500

69 z_rel6 4 40.475 5.0408 2.5204 .12454 36.200 47.600 11.400

78 z_rel7 1 43.700 0.0000 0.0000 0.0000 43.700 43.700 0.0000

87 z_rel8 16 35.956 4.2417 1.0604 .11797 26.600 46.800 20.200

16 d_rel0 16 58.275 7.5841 1.8960 .13014 44.100 70.300 26.200

25 d_rel1 16 43.369 8.0354 2.0088 .18528 32.100 53.600 21.500

34 d_rel2 16 38.531 8.0440 2.0110 .20877 27.200 50.900 23.700

43 d_rel3 9 42.900 6.8218 2.2739 .15902 34.300 50.700 16.400

52 d_rel4 5 45.340 2.1686 .96985 .04783 43.400 47.700 4.3000

61 d_rel5 5 40.740 5.5891 2.4995 .13719 35.000 47.300 12.300

70 d_rel6 4 34.550 4.8925 2.4463 .14161 28.400 39.100 10.700

79 d_rel7 1 33.400 0.0000 0.0000 0.0000 33.400 33.400 0.0000

88 d_rel8 16 37.925 3.3670 .84175 .08878 32.100 45.800 13.700

108 dges0 16 282.46 45.841 11.460 .16229 170.86 337.53 166.67

109 dges1 16 290.91 45.745 11.436 .15725 182.80 361.64 178.84

110 dges2 16 293.92 49.666 12.416 .16898 184.49 374.58 190.09

111 dges3 9 321.09 39.081 13.027 .12171 267.09 370.54 103.45

112 dges4 5 320.67 37.651 16.838 .11741 273.83 362.77 88.940

113 dges5 5 313.30 41.784 18.686 .13337 266.62 359.31 92.690

114 dges6 4 306.89 36.813 18.407 .11996 273.02 339.45 66.430

115 dges7 1 326.72 0.0000 0.0000 0.0000 326.72 326.72 0.0000

116 dges8 16 290.93 46.895 11.724 .16119 188.57 360.36 171.79

90 ps_r20 16 31.819 3.8969 .97422 .12247 25.378 39.733 14.355

91 ps_r21 16 41.663 3.3050 .82625 .07933 32.431 46.323 13.893

92 ps_r22 16 42.066 1.7951 .44878 .04267 39.175 45.722 6.5471

93 ps_r23 9 39.743 2.0723 .69077 .05214 36.751 42.341 5.5891

94 ps_r24 5 41.221 3.6860 1.6484 .08942 38.254 47.381 9.1277

95 ps_r25 5 39.833 2.8098 1.2566 .07054 37.243 44.597 7.3542

96 ps_r26 4 42.199 3.7271 1.8636 .08832 38.747 45.883 7.1357

97 ps_r27 1 40.637 0.0000 0.0000 0.0000 40.637 40.637 0.0000

98 ps_r28 16 40.740 3.9343 .98358 .09657 37.127 51.268 14.141

99 id_r20 16 68.181 3.8975 .97438 .05716 60.267 74.626 14.358

100 id_r21 16 58.337 3.3050 .82625 .05665 53.677 67.569 13.893

101 id_r22 16 57.934 1.7950 .44875 .03098 54.274 60.825 6.5505

102 id_r23 9 60.257 2.0728 .69094 .03440 57.659 63.249 5.5891

103 id_r24 5 58.778 3.6853 1.6481 .06270 52.619 61.743 9.1240

104 id_r25 5 60.164 2.8101 1.2567 .04671 55.399 62.754 7.3550

105 id_r26 4 57.802 3.7279 1.8640 .06450 54.117 61.253 7.1357

106 id_r27 1 59.363 0.0000 0.0000 0.0000 59.363 59.363 0.0000

107 id_r28 16 59.261 3.9346 .98366 .06640 48.728 62.870 14.142

117 STps 16 .68289 .52635 .13159 .77077 -.11643 1.7245 1.8409

118 STpz 16 3.7048 1.4794 .36985 .39932 1.4930 7.5728 6.0798

119 STid 16 -.38397 .60354 .15089 -1.5718 -2.1609 .45713 2.6180

120 STges 16 3.9974 1.7050 .42625 .42652 2.1687 8.2274 6.0587

121 STs_rel 16 -.07953 .11042 .02760 -1.3884 -.23804 .24584 .48388

122 STz_rel 16 .61927 .21218 .05305 .34264 .17742 1.0073 .82991

123 STd_rel 16 -.53972 .21815 .05454 -.40419 -.87326 -.16331 .70996

124 STdges 16 .29265 .50220 .12555 1.7160 -.43653 1.3783 1.8148

125 STpsr2 16 .20630 .16401 .04100 .79501 -.03888 .59045 .62933

126 STidr2 16 -.20628 .16400 .04100 -.79505 -.59042 .03885 .62927

************ ************

HISTOGRAM OF * STps * ( 117) GROUPED BY * pferd * ( 2)

************ ************

CASES WITH

UNUSED

VALUES FOR

*7 *8 *9 *10 pferd

MIDPOINTS.......................+.......................+.......................+.......................+.......................+

1.80000) *

1.62000)*

1.44000)

1.26000) M*

1.08000)N

0.90000) *

0.72000) * N M

0.54000)* ** *

0.36000) * *

0.18000) M

0.00000) *

-0.18000) *

LEGEND FOR GROUP MEANS: M - MEAN COINCIDES WITH AN ASTERISK

N - MEAN DOES NOT COINCIDE WITH ANY ASTERISK

MEAN 1.036 1.248 0.250 0.643 0.686

STD.DEV. 0.742 0.031 0.310 0.151 0.638

S. E. M. 0.525 0.022 0.155 0.087 0.285

MAXIMUM 1.560 1.270 0.632 0.811 1.724

MINIMUM 0.511 1.226 -0.116 0.517 0.068

CASES EXCL. ( 0) ( 0) ( 0) ( 0) ( 0)

CASES INCL. 2 2 4 3 5

---------------------------------------------------------------------------- ALL GROUPS COMBINED

| ANALYSIS OF VARIANCE TABLE FOR MEANS TAIL | (EXCEPT CASES WITH UNUSED

| SOURCE SUM OF SQUARES DF MEAN SQUARE F VALUE PROBABILITY | VALUES FOR VARIABLE pferd )

| -------- -------------- ---- ----------- ------- ----------- | MEAN 0.682

| pferd 1.6397 3 0.5466 4.32 0.0508 | STD. DEV. 0.503

| ERROR 0.8863 7 0.1266 | S. E. M. 0.152

|--------------------------------------------------------------------------| MAXIMUM 1.560

| EQUALITY OF MEANS TESTS; VARIANCES ARE NOT ASSUMED TO BE EQUAL | MINIMUM -0.116

| WELCH 3, 3 18.79 0.0190 | CASES EXCLUDED ( 5)

| BROWN-FORSYTHE 3, 1 3.10 0.3904 | CASES INCLUDED 11

|--------------------------------------------------------------------------|

| LEVENE'S TEST FOR VARIANCES 3, 7 6.70 0.0182 | ROBUST S.D. 0.511

----------------------------------------------------------------------------

************ ************

HISTOGRAM OF * STpz * ( 118) GROUPED BY * pferd * ( 2)

************ ************

CASES WITH

UNUSED

VALUES FOR

*7 *8 *9 *10 pferd

MIDPOINTS.......................+.......................+.......................+.......................+.......................+

7.80000) *

7.20000)

6.60000) N

6.00000)

5.40000)* *

4.80000)N *

4.20000)* *

3.60000) * M

3.00000) M* M ***

2.40000)

1.80000) *

1.20000) *

LEGEND FOR GROUP MEANS: M - MEAN COINCIDES WITH AN ASTERISK

N - MEAN DOES NOT COINCIDE WITH ANY ASTERISK

MEAN 4.737 6.361 2.704 2.809 3.568

STD.DEV. 0.627 1.714 0.555 1.323 0.763

S. E. M. 0.444 1.212 0.278 0.764 0.341

MAXIMUM 5.180 7.573 3.321 4.138 4.889

MINIMUM 4.293 5.149 1.971 1.493 2.951

CASES EXCL. ( 0) ( 0) ( 0) ( 0) ( 0)

CASES INCL. 2 2 4 3 5

---------------------------------------------------------------------------- ALL GROUPS COMBINED

| ANALYSIS OF VARIANCE TABLE FOR MEANS TAIL | (EXCEPT CASES WITH UNUSED

| SOURCE SUM OF SQUARES DF MEAN SQUARE F VALUE PROBABILITY | VALUES FOR VARIABLE pferd )

| -------- -------------- ---- ----------- ------- ----------- | MEAN 3.767

| pferd 22.6106 3 7.5369 6.80 0.0175 | STD. DEV. 1.743

| ERROR 7.7543 7 1.1078 | S. E. M. 0.525

|--------------------------------------------------------------------------| MAXIMUM 7.573

| EQUALITY OF MEANS TESTS; VARIANCES ARE NOT ASSUMED TO BE EQUAL | MINIMUM 1.493

| WELCH 3, 3 4.78 0.1155 | CASES EXCLUDED ( 5)

| BROWN-FORSYTHE 3, 3 5.39 0.1000 | CASES INCLUDED 11

|--------------------------------------------------------------------------|

| LEVENE'S TEST FOR VARIANCES 3, 7 1.83 0.2301 | ROBUST S.D. 1.792

----------------------------------------------------------------------------

************ ************

HISTOGRAM OF * STid * ( 119) GROUPED BY * pferd * ( 2)

************ ************

CASES WITH

UNUSED

VALUES FOR

*7 *8 *9 *10 pferd

MIDPOINTS.......................+.......................+.......................+.......................+.......................+

0.50000) * *

0.25000)

0.00000) * N *

-0.25000)M M *

-0.50000)* M* * *

-0.75000) * M*

-1.00000)

-1.25000)

-1.50000)

-1.75000)

-2.00000)

-2.25000) *

LEGEND FOR GROUP MEANS: M - MEAN COINCIDES WITH AN ASTERISK

N - MEAN DOES NOT COINCIDE WITH ANY ASTERISK

MEAN -0.297 -0.528 -0.291 -0.062 -0.629

STD.DEV. 0.162 0.123 0.344 0.446 0.996

S. E. M. 0.114 0.087 0.172 0.258 0.445

MAXIMUM -0.182 -0.441 0.116 0.441 0.457

MINIMUM -0.411 -0.615 -0.681 -0.411 -2.161

CASES EXCL. ( 0) ( 0) ( 0) ( 0) ( 0)

CASES INCL. 2 2 4 3 5

---------------------------------------------------------------------------- ALL GROUPS COMBINED

| ANALYSIS OF VARIANCE TABLE FOR MEANS TAIL | (EXCEPT CASES WITH UNUSED

| SOURCE SUM OF SQUARES DF MEAN SQUARE F VALUE PROBABILITY | VALUES FOR VARIABLE pferd )

| -------- -------------- ---- ----------- ------- ----------- | MEAN -0.273

| pferd 0.2663 3 0.0888 0.78 0.5405 | STD. DEV. 0.326

| ERROR 0.7945 7 0.1135 | S. E. M. 0.098

|--------------------------------------------------------------------------| MAXIMUM 0.441

| EQUALITY OF MEANS TESTS; VARIANCES ARE NOT ASSUMED TO BE EQUAL | MINIMUM -0.681

| WELCH 3, 4 1.23 0.4078 | CASES EXCLUDED ( 5)

| BROWN-FORSYTHE 3, 5 1.05 0.4477 | CASES INCLUDED 11

|--------------------------------------------------------------------------|

| LEVENE'S TEST FOR VARIANCES 3, 7 1.88 0.2205 | ROBUST S.D. 0.325

----------------------------------------------------------------------------

************ ************

HISTOGRAM OF * STges * ( 120) GROUPED BY * pferd * ( 2)

************ ************

CASES WITH

UNUSED

VALUES FOR

*7 *8 *9 *10 pferd

MIDPOINTS.......................+.......................+.......................+.......................+.......................+

9.00000)

8.40000) *

7.80000)

7.20000) N

6.60000)*

6.00000) *

5.40000)N

4.80000) *

4.20000)* *

3.60000) * M M*

3.00000) * *

2.40000) M* * *

LEGEND FOR GROUP MEANS: M - MEAN COINCIDES WITH AN ASTERISK

N - MEAN DOES NOT COINCIDE WITH ANY ASTERISK

MEAN 5.475 7.080 2.663 3.390 3.605

STD.DEV. 1.532 1.622 0.505 0.940 0.965

S. E. M. 1.083 1.147 0.252 0.543 0.432

MAXIMUM 6.558 8.227 3.321 4.330 5.016

MINIMUM 4.392 5.933 2.169 2.451 2.515

CASES EXCL. ( 0) ( 0) ( 0) ( 0) ( 0)

CASES INCL. 2 2 4 3 5

---------------------------------------------------------------------------- ALL GROUPS COMBINED

| ANALYSIS OF VARIANCE TABLE FOR MEANS TAIL | (EXCEPT CASES WITH UNUSED

| SOURCE SUM OF SQUARES DF MEAN SQUARE F VALUE PROBABILITY | VALUES FOR VARIABLE pferd )

| -------- -------------- ---- ----------- ------- ----------- | MEAN 4.176

| pferd 31.2521 3 10.4174 9.71 0.0069 | STD. DEV. 1.969

| ERROR 7.5076 7 1.0725 | S. E. M. 0.594

|--------------------------------------------------------------------------| MAXIMUM 8.227

| EQUALITY OF MEANS TESTS; VARIANCES ARE NOT ASSUMED TO BE EQUAL | MINIMUM 2.169

| WELCH 3, 2 4.21 0.1978 | CASES EXCLUDED ( 5)

| BROWN-FORSYTHE 3, 3 6.41 0.0807 | CASES INCLUDED 11

|--------------------------------------------------------------------------|

| LEVENE'S TEST FOR VARIANCES 3, 7 3.38 0.0835 | ROBUST S.D. 2.046

----------------------------------------------------------------------------

************ ************

HISTOGRAM OF * STs_rel * ( 121) GROUPED BY * pferd * ( 2)

************ ************

CASES WITH

UNUSED

VALUES FOR

*7 *8 *9 *10 pferd

MIDPOINTS.......................+.......................+.......................+.......................+.......................+

0.300000)

0.250000) *

0.200000)

0.150000)

0.100000)

0.050000)

0.000000)* * *

-.050000) * M M

-.100000)N N M *

-.150000)* * * * *

-.200000) *

-.250000) *

LEGEND FOR GROUP MEANS: M - MEAN COINCIDES WITH AN ASTERISK

N - MEAN DOES NOT COINCIDE WITH ANY ASTERISK

MEAN -0.077 -0.084 -0.121 -0.070 -0.051

STD.DEV. 0.114 0.069 0.094 0.069 0.172

S. E. M. 0.081 0.049 0.047 0.040 0.077

MAXIMUM 0.004 -0.035 -0.012 -0.014 0.246

MINIMUM -0.157 -0.133 -0.238 -0.146 -0.176

CASES EXCL. ( 0) ( 0) ( 0) ( 0) ( 0)

CASES INCL. 2 2 4 3 5

---------------------------------------------------------------------------- ALL GROUPS COMBINED

| ANALYSIS OF VARIANCE TABLE FOR MEANS TAIL | (EXCEPT CASES WITH UNUSED

| SOURCE SUM OF SQUARES DF MEAN SQUARE F VALUE PROBABILITY | VALUES FOR VARIABLE pferd )

| -------- -------------- ---- ----------- ------- ----------- | MEAN -0.092

| pferd 0.0055 3 0.0018 0.24 0.8660 | STD. DEV. 0.077

| ERROR 0.0535 7 0.0076 | S. E. M. 0.023

|--------------------------------------------------------------------------| MAXIMUM 0.004

| EQUALITY OF MEANS TESTS; VARIANCES ARE NOT ASSUMED TO BE EQUAL | MINIMUM -0.238

| WELCH 3, 3 0.17 0.9110 | CASES EXCLUDED ( 5)

| BROWN-FORSYTHE 3, 4 0.23 0.8692 | CASES INCLUDED 11

|--------------------------------------------------------------------------|

| LEVENE'S TEST FOR VARIANCES 3, 7 0.33 0.8029 | ROBUST S.D. 0.085

----------------------------------------------------------------------------

************ ************

HISTOGRAM OF * STz_rel * ( 122) GROUPED BY * pferd * ( 2)

************ ************

CASES WITH

UNUSED

VALUES FOR

*7 *8 *9 *10 pferd

MIDPOINTS.......................+.......................+.......................+.......................+.......................+

1.04000) *

0.96000) *

0.88000) N

0.80000)*

0.72000)M * **

0.64000) *

0.56000) ** N M

0.48000) M *

0.40000) * *

0.32000)

0.24000)

0.16000) *

LEGEND FOR GROUP MEANS: M - MEAN COINCIDES WITH AN ASTERISK

N - MEAN DOES NOT COINCIDE WITH ANY ASTERISK

MEAN 0.757 0.872 0.496 0.597 0.575

STD.DEV. 0.036 0.191 0.065 0.387 0.149

S. E. M. 0.025 0.135 0.033 0.223 0.066

MAXIMUM 0.783 1.007 0.554 0.939 0.725

MINIMUM 0.732 0.737 0.414 0.177 0.369

CASES EXCL. ( 0) ( 0) ( 0) ( 0) ( 0)

CASES INCL. 2 2 4 3 5

---------------------------------------------------------------------------- ALL GROUPS COMBINED

| ANALYSIS OF VARIANCE TABLE FOR MEANS TAIL | (EXCEPT CASES WITH UNUSED

| SOURCE SUM OF SQUARES DF MEAN SQUARE F VALUE PROBABILITY | VALUES FOR VARIABLE pferd )

| -------- -------------- ---- ----------- ------- ----------- | MEAN 0.640

| pferd 0.2230 3 0.0743 1.49 0.2985 | STD. DEV. 0.239

| ERROR 0.3495 7 0.0499 | S. E. M. 0.072

|--------------------------------------------------------------------------| MAXIMUM 1.007

| EQUALITY OF MEANS TESTS; VARIANCES ARE NOT ASSUMED TO BE EQUAL | MINIMUM 0.177

| WELCH 3, 3 9.58 0.0479 | CASES EXCLUDED ( 5)

| BROWN-FORSYTHE 3, 3 1.57 0.3608 | CASES INCLUDED 11

|--------------------------------------------------------------------------|

| LEVENE'S TEST FOR VARIANCES 3, 7 3.99 0.0599 | ROBUST S.D. 0.247

----------------------------------------------------------------------------

************ ************

HISTOGRAM OF * STd_rel * ( 123) GROUPED BY * pferd * ( 2)

************ ************

CASES WITH

UNUSED

VALUES FOR

*7 *8 *9 *10 pferd

MIDPOINTS.......................+.......................+.......................+.......................+.......................+

-.070000)

-.140000) *

-.210000) *

-.280000) *

-.350000) M

-.420000) ** *

-.490000) N

-.560000) N

-.630000)* * **

-.700000)N *

-.770000)* N * *

-.840000) *

LEGEND FOR GROUP MEANS: M - MEAN COINCIDES WITH AN ASTERISK

N - MEAN DOES NOT COINCIDE WITH ANY ASTERISK

MEAN -0.682 -0.787 -0.374 -0.527 -0.524

STD.DEV. 0.079 0.122 0.067 0.326 0.220

S. E. M. 0.056 0.086 0.033 0.188 0.098

MAXIMUM -0.626 -0.701 -0.305 -0.163 -0.192

MINIMUM -0.737 -0.873 -0.455 -0.794 -0.742

CASES EXCL. ( 0) ( 0) ( 0) ( 0) ( 0)

CASES INCL. 2 2 4 3 5

---------------------------------------------------------------------------- ALL GROUPS COMBINED

| ANALYSIS OF VARIANCE TABLE FOR MEANS TAIL | (EXCEPT CASES WITH UNUSED

| SOURCE SUM OF SQUARES DF MEAN SQUARE F VALUE PROBABILITY | VALUES FOR VARIABLE pferd )

| -------- -------------- ---- ----------- ------- ----------- | MEAN -0.547

| pferd 0.2719 3 0.0906 2.57 0.1373 | STD. DEV. 0.228

| ERROR 0.2472 7 0.0353 | S. E. M. 0.069

|--------------------------------------------------------------------------| MAXIMUM -0.163

| EQUALITY OF MEANS TESTS; VARIANCES ARE NOT ASSUMED TO BE EQUAL | MINIMUM -0.873

| WELCH 3, 3 7.90 0.0618 | CASES EXCLUDED ( 5)

| BROWN-FORSYTHE 3, 3 2.79 0.2108 | CASES INCLUDED 11

|--------------------------------------------------------------------------|

| LEVENE'S TEST FOR VARIANCES 3, 7 4.38 0.0491 | ROBUST S.D. 0.257

----------------------------------------------------------------------------

************ ************

HISTOGRAM OF * STdges * ( 124) GROUPED BY * pferd * ( 2)

************ ************

CASES WITH

UNUSED

VALUES FOR

*7 *8 *9 *10 pferd

MIDPOINTS.......................+.......................+.......................+.......................+.......................+

1.62000)

1.44000)*

1.26000)

1.08000)

0.90000) *

0.72000)N M*

0.54000) M *

0.36000) *

0.18000)* * * *

0.00000) M* N

-0.18000)

-0.36000) * **

LEGEND FOR GROUP MEANS: M - MEAN COINCIDES WITH AN ASTERISK

N - MEAN DOES NOT COINCIDE WITH ANY ASTERISK

MEAN 0.739 0.719 -0.041 0.581 0.037

STD.DEV. 0.904 0.092 0.239 0.383 0.421

S. E. M. 0.639 0.065 0.120 0.221 0.188

MAXIMUM 1.378 0.784 0.197 0.958 0.525

MINIMUM 0.099 0.655 -0.373 0.192 -0.437

CASES EXCL. ( 0) ( 0) ( 0) ( 0) ( 0)

CASES INCL. 2 2 4 3 5

---------------------------------------------------------------------------- ALL GROUPS COMBINED

| ANALYSIS OF VARIANCE TABLE FOR MEANS TAIL | (EXCEPT CASES WITH UNUSED

| SOURCE SUM OF SQUARES DF MEAN SQUARE F VALUE PROBABILITY | VALUES FOR VARIABLE pferd )

| -------- -------------- ---- ----------- ------- ----------- | MEAN 0.409

| pferd 1.3086 3 0.4362 2.36 0.1571 | STD. DEV. 0.510

| ERROR 1.2914 7 0.1845 | S. E. M. 0.154

|--------------------------------------------------------------------------| MAXIMUM 1.378

| EQUALITY OF MEANS TESTS; VARIANCES ARE NOT ASSUMED TO BE EQUAL | MINIMUM -0.373

| WELCH 3, 3 7.18 0.0699 | CASES EXCLUDED ( 5)

| BROWN-FORSYTHE 3, 1 1.60 0.5134 | CASES INCLUDED 11

|--------------------------------------------------------------------------|

| LEVENE'S TEST FOR VARIANCES 3, 7 6.12 0.0228 | ROBUST S.D. 0.556

----------------------------------------------------------------------------

************ ************

HISTOGRAM OF * STpsr2 * ( 125) GROUPED BY * pferd * ( 2)

************ ************

CASES WITH

UNUSED

VALUES FOR

*7 *8 *9 *10 pferd

MIDPOINTS.......................+.......................+.......................+.......................+.......................+

0.600000) *

0.540000)

0.480000)

0.420000)

0.360000)* M

0.300000) * *

0.240000)N M M*

0.180000)* **

0.120000) N

0.060000) * * *

0.000000)

-.060000) * *

LEGEND FOR GROUP MEANS: M - MEAN COINCIDES WITH AN ASTERISK

N - MEAN DOES NOT COINCIDE WITH ANY ASTERISK

MEAN 0.250 0.343 0.097 0.214 0.217

STD.DEV. 0.119 0.026 0.106 0.131 0.240

S. E. M. 0.084 0.018 0.053 0.076 0.107

MAXIMUM 0.334 0.362 0.202 0.315 0.590

MINIMUM 0.165 0.325 -0.033 0.066 -0.039

CASES EXCL. ( 0) ( 0) ( 0) ( 0) ( 0)

CASES INCL. 2 2 4 3 5

---------------------------------------------------------------------------- ALL GROUPS COMBINED

| ANALYSIS OF VARIANCE TABLE FOR MEANS TAIL | (EXCEPT CASES WITH UNUSED

| SOURCE SUM OF SQUARES DF MEAN SQUARE F VALUE PROBABILITY | VALUES FOR VARIABLE pferd )

| -------- -------------- ---- ----------- ------- ----------- | MEAN 0.202

| pferd 0.0889 3 0.0296 2.49 0.1441 | STD. DEV. 0.131

| ERROR 0.0832 7 0.0119 | S. E. M. 0.040

|--------------------------------------------------------------------------| MAXIMUM 0.362

| EQUALITY OF MEANS TESTS; VARIANCES ARE NOT ASSUMED TO BE EQUAL | MINIMUM -0.033

| WELCH 3, 3 4.92 0.1116 | CASES EXCLUDED ( 5)

| BROWN-FORSYTHE 3, 4 2.79 0.1737 | CASES INCLUDED 11

|--------------------------------------------------------------------------|

| LEVENE'S TEST FOR VARIANCES 3, 7 2.10 0.1887 | ROBUST S.D. 0.141

----------------------------------------------------------------------------

************ ************

HISTOGRAM OF * STidr2 * ( 126) GROUPED BY * pferd * ( 2)

************ ************

CASES WITH

UNUSED

VALUES FOR

*7 *8 *9 *10 pferd

MIDPOINTS.......................+.......................+.......................+.......................+.......................+

0.060000) * *

0.000000)

-.060000) * * *

-.120000) N

-.180000)* **

-.240000)N M M*

-.300000) * *

-.360000)* M

-.420000)

-.480000)

-.540000)

-.600000) *

LEGEND FOR GROUP MEANS: M - MEAN COINCIDES WITH AN ASTERISK

N - MEAN DOES NOT COINCIDE WITH ANY ASTERISK

MEAN -0.250 -0.343 -0.097 -0.214 -0.217

STD.DEV. 0.119 0.026 0.106 0.131 0.240

S. E. M. 0.084 0.019 0.053 0.076 0.107

MAXIMUM -0.165 -0.325 0.033 -0.066 0.039

MINIMUM -0.334 -0.362 -0.202 -0.314 -0.590

CASES EXCL. ( 0) ( 0) ( 0) ( 0) ( 0)

CASES INCL. 2 2 4 3 5

---------------------------------------------------------------------------- ALL GROUPS COMBINED

| ANALYSIS OF VARIANCE TABLE FOR MEANS TAIL | (EXCEPT CASES WITH UNUSED

| SOURCE SUM OF SQUARES DF MEAN SQUARE F VALUE PROBABILITY | VALUES FOR VARIABLE pferd )

| -------- -------------- ---- ----------- ------- ----------- | MEAN -0.202

| pferd 0.0889 3 0.0296 2.49 0.1445 | STD. DEV. 0.131

| ERROR 0.0833 7 0.0119 | S. E. M. 0.040

|--------------------------------------------------------------------------| MAXIMUM 0.033

| EQUALITY OF MEANS TESTS; VARIANCES ARE NOT ASSUMED TO BE EQUAL | MINIMUM -0.362

| WELCH 3, 3 4.91 0.1120 | CASES EXCLUDED ( 5)

| BROWN-FORSYTHE 3, 4 2.78 0.1742 | CASES INCLUDED 11

|--------------------------------------------------------------------------|

| LEVENE'S TEST FOR VARIANCES 3, 7 2.10 0.1891 | ROBUST S.D. 0.141

----------------------------------------------------------------------------

/ FINISH

PROGRAM TERMINATED

BMDP7D - ONE- AND TWO-WAY ANALYSIS OF VARIANCE WITH DATA SCREENING

Copyright 1977, 1979, 1981, 1982, 1983, 1985, 1987, 1988, 1990, 1993

by BMDP Statistical Software, Inc.

Statistical Solutions Ltd. | Statistical Solutions

Unit 1A, South Ring Business Park | Stonehill Corporate Center, Suite 104

Kinsale Road, Cork, Ireland | 999 Broadway, Saugus, MA 01906, USA

Phone: + 353 21 4319629 | Phone: 781.231.7680

Fax: + 353 21 4319630 | Fax: 781.231.7684

e-mail: sales@statsol.ie | e-mail: info@statsolusa.com

Website: http://www.statsol.ie | Website: http://www.statsolusa.com

Release: 8.1 (Windows 9x, 2000, Me, Xp) Date: 05/31/17 at 10:04:37

Manual: BMDP Manual Volumes 1, 2, and 3.

Digest: BMDP User's Digest.

IBM PC: BMDP PC Supplement -- Installation and Special Features.

PROGRAM INSTRUCTIONS

/prob title = 'Herr Lauritz Englisch: C7D1.inp *** Quantitative Studien

zum Schmelzgehalt in Pferdebackenzaehnen.

- 1 Fall = 1 Zahn = 10 Lokalisationen = 10 Zeilen

- Hier: Oberkiefer, Zahn 7 - 10

- Umrechnung der relativen Werte in Prozentwerte

- Berechnung der Flächenanteile ohne aeusseren Zement

- mit Berechnung der Steigung "ST" ueber die Schnittebenen

- einfaktorielle Varianzanalyse

- ergaenzt um die Parameter infm = ism+izm und infd = isd+izd

***'.

/inp var = 278.

file = c.

format = '34f10,9(/70x,27f10), /40x,1f10'. ## Mit Abstandsangabe für die Ebene

/var names = zahnid,pferd,rasse,zp,qu,znr,za,

for lo = 0 to 9.% ## Lokalisation

for va = lok,statu,ps,pz,isd,izd,ism,izm,idz,id,ges,sges,dz_sb,i_zb,

inf,ps_rel,s_rel,pz_r,isd_r,izd_r,ism_r,izm_r,idz_r,

id_r,sges_r,i_zb_r,inf_r.%

|va||lo|,%%

abst99. ## Mit Abstandsangabe für die Ebene 99

use = pferd,zp,qu,znr,za,

for va = lok,statu,ps,pz,isd,izd,ism,izm,idz,id,ges,sges,dz_sb,i_zb,

inf,ps_rel,s_rel,pz_r,isd_r,izd_r,ism_r,izm_r,idz_r,

id_r,sges_r,i_zb_r,inf_r,dges,infm,infd.%

for lo = 0 to 9.% ## Lokalisation

|va||lo|,%%

for va = ps,pz,isd,izd,ism,izm,idz,id,ges,sges,dz_sb,i_zb,

inf,ps_rel,s_rel,pz_r,isd_r,izd_r,ism_r,izm_r,idz_r,

id_r,sges_r,i_zb_r,inf_r,dges,infm,infd.%

ST|va|,%

for va = ps,id,isd,ism,sges,inf,infm,infd.%

ST|va|r2,%

.

/group codes(pferd) = 9,10,15,16,18. ## diese Pferde haben mehr als 1 Zahn in

/trans use = ((znr ge 7) AND (znr le 10)).

lok9 = abst99. ## Abstandsangabe für die Ebene 99

# Umrechnung der relativen Werte in Prozentwerte

for va = ps_rel,s_rel,pz_r,isd_r,izd_r,ism_r,izm_r,idz_r,

id_r,sges_r,i_zb_r,inf_r.%

for lo = 0 to 9.%

|va||lo| = |va||lo| * 100.%%

# Berechnung zusaetzlicher Flaechen

# infd = isd+izd und infm = ism+izm

for se = m, d.%

for lo = 0 to 9.%

inf|se||lo| = is|se||lo| + is|se||lo|.%%

# Berechnung der Flächenanteile ohne aeusseren Zement

for va = ps,id,isd,ism,sges,inf,infm,infd.%

for lo = 0 to 9.%

|va|_r2|lo| = |va||lo|/(ges|lo|-pz|lo|) * 100.%%

# Berechnung der Gesamtflaeche abzüglich peripherem Zement

for lo = 0 to 9.%

dges|lo| = ges|lo|-pz|lo|.%

# Berechnung der Steigung "ST" ueber die Schnittebenen

for va = ps,pz,isd,izd,ism,izm,idz,id,ges,sges,dz_sb,i_zb,

inf,ps_rel,s_rel,pz_r,isd_r,izd_r,ism_r,izm_r,idz_r,

id_r,sges_r,i_zb_r,inf_r,dges,infm,infd.%

ST|va| = b(for lo = 0 to 9.% lok|lo|,|va||lo|,%).

%

for va = ps,id,isd,ism,sges,inf,infm,infd.%

ST|va|r2 = b(for lo = 0 to 9.% lok|lo|,|va|_r2|lo|,%).

%

/hist group = Pferd.

var = for va = ps,pz,isd,izd,ism,izm,idz,id,ges,sges,dz_sb,i_zb,

inf,ps_rel,s_rel,pz_r,isd_r,izd_r,ism_r,izm_r,idz_r,

id_r,sges_r,i_zb_r,inf_r,dges,infm,infd.%

ST|va|,%

for va = ps,id,isd,ism,sges,inf,infm,infd.%

ST|va|r2,%.

/print level = min.

case = 0.

/end

--- PROGRAM INSTRUCTIONS AFTER "FOR %" EXPANSION ---

/prob title = 'Herr Lauritz Englisch: C7D1.inp *** Quantitative Studien zum

Schmelzgehalt in Pferdebackenzaehnen.

- 1 Fall = 1 Zahn = 10 Lokalisationen =

10 Zeilen - Hier: Oberkiefer,

Zahn 7 - 10 - Umrechnung der relativen Werte in Prozentwerte -

Berechnung der Flächenanteile ohne aeusseren Zement - mit Berechnung

der Steigung "ST" ueber die Schnittebenen - einfaktorielle

Varianzanalyse - ergaenzt um die Parameter infm =

ism+izm und infd = isd+izd ***'.

/inp var = 278. file = c. format = '34f10,9(/70x,27f10),

/40x,1f10'.

/var names = zahnid,pferd,rasse,zp,qu,znr,za, lok0, statu0, ps0, pz0, isd0,

izd0, ism0, izm0, idz0, id0, ges0, sges0, dz_sb0, i_zb0, inf0,

...

STsgesr2, STinfr2, STinfmr2, STinfdr2 .

/group codes(pferd) = 9,10,15,16,18.

/trans use = ((znr ge 7) AND (znr le 10)). lok9 = abst99.

ps_rel0 = ps_rel0 * 100. ps_rel1 = ps_rel1 * 100.

...

STinfdr2 = b( lok0,infd_r20, lok1,infd_r21, lok2,infd_r22, lok3,infd_r23,

lok4,infd_r24, lok5,infd_r25, lok6,infd_r26, lok7,infd_r27, lok8,

infd_r28, lok9,infd_r29).

/hist group = Pferd. var = STps, STpz, STisd, STizd, STism, STizm, STidz,

STid, STges, STsges, STdz_sb, STi_zb, STinf, STps_rel, STs_rel,

STpz_r, STisd_r, STizd_r, STism_r, STizm_r, STidz_r, STid_r,

STsges_r, STi_zb_r, STinf_r, STdges, STinfm, STinfd, STpsr2,

STidr2, STisdr2, STismr2, STsgesr2, STinfr2, STinfmr2, STinfdr2.

/print level = min. case = 0.

/end/

NUMBER OF CASES READ. . . . . . . . . . . . . . 28

CASES WITH USE SET TO ZERO . . . . . . . . . 8

REMAINING NUMBER OF CASES . . . . . . . . 20

DESCRIPTIVE STATISTICS OF DATA

----------- ---------- -- ----

VARIABLE TOTAL STANDARD ST.ERR COEFF SMALLEST LARGEST

NO. NAME FREQ. MEAN DEV. OF MEAN OF VAR VALUE VALUE RANGE

2 pferd 20 12.400 4.4651 .99842 .36009 2.0000 18.000 16.000

4 zp 20 148.50 50.380 11.265 .33926 107.00 210.00 103.00

5 qu 20 1.4000 .50262 .11239 .35902 1.0000 2.0000 1.0000

6 znr 20 8.5000 1.1471 .25649 .13495 7.0000 10.000 3.0000

7 za 20 10.525 5.7181 1.2786 .54329 1.0000 20.000 19.000

8 lok0 20 0.0000 0.0000 0.0000 0.0000 0.0000 0.0000

35 lok1 20 10.000 0.0000 0.0000 0.0000 10.000 10.000 0.0000

62 lok2 20 20.000 0.0000 0.0000 0.0000 20.000 20.000 0.0000

89 lok3 20 30.000 0.0000 0.0000 0.0000 30.000 30.000 0.0000

116 lok4 20 40.000 0.0000 0.0000 0.0000 40.000 40.000 0.0000

143 lok5 20 50.000 0.0000 0.0000 0.0000 50.000 50.000 0.0000

170 lok6 20 60.000 0.0000 0.0000 0.0000 60.000 60.000 0.0000

197 lok7 20 70.000 0.0000 0.0000 0.0000 70.000 70.000 0.0000

224 lok8 20 80.000 0.0000 0.0000 0.0000 80.000 80.000 0.0000

251 lok9 20 38.955 13.905 3.1093 .35696 21.910 62.490 40.580

9 statu0 20 1.0000 0.0000 0.0000 0.0000 1.0000 1.0000 0.0000

36 statu1 20 1.3500 .48936 .10942 .36249 1.0000 2.0000 1.0000

63 statu2 20 1.9500 .94451 .21120 .48437 1.0000 3.0000 2.0000

90 statu3 20 2.2000 .95145 .21275 .43248 1.0000 3.0000 2.0000

117 statu4 20 2.5500 .75915 .16975 .29771 1.0000 3.0000 2.0000

144 statu5 20 2.8500 .48936 .10942 .17171 1.0000 3.0000 2.0000

171 statu6 20 3.0000 0.0000 0.0000 0.0000 3.0000 3.0000 0.0000

198 statu7 20 3.0000 0.0000 0.0000 0.0000 3.0000 3.0000 0.0000

225 statu8 20 3.0000 0.0000 0.0000 0.0000 3.0000 3.0000 0.0000

252 statu9 20 3.0000 0.0000 0.0000 0.0000 3.0000 3.0000 0.0000

10 ps0 19 91.440 19.876 4.5600 .21737 61.020 126.63 65.610

37 ps1 19 109.67 17.667 4.0530 .16108 80.020 140.16 60.140

64 ps2 19 105.02 14.384 3.2999 .13697 81.630 130.06 48.430

91 ps3 9 107.33 14.584 4.8612 .13588 88.040 139.81 51.770

118 ps4 9 104.03 13.034 4.3446 .12528 86.670 125.62 38.950

145 ps5 5 99.996 6.7903 3.0367 .06791 93.090 109.45 16.360

172 ps6 2 110.14 1.1950 .84500 .01085 109.29 110.98 1.6900

199 ps7 0

226 ps8 0

253 ps9 20 100.38 14.910 3.3340 .14853 72.580 123.85 51.270

11 pz0 19 27.856 12.064 2.7676 .43307 10.680 57.830 47.150

38 pz1 19 76.448 40.825 9.3659 .53402 30.350 165.71 135.36

65 pz2 19 104.01 59.192 13.580 .56912 33.970 201.47 167.50

92 pz3 9 62.862 29.859 9.9528 .47498 36.950 133.22 96.270

119 pz4 9 107.63 49.818 16.606 .46284 43.970 180.19 136.22

146 pz5 5 133.81 61.615 27.555 .46047 67.240 206.04 138.80

173 pz6 2 144.55 8.2095 5.8050 .05679 138.75 150.36 11.610

200 pz7 0

227 pz8 0

254 pz9 20 143.61 33.160 7.4148 .23090 58.230 185.52 127.29

12 isd0 2 17.975 11.646 8.2350 .64790 9.7400 26.210 16.470

39 isd1 19 19.391 5.5500 1.2732 .28621 9.0600 28.860 19.800

66 isd2 19 23.437 4.1787 .95867 .17830 16.330 30.330 14.000

93 isd3 9 26.367 4.5894 1.5298 .17406 20.620 34.060 13.440

120 isd4 9 25.148 5.3570 1.7857 .21302 18.380 32.790 14.410

147 isd5 5 25.024 4.2302 1.8918 .16904 22.010 32.170 10.160

174 isd6 2 27.405 1.0394 .73500 .03793 26.670 28.140 1.4700

201 isd7 0

228 isd8 0

255 isd9 20 25.475 5.8267 1.3029 .22873 12.740 38.880 26.140

13 izd0 2 17.375 8.6479 6.1150 .49772 11.260 23.490 12.230

40 izd1 18 32.674 11.566 2.7262 .35398 18.500 57.470 38.970

67 izd2 18 39.324 11.196 2.6390 .28472 27.020 67.690 40.670

94 izd3 9 45.081 7.1701 2.3900 .15905 35.300 56.420 21.120

121 izd4 9 48.486 8.0586 2.6862 .16621 36.650 60.170 23.520

148 izd5 5 51.936 7.3663 3.2943 .14183 42.930 60.070 17.140

175 izd6 2 55.440 12.756 9.0200 .23009 46.420 64.460 18.040

202 izd7 0

229 izd8 0

256 izd9 19 43.121 11.548 2.6492 .26779 18.890 67.430 48.540

14 ism0 2 25.075 9.9207 7.0150 .39564 18.060 32.090 14.030

41 ism1 16 19.428 7.0617 1.7654 .36348 7.0200 30.200 23.180

68 ism2 18 23.761 5.7375 1.3524 .24147 12.340 35.950 23.610

95 ism3 9 27.048 5.4905 1.8302 .20299 19.820 35.990 16.170

122 ism4 9 28.280 5.1347 1.7116 .18157 22.980 37.670 14.690

149 ism5 5 29.516 3.9994 1.7886 .13550 25.220 36.090 10.870

176 ism6 2 28.525 2.0577 1.4550 .07214 27.070 29.980 2.9100

203 ism7 0

230 ism8 0

257 ism9 19 27.346 6.9131 1.5860 .25280 8.1300 35.520 27.390

15 izm0 2 29.445 14.545 10.285 .49398 19.160 39.730 20.570

42 izm1 16 34.914 16.757 4.1892 .47994 11.600 70.430 58.830

69 izm2 17 42.056 15.164 3.6778 .36056 24.110 87.670 63.560

96 izm3 9 47.191 13.019 4.3396 .27588 30.040 77.700 47.660

123 izm4 9 50.382 9.9108 3.3036 .19671 34.730 65.400 30.670

150 izm5 5 53.678 9.5213 4.2580 .17738 44.420 67.150 22.730

177 izm6 2 67.620 17.706 12.520 .26184 55.100 80.140 25.040

204 izm7 0

231 izm8 0

258 izm9 19 45.087 15.460 3.5468 .34290 10.740 79.630 68.890

16 idz0 16 408.09 64.338 16.085 .15766 268.83 532.84 264.01

43 idz1 3 428.54 64.107 37.012 .14959 374.97 499.57 124.60

70 idz2 2 419.08 62.282 44.040 .14862 375.04 463.12 88.080

97 idz3 0

124 idz4 0

151 idz5 0

178 idz6 0

205 idz7 0

232 idz8 0

259 idz9 1 464.16 0.0000 0.0000 0.0000 464.16 464.16 0.0000

17 id0 3 371.88 34.456 19.893 .09265 332.15 393.62 61.470

44 id1 15 307.15 42.389 10.945 .13801 217.86 361.91 144.05

71 id2 16 296.72 37.677 9.4194 .12698 221.12 347.10 125.98

98 id3 9 306.56 37.375 12.458 .12192 236.68 352.81 116.13

125 id4 9 311.45 42.988 14.329 .13803 237.23 376.13 138.90

152 id5 5 318.26 31.445 14.063 .09880 281.96 347.51 65.550

179 id6 2 302.71 45.806 32.390 .15132 270.32 335.10 64.780

206 id7 0

233 id8 0

260 id9 18 303.18 42.772 10.082 .14108 219.02 381.43 162.41

18 ges0 19 531.13 84.211 19.319 .15855 364.46 698.46 334.00

45 ges1 19 592.38 112.61 25.835 .19010 256.10 769.83 513.73

72 ges2 19 623.84 108.77 24.953 .17435 310.19 816.44 506.25

99 ges3 9 622.43 50.465 16.822 .08108 558.93 713.23 154.30

126 ges4 9 675.41 95.434 31.811 .14130 557.10 820.81 263.71

153 ges5 5 712.23 82.969 37.105 .11649 585.75 811.21 225.46

180 ges6 2 736.38 80.186 56.700 .10889 679.68 793.08 113.40

207 ges7 0

234 ges8 0

261 ges9 20 675.32 134.03 29.971 .19848 274.95 837.13 562.18

19 sges0 19 95.972 30.246 6.9388 .31515 61.020 180.76 119.74

46 sges1 19 145.43 26.976 6.1887 .18550 104.59 191.73 87.140

73 sges2 19 150.97 22.395 5.1377 .14834 116.70 196.33 79.630

100 sges3 9 160.74 22.288 7.4294 .13866 136.65 204.78 68.130

127 sges4 9 157.46 21.960 7.3200 .13946 136.68 195.15 58.470

154 sges5 5 154.54 12.362 5.5286 .08000 143.28 172.88 29.600

181 sges6 2 166.07 4.2921 3.0350 .02585 163.03 169.10 6.0700

208 sges7 0

235 sges8 0

262 sges9 20 151.84 24.166 5.4036 .15916 109.22 197.56 88.340

20 dz_sb0 19 411.84 66.938 15.357 .16254 268.83 532.84 264.01

47 dz_sb1 19 406.26 102.02 23.406 .25113 64.550 509.58 445.03

74 dz_sb2 19 414.81 95.007 21.796 .22904 90.360 498.70 408.34

101 dz_sb3 9 452.24 53.317 17.772 .11789 351.08 510.37 159.29

128 dz_sb4 9 463.74 60.647 20.216 .13078 358.62 539.28 180.66

155 dz_sb5 5 478.42 47.633 21.302 .09956 421.74 521.97 100.23

182 dz_sb6 2 481.70 73.178 51.745 .15192 429.95 533.44 103.49

209 dz_sb7 0

236 dz_sb8 0

263 dz_sb9 20 431.32 99.738 22.302 .23124 105.38 558.56 453.18

21 i_zb0 6 15.607 26.309 10.740 1.6857 0.0000 63.220 63.220

48 i_zb1 18 63.708 25.984 6.1245 .40786 20.620 115.16 94.540

75 i_zb2 18 79.044 27.321 6.4396 .34564 27.670 150.00 122.33

102 i_zb3 9 92.272 19.559 6.5196 .21197 65.340 134.12 68.780

129 i_zb4 9 98.867 17.898 5.9659 .18103 71.380 125.57 54.190

156 i_zb5 5 105.61 16.684 7.4611 .15797 88.320 127.22 38.900

183 i_zb6 2 123.06 30.462 21.540 .24754 101.52 144.60 43.080

210 i_zb7 0

237 i_zb8 0

264 i_zb9 19 88.209 26.547 6.0904 .30096 29.630 147.06 117.43

22 inf0 7 25.679 47.510 17.957 1.8502 0.0000 121.53 121.53

49 inf1 19 96.108 40.188 9.2197 .41815 10.010 166.57 156.56

76 inf2 19 120.83 38.757 8.8915 .32075 18.340 192.82 174.48

103 inf3 9 145.69 19.430 6.4767 .13337 114.40 177.40 63.000

130 inf4 9 152.30 20.014 6.6714 .13142 121.40 173.17 51.770

157 inf5 5 160.16 17.008 7.6064 .10620 139.78 174.63 34.850

184 inf6 2 178.99 27.372 19.355 .15293 159.63 198.34 38.710

211 inf7 0

238 inf8 0

265 inf9 20 135.25 44.370 9.9213 .32805 20.340 203.33 182.99

23 ps_rel0 18 17.235 2.3821 .56147 .13821 11.363 20.457 9.0941

50 ps_rel1 19 18.911 3.2957 .75608 .17427 15.228 31.246 16.017

77 ps_rel2 19 17.173 2.8624 .65668 .16668 14.413 26.316 11.903

104 ps_rel3 9 17.250 1.8453 .61512 .10698 14.672 19.603 4.9309

131 ps_rel4 9 15.552 2.0834 .69445 .13396 12.433 19.228 6.7956

158 ps_rel5 5 14.153 1.4748 .65956 .10421 12.897 16.521 3.6242

185 ps_rel6 2 15.054 1.8020 1.2742 .11970 13.780 16.328 2.5484

212 ps_rel7 0

239 ps_rel8 0

266 ps_rel9 20 15.291 2.9558 .66094 .19331 12.785 26.398 13.613

24 s_rel0 18 22.366 3.9169 .92322 .17513 13.500 28.261 14.762

51 s_rel1 19 31.444 22.554 5.1743 .71730 20.629 123.97 103.34

78 s_rel2 19 28.192 15.316 3.5137 .54327 19.147 90.339 71.191

105 s_rel3 9 23.933 3.5218 1.1739 .14715 19.073 29.790 10.716

132 s_rel4 9 22.596 2.6601 .88670 .11772 19.078 26.384 7.3056

159 s_rel5 5 21.002 1.6496 .73773 .07855 18.401 22.946 4.5451

186 s_rel6 2 23.149 3.7655 2.6626 .16266 20.487 25.812 5.3252

213 s_rel7 0

240 s_rel8 0

267 s_rel9 20 25.135 10.496 2.3469 .41757 19.720 68.875 49.155

25 pz_r0 18 5.1635 2.0452 .48205 .39608 1.8497 9.2853 7.4356

52 pz_r1 19 13.779 9.3213 2.1384 .67648 4.8429 43.549 38.707

79 pz_r2 19 17.055 10.365 2.3778 .60773 5.3265 44.553 39.227

106 pz_r3 9 10.176 5.1604 1.7201 .50713 6.5927 22.622 16.029

133 pz_r4 9 15.560 6.2185 2.0728 .39964 7.8924 25.340 17.448

160 pz_r5 5 18.340 7.1584 3.2013 .39031 11.479 26.208 14.729

187 pz_r6 2 19.686 1.0292 .72779 .05228 18.958 20.414 1.4556

214 pz_r7 0

241 pz_r8 0

268 pz_r9 20 21.576 4.5885 1.0260 .21267 12.054 35.276 23.222

26 isd_r0 2 2.7414 1.7001 1.2022 .62017 1.5393 3.9436 2.4044

53 isd_r1 19 3.3935 1.0730 .24616 .31620 1.3463 5.5994 4.2530

80 isd_r2 19 3.8459 .82103 .18836 .21348 2.4771 5.8996 3.4225

107 isd_r3 9 4.2309 .58326 .19442 .13786 3.2526 4.7995 1.5469

134 isd_r4 9 3.7211 .54329 .18110 .14600 2.7823 4.2655 1.4832

161 isd_r5 5 3.5156 .39930 .17857 .11358 3.0639 3.9658 .90189

188 isd_r6 2 3.7518 .54929 .38841 .14641 3.3633 4.1402 .77681

215 isd_r7 0

242 isd_r8 0

269 isd_r9 20 3.8701 1.0192 .22790 .26335 2.6377 7.4486 4.8110

27 izd_r0 2 2.6574 1.2400 .87681 .46663 1.7806 3.5342 1.7536

54 izd_r1 18 5.6955 1.9160 .45162 .33641 3.2422 9.1092 5.8670

81 izd_r2 18 6.5196 1.9324 .45547 .29640 3.8515 10.720 6.8688

108 izd_r3 9 7.2446 1.0117 .33725 .13965 5.9946 8.8987 2.9041

135 izd_r4 9 7.2095 .99035 .33012 .13737 5.6950 9.1072 3.4121

162 izd_r5 5 7.3131 .86338 .38611 .11806 6.4226 8.5835 2.1609

189 izd_r6 2 7.4791 .91792 .64907 .12273 6.8300 8.1282 1.2981

216 izd_r7 0

243 izd_r8 0

270 izd_r9 19 6.5553 1.7683 .40566 .26974 3.9113 11.999 8.0873

28 ism_r0 2 3.8416 1.3949 .98636 .36311 2.8553 4.8280 1.9727

55 ism_r1 16 3.3451 .93142 .23286 .27845 1.2011 4.5147 3.3136

82 ism_r2 18 3.9205 .91366 .21535 .23304 1.7175 5.4562 3.7387

109 ism_r3 9 4.3414 .76784 .25595 .17687 3.1345 5.4828 2.3484

136 ism_r4 9 4.1975 .54331 .18110 .12943 3.4784 4.9788 1.5003

163 ism_r5 5 4.1668 .54467 .24358 .13072 3.6038 4.9716 1.3678

190 ism_r6 2 3.9118 .70489 .49844 .18020 3.4134 4.4102 .99687

217 ism_r7 0

244 ism_r8 0

271 ism_r9 19 4.1423 1.0894 .24992 .26300 1.6828 7.4123 5.7295

29 izm_r0 2 4.5030 2.0852 1.4744 .46306 3.0285 5.9774 2.9488

56 izm_r1 16 6.0086 2.5302 .63255 .42110 2.3230 11.848 9.5254

83 izm_r2 17 6.9379 2.2802 .55302 .32865 4.4626 13.901 9.4386

110 izm_r3 9 7.5686 1.9458 .64859 .25709 5.1005 12.255 7.1546

137 izm_r4 9 7.4888 1.3050 .43499 .17426 5.7122 9.8979 4.1857

164 izm_r5 5 7.5774 1.3348 .59693 .17615 6.2366 9.5951 3.3585

191 izm_r6 2 9.1053 1.4130 .99912 .15518 8.1062 10.104 1.9982

218 izm_r7 0

245 izm_r8 0

272 izm_r9 19 6.7762 2.1116 .48444 .31162 2.2229 11.468 9.2447

30 idz_r0 16 78.036 3.3612 .84029 .04307 72.178 84.171 11.993

57 idz_r1 3 65.544 1.4224 .82120 .02170 64.583 67.178 2.5950

84 idz_r2 2 57.385 7.3266 5.1807 .12768 52.204 62.566 10.361

111 idz_r3 0

138 idz_r4 0

165 idz_r5 0

192 idz_r6 0

219 idz_r7 0

246 idz_r8 0

273 idz_r9 1 62.200 0.0000 0.0000 0.0000 62.200 62.200 0.0000

31 id_r0 3 65.682 9.1836 5.3021 .13982 59.216 76.193 16.977

58 id_r1 15 51.040 3.7470 .96747 .07341 44.829 56.947 12.119

85 id_r2 16 47.248 4.6451 1.1613 .09831 40.087 53.826 13.738

112 id_r3 9 49.189 3.7591 1.2530 .07642 40.192 53.226 13.034

139 id_r4 9 46.270 3.9831 1.3277 .08608 40.315 51.445 11.130

166 id_r5 5 44.934 4.3640 1.9516 .09712 40.178 49.654 9.4761

193 id_r6 2 41.012 1.7545 1.2406 .04278 39.771 42.253 2.4812

220 id_r7 0

247 id_r8 0

274 id_r9 17 43.703 4.5509 1.1037 .10413 38.699 59.202 20.503

32 sges_r0 19 17.900 3.5740 .81993 .19967 11.363 27.194 15.832

59 sges_r1 19 25.122 4.9465 1.1348 .19690 17.917 40.840 22.923

86 sges_r2 19 24.733 4.4974 1.0318 .18184 17.579 37.622 20.043

113 sges_r3 9 25.822 2.7339 .91131 .10588 21.553 28.878 7.3256

140 sges_r4 9 23.471 2.7650 .92166 .11780 19.345 28.044 8.6991

167 sges_r5 5 21.835 1.9877 .88893 .09103 20.473 25.306 4.8330

194 sges_r6 2 22.718 3.0561 2.1610 .13453 20.557 24.879 4.3220

221 sges_r7 0

248 sges_r8 0

275 sges_r9 20 23.096 4.5919 1.0268 .19882 17.310 41.258 23.948

33 i_zb_r0 3 4.7735 4.7559 2.7458 .99630 0.0000 9.5116 9.5116

60 i_zb_r1 18 11.036 4.0861 .96310 .37023 3.2543 19.373 16.119

87 i_zb_r2 18 13.072 4.4220 1.0423 .33828 3.8515 23.785 19.934

114 i_zb_r3 9 14.813 2.8507 .95024 .19245 11.095 21.154 10.059

141 i_zb_r4 9 14.698 2.2853 .76176 .15548 11.407 19.005 7.5978

168 i_zb_r5 5 14.890 2.1952 .98173 .14742 12.659 18.179 5.5194

195 i_zb_r6 2 16.584 2.3309 1.6482 .14055 14.936 18.233 3.2964

222 i_zb_r7 0

249 i_zb_r8 0

276 i_zb_r9 19 13.331 3.7776 .86663 .28336 6.1341 23.466 17.332

34 inf_r0 3 9.1622 9.1416 5.2779 .99775 0.0000 18.283 18.283

61 inf_r1 19 16.666 6.5257 1.4971 .39156 1.3463 28.023 26.676

88 inf_r2 19 19.944 6.5318 1.4985 .32751 2.4771 30.575 28.098

115 inf_r3 9 23.386 2.3177 .77257 .09911 19.426 27.979 8.5533

142 inf_r4 9 22.617 1.5852 .52839 .07009 19.878 25.266 5.3881

169 inf_r5 5 22.573 1.8701 .83632 .08285 20.200 24.928 4.7284

196 inf_r6 2 24.248 1.0767 .76135 .04440 23.487 25.009 1.5227

223 inf_r7 0

250 inf_r8 0

277 inf_r9 20 20.470 6.7460 1.5085 .32955 2.7257 38.327 35.601

379 dges0 19 503.28 80.411 18.448 .15978 339.24 649.47 310.23

380 dges1 19 515.93 115.56 26.511 .22398 144.57 636.55 491.98

381 dges2 19 519.83 104.45 23.963 .20093 171.99 623.73 451.74

382 dges3 9 559.57 60.374 20.125 .10789 455.66 650.19 194.53

383 dges4 9 567.77 69.301 23.100 .12206 445.29 664.91 219.62

384 dges5 5 578.42 52.007 23.258 .08991 518.51 624.82 106.31

385 dges6 2 591.83 71.976 50.895 .12162 540.93 642.72 101.79

386 dges7 0

387 dges8 0

388 dges9 20 531.71 111.96 25.036 .21058 177.96 682.40 504.44

279 infm0 2 50.150 19.841 14.030 .39564 36.120 64.180 28.060

280 infm1 16 38.856 14.123 3.5308 .36348 14.040 60.400 46.360

281 infm2 18 47.521 11.475 2.7047 .24147 24.680 71.900 47.220

282 infm3 9 54.096 10.981 3.6604 .20299 39.640 71.980 32.340

283 infm4 9 56.560 10.269 3.4231 .18157 45.960 75.340 29.380

284 infm5 5 59.032 7.9987 3.5771 .13550 50.440 72.180 21.740

285 infm6 2 57.050 4.1154 2.9100 .07214 54.140 59.960 5.8200

286 infm7 0

287 infm8 0

288 infm9 19 54.692 13.826 3.1719 .25280 16.260 71.040 54.780

289 infd0 2 35.950 23.292 16.470 .64790 19.480 52.420 32.940

290 infd1 19 38.782 11.100 2.5465 .28621 18.120 57.720 39.600

291 infd2 19 46.874 8.3575 1.9173 .17830 32.660 60.660 28.000

292 infd3 9 52.733 9.1788 3.0596 .17406 41.240 68.120 26.880

293 infd4 9 50.296 10.714 3.5714 .21302 36.760 65.580 28.820

294 infd5 5 50.048 8.4603 3.7836 .16904 44.020 64.340 20.320

295 infd6 2 54.810 2.0789 1.4700 .03793 53.340 56.280 2.9400

296 infd7 0

297 infd8 0

298 infd9 20 50.949 11.653 2.6058 .22873 25.480 77.760 52.280

389 STps 20 .10244 .44074 .09855 4.3023 -.72635 .94844 1.6748

390 STpz 20 3.5175 1.5044 .33639 .42769 1.1825 6.1917 5.0092

391 STisd 20 .24767 .29963 .06700 1.2098 -.36397 .85278 1.2168

392 STizd 19 .41392 .47767 .10959 1.1540 -.72621 1.1859 1.9121

393 STism 19 .31048 .36442 .08360 1.1737 -.40273 1.2963 1.6990

394 STizm 18 .44786 .50214 .11836 1.1212 -.45150 1.6454 2.0969

395 STidz 3 -3.8165 .56340 .32528 -.14762 -4.3950 -3.2695 1.1255

396 STid 17 -.43881 1.0030 .24327 -2.2858 -2.6450 1.4015 4.0465

397 STges 20 3.9876 2.7644 .61814 .69326 -4.2186 7.5714 11.790

398 STsges 20 1.2304 .77398 .17307 .62903 -.28135 2.7688 3.0502

399 STdz_sb 20 .36759 2.0736 .46367 5.6411 -6.9899 2.4539 9.4438

400 STi_zb 19 1.2475 1.0851 .24893 .86980 -.71585 3.2989 4.0147

401 STinf 20 2.0636 1.7793 .39786 .86223 -1.4825 5.1589 6.6414

402 STps_rel 20 -.08023 .11444 .02559 -1.4265 -.28924 .26502 .55426

403 STs_rel 20 .04039 .31892 .07131 7.8965 -.27108 1.2871 1.5582

404 STpz_r 20 .48786 .21698 .04852 .44477 .23418 .99384 .75966

405 STisd_r 20 .02146 .04762 .01065 2.2192 -.04506 .11447 .15954

406 STizd_r 19 .03995 .08885 .02038 2.2239 -.18255 .20404 .38659

407 STism_r 19 .03412 .06733 .01545 1.9731 -.05901 .19337 .25239

408 STizm_r 18 .04332 .08869 .02090 2.0471 -.06623 .23450 .30072

409 STidz_r 3 -1.1389 .46445 .26815 -.40782 -1.5502 -.63518 .91502

410 STid_r 17 -.36461 .21140 .05127 -.57982 -.86967 -.03916 .83051

411 STsges_r 20 .09107 .19685 .04402 2.1616 -.23723 .77115 1.0084

412 STi_zb_r 19 .11567 .20403 .04681 1.7638 -.08151 .79413 .87563

413 STinf_r 20 .17689 .31973 .07149 1.8075 -.18558 1.3003 1.4858

414 STdges 20 .47005 2.1068 .47110 4.4822 -6.9082 2.5680 9.4762

415 STinfm 19 .62096 .72883 .16721 1.1737 -.80547 2.5925 3.3980

416 STinfd 20 .49534 .59926 .13400 1.2098 -.72794 1.7056 2.4335

417 STpsr2 20 .02583 .17782 .03976 6.8854 -.17178 .69706 .86884

418 STidr2 17 -.16024 .17480 .04239 -1.0909 -.45418 .16972 .62390

419 STisdr2 20 .04122 .05515 .01233 1.3379 -.07829 .15309 .23138

420 STismr2 19 .06007 .08277 .01899 1.3778 -.08885 .25253 .34138

421 STsgesr2 20 .26166 .32271 .07216 1.2333 -.07710 1.4956 1.5727

422 STinfr2 20 .43071 .51130 .11433 1.1871 -.31938 2.0505 2.3699

423 STinfmr2 19 .12015 .16555 .03798 1.3778 -.17770 .50507 .68276

424 STinfdr2 20 .08244 .11029 .02466 1.3379 -.15659 .30618 .46277

************ ************

HISTOGRAM OF * STps * ( 389) GROUPED BY * pferd * ( 2)

************ ************

CASES WITH

UNUSED

VALUES FOR

*9 *10 *15 *16 *18 pferd

MIDPOINTS...................+...................+...................+...................+...................+...................+

1.05000)

0.90000) * *

0.75000)

0.60000) **

0.45000)

0.30000) N * *

0.15000) M** M M*

0.00000) N *

-0.15000)M*

-0.30000) **

-0.45000) * *

-0.60000)

-0.75000) *

LEGEND FOR GROUP MEANS: M - MEAN COINCIDES WITH AN ASTERISK

N - MEAN DOES NOT COINCIDE WITH ANY ASTERISK

MEAN -0.122 0.142 0.026 0.249 0.178 0.130

STD.DEV. 0.002 0.060 0.721 0.921 0.129 0.422

S. E. M. 0.001 0.034 0.361 0.652 0.091 0.160

MAXIMUM -0.121 0.201 0.672 0.901 0.269 0.948

MINIMUM -0.123 0.082 -0.726 -0.402 0.087 -0.326

CASES EXCL. ( 0) ( 0) ( 0) ( 0) ( 0) ( 0)

CASES INCL. 2 3 4 2 2 7

---------------------------------------------------------------------------- ALL GROUPS COMBINED

| ANALYSIS OF VARIANCE TABLE FOR MEANS TAIL | (EXCEPT CASES WITH UNUSED

| SOURCE SUM OF SQUARES DF MEAN SQUARE F VALUE PROBABILITY | VALUES FOR VARIABLE pferd )

| -------- -------------- ---- ----------- ------- ----------- | MEAN 0.088

| pferd 0.1805 4 0.0451 0.15 0.9586 | STD. DEV. 0.467

| ERROR 2.4331 8 0.3041 | S. E. M. 0.129

|--------------------------------------------------------------------------| MAXIMUM 0.901

| EQUALITY OF MEANS TESTS; VARIANCES ARE NOT ASSUMED TO BE EQUAL | MINIMUM -0.726

| WELCH 4, 3 10.25 0.0427 | CASES EXCLUDED ( 7)

| BROWN-FORSYTHE 4, 2 0.16 0.9386 | CASES INCLUDED 13

|--------------------------------------------------------------------------|

| LEVENE'S TEST FOR VARIANCES 4, 8 55.19 0.0000 | ROBUST S.D. 0.456

----------------------------------------------------------------------------

************ ************

HISTOGRAM OF * STpz * ( 390) GROUPED BY * pferd * ( 2)

************ ************

CASES WITH

UNUSED

VALUES FOR

*9 *10 *15 *16 *18 pferd

MIDPOINTS...................+...................+...................+...................+...................+...................+

6.50000)

6.00000) **

5.50000) * *

5.00000) N *

4.50000) *

4.00000) **

3.50000) * * N N

3.00000)* M *

2.50000)N * *

2.00000) * M*

1.50000)*

1.00000) *

0.50000)

LEGEND FOR GROUP MEANS: M - MEAN COINCIDES WITH AN ASTERISK

N - MEAN DOES NOT COINCIDE WITH ANY ASTERISK

MEAN 2.329 3.040 5.230 3.359 2.021 3.557

STD.DEV. 0.898 0.311 1.362 1.845 0.103 1.486

S. E. M. 0.635 0.179 0.681 1.305 0.073 0.562

MAXIMUM 2.964 3.284 6.192 4.664 2.093 5.390

MINIMUM 1.695 2.690 3.259 2.054 1.948 1.183

CASES EXCL. ( 0) ( 0) ( 0) ( 0) ( 0) ( 0)

CASES INCL. 2 3 4 2 2 7

---------------------------------------------------------------------------- ALL GROUPS COMBINED

| ANALYSIS OF VARIANCE TABLE FOR MEANS TAIL | (EXCEPT CASES WITH UNUSED

| SOURCE SUM OF SQUARES DF MEAN SQUARE F VALUE PROBABILITY | VALUES FOR VARIABLE pferd )

| -------- -------------- ---- ----------- ------- ----------- | MEAN 3.496

| pferd 19.7606 4 4.9402 3.96 0.0464 | STD. DEV. 1.574

| ERROR 9.9817 8 1.2477 | S. E. M. 0.437

|--------------------------------------------------------------------------| MAXIMUM 6.192

| EQUALITY OF MEANS TESTS; VARIANCES ARE NOT ASSUMED TO BE EQUAL | MINIMUM 1.695

| WELCH 4, 3 7.26 0.0676 | CASES EXCLUDED ( 7)

| BROWN-FORSYTHE 4, 3 4.01 0.1417 | CASES INCLUDED 13

|--------------------------------------------------------------------------|

| LEVENE'S TEST FOR VARIANCES 4, 8 2.91 0.0927 | ROBUST S.D. 1.673

----------------------------------------------------------------------------

************ ************

HISTOGRAM OF * STisd * ( 391) GROUPED BY * pferd * ( 2)

************ ************

CASES WITH

UNUSED

VALUES FOR

*9 *10 *15 *16 *18 pferd

MIDPOINTS...................+...................+...................+...................+...................+...................+

0.960000)

0.840000) * **

0.720000)

0.600000)

0.480000) N

0.360000) **

0.240000) N ** * M M*

0.120000)M * M* * *

0.000000)* *

-.120000)

-.240000)

-.360000) *

-.480000)

LEGEND FOR GROUP MEANS: M - MEAN COINCIDES WITH AN ASTERISK

N - MEAN DOES NOT COINCIDE WITH ANY ASTERISK

MEAN 0.067 0.287 0.156 0.533 0.182 0.272

STD.DEV. 0.040 0.117 0.070 0.452 0.106 0.441

S. E. M. 0.028 0.067 0.035 0.320 0.075 0.167

MAXIMUM 0.096 0.356 0.241 0.853 0.257 0.850

MINIMUM 0.039 0.152 0.098 0.213 0.107 -0.364

CASES EXCL. ( 0) ( 0) ( 0) ( 0) ( 0) ( 0)

CASES INCL. 2 3 4 2 2 7

---------------------------------------------------------------------------- ALL GROUPS COMBINED

| ANALYSIS OF VARIANCE TABLE FOR MEANS TAIL | (EXCEPT CASES WITH UNUSED

| SOURCE SUM OF SQUARES DF MEAN SQUARE F VALUE PROBABILITY | VALUES FOR VARIABLE pferd )

| -------- -------------- ---- ----------- ------- ----------- | MEAN 0.235

| pferd 0.2722 4 0.0681 2.10 0.1728 | STD. DEV. 0.210

| ERROR 0.2594 8 0.0324 | S. E. M. 0.058

|--------------------------------------------------------------------------| MAXIMUM 0.853

| EQUALITY OF MEANS TESTS; VARIANCES ARE NOT ASSUMED TO BE EQUAL | MINIMUM 0.039

| WELCH 4, 3 1.94 0.3070 | CASES EXCLUDED ( 7)

| BROWN-FORSYTHE 4, 1 1.38 0.5580 | CASES INCLUDED 13

|--------------------------------------------------------------------------|

| LEVENE'S TEST FOR VARIANCES 4, 8 52.15 0.0000 | ROBUST S.D. 0.178

----------------------------------------------------------------------------

************ ************

HISTOGRAM OF * STizd * ( 392) GROUPED BY * pferd * ( 2)

************ ************

CASES WITH

UNUSED

VALUES FOR

*9 *10 *15 *16 *18 pferd

MIDPOINTS...................+...................+...................+...................+...................+...................+

1.44000)

1.26000) *

1.08000) *

0.90000) M

0.72000) * * *

0.54000) ** M**

0.36000) *

0.18000) N M*

0.00000)M* M

-0.18000) *

-0.36000)

-0.54000)

-0.72000) *

LEGEND FOR GROUP MEANS: M - MEAN COINCIDES WITH AN ASTERISK

N - MEAN DOES NOT COINCIDE WITH ANY ASTERISK

MEAN 0.043 0.907 0.248 -0.031 0.173 0.535

STD.DEV. 0.066 0.221 0.654 0.000 0.049 0.445

S. E. M. 0.047 0.128 0.327 0.000 0.035 0.168

MAXIMUM 0.090 1.114 0.679 -0.031 0.208 1.186

MINIMUM -0.003 0.674 -0.726 -0.031 0.138 -0.265

CASES EXCL. ( 0) ( 0) ( 0) ( 1) ( 0) ( 0)

CASES INCL. 2 3 4 1 2 7

---------------------------------------------------------------------------- ALL GROUPS COMBINED

| ANALYSIS OF VARIANCE TABLE FOR MEANS TAIL | (EXCEPT CASES WITH UNUSED

| SOURCE SUM OF SQUARES DF MEAN SQUARE F VALUE PROBABILITY | VALUES FOR VARIABLE pferd )

| -------- -------------- ---- ----------- ------- ----------- | MEAN 0.343

| pferd 1.3678 4 0.3419 1.72 0.2482 | STD. DEV. 0.501

| ERROR 1.3878 7 0.1983 | S. E. M. 0.144

|--------------------------------------------------------------------------| MAXIMUM 1.114

| EQUALITY OF MEANS TESTS; VARIANCES ARE NOT ASSUMED TO BE EQUAL | MINIMUM -0.726

| WELCH 4, 6 7.56 0.0159 | CASES EXCLUDED ( 8)

| BROWN-FORSYTHE 4, 4 4.18 0.0976 | CASES INCLUDED 12

|--------------------------------------------------------------------------|

| LEVENE'S TEST FOR VARIANCES 4, 7 2.32 0.1566 | ROBUST S.D. 0.520

----------------------------------------------------------------------------

************ ************

HISTOGRAM OF * STism * ( 393) GROUPED BY * pferd * ( 2)

************ ************

CASES WITH

UNUSED

VALUES FOR

*9 *10 *15 *16 *18 pferd

MIDPOINTS...................+...................+...................+...................+...................+...................+

1.35000) *

1.20000)

1.05000)

0.90000) *

0.75000)

0.60000) *

0.45000) * * **

0.30000)* N N M N

0.15000)M * * M * ***

0.00000)

-0.15000) *

-0.30000)

-0.45000) *

LEGEND FOR GROUP MEANS: M - MEAN COINCIDES WITH AN ASTERISK

N - MEAN DOES NOT COINCIDE WITH ANY ASTERISK

MEAN 0.209 0.366 0.331 0.193 0.253 0.337

STD.DEV. 0.127 0.251 0.453 0.000 0.078 0.512

S. E. M. 0.090 0.145 0.226 0.000 0.055 0.193

MAXIMUM 0.298 0.588 0.945 0.193 0.308 1.296

MINIMUM 0.119 0.093 -0.082 0.193 0.198 -0.403

CASES EXCL. ( 0) ( 0) ( 0) ( 1) ( 0) ( 0)

CASES INCL. 2 3 4 1 2 7

---------------------------------------------------------------------------- ALL GROUPS COMBINED

| ANALYSIS OF VARIANCE TABLE FOR MEANS TAIL | (EXCEPT CASES WITH UNUSED

| SOURCE SUM OF SQUARES DF MEAN SQUARE F VALUE PROBABILITY | VALUES FOR VARIABLE pferd )

| -------- -------------- ---- ----------- ------- ----------- | MEAN 0.295

| pferd 0.0494 4 0.0123 0.11 0.9738 | STD. DEV. 0.272

| ERROR 0.7632 7 0.1090 | S. E. M. 0.078

|--------------------------------------------------------------------------| MAXIMUM 0.945

| EQUALITY OF MEANS TESTS; VARIANCES ARE NOT ASSUMED TO BE EQUAL | MINIMUM -0.082

| WELCH 4, 6 0.18 0.9417 | CASES EXCLUDED ( 8)

| BROWN-FORSYTHE 4, 5 0.24 0.9018 | CASES INCLUDED 12

|--------------------------------------------------------------------------|

| LEVENE'S TEST FOR VARIANCES 4, 7 1.56 0.2847 | ROBUST S.D. 0.256

----------------------------------------------------------------------------

************ ************

HISTOGRAM OF * STizm * ( 394) GROUPED BY * pferd * ( 2)

************ ************

CASES WITH

UNUSED

VALUES FOR

*9 *10 *15 *16 *18 pferd

MIDPOINTS...................+...................+...................+...................+...................+...................+

1.62000) *

1.44000)

1.26000)

1.08000) *

0.90000) * *

0.72000) M *

0.54000) * N M

0.36000)* * M *

0.18000)N * N

0.00000)* * * *

-0.18000)

-0.36000)

-0.54000) *

LEGEND FOR GROUP MEANS: M - MEAN COINCIDES WITH AN ASTERISK

N - MEAN DOES NOT COINCIDE WITH ANY ASTERISK

MEAN 0.094 0.753 0.556 0.306 0.191 0.450

STD.DEV. 0.253 0.145 0.738 0.000 0.206 0.596

S. E. M. 0.179 0.084 0.369 0.000 0.146 0.243

MAXIMUM 0.273 0.902 1.645 0.306 0.337 1.131

MINIMUM -0.085 0.613 0.012 0.306 0.046 -0.452

CASES EXCL. ( 0) ( 0) ( 0) ( 1) ( 0) ( 1)

CASES INCL. 2 3 4 1 2 6

---------------------------------------------------------------------------- ALL GROUPS COMBINED

| ANALYSIS OF VARIANCE TABLE FOR MEANS TAIL | (EXCEPT CASES WITH UNUSED

| SOURCE SUM OF SQUARES DF MEAN SQUARE F VALUE PROBABILITY | VALUES FOR VARIABLE pferd )

| -------- -------------- ---- ----------- ------- ----------- | MEAN 0.447

| pferd 0.7284 4 0.1821 0.72 0.6071 | STD. DEV. 0.478

| ERROR 1.7807 7 0.2544 | S. E. M. 0.138

|--------------------------------------------------------------------------| MAXIMUM 1.645

| EQUALITY OF MEANS TESTS; VARIANCES ARE NOT ASSUMED TO BE EQUAL | MINIMUM -0.085

| WELCH 4, 5 3.20 0.1167 | CASES EXCLUDED ( 8)

| BROWN-FORSYTHE 4, 5 1.56 0.3153 | CASES INCLUDED 12

|--------------------------------------------------------------------------|

| LEVENE'S TEST FOR VARIANCES 4, 7 1.94 0.2079 | ROBUST S.D. 0.462

----------------------------------------------------------------------------

************ ************

HISTOGRAM OF * STidz * ( 395) GROUPED BY * pferd * ( 2)

************ ************

CASES WITH

UNUSED

VALUES FOR

*9 *10 *15 *16 *18 pferd

MIDPOINTS...................+...................+...................+...................+...................+...................+

-3.20000)

-3.30000) M

-3.40000)

-3.50000)

-3.60000)

-3.70000)

-3.80000) M

-3.90000)

-4.00000)

-4.10000)

-4.20000)

-4.30000)

-4.40000)M

LEGEND FOR GROUP MEANS: M - MEAN COINCIDES WITH AN ASTERISK

N - MEAN DOES NOT COINCIDE WITH ANY ASTERISK

MEAN -4.395 0.000 0.000 -3.270 0.000 -3.785

STD.DEV. 0.000 0.000 0.000 0.000 0.000 0.000

S. E. M. 0.000 0.000 0.000 0.000 0.000 0.000

MAXIMUM -4.395 0.000 0.000 -3.270 0.000 -3.785

MINIMUM -4.395 0.000 0.000 -3.270 0.000 -3.785

CASES EXCL. ( 1) ( 3) ( 4) ( 1) ( 2) ( 6)

CASES INCL. 1 0 0 1 0 1

*** WARNING *** AN ANOVA WILL NOT BE COMPUTED SINCE THERE IS AT MOST ONE GROUP WITH VARIANCE GREATER THAN ZERO. THIS RESTRICTION

ALSO APPLIES WHEN THERE IS ONLY ONE CASE PER GROUP.

************ ************

HISTOGRAM OF * STid * ( 396) GROUPED BY * pferd * ( 2)

************ ************

CASES WITH

UNUSED

VALUES FOR

*9 *10 *15 *16 *18 pferd

MIDPOINTS...................+...................+...................+...................+...................+...................+

1.40000) *

1.05000)

0.70000) *

0.35000) * * *

0.00000)* * M

-0.35000)M N * N

-0.70000) * M

-1.05000) N *

-1.40000) *

-1.75000) **

-2.10000)

-2.45000)

-2.80000) *

LEGEND FOR GROUP MEANS: M - MEAN COINCIDES WITH AN ASTERISK

N - MEAN DOES NOT COINCIDE WITH ANY ASTERISK

MEAN -0.263 -0.389 -0.944 -0.789 0.138 -0.311

STD.DEV. 0.264 0.640 1.342 0.000 0.350 1.274

S. E. M. 0.187 0.453 0.671 0.000 0.247 0.520

MAXIMUM -0.076 0.064 0.476 -0.789 0.386 1.402

MINIMUM -0.449 -0.842 -2.645 -0.789 -0.109 -1.635

CASES EXCL. ( 0) ( 1) ( 0) ( 1) ( 0) ( 1)

CASES INCL. 2 2 4 1 2 6

---------------------------------------------------------------------------- ALL GROUPS COMBINED

| ANALYSIS OF VARIANCE TABLE FOR MEANS TAIL | (EXCEPT CASES WITH UNUSED

| SOURCE SUM OF SQUARES DF MEAN SQUARE F VALUE PROBABILITY | VALUES FOR VARIABLE pferd )

| -------- -------------- ---- ----------- ------- ----------- | MEAN -0.508

| pferd 1.8235 4 0.4559 0.46 0.7666 | STD. DEV. 0.885

| ERROR 6.0024 6 1.0004 | S. E. M. 0.267

|--------------------------------------------------------------------------| MAXIMUM 0.476

| EQUALITY OF MEANS TESTS; VARIANCES ARE NOT ASSUMED TO BE EQUAL | MINIMUM -2.645

| WELCH 4, 4 0.58 0.6951 | CASES EXCLUDED ( 9)

| BROWN-FORSYTHE 4, 5 1.11 0.4432 | CASES INCLUDED 11

|--------------------------------------------------------------------------|

| LEVENE'S TEST FOR VARIANCES 4, 6 1.95 0.2209 | ROBUST S.D. 0.841

----------------------------------------------------------------------------

************ ************

HISTOGRAM OF * STges * ( 397) GROUPED BY * pferd * ( 2)

************ ************

CASES WITH

UNUSED

VALUES FOR

*9 *10 *15 *16 *18 pferd

MIDPOINTS...................+...................+...................+...................+...................+...................+

8.00000) *

7.00000) * *

6.00000) * M ***

5.00000) * M

4.00000)

3.00000)* * M* *

2.00000)M N * M

1.00000) *

0.00000)

-1.00000)

-2.00000)

-3.00000)

-4.00000) *

LEGEND FOR GROUP MEANS: M - MEAN COINCIDES WITH AN ASTERISK

N - MEAN DOES NOT COINCIDE WITH ANY ASTERISK

MEAN 2.302 2.175 5.808 2.249 3.155 4.940

STD.DEV. 0.886 5.539 2.303 0.485 0.007 2.013

S. E. M. 0.627 3.198 1.152 0.343 0.005 0.761

MAXIMUM 2.928 5.533 7.571 2.592 3.160 7.360

MINIMUM 1.675 -4.219 2.476 1.906 3.149 1.452

CASES EXCL. ( 0) ( 0) ( 0) ( 0) ( 0) ( 0)

CASES INCL. 2 3 4 2 2 7

---------------------------------------------------------------------------- ALL GROUPS COMBINED

| ANALYSIS OF VARIANCE TABLE FOR MEANS TAIL | (EXCEPT CASES WITH UNUSED

| SOURCE SUM OF SQUARES DF MEAN SQUARE F VALUE PROBABILITY | VALUES FOR VARIABLE pferd )

| -------- -------------- ---- ----------- ------- ----------- | MEAN 3.474

| pferd 32.8027 4 8.2007 0.84 0.5380 | STD. DEV. 3.043

| ERROR 78.3025 8 9.7878 | S. E. M. 0.844

|--------------------------------------------------------------------------| MAXIMUM 7.571

| EQUALITY OF MEANS TESTS; VARIANCES ARE NOT ASSUMED TO BE EQUAL | MINIMUM -4.219

| WELCH 4, 3 2.08 0.2863 | CASES EXCLUDED ( 7)

| BROWN-FORSYTHE 4, 3 1.17 0.4685 | CASES INCLUDED 13

|--------------------------------------------------------------------------|

| LEVENE'S TEST FOR VARIANCES 4, 8 5.38 0.0211 | ROBUST S.D. 2.835

----------------------------------------------------------------------------

************ ************

HISTOGRAM OF * STsges * ( 398) GROUPED BY * pferd * ( 2)

************ ************

CASES WITH

UNUSED

VALUES FOR

*9 *10 *15 *16 *18 pferd

MIDPOINTS...................+...................+...................+...................+...................+...................+

3.00000)

2.70000) *

2.40000) **

2.10000)

1.80000) * *

1.50000) M* M

1.20000) N * M***

0.90000)* N *

0.60000)M * *

0.30000)

0.00000) *

-0.30000) *

-0.60000)

LEGEND FOR GROUP MEANS: M - MEAN COINCIDES WITH AN ASTERISK

N - MEAN DOES NOT COINCIDE WITH ANY ASTERISK

MEAN 0.644 1.595 1.484 1.159 0.922 1.205

STD.DEV. 0.237 0.161 1.279 0.915 0.308 0.804

S. E. M. 0.167 0.093 0.640 0.647 0.218 0.304

MAXIMUM 0.811 1.776 2.505 1.805 1.140 2.769

MINIMUM 0.476 1.466 -0.281 0.512 0.705 0.087

CASES EXCL. ( 0) ( 0) ( 0) ( 0) ( 0) ( 0)

CASES INCL. 2 3 4 2 2 7

---------------------------------------------------------------------------- ALL GROUPS COMBINED

| ANALYSIS OF VARIANCE TABLE FOR MEANS TAIL | (EXCEPT CASES WITH UNUSED

| SOURCE SUM OF SQUARES DF MEAN SQUARE F VALUE PROBABILITY | VALUES FOR VARIABLE pferd )

| -------- -------------- ---- ----------- ------- ----------- | MEAN 1.244

| pferd 1.5431 4 0.3858 0.52 0.7250 | STD. DEV. 0.790

| ERROR 5.9494 8 0.7437 | S. E. M. 0.219

|--------------------------------------------------------------------------| MAXIMUM 2.505

| EQUALITY OF MEANS TESTS; VARIANCES ARE NOT ASSUMED TO BE EQUAL | MINIMUM -0.281

| WELCH 4, 3 4.22 0.1334 | CASES EXCLUDED ( 7)

| BROWN-FORSYTHE 4, 4 0.78 0.5941 | CASES INCLUDED 13

|--------------------------------------------------------------------------|

| LEVENE'S TEST FOR VARIANCES 4, 8 2.34 0.1422 | ROBUST S.D. 0.823

----------------------------------------------------------------------------

************ ************

HISTOGRAM OF * STdz_sb * ( 399) GROUPED BY * pferd * ( 2)

************ ************

CASES WITH

UNUSED

VALUES FOR

*9 *10 *15 *16 *18 pferd

MIDPOINTS...................+...................+...................+...................+...................+...................+

2.40000) * ***

1.60000) * * N

0.80000) M M* **

0.00000)M* ** * **

-0.80000) N

-1.60000) N

-2.40000) *

-3.20000)

-4.00000)

-4.80000)

-5.60000)

-6.40000)

-7.20000) *

LEGEND FOR GROUP MEANS: M - MEAN COINCIDES WITH AN ASTERISK

N - MEAN DOES NOT COINCIDE WITH ANY ASTERISK

MEAN 0.094 -1.007 0.553 -1.359 0.956 1.254

STD.DEV. 0.013 5.186 0.749 1.409 0.224 1.008

S. E. M. 0.009 2.994 0.375 0.996 0.158 0.381

MAXIMUM 0.104 2.187 1.453 -0.363 1.115 2.454

MINIMUM 0.085 -6.990 -0.326 -2.355 0.798 0.095

CASES EXCL. ( 0) ( 0) ( 0) ( 0) ( 0) ( 0)

CASES INCL. 2 3 4 2 2 7

---------------------------------------------------------------------------- ALL GROUPS COMBINED

| ANALYSIS OF VARIANCE TABLE FOR MEANS TAIL | (EXCEPT CASES WITH UNUSED

| SOURCE SUM OF SQUARES DF MEAN SQUARE F VALUE PROBABILITY | VALUES FOR VARIABLE pferd )

| -------- -------------- ---- ----------- ------- ----------- | MEAN -0.110

| pferd 9.6491 4 2.4123 0.34 0.8467 | STD. DEV. 2.365

| ERROR 57.4976 8 7.1872 | S. E. M. 0.656

|--------------------------------------------------------------------------| MAXIMUM 2.187

| EQUALITY OF MEANS TESTS; VARIANCES ARE NOT ASSUMED TO BE EQUAL | MINIMUM -6.990

| WELCH 4, 3 4.88 0.1117 | CASES EXCLUDED ( 7)

| BROWN-FORSYTHE 4, 2 0.42 0.7898 | CASES INCLUDED 13

|--------------------------------------------------------------------------|

| LEVENE'S TEST FOR VARIANCES 4, 8 9.25 0.0043 | ROBUST S.D. 1.926

----------------------------------------------------------------------------

************ ************

HISTOGRAM OF * STi_zb * ( 400) GROUPED BY * pferd * ( 2)

************ ************

CASES WITH

UNUSED

VALUES FOR

*9 *10 *15 *16 *18 pferd

MIDPOINTS...................+...................+...................+...................+...................+...................+

3.50000)

3.15000) **

2.80000)

2.45000) * *

2.10000) M *

1.75000) N

1.40000)* * *

1.05000) M*

0.70000)N **

0.35000) M M* *

0.00000)*

-0.35000)

-0.70000) *

LEGEND FOR GROUP MEANS: M - MEAN COINCIDES WITH AN ASTERISK

N - MEAN DOES NOT COINCIDE WITH ANY ASTERISK

MEAN 0.683 1.953 1.919 0.276 0.364 1.114

STD.DEV. 1.092 0.379 1.463 0.000 0.157 1.057

S. E. M. 0.772 0.219 0.732 0.000 0.111 0.400

MAXIMUM 1.455 2.297 3.299 0.276 0.475 2.318

MINIMUM -0.089 1.547 0.538 0.276 0.254 -0.716

CASES EXCL. ( 0) ( 0) ( 0) ( 1) ( 0) ( 0)

CASES INCL. 2 3 4 1 2 7

---------------------------------------------------------------------------- ALL GROUPS COMBINED

| ANALYSIS OF VARIANCE TABLE FOR MEANS TAIL | (EXCEPT CASES WITH UNUSED

| SOURCE SUM OF SQUARES DF MEAN SQUARE F VALUE PROBABILITY | VALUES FOR VARIABLE pferd )

| -------- -------------- ---- ----------- ------- ----------- | MEAN 1.326

| pferd 6.3649 4 1.5912 1.41 0.3251 | STD. DEV. 1.140

| ERROR 7.9258 7 1.1323 | S. E. M. 0.329

|--------------------------------------------------------------------------| MAXIMUM 3.299

| EQUALITY OF MEANS TESTS; VARIANCES ARE NOT ASSUMED TO BE EQUAL | MINIMUM -0.089

| WELCH 4, 5 7.91 0.0217 | CASES EXCLUDED ( 8)

| BROWN-FORSYTHE 4, 4 2.50 0.1985 | CASES INCLUDED 12

|--------------------------------------------------------------------------|

| LEVENE'S TEST FOR VARIANCES 4, 7 41.99 0.0001 | ROBUST S.D. 1.249

----------------------------------------------------------------------------

************ ************

HISTOGRAM OF * STinf * ( 401) GROUPED BY * pferd * ( 2)

************ ************

CASES WITH

UNUSED

VALUES FOR

*9 *10 *15 *16 *18 pferd

MIDPOINTS...................+...................+...................+...................+...................+...................+

6.00000)

5.40000) *

4.80000) *

4.20000) * *

3.60000) * N *

3.00000) N

2.40000)* **

1.80000) M**

1.20000)N *

0.60000) M* M* *

0.00000)*

-0.60000)

-1.20000) *

LEGEND FOR GROUP MEANS: M - MEAN COINCIDES WITH AN ASTERISK

N - MEAN DOES NOT COINCIDE WITH ANY ASTERISK

MEAN 1.228 2.779 3.830 0.767 0.799 1.718

STD.DEV. 1.639 0.866 1.959 0.121 0.027 1.898

S. E. M. 1.159 0.500 0.980 0.086 0.019 0.718

MAXIMUM 2.387 3.759 5.159 0.853 0.818 4.244

MINIMUM 0.070 2.116 0.952 0.681 0.780 -1.483

CASES EXCL. ( 0) ( 0) ( 0) ( 0) ( 0) ( 0)

CASES INCL. 2 3 4 2 2 7

---------------------------------------------------------------------------- ALL GROUPS COMBINED

| ANALYSIS OF VARIANCE TABLE FOR MEANS TAIL | (EXCEPT CASES WITH UNUSED

| SOURCE SUM OF SQUARES DF MEAN SQUARE F VALUE PROBABILITY | VALUES FOR VARIABLE pferd )

| -------- -------------- ---- ----------- ------- ----------- | MEAN 2.250

| pferd 21.5202 4 5.3800 2.74 0.1051 | STD. DEV. 1.762

| ERROR 15.7201 8 1.9650 | S. E. M. 0.489

|--------------------------------------------------------------------------| MAXIMUM 5.159

| EQUALITY OF MEANS TESTS; VARIANCES ARE NOT ASSUMED TO BE EQUAL | MINIMUM 0.070

| WELCH 4, 3 3.78 0.1516 | CASES EXCLUDED ( 7)

| BROWN-FORSYTHE 4, 4 3.90 0.1080 | CASES INCLUDED 13

|--------------------------------------------------------------------------|

| LEVENE'S TEST FOR VARIANCES 4, 8 2.43 0.1329 | ROBUST S.D. 1.902

----------------------------------------------------------------------------

************ ************

HISTOGRAM OF * STps_rel * ( 402) GROUPED BY * pferd * ( 2)

************ ************

CASES WITH

UNUSED

VALUES FOR

*9 *10 *15 *16 *18 pferd

MIDPOINTS...................+...................+...................+...................+...................+...................+

0.300000)

0.250000) *

0.200000)

0.150000)

0.100000) *

0.050000)

0.000000) N *

-.050000)* * N M* **

-.100000)M * ** M*

-.150000) * N *

-.200000) **

-.250000)

-.300000) *

LEGEND FOR GROUP MEANS: M - MEAN COINCIDES WITH AN ASTERISK

N - MEAN DOES NOT COINCIDE WITH ANY ASTERISK

MEAN -0.080 -0.004 -0.144 -0.037 -0.046 -0.099

STD.DEV. 0.018 0.235 0.099 0.174 0.020 0.078

S. E. M. 0.013 0.136 0.049 0.123 0.014 0.029

MAXIMUM -0.067 0.265 -0.073 0.087 -0.032 0.014

MINIMUM -0.093 -0.166 -0.289 -0.160 -0.060 -0.205

CASES EXCL. ( 0) ( 0) ( 0) ( 0) ( 0) ( 0)

CASES INCL. 2 3 4 2 2 7

---------------------------------------------------------------------------- ALL GROUPS COMBINED

| ANALYSIS OF VARIANCE TABLE FOR MEANS TAIL | (EXCEPT CASES WITH UNUSED

| SOURCE SUM OF SQUARES DF MEAN SQUARE F VALUE PROBABILITY | VALUES FOR VARIABLE pferd )

| -------- -------------- ---- ----------- ------- ----------- | MEAN -0.070

| pferd 0.0384 4 0.0096 0.45 0.7698 | STD. DEV. 0.132

| ERROR 0.1705 8 0.0213 | S. E. M. 0.037

|--------------------------------------------------------------------------| MAXIMUM 0.265

| EQUALITY OF MEANS TESTS; VARIANCES ARE NOT ASSUMED TO BE EQUAL | MINIMUM -0.289

| WELCH 4, 3 0.95 0.5383 | CASES EXCLUDED ( 7)

| BROWN-FORSYTHE 4, 4 0.51 0.7350 | CASES INCLUDED 13

|--------------------------------------------------------------------------|

| LEVENE'S TEST FOR VARIANCES 4, 8 4.68 0.0305 | ROBUST S.D. 0.109

----------------------------------------------------------------------------

************ ************

HISTOGRAM OF * STs_rel * ( 403) GROUPED BY * pferd * ( 2)

************ ************

CASES WITH

UNUSED

VALUES FOR

*9 *10 *15 *16 *18 pferd

MIDPOINTS...................+...................+...................+...................+...................+...................+

1.50000)

1.35000) *

1.20000)

1.05000)

0.90000)

0.75000)

0.60000)

0.45000)

0.30000) N

0.15000) ** * *

0.00000)M* * N N M* M**

-0.15000) * * * ***

-0.30000) *

LEGEND FOR GROUP MEANS: M - MEAN COINCIDES WITH AN ASTERISK

N - MEAN DOES NOT COINCIDE WITH ANY ASTERISK

MEAN -0.033 0.371 -0.024 0.046 -0.005 -0.033

STD.DEV. 0.001 0.794 0.192 0.247 0.036 0.131

S. E. M. 0.001 0.458 0.096 0.175 0.025 0.050

MAXIMUM -0.032 1.287 0.150 0.221 0.021 0.205

MINIMUM -0.034 -0.113 -0.271 -0.128 -0.030 -0.175

CASES EXCL. ( 0) ( 0) ( 0) ( 0) ( 0) ( 0)

CASES INCL. 2 3 4 2 2 7

---------------------------------------------------------------------------- ALL GROUPS COMBINED

| ANALYSIS OF VARIANCE TABLE FOR MEANS TAIL | (EXCEPT CASES WITH UNUSED

| SOURCE SUM OF SQUARES DF MEAN SQUARE F VALUE PROBABILITY | VALUES FOR VARIABLE pferd )

| -------- -------------- ---- ----------- ------- ----------- | MEAN 0.080

| pferd 0.3397 4 0.0849 0.47 0.7541 | STD. DEV. 0.384

| ERROR 1.4319 8 0.1790 | S. E. M. 0.107

|--------------------------------------------------------------------------| MAXIMUM 1.287

| EQUALITY OF MEANS TESTS; VARIANCES ARE NOT ASSUMED TO BE EQUAL | MINIMUM -0.271

| WELCH 4, 3 0.32 0.8496 | CASES EXCLUDED ( 7)

| BROWN-FORSYTHE 4, 3 0.60 0.6881 | CASES INCLUDED 13

|--------------------------------------------------------------------------|

| LEVENE'S TEST FOR VARIANCES 4, 8 8.10 0.0065 | ROBUST S.D. 0.289

----------------------------------------------------------------------------

************ ************

HISTOGRAM OF * STpz_r * ( 404) GROUPED BY * pferd * ( 2)

************ ************

CASES WITH

UNUSED

VALUES FOR

*9 *10 *15 *16 *18 pferd

MIDPOINTS...................+...................+...................+...................+...................+...................+

1.05000)

0.98000) *

0.91000)

0.84000) *

0.77000) *

0.70000) M *

0.63000) N *

0.56000)

0.49000) * * *

0.42000)* * * M

0.35000)N N **

0.28000)* * M

0.21000) * *

LEGEND FOR GROUP MEANS: M - MEAN COINCIDES WITH AN ASTERISK

N - MEAN DOES NOT COINCIDE WITH ANY ASTERISK

MEAN 0.322 0.655 0.685 0.379 0.245 0.452

STD.DEV. 0.108 0.296 0.193 0.137 0.015 0.149

S. E. M. 0.077 0.171 0.096 0.097 0.011 0.056

MAXIMUM 0.398 0.994 0.835 0.475 0.256 0.666

MINIMUM 0.245 0.451 0.405 0.282 0.234 0.237

CASES EXCL. ( 0) ( 0) ( 0) ( 0) ( 0) ( 0)

CASES INCL. 2 3 4 2 2 7

---------------------------------------------------------------------------- ALL GROUPS COMBINED

| ANALYSIS OF VARIANCE TABLE FOR MEANS TAIL | (EXCEPT CASES WITH UNUSED

| SOURCE SUM OF SQUARES DF MEAN SQUARE F VALUE PROBABILITY | VALUES FOR VARIABLE pferd )

| -------- -------------- ---- ----------- ------- ----------- | MEAN 0.507

| pferd 0.4308 4 0.1077 2.72 0.1067 | STD. DEV. 0.250

| ERROR 0.3170 8 0.0396 | S. E. M. 0.069

|--------------------------------------------------------------------------| MAXIMUM 0.994

| EQUALITY OF MEANS TESTS; VARIANCES ARE NOT ASSUMED TO BE EQUAL | MINIMUM 0.234

| WELCH 4, 3 4.23 0.1327 | CASES EXCLUDED ( 7)

| BROWN-FORSYTHE 4, 5 3.62 0.0954 | CASES INCLUDED 13

|--------------------------------------------------------------------------|

| LEVENE'S TEST FOR VARIANCES 4, 8 2.36 0.1405 | ROBUST S.D. 0.263

----------------------------------------------------------------------------

************ ************

HISTOGRAM OF * STisd_r * ( 405) GROUPED BY * pferd * ( 2)

************ ************

CASES WITH

UNUSED

VALUES FOR

*9 *10 *15 *16 *18 pferd

MIDPOINTS...................+...................+...................+...................+...................+...................+

0.135000)

0.120000) *

0.105000) * *

0.090000) *

0.075000) N

0.060000)

0.045000) M

0.030000) *

0.015000) M N

0.000000)M* * M*** * **

-.015000) *

-.030000) *

-.045000) *

LEGEND FOR GROUP MEANS: M - MEAN COINCIDES WITH AN ASTERISK

N - MEAN DOES NOT COINCIDE WITH ANY ASTERISK

MEAN -0.005 0.050 0.002 0.072 0.011 0.016

STD.DEV. 0.000 0.049 0.003 0.060 0.015 0.062

S. E. M. 0.000 0.028 0.002 0.043 0.011 0.024

MAXIMUM -0.005 0.102 0.007 0.114 0.022 0.111

MINIMUM -0.005 0.004 -0.000 0.029 0.001 -0.045

CASES EXCL. ( 0) ( 0) ( 0) ( 0) ( 0) ( 0)

CASES INCL. 2 3 4 2 2 7

---------------------------------------------------------------------------- ALL GROUPS COMBINED

| ANALYSIS OF VARIANCE TABLE FOR MEANS TAIL | (EXCEPT CASES WITH UNUSED

| SOURCE SUM OF SQUARES DF MEAN SQUARE F VALUE PROBABILITY | VALUES FOR VARIABLE pferd )

| -------- -------------- ---- ----------- ------- ----------- | MEAN 0.024

| pferd 0.0107 4 0.0027 2.43 0.1329 | STD. DEV. 0.040

| ERROR 0.0088 8 0.0011 | S. E. M. 0.011

|--------------------------------------------------------------------------| MAXIMUM 0.114

| EQUALITY OF MEANS TESTS; VARIANCES ARE NOT ASSUMED TO BE EQUAL | MINIMUM -0.005

| WELCH 4, 3 4.10 0.1379 | CASES EXCLUDED ( 7)

| BROWN-FORSYTHE 4, 2 2.06 0.3519 | CASES INCLUDED 13

|--------------------------------------------------------------------------|

| LEVENE'S TEST FOR VARIANCES 4, 8 5.66 0.0184 | ROBUST S.D. 0.039

----------------------------------------------------------------------------

************ ************

HISTOGRAM OF * STizd_r * ( 406) GROUPED BY * pferd * ( 2)

************ ************

CASES WITH

UNUSED

VALUES FOR

*9 *10 *15 *16 *18 pferd

MIDPOINTS...................+...................+...................+...................+...................+...................+

0.245000)

0.210000) *

0.175000) M

0.140000) * *

0.105000) * *

0.070000) *

0.035000) ** M

0.000000) N M M **

-.035000)M* * *

-.070000)

-.105000)

-.140000)

-.175000) *

LEGEND FOR GROUP MEANS: M - MEAN COINCIDES WITH AN ASTERISK

N - MEAN DOES NOT COINCIDE WITH ANY ASTERISK

MEAN -0.021 0.173 -0.002 -0.010 -0.014 0.047

STD.DEV. 0.002 0.028 0.123 0.000 0.010 0.056

S. E. M. 0.002 0.016 0.061 0.000 0.007 0.021

MAXIMUM -0.020 0.204 0.090 -0.010 -0.007 0.139

MINIMUM -0.023 0.150 -0.183 -0.010 -0.022 -0.018

CASES EXCL. ( 0) ( 0) ( 0) ( 1) ( 0) ( 0)

CASES INCL. 2 3 4 1 2 7

---------------------------------------------------------------------------- ALL GROUPS COMBINED

| ANALYSIS OF VARIANCE TABLE FOR MEANS TAIL | (EXCEPT CASES WITH UNUSED

| SOURCE SUM OF SQUARES DF MEAN SQUARE F VALUE PROBABILITY | VALUES FOR VARIABLE pferd )

| -------- -------------- ---- ----------- ------- ----------- | MEAN 0.036

| pferd 0.0755 4 0.0189 2.81 0.1105 | STD. DEV. 0.106

| ERROR 0.0470 7 0.0067 | S. E. M. 0.030

|--------------------------------------------------------------------------| MAXIMUM 0.204

| EQUALITY OF MEANS TESTS; VARIANCES ARE NOT ASSUMED TO BE EQUAL | MINIMUM -0.183

| WELCH 4, 5 25.04 0.0017 | CASES EXCLUDED ( 8)

| BROWN-FORSYTHE 4, 3 7.02 0.0706 | CASES INCLUDED 12

|--------------------------------------------------------------------------|

| LEVENE'S TEST FOR VARIANCES 4, 7 2.44 0.1430 | ROBUST S.D. 0.105

----------------------------------------------------------------------------

************ ************

HISTOGRAM OF * STism_r * ( 407) GROUPED BY * pferd * ( 2)

************ ************

CASES WITH

UNUSED

VALUES FOR

*9 *10 *15 *16 *18 pferd

MIDPOINTS...................+...................+...................+...................+...................+...................+

0.225000)

0.200000) *

0.175000) *

0.150000)

0.125000) *

0.100000)

0.075000) N *

0.050000) * *

0.025000)* M M M N

0.000000)M * * * **

-.025000) * *

-.050000) *

-.075000)

LEGEND FOR GROUP MEANS: M - MEAN COINCIDES WITH AN ASTERISK

N - MEAN DOES NOT COINCIDE WITH ANY ASTERISK

MEAN 0.011 0.085 0.029 0.027 0.020 0.027

STD.DEV. 0.005 0.098 0.067 0.000 0.012 0.081

S. E. M. 0.004 0.057 0.033 0.000 0.008 0.030

MAXIMUM 0.015 0.193 0.127 0.027 0.028 0.184

MINIMUM 0.007 0.002 -0.022 0.027 0.012 -0.059

CASES EXCL. ( 0) ( 0) ( 0) ( 1) ( 0) ( 0)

CASES INCL. 2 3 4 1 2 7

---------------------------------------------------------------------------- ALL GROUPS COMBINED

| ANALYSIS OF VARIANCE TABLE FOR MEANS TAIL | (EXCEPT CASES WITH UNUSED

| SOURCE SUM OF SQUARES DF MEAN SQUARE F VALUE PROBABILITY | VALUES FOR VARIABLE pferd )

| -------- -------------- ---- ----------- ------- ----------- | MEAN 0.038

| pferd 0.0092 4 0.0023 0.49 0.7439 | STD. DEV. 0.062

| ERROR 0.0329 7 0.0047 | S. E. M. 0.018

|--------------------------------------------------------------------------| MAXIMUM 0.193

| EQUALITY OF MEANS TESTS; VARIANCES ARE NOT ASSUMED TO BE EQUAL | MINIMUM -0.022

| WELCH 4, 5 0.51 0.7327 | CASES EXCLUDED ( 8)

| BROWN-FORSYTHE 4, 4 0.89 0.5435 | CASES INCLUDED 12

|--------------------------------------------------------------------------|

| LEVENE'S TEST FOR VARIANCES 4, 7 2.21 0.1692 | ROBUST S.D. 0.058

----------------------------------------------------------------------------

************ ************

HISTOGRAM OF * STizm_r * ( 408) GROUPED BY * pferd * ( 2)

************ ************

CASES WITH

UNUSED

VALUES FOR

*9 *10 *15 *16 *18 pferd

MIDPOINTS...................+...................+...................+...................+...................+...................+

0.270000)

0.240000) *

0.210000) *

0.180000)

0.150000) N

0.120000) * **

0.090000) *

0.060000) N *

0.030000) * M N

0.000000)* * * *

-.030000)M M

-.060000) * **

-.090000)

LEGEND FOR GROUP MEANS: M - MEAN COINCIDES WITH AN ASTERISK

N - MEAN DOES NOT COINCIDE WITH ANY ASTERISK

MEAN -0.024 0.146 0.050 0.042 -0.017 0.030

STD.DEV. 0.018 0.081 0.107 0.000 0.026 0.085

S. E. M. 0.013 0.047 0.054 0.000 0.018 0.035

MAXIMUM -0.011 0.234 0.202 0.042 0.001 0.123

MINIMUM -0.037 0.075 -0.049 0.042 -0.035 -0.066

CASES EXCL. ( 0) ( 0) ( 0) ( 1) ( 0) ( 1)

CASES INCL. 2 3 4 1 2 6

---------------------------------------------------------------------------- ALL GROUPS COMBINED

| ANALYSIS OF VARIANCE TABLE FOR MEANS TAIL | (EXCEPT CASES WITH UNUSED

| SOURCE SUM OF SQUARES DF MEAN SQUARE F VALUE PROBABILITY | VALUES FOR VARIABLE pferd )

| -------- -------------- ---- ----------- ------- ----------- | MEAN 0.050

| pferd 0.0477 4 0.0119 1.71 0.2507 | STD. DEV. 0.094

| ERROR 0.0487 7 0.0070 | S. E. M. 0.027

|--------------------------------------------------------------------------| MAXIMUM 0.234

| EQUALITY OF MEANS TESTS; VARIANCES ARE NOT ASSUMED TO BE EQUAL | MINIMUM -0.049

| WELCH 4, 6 2.53 0.1483 | CASES EXCLUDED ( 8)

| BROWN-FORSYTHE 4, 6 3.55 0.0816 | CASES INCLUDED 12

|--------------------------------------------------------------------------|

| LEVENE'S TEST FOR VARIANCES 4, 7 1.24 0.3771 | ROBUST S.D. 0.096

----------------------------------------------------------------------------

************ ************

HISTOGRAM OF * STidz_r * ( 409) GROUPED BY * pferd * ( 2)

************ ************

CASES WITH

UNUSED

VALUES FOR

*9 *10 *15 *16 *18 pferd

MIDPOINTS...................+...................+...................+...................+...................+...................+

-0.56000)

-0.64000) M

-0.72000)

-0.80000)

-0.88000)

-0.96000)

-1.04000)

-1.12000)

-1.20000)M

-1.28000)

-1.36000)

-1.44000)

-1.52000) M

LEGEND FOR GROUP MEANS: M - MEAN COINCIDES WITH AN ASTERISK

N - MEAN DOES NOT COINCIDE WITH ANY ASTERISK

MEAN -1.231 0.000 0.000 -0.635 0.000 -1.550

STD.DEV. 0.000 0.000 0.000 0.000 0.000 0.000

S. E. M. 0.000 0.000 0.000 0.000 0.000 0.000

MAXIMUM -1.231 0.000 0.000 -0.635 0.000 -1.550

MINIMUM -1.231 0.000 0.000 -0.635 0.000 -1.550

CASES EXCL. ( 1) ( 3) ( 4) ( 1) ( 2) ( 6)

CASES INCL. 1 0 0 1 0 1

*** WARNING *** AN ANOVA WILL NOT BE COMPUTED SINCE THERE IS AT MOST ONE GROUP WITH VARIANCE GREATER THAN ZERO. THIS RESTRICTION

ALSO APPLIES WHEN THERE IS ONLY ONE CASE PER GROUP.

************ ************

HISTOGRAM OF * STid_r * ( 410) GROUPED BY * pferd * ( 2)

************ ************

CASES WITH

UNUSED

VALUES FOR

*9 *10 *15 *16 *18 pferd

MIDPOINTS...................+...................+...................+...................+...................+...................+

0.000000)

-.070000) *

-.140000)* M *

-.210000) N *

-.280000)N *

-.350000) M* * *

-.420000)* N M*

-.490000)

-.560000) * *

-.630000) *

-.700000)

-.770000)

-.840000) *

LEGEND FOR GROUP MEANS: M - MEAN COINCIDES WITH AN ASTERISK

N - MEAN DOES NOT COINCIDE WITH ANY ASTERISK

MEAN -0.285 -0.339 -0.445 -0.165 -0.212 -0.430

STD.DEV. 0.225 0.027 0.347 0.000 0.071 0.170

S. E. M. 0.159 0.019 0.174 0.000 0.050 0.070

MAXIMUM -0.126 -0.320 -0.039 -0.165 -0.162 -0.184

MINIMUM -0.444 -0.358 -0.870 -0.165 -0.263 -0.660

CASES EXCL. ( 0) ( 1) ( 0) ( 1) ( 0) ( 1)

CASES INCL. 2 2 4 1 2 6

---------------------------------------------------------------------------- ALL GROUPS COMBINED

| ANALYSIS OF VARIANCE TABLE FOR MEANS TAIL | (EXCEPT CASES WITH UNUSED

| SOURCE SUM OF SQUARES DF MEAN SQUARE F VALUE PROBABILITY | VALUES FOR VARIABLE pferd )

| -------- -------------- ---- ----------- ------- ----------- | MEAN -0.329

| pferd 0.1122 4 0.0280 0.40 0.8009 | STD. DEV. 0.230

| ERROR 0.4177 6 0.0696 | S. E. M. 0.069

|--------------------------------------------------------------------------| MAXIMUM -0.039

| EQUALITY OF MEANS TESTS; VARIANCES ARE NOT ASSUMED TO BE EQUAL | MINIMUM -0.870

| WELCH 4, 4 1.00 0.5007 | CASES EXCLUDED ( 9)

| BROWN-FORSYTHE 4, 4 0.91 0.5337 | CASES INCLUDED 11

|--------------------------------------------------------------------------|

| LEVENE'S TEST FOR VARIANCES 4, 6 1.63 0.2816 | ROBUST S.D. 0.215

----------------------------------------------------------------------------

************ ************

HISTOGRAM OF * STsges_r * ( 411) GROUPED BY * pferd * ( 2)

************ ************

CASES WITH

UNUSED

VALUES FOR

*9 *10 *15 *16 *18 pferd

MIDPOINTS...................+...................+...................+...................+...................+...................+

0.900000)

0.800000) *

0.700000)

0.600000)

0.500000)

0.400000)

0.300000) N *

0.200000) * ** *

0.100000) * N N * **

0.000000)M* * * M M**

-.100000) *

-.200000) *

-.300000)

LEGEND FOR GROUP MEANS: M - MEAN COINCIDES WITH AN ASTERISK

N - MEAN DOES NOT COINCIDE WITH ANY ASTERISK

MEAN 0.025 0.330 0.054 0.122 0.043 0.034

STD.DEV. 0.012 0.384 0.136 0.136 0.048 0.158

S. E. M. 0.009 0.222 0.068 0.096 0.034 0.060

MAXIMUM 0.034 0.771 0.166 0.218 0.077 0.268

MINIMUM 0.017 0.069 -0.124 0.026 0.009 -0.237

CASES EXCL. ( 0) ( 0) ( 0) ( 0) ( 0) ( 0)

CASES INCL. 2 3 4 2 2 7

---------------------------------------------------------------------------- ALL GROUPS COMBINED

| ANALYSIS OF VARIANCE TABLE FOR MEANS TAIL | (EXCEPT CASES WITH UNUSED

| SOURCE SUM OF SQUARES DF MEAN SQUARE F VALUE PROBABILITY | VALUES FOR VARIABLE pferd )

| -------- -------------- ---- ----------- ------- ----------- | MEAN 0.122

| pferd 0.1801 4 0.0450 0.97 0.4738 | STD. DEV. 0.214

| ERROR 0.3711 8 0.0464 | S. E. M. 0.059

|--------------------------------------------------------------------------| MAXIMUM 0.771

| EQUALITY OF MEANS TESTS; VARIANCES ARE NOT ASSUMED TO BE EQUAL | MINIMUM -0.124

| WELCH 4, 3 0.48 0.7526 | CASES EXCLUDED ( 7)

| BROWN-FORSYTHE 4, 3 1.25 0.4443 | CASES INCLUDED 13

|--------------------------------------------------------------------------|

| LEVENE'S TEST FOR VARIANCES 4, 8 5.74 0.0177 | ROBUST S.D. 0.171

----------------------------------------------------------------------------

************ ************

HISTOGRAM OF * STi_zb_r * ( 412) GROUPED BY * pferd * ( 2)

************ ************

CASES WITH

UNUSED

VALUES FOR

*9 *10 *15 *16 *18 pferd

MIDPOINTS...................+...................+...................+...................+...................+...................+

0.880000)

0.800000) *

0.720000)

0.640000)

0.560000)

0.480000)

0.400000) N

0.320000) * *

0.240000) * *

0.160000) * *

0.080000)* M M

0.000000)N ** M M *

-.080000)* * **

LEGEND FOR GROUP MEANS: M - MEAN COINCIDES WITH AN ASTERISK

N - MEAN DOES NOT COINCIDE WITH ANY ASTERISK

MEAN 0.012 0.437 0.048 0.032 -0.031 0.100

STD.DEV. 0.096 0.311 0.060 0.000 0.015 0.149

S. E. M. 0.068 0.179 0.030 0.000 0.011 0.056

MAXIMUM 0.080 0.794 0.124 0.032 -0.021 0.281

MINIMUM -0.056 0.226 -0.015 0.032 -0.042 -0.082

CASES EXCL. ( 0) ( 0) ( 0) ( 1) ( 0) ( 0)

CASES INCL. 2 3 4 1 2 7

---------------------------------------------------------------------------- ALL GROUPS COMBINED

| ANALYSIS OF VARIANCE TABLE FOR MEANS TAIL | (EXCEPT CASES WITH UNUSED

| SOURCE SUM OF SQUARES DF MEAN SQUARE F VALUE PROBABILITY | VALUES FOR VARIABLE pferd )

| -------- -------------- ---- ----------- ------- ----------- | MEAN 0.125

| pferd 0.3992 4 0.0998 3.27 0.0819 | STD. DEV. 0.236

| ERROR 0.2135 7 0.0305 | S. E. M. 0.068

|--------------------------------------------------------------------------| MAXIMUM 0.794

| EQUALITY OF MEANS TESTS; VARIANCES ARE NOT ASSUMED TO BE EQUAL | MINIMUM -0.056

| WELCH 4, 5 2.28 0.1957 | CASES EXCLUDED ( 8)

| BROWN-FORSYTHE 4, 3 4.82 0.1135 | CASES INCLUDED 12

|--------------------------------------------------------------------------|

| LEVENE'S TEST FOR VARIANCES 4, 7 6.46 0.0168 | ROBUST S.D. 0.204

----------------------------------------------------------------------------

************ ************

HISTOGRAM OF * STinf_r * ( 413) GROUPED BY * pferd * ( 2)

************ ************

CASES WITH

UNUSED

VALUES FOR

*9 *10 *15 *16 *18 pferd

MIDPOINTS...................+...................+...................+...................+...................+...................+

1.50000)

1.35000) *

1.20000)

1.05000)

0.90000)

0.75000)

0.60000) N *

0.45000) *

0.30000) ** *

0.15000)* M* M* N

0.00000)M ** M* **

-0.15000) **

-0.30000)

LEGEND FOR GROUP MEANS: M - MEAN COINCIDES WITH AN ASTERISK

N - MEAN DOES NOT COINCIDE WITH ANY ASTERISK

MEAN 0.047 0.643 0.079 0.101 -0.000 0.142

STD.DEV. 0.143 0.570 0.062 0.019 0.012 0.275

S. E. M. 0.101 0.329 0.031 0.014 0.008 0.104

MAXIMUM 0.148 1.300 0.146 0.114 0.008 0.570

MINIMUM -0.054 0.289 0.003 0.087 -0.008 -0.186

CASES EXCL. ( 0) ( 0) ( 0) ( 0) ( 0) ( 0)

CASES INCL. 2 3 4 2 2 7

---------------------------------------------------------------------------- ALL GROUPS COMBINED

| ANALYSIS OF VARIANCE TABLE FOR MEANS TAIL | (EXCEPT CASES WITH UNUSED

| SOURCE SUM OF SQUARES DF MEAN SQUARE F VALUE PROBABILITY | VALUES FOR VARIABLE pferd )

| -------- -------------- ---- ----------- ------- ----------- | MEAN 0.195

| pferd 0.7933 4 0.1983 2.33 0.1438 | STD. DEV. 0.351

| ERROR 0.6818 8 0.0852 | S. E. M. 0.097

|--------------------------------------------------------------------------| MAXIMUM 1.300

| EQUALITY OF MEANS TESTS; VARIANCES ARE NOT ASSUMED TO BE EQUAL | MINIMUM -0.054

| WELCH 4, 3 7.27 0.0675 | CASES EXCLUDED ( 7)

| BROWN-FORSYTHE 4, 2 2.94 0.2698 | CASES INCLUDED 13

|--------------------------------------------------------------------------|

| LEVENE'S TEST FOR VARIANCES 4, 8 9.79 0.0036 | ROBUST S.D. 0.269

----------------------------------------------------------------------------

************ ************

HISTOGRAM OF * STdges * ( 414) GROUPED BY * pferd * ( 2)

************ ************

CASES WITH

UNUSED

VALUES FOR

*9 *10 *15 *16 *18 pferd

MIDPOINTS...................+...................+...................+...................+...................+...................+

2.40000) * *

1.60000) * * * M**

0.80000) M* * M **

0.00000)M* *

-0.80000) N * N

-1.60000)

-2.40000) *

-3.20000)

-4.00000)

-4.80000)

-5.60000)

-6.40000)

-7.20000) *

LEGEND FOR GROUP MEANS: M - MEAN COINCIDES WITH AN ASTERISK

N - MEAN DOES NOT COINCIDE WITH ANY ASTERISK

MEAN -0.027 -0.864 0.578 -1.110 1.134 1.384

STD.DEV. 0.012 5.239 0.946 2.330 0.095 0.787

S. E. M. 0.008 3.025 0.473 1.648 0.067 0.297

MAXIMUM -0.019 2.388 1.380 0.538 1.201 2.568

MINIMUM -0.035 -6.908 -0.783 -2.757 1.067 0.270

CASES EXCL. ( 0) ( 0) ( 0) ( 0) ( 0) ( 0)

CASES INCL. 2 3 4 2 2 7

---------------------------------------------------------------------------- ALL GROUPS COMBINED

| ANALYSIS OF VARIANCE TABLE FOR MEANS TAIL | (EXCEPT CASES WITH UNUSED

| SOURCE SUM OF SQUARES DF MEAN SQUARE F VALUE PROBABILITY | VALUES FOR VARIABLE pferd )

| -------- -------------- ---- ----------- ------- ----------- | MEAN -0.022

| pferd 8.6112 4 2.1528 0.27 0.8872 | STD. DEV. 2.443

| ERROR 63.0206 8 7.8776 | S. E. M. 0.678

|--------------------------------------------------------------------------| MAXIMUM 2.388

| EQUALITY OF MEANS TESTS; VARIANCES ARE NOT ASSUMED TO BE EQUAL | MINIMUM -6.908

| WELCH 4, 3 43.44 0.0055 | CASES EXCLUDED ( 7)

| BROWN-FORSYTHE 4, 3 0.33 0.8458 | CASES INCLUDED 13

|--------------------------------------------------------------------------|

| LEVENE'S TEST FOR VARIANCES 4, 8 8.64 0.0053 | ROBUST S.D. 2.086

----------------------------------------------------------------------------

************ ************

HISTOGRAM OF * STinfm * ( 415) GROUPED BY * pferd * ( 2)

************ ************

CASES WITH

UNUSED

VALUES FOR

*9 *10 *15 *16 *18 pferd

MIDPOINTS...................+...................+...................+...................+...................+...................+

2.70000) *

2.40000)

2.10000)

1.80000) *

1.50000)

1.20000) *

0.90000) * * **

0.60000)* N N M N

0.30000)M * * M * ***

0.00000)

-0.30000) *

-0.60000)

-0.90000) *

LEGEND FOR GROUP MEANS: M - MEAN COINCIDES WITH AN ASTERISK

N - MEAN DOES NOT COINCIDE WITH ANY ASTERISK

MEAN 0.418 0.732 0.663 0.385 0.505 0.674

STD.DEV. 0.254 0.502 0.905 0.000 0.155 1.023

S. E. M. 0.179 0.290 0.453 0.000 0.110 0.387

MAXIMUM 0.597 1.175 1.890 0.385 0.615 2.593

MINIMUM 0.238 0.186 -0.164 0.385 0.395 -0.805

CASES EXCL. ( 0) ( 0) ( 0) ( 1) ( 0) ( 0)

CASES INCL. 2 3 4 1 2 7

---------------------------------------------------------------------------- ALL GROUPS COMBINED

| ANALYSIS OF VARIANCE TABLE FOR MEANS TAIL | (EXCEPT CASES WITH UNUSED

| SOURCE SUM OF SQUARES DF MEAN SQUARE F VALUE PROBABILITY | VALUES FOR VARIABLE pferd )

| -------- -------------- ---- ----------- ------- ----------- | MEAN 0.590

| pferd 0.1976 4 0.0494 0.11 0.9738 | STD. DEV. 0.544

| ERROR 3.0527 7 0.4361 | S. E. M. 0.157

|--------------------------------------------------------------------------| MAXIMUM 1.890

| EQUALITY OF MEANS TESTS; VARIANCES ARE NOT ASSUMED TO BE EQUAL | MINIMUM -0.164

| WELCH 4, 6 0.18 0.9417 | CASES EXCLUDED ( 8)

| BROWN-FORSYTHE 4, 5 0.24 0.9018 | CASES INCLUDED 12

|--------------------------------------------------------------------------|

| LEVENE'S TEST FOR VARIANCES 4, 7 1.56 0.2847 | ROBUST S.D. 0.511

----------------------------------------------------------------------------

************ ************

HISTOGRAM OF * STinfd * ( 416) GROUPED BY * pferd * ( 2)

************ ************

CASES WITH

UNUSED

VALUES FOR

*9 *10 *15 *16 *18 pferd

MIDPOINTS...................+...................+...................+...................+...................+...................+

2.00000)

1.75000) * **

1.50000)

1.25000)

1.00000) N

0.75000) **

0.50000) N ** * * M*

0.25000)M * M* M *

0.00000)* *

-0.25000)

-0.50000)

-0.75000) *

-1.00000)

LEGEND FOR GROUP MEANS: M - MEAN COINCIDES WITH AN ASTERISK

N - MEAN DOES NOT COINCIDE WITH ANY ASTERISK

MEAN 0.135 0.573 0.313 1.066 0.363 0.544

STD.DEV. 0.080 0.234 0.141 0.905 0.212 0.882

S. E. M. 0.057 0.135 0.070 0.640 0.150 0.333

MAXIMUM 0.191 0.711 0.481 1.706 0.513 1.699

MINIMUM 0.078 0.304 0.196 0.426 0.214 -0.728

CASES EXCL. ( 0) ( 0) ( 0) ( 0) ( 0) ( 0)

CASES INCL. 2 3 4 2 2 7

---------------------------------------------------------------------------- ALL GROUPS COMBINED

| ANALYSIS OF VARIANCE TABLE FOR MEANS TAIL | (EXCEPT CASES WITH UNUSED

| SOURCE SUM OF SQUARES DF MEAN SQUARE F VALUE PROBABILITY | VALUES FOR VARIABLE pferd )

| -------- -------------- ---- ----------- ------- ----------- | MEAN 0.469

| pferd 1.0890 4 0.2722 2.10 0.1728 | STD. DEV. 0.421

| ERROR 1.0377 8 0.1297 | S. E. M. 0.117

|--------------------------------------------------------------------------| MAXIMUM 1.706

| EQUALITY OF MEANS TESTS; VARIANCES ARE NOT ASSUMED TO BE EQUAL | MINIMUM 0.078

| WELCH 4, 3 1.94 0.3070 | CASES EXCLUDED ( 7)

| BROWN-FORSYTHE 4, 1 1.38 0.5580 | CASES INCLUDED 13

|--------------------------------------------------------------------------|

| LEVENE'S TEST FOR VARIANCES 4, 8 52.15 0.0000 | ROBUST S.D. 0.355

----------------------------------------------------------------------------

************ ************

HISTOGRAM OF * STpsr2 * ( 417) GROUPED BY * pferd * ( 2)

************ ************

CASES WITH

UNUSED

VALUES FOR

*9 *10 *15 *16 *18 pferd

MIDPOINTS...................+...................+...................+...................+...................+...................+

0.800000)

0.720000) *

0.640000)

0.560000)

0.480000)

0.400000)

0.320000)

0.240000)

0.160000) N * *

0.080000) ** N

0.000000)M* N * M* M**

-.080000) ** * ***

-.160000) *

LEGEND FOR GROUP MEANS: M - MEAN COINCIDES WITH AN ASTERISK

N - MEAN DOES NOT COINCIDE WITH ANY ASTERISK

MEAN -0.021 0.194 -0.014 0.085 -0.003 -0.019

STD.DEV. 0.000 0.436 0.124 0.097 0.024 0.088

S. E. M. 0.000 0.252 0.062 0.068 0.017 0.033

MAXIMUM -0.021 0.697 0.100 0.154 0.014 0.144

MINIMUM -0.021 -0.073 -0.172 0.017 -0.020 -0.111

CASES EXCL. ( 0) ( 0) ( 0) ( 0) ( 0) ( 0)

CASES INCL. 2 3 4 2 2 7

---------------------------------------------------------------------------- ALL GROUPS COMBINED

| ANALYSIS OF VARIANCE TABLE FOR MEANS TAIL | (EXCEPT CASES WITH UNUSED

| SOURCE SUM OF SQUARES DF MEAN SQUARE F VALUE PROBABILITY | VALUES FOR VARIABLE pferd )

| -------- -------------- ---- ----------- ------- ----------- | MEAN 0.050

| pferd 0.0971 4 0.0243 0.45 0.7731 | STD. DEV. 0.211

| ERROR 0.4355 8 0.0544 | S. E. M. 0.058

|--------------------------------------------------------------------------| MAXIMUM 0.697

| EQUALITY OF MEANS TESTS; VARIANCES ARE NOT ASSUMED TO BE EQUAL | MINIMUM -0.172

| WELCH 4, 3 0.63 0.6771 | CASES EXCLUDED ( 7)

| BROWN-FORSYTHE 4, 3 0.59 0.6962 | CASES INCLUDED 13

|--------------------------------------------------------------------------|

| LEVENE'S TEST FOR VARIANCES 4, 8 7.76 0.0074 | ROBUST S.D. 0.164

----------------------------------------------------------------------------

************ ************

HISTOGRAM OF * STidr2 * ( 418) GROUPED BY * pferd * ( 2)

************ ************

CASES WITH

UNUSED

VALUES FOR

*9 *10 *15 *16 *18 pferd

MIDPOINTS...................+...................+...................+...................+...................+...................+

0.240000)

0.180000) *

0.120000)

0.060000) *

0.000000)*

-.060000)N M *

-.120000)* M* M *

-.180000) *

-.240000) * M

-.300000) M

-.360000)

-.420000) * *

-.480000) *

LEGEND FOR GROUP MEANS: M - MEAN COINCIDES WITH AN ASTERISK

N - MEAN DOES NOT COINCIDE WITH ANY ASTERISK

MEAN -0.045 -0.290 -0.116 -0.113 -0.078 -0.220

STD.DEV. 0.094 0.045 0.234 0.000 0.044 0.203

S. E. M. 0.067 0.032 0.117 0.000 0.031 0.083

MAXIMUM 0.022 -0.258 0.170 -0.113 -0.047 0.056

MINIMUM -0.112 -0.321 -0.402 -0.113 -0.109 -0.454

CASES EXCL. ( 0) ( 1) ( 0) ( 1) ( 0) ( 1)

CASES INCL. 2 2 4 1 2 6

---------------------------------------------------------------------------- ALL GROUPS COMBINED

| ANALYSIS OF VARIANCE TABLE FOR MEANS TAIL | (EXCEPT CASES WITH UNUSED

| SOURCE SUM OF SQUARES DF MEAN SQUARE F VALUE PROBABILITY | VALUES FOR VARIABLE pferd )

| -------- -------------- ---- ----------- ------- ----------- | MEAN -0.127

| pferd 0.0719 4 0.0180 0.61 0.6712 | STD. DEV. 0.158

| ERROR 0.1770 6 0.0295 | S. E. M. 0.048

|--------------------------------------------------------------------------| MAXIMUM 0.170

| EQUALITY OF MEANS TESTS; VARIANCES ARE NOT ASSUMED TO BE EQUAL | MINIMUM -0.402

| WELCH 4, 5 4.61 0.0623 | CASES EXCLUDED ( 9)

| BROWN-FORSYTHE 4, 4 1.59 0.3330 | CASES INCLUDED 11

|--------------------------------------------------------------------------|

| LEVENE'S TEST FOR VARIANCES 4, 6 0.80 0.5693 | ROBUST S.D. 0.145

----------------------------------------------------------------------------

************ ************

HISTOGRAM OF * STisdr2 * ( 419) GROUPED BY * pferd * ( 2)

************ ************

CASES WITH

UNUSED

VALUES FOR

*9 *10 *15 *16 *18 pferd

MIDPOINTS...................+...................+...................+...................+...................+...................+

0.160000) * *

0.140000) *

0.120000)

0.100000) * N

0.080000)

0.060000) M

0.040000) M* * * N

0.020000)M* * ** N ***

0.000000) *

-.020000) *

-.040000)

-.060000)

-.080000) *

LEGEND FOR GROUP MEANS: M - MEAN COINCIDES WITH AN ASTERISK

N - MEAN DOES NOT COINCIDE WITH ANY ASTERISK

MEAN 0.015 0.057 0.033 0.097 0.023 0.036

STD.DEV. 0.006 0.036 0.011 0.080 0.019 0.080

S. E. M. 0.004 0.021 0.006 0.057 0.013 0.030

MAXIMUM 0.019 0.090 0.043 0.153 0.037 0.150

MINIMUM 0.011 0.018 0.022 0.040 0.010 -0.078

CASES EXCL. ( 0) ( 0) ( 0) ( 0) ( 0) ( 0)

CASES INCL. 2 3 4 2 2 7

---------------------------------------------------------------------------- ALL GROUPS COMBINED

| ANALYSIS OF VARIANCE TABLE FOR MEANS TAIL | (EXCEPT CASES WITH UNUSED

| SOURCE SUM OF SQUARES DF MEAN SQUARE F VALUE PROBABILITY | VALUES FOR VARIABLE pferd )

| -------- -------------- ---- ----------- ------- ----------- | MEAN 0.044

| pferd 0.0091 4 0.0023 1.85 0.2124 | STD. DEV. 0.040

| ERROR 0.0098 8 0.0012 | S. E. M. 0.011

|--------------------------------------------------------------------------| MAXIMUM 0.153

| EQUALITY OF MEANS TESTS; VARIANCES ARE NOT ASSUMED TO BE EQUAL | MINIMUM 0.010

| WELCH 4, 3 1.66 0.3524 | CASES EXCLUDED ( 7)

| BROWN-FORSYTHE 4, 2 1.33 0.4726 | CASES INCLUDED 13

|--------------------------------------------------------------------------|

| LEVENE'S TEST FOR VARIANCES 4, 8 12.36 0.0017 | ROBUST S.D. 0.035

----------------------------------------------------------------------------

************ ************

HISTOGRAM OF * STismr2 * ( 420) GROUPED BY * pferd * ( 2)

************ ************

CASES WITH

UNUSED

VALUES FOR

*9 *10 *15 *16 *18 pferd

MIDPOINTS...................+...................+...................+...................+...................+...................+

0.270000)

0.240000) * *

0.210000)

0.180000) *

0.150000)

0.120000) N

0.090000) * *

0.060000)* M M

0.030000)M * M M* *

0.000000) * * **

-.030000)

-.060000)

-.090000) *

LEGEND FOR GROUP MEANS: M - MEAN COINCIDES WITH AN ASTERISK

N - MEAN DOES NOT COINCIDE WITH ANY ASTERISK

MEAN 0.040 0.116 0.067 0.036 0.034 0.049

STD.DEV. 0.020 0.124 0.087 0.000 0.015 0.097

S. E. M. 0.014 0.072 0.043 0.000 0.010 0.037

MAXIMUM 0.054 0.253 0.188 0.036 0.044 0.226

MINIMUM 0.025 0.009 -0.009 0.036 0.024 -0.089

CASES EXCL. ( 0) ( 0) ( 0) ( 1) ( 0) ( 0)

CASES INCL. 2 3 4 1 2 7

---------------------------------------------------------------------------- ALL GROUPS COMBINED

| ANALYSIS OF VARIANCE TABLE FOR MEANS TAIL | (EXCEPT CASES WITH UNUSED

| SOURCE SUM OF SQUARES DF MEAN SQUARE F VALUE PROBABILITY | VALUES FOR VARIABLE pferd )

| -------- -------------- ---- ----------- ------- ----------- | MEAN 0.067

| pferd 0.0118 4 0.0030 0.38 0.8145 | STD. DEV. 0.077

| ERROR 0.0541 7 0.0077 | S. E. M. 0.022

|--------------------------------------------------------------------------| MAXIMUM 0.253

| EQUALITY OF MEANS TESTS; VARIANCES ARE NOT ASSUMED TO BE EQUAL | MINIMUM -0.009

| WELCH 4, 6 0.33 0.8483 | CASES EXCLUDED ( 8)

| BROWN-FORSYTHE 4, 4 0.69 0.6355 | CASES INCLUDED 12

|--------------------------------------------------------------------------|

| LEVENE'S TEST FOR VARIANCES 4, 7 1.75 0.2439 | ROBUST S.D. 0.072

----------------------------------------------------------------------------

************ ************

HISTOGRAM OF * STsgesr2 * ( 421) GROUPED BY * pferd * ( 2)

************ ************

CASES WITH

UNUSED

VALUES FOR

*9 *10 *15 *16 *18 pferd

MIDPOINTS...................+...................+...................+...................+...................+...................+

1.65000)

1.50000) *

1.35000)

1.20000)

1.05000)

0.90000)

0.75000)

0.60000) N

0.45000) ** *

0.30000) ** M M *

0.15000)M* * M M***

0.00000) * *

-0.15000) *

LEGEND FOR GROUP MEANS: M - MEAN COINCIDES WITH AN ASTERISK

N - MEAN DOES NOT COINCIDE WITH ANY ASTERISK

MEAN 0.125 0.675 0.268 0.245 0.111 0.168

STD.DEV. 0.043 0.711 0.204 0.104 0.057 0.167

S. E. M. 0.030 0.411 0.102 0.073 0.040 0.063

MAXIMUM 0.156 1.496 0.428 0.319 0.151 0.471

MINIMUM 0.095 0.233 -0.009 0.172 0.070 -0.077

CASES EXCL. ( 0) ( 0) ( 0) ( 0) ( 0) ( 0)

CASES INCL. 2 3 4 2 2 7

---------------------------------------------------------------------------- ALL GROUPS COMBINED

| ANALYSIS OF VARIANCE TABLE FOR MEANS TAIL | (EXCEPT CASES WITH UNUSED

| SOURCE SUM OF SQUARES DF MEAN SQUARE F VALUE PROBABILITY | VALUES FOR VARIABLE pferd )

| -------- -------------- ---- ----------- ------- ----------- | MEAN 0.312

| pferd 0.5626 4 0.1407 0.98 0.4718 | STD. DEV. 0.378

| ERROR 1.1535 8 0.1442 | S. E. M. 0.105

|--------------------------------------------------------------------------| MAXIMUM 1.496

| EQUALITY OF MEANS TESTS; VARIANCES ARE NOT ASSUMED TO BE EQUAL | MINIMUM -0.009

| WELCH 4, 3 0.96 0.5340 | CASES EXCLUDED ( 7)

| BROWN-FORSYTHE 4, 2 1.30 0.4777 | CASES INCLUDED 13

|--------------------------------------------------------------------------|

| LEVENE'S TEST FOR VARIANCES 4, 8 7.06 0.0098 | ROBUST S.D. 0.283

----------------------------------------------------------------------------

************ ************

HISTOGRAM OF * STinfr2 * ( 422) GROUPED BY * pferd * ( 2)

************ ************

CASES WITH

UNUSED

VALUES FOR

*9 *10 *15 *16 *18 pferd

MIDPOINTS...................+...................+...................+...................+...................+...................+

2.00000) *

1.80000)

1.60000)

1.40000)

1.20000)

1.00000) N **

0.80000) M *

0.60000) *

0.40000)* ** *

0.20000)N * M* M*

0.00000)* M* *

-0.20000)

-0.40000) *

LEGEND FOR GROUP MEANS: M - MEAN COINCIDES WITH AN ASTERISK

N - MEAN DOES NOT COINCIDE WITH ANY ASTERISK

MEAN 0.244 0.981 0.737 0.142 0.079 0.256

STD.DEV. 0.297 0.926 0.380 0.015 0.020 0.353

S. E. M. 0.210 0.535 0.190 0.011 0.014 0.133

MAXIMUM 0.454 2.051 1.004 0.153 0.093 0.754

MINIMUM 0.034 0.430 0.186 0.132 0.065 -0.319

CASES EXCL. ( 0) ( 0) ( 0) ( 0) ( 0) ( 0)

CASES INCL. 2 3 4 2 2 7

---------------------------------------------------------------------------- ALL GROUPS COMBINED

| ANALYSIS OF VARIANCE TABLE FOR MEANS TAIL | (EXCEPT CASES WITH UNUSED

| SOURCE SUM OF SQUARES DF MEAN SQUARE F VALUE PROBABILITY | VALUES FOR VARIABLE pferd )

| -------- -------------- ---- ----------- ------- ----------- | MEAN 0.525

| pferd 1.6531 4 0.4133 1.48 0.2954 | STD. DEV. 0.569

| ERROR 2.2377 8 0.2797 | S. E. M. 0.158

|--------------------------------------------------------------------------| MAXIMUM 2.051

| EQUALITY OF MEANS TESTS; VARIANCES ARE NOT ASSUMED TO BE EQUAL | MINIMUM 0.034

| WELCH 4, 3 4.08 0.1386 | CASES EXCLUDED ( 7)

| BROWN-FORSYTHE 4, 3 1.98 0.3006 | CASES INCLUDED 13

|--------------------------------------------------------------------------|

| LEVENE'S TEST FOR VARIANCES 4, 8 5.33 0.0217 | ROBUST S.D. 0.544

----------------------------------------------------------------------------

************ ************

HISTOGRAM OF * STinfmr2 * ( 423) GROUPED BY * pferd * ( 2)

************ ************

CASES WITH

UNUSED

VALUES FOR

*9 *10 *15 *16 *18 pferd

MIDPOINTS...................+...................+...................+...................+...................+...................+

0.540000)

0.480000) * *

0.420000)

0.360000) *

0.300000)

0.240000) N

0.180000) * *

0.120000)* M M

0.060000)M * M M* *

0.000000) * * **

-.060000)

-.120000)

-.180000) *

LEGEND FOR GROUP MEANS: M - MEAN COINCIDES WITH AN ASTERISK

N - MEAN DOES NOT COINCIDE WITH ANY ASTERISK

MEAN 0.080 0.232 0.133 0.072 0.068 0.098

STD.DEV. 0.041 0.249 0.173 0.000 0.030 0.193

S. E. M. 0.029 0.144 0.087 0.000 0.021 0.073

MAXIMUM 0.109 0.505 0.375 0.072 0.089 0.451

MINIMUM 0.051 0.018 -0.017 0.072 0.047 -0.178

CASES EXCL. ( 0) ( 0) ( 0) ( 1) ( 0) ( 0)

CASES INCL. 2 3 4 1 2 7

---------------------------------------------------------------------------- ALL GROUPS COMBINED

| ANALYSIS OF VARIANCE TABLE FOR MEANS TAIL | (EXCEPT CASES WITH UNUSED

| SOURCE SUM OF SQUARES DF MEAN SQUARE F VALUE PROBABILITY | VALUES FOR VARIABLE pferd )

| -------- -------------- ---- ----------- ------- ----------- | MEAN 0.133

| pferd 0.0474 4 0.0118 0.38 0.8145 | STD. DEV. 0.155

| ERROR 0.2165 7 0.0309 | S. E. M. 0.045

|--------------------------------------------------------------------------| MAXIMUM 0.505

| EQUALITY OF MEANS TESTS; VARIANCES ARE NOT ASSUMED TO BE EQUAL | MINIMUM -0.017

| WELCH 4, 6 0.33 0.8483 | CASES EXCLUDED ( 8)

| BROWN-FORSYTHE 4, 4 0.69 0.6355 | CASES INCLUDED 12

|--------------------------------------------------------------------------|

| LEVENE'S TEST FOR VARIANCES 4, 7 1.75 0.2439 | ROBUST S.D. 0.144

----------------------------------------------------------------------------

************ ************

HISTOGRAM OF * STinfdr2 * ( 424) GROUPED BY * pferd * ( 2)

************ ************

CASES WITH

UNUSED

VALUES FOR

*9 *10 *15 *16 *18 pferd

MIDPOINTS...................+...................+...................+...................+...................+...................+

0.320000) * *

0.280000) *

0.240000)

0.200000) * N

0.160000)

0.120000) M

0.080000) M* * * N

0.040000)M* * ** N ***

0.000000) *

-.040000) *

-.080000)

-.120000)

-.160000) *

LEGEND FOR GROUP MEANS: M - MEAN COINCIDES WITH AN ASTERISK

N - MEAN DOES NOT COINCIDE WITH ANY ASTERISK

MEAN 0.030 0.115 0.066 0.193 0.047 0.071

STD.DEV. 0.012 0.073 0.022 0.160 0.038 0.160

S. E. M. 0.009 0.042 0.011 0.113 0.027 0.061

MAXIMUM 0.039 0.181 0.087 0.306 0.073 0.300

MINIMUM 0.021 0.037 0.044 0.080 0.020 -0.157

CASES EXCL. ( 0) ( 0) ( 0) ( 0) ( 0) ( 0)

CASES INCL. 2 3 4 2 2 7

---------------------------------------------------------------------------- ALL GROUPS COMBINED

| ANALYSIS OF VARIANCE TABLE FOR MEANS TAIL | (EXCEPT CASES WITH UNUSED

| SOURCE SUM OF SQUARES DF MEAN SQUARE F VALUE PROBABILITY | VALUES FOR VARIABLE pferd )

| -------- -------------- ---- ----------- ------- ----------- | MEAN 0.088

| pferd 0.0363 4 0.0091 1.85 0.2124 | STD. DEV. 0.079

| ERROR 0.0392 8 0.0049 | S. E. M. 0.022

|--------------------------------------------------------------------------| MAXIMUM 0.306

| EQUALITY OF MEANS TESTS; VARIANCES ARE NOT ASSUMED TO BE EQUAL | MINIMUM 0.020

| WELCH 4, 3 1.66 0.3524 | CASES EXCLUDED ( 7)

| BROWN-FORSYTHE 4, 2 1.33 0.4726 | CASES INCLUDED 13

|--------------------------------------------------------------------------|

| LEVENE'S TEST FOR VARIANCES 4, 8 12.36 0.0017 | ROBUST S.D. 0.070

----------------------------------------------------------------------------

/ FINISH

PROGRAM TERMINATED

BMDP7D - ONE- AND TWO-WAY ANALYSIS OF VARIANCE WITH DATA SCREENING

Copyright 1977, 1979, 1981, 1982, 1983, 1985, 1987, 1988, 1990, 1993

by BMDP Statistical Software, Inc.

Statistical Solutions Ltd. | Statistical Solutions

Unit 1A, South Ring Business Park | Stonehill Corporate Center, Suite 104

Kinsale Road, Cork, Ireland | 999 Broadway, Saugus, MA 01906, USA

Phone: + 353 21 4319629 | Phone: 781.231.7680

Fax: + 353 21 4319630 | Fax: 781.231.7684

e-mail: sales@statsol.ie | e-mail: info@statsolusa.com

Website: http://www.statsol.ie | Website: http://www.statsolusa.com

Release: 8.1 (Windows 9x, 2000, Me, Xp) Date: 06/07/17 at 10:18:06

Manual: BMDP Manual Volumes 1, 2, and 3.

Digest: BMDP User's Digest.

IBM PC: BMDP PC Supplement -- Installation and Special Features.

PROGRAM INSTRUCTIONS

/prob title = 'Herr Lauritz Englisch: C7D2.inp *** Quantitative Studien

zum Schmelzgehalt in Pferdebackenzaehnen.

- 1 Fall = 1 Zahn = 10 Lokalisationen = 10 Zeilen

- Hier: Oberkiefer, Zahn 7 - 10

- Vergleich der Mittelwerte des Zahnzements

bzgl. der Lokalisation im Zahnfach

- Umrechnung der relativen Werte in Prozentwerte

- Berechnung der Mittelwerte "M" ueber die Schnittebenen

getrennt fuer innerhalb und ausserhalb des Zahnfachs

- Berechnung der Differenz der Mittelwerte "M" innerhalb jedes Zahnes

- einfaktorielle Varianzanalyse

***'.

/inp var = 278.

file = c.

format = '34f10,9(/70x,27f10), /40x,1f10'. ## Mit Abstandsangabe für die Ebene

/var names = zahnid,pferd,rasse,zp,qu,znr,za,

for lo = 0 to 9.% ## Lokalisation

for va = lok,statu,ps,pz,isd,izd,ism,izm,idz,id,ges,sges,dz_sb,i_zb,

inf,ps_rel,s_rel,pz_r,isd_r,izd_r,ism_r,izm_r,idz_r,

id_r,sges_r,i_zb_r,inf_r.%

|va||lo|,%%

abst99. ## Mit Abstandsangabe für die Ebene 99

use = pferd,znr,

for va = pz,pz_r.%

for lo = 0 to 9.% ## Lokalisation

|va||lo|,%%

for va = pz,pz_r.%

M1|va|,M3|va|,D13|va|,%.

/group codes(pferd) = 9,15,18. ## diese Pferde haben mehr als 1 Zahn in der Au

/trans use = ((znr ge 7) AND (znr le 10)).

# Umrechnung der relativen Werte in Prozentwerte

for lo = 0 to 9.%

pz_r|lo| = pz_r|lo| * 100.%

# Selektion der Schnittebenen nach Position bzgl. des Zahnfachs

for va = pz,pz_r.%

# status = 1

for lo = 0 to 9.%

tmp|lo| = XMIS. ## vorbesetzen

if (statu|lo| eq 1 ) then tmp|lo| = |va||lo|.

%

M1|va| = mean(for lo = 0 to 9.% tmp|lo|,%).

# status = 3

for lo = 0 to 9.%

tmp|lo| = XMIS. ## vorbesetzen

if (statu|lo| eq 3 ) then tmp|lo| = |va||lo|.

%

M3|va| = mean(for lo = 0 to 9.% tmp|lo|,%).

# Berechnung der Differenz

D13|va| = M1|va| - M3|va|.

%

/hist group = Pferd.

var = for va = pz,pz_r.%

D13|va|,%.

/print level = min.

case = 0.

/end

--- PROGRAM INSTRUCTIONS AFTER "FOR %" EXPANSION ---

/prob title = 'Herr Lauritz Englisch: C7D2.inp *** Quantitative Studien zum

Schmelzgehalt in Pferdebackenzaehnen.

- 1 Fall = 1 Zahn = 10 Lokalisationen =

10 Zeilen - Hier: Oberkiefer,

Zahn 7 - 10 - Vergleich der Mittelwerte des Zahnzements bzgl.

der Lokalisation im Zahnfach - Umrechnung der relativen Werte in

Prozentwerte - Berechnung der Mittelwerte "M" ueber die

Schnittebenen getrennt fuer innerhalb und ausserhalb des Zahnfachs -

Berechnung der Differenz der Mittelwerte "M" innerhalb jedes Zahnes

- einfaktorielle Varianzanalyse ***'.

/inp var = 278. file = c. format = '34f10,9(/70x,27f10),

/40x,1f10'.

/var names = zahnid,pferd,rasse,zp,qu,znr,za, lok0, statu0, ps0, pz0, isd0,

izd0, ism0, izm0, idz0, id0, ges0, sges0, dz_sb0, i_zb0, inf0,

ps_rel0, s_rel0, pz_r0, isd_r0, izd_r0, ism_r0, izm_r0, idz_r0,

id_r0, sges_r0, i_zb_r0, inf_r0, lok1, statu1, ps1, pz1, isd1,

izd1, ism1, izm1, idz1, id1, ges1, sges1, dz_sb1, i_zb1, inf1,

ps_rel1, s_rel1, pz_r1, isd_r1, izd_r1, ism_r1, izm_r1, idz_r1,

id_r1, sges_r1, i_zb_r1, inf_r1, lok2, statu2, ps2, pz2, isd2,

izd2, ism2, izm2, idz2, id2, ges2, sges2, dz_sb2, i_zb2, inf2,

ps_rel2, s_rel2, pz_r2, isd_r2, izd_r2, ism_r2, izm_r2, idz_r2,

id_r2, sges_r2, i_zb_r2, inf_r2, lok3, statu3, ps3, pz3, isd3,

izd3, ism3, izm3, idz3, id3, ges3, sges3, dz_sb3, i_zb3, inf3,

ps_rel3, s_rel3, pz_r3, isd_r3, izd_r3, ism_r3, izm_r3, idz_r3,

id_r3, sges_r3, i_zb_r3, inf_r3, lok4, statu4, ps4, pz4, isd4,

izd4, ism4, izm4, idz4, id4, ges4, sges4, dz_sb4, i_zb4, inf4,

ps_rel4, s_rel4, pz_r4, isd_r4, izd_r4, ism_r4, izm_r4, idz_r4,

id_r4, sges_r4, i_zb_r4, inf_r4, lok5, statu5, ps5, pz5, isd5,

izd5, ism5, izm5, idz5, id5, ges5, sges5, dz_sb5, i_zb5, inf5,

ps_rel5, s_rel5, pz_r5, isd_r5, izd_r5, ism_r5, izm_r5, idz_r5,

id_r5, sges_r5, i_zb_r5, inf_r5, lok6, statu6, ps6, pz6, isd6,

izd6, ism6, izm6, idz6, id6, ges6, sges6, dz_sb6, i_zb6, inf6,

ps_rel6, s_rel6, pz_r6, isd_r6, izd_r6, ism_r6, izm_r6, idz_r6,

id_r6, sges_r6, i_zb_r6, inf_r6, lok7, statu7, ps7, pz7, isd7,

izd7, ism7, izm7, idz7, id7, ges7, sges7, dz_sb7, i_zb7, inf7,

ps_rel7, s_rel7, pz_r7, isd_r7, izd_r7, ism_r7, izm_r7, idz_r7,

id_r7, sges_r7, i_zb_r7, inf_r7, lok8, statu8, ps8, pz8, isd8,

izd8, ism8, izm8, idz8, id8, ges8, sges8, dz_sb8, i_zb8, inf8,

ps_rel8, s_rel8, pz_r8, isd_r8, izd_r8, ism_r8, izm_r8, idz_r8,

id_r8, sges_r8, i_zb_r8, inf_r8, lok9, statu9, ps9, pz9, isd9,

izd9, ism9, izm9, idz9, id9, ges9, sges9, dz_sb9, i_zb9, inf9,

ps_rel9, s_rel9, pz_r9, isd_r9, izd_r9, ism_r9, izm_r9, idz_r9,

id_r9, sges_r9, i_zb_r9, inf_r9, abst99.

use = pferd,znr, pz0, pz1, pz2, pz3, pz4, pz5, pz6, pz7, pz8,

pz9, pz_r0, pz_r1, pz_r2, pz_r3, pz_r4, pz_r5, pz_r6,

pz_r7, pz_r8, pz_r9, M1pz,M3pz,D13pz, M1pz_r,M3pz_r,D13pz_r.

/group codes(pferd) = 9,15,18.

/trans use = ((znr ge 7) AND (znr le 10)). pz_r0 = pz_r0 * 100.

pz_r1 = pz_r1 * 100. pz_r2 = pz_r2 * 100. pz_r3 = pz_r3 * 100.

pz_r4 = pz_r4 * 100. pz_r5 = pz_r5 * 100. pz_r6 = pz_r6 * 100.

pz_r7 = pz_r7 * 100. pz_r8 = pz_r8 * 100. pz_r9 = pz_r9 * 100.

tmp0 = XMIS. if (statu0 eq 1 ) then tmp0 = pz0. tmp1 = XMIS.

if (statu1 eq 1 ) then tmp1 = pz1. tmp2 = XMIS.

if (statu2 eq 1 ) then tmp2 = pz2. tmp3 = XMIS.

if (statu3 eq 1 ) then tmp3 = pz3. tmp4 = XMIS.

if (statu4 eq 1 ) then tmp4 = pz4. tmp5 = XMIS.

if (statu5 eq 1 ) then tmp5 = pz5. tmp6 = XMIS.

if (statu6 eq 1 ) then tmp6 = pz6. tmp7 = XMIS.

if (statu7 eq 1 ) then tmp7 = pz7. tmp8 = XMIS.

if (statu8 eq 1 ) then tmp8 = pz8. tmp9 = XMIS.

if (statu9 eq 1 ) then tmp9 = pz9.

M1pz = mean( tmp0, tmp1, tmp2, tmp3, tmp4, tmp5, tmp6, tmp7, tmp8, tmp9).

tmp0 = XMIS. if (statu0 eq 3 ) then tmp0 = pz0. tmp1 = XMIS.

if (statu1 eq 3 ) then tmp1 = pz1. tmp2 = XMIS.

if (statu2 eq 3 ) then tmp2 = pz2. tmp3 = XMIS.

if (statu3 eq 3 ) then tmp3 = pz3. tmp4 = XMIS.

if (statu4 eq 3 ) then tmp4 = pz4. tmp5 = XMIS.

if (statu5 eq 3 ) then tmp5 = pz5. tmp6 = XMIS.

if (statu6 eq 3 ) then tmp6 = pz6. tmp7 = XMIS.

if (statu7 eq 3 ) then tmp7 = pz7. tmp8 = XMIS.

if (statu8 eq 3 ) then tmp8 = pz8. tmp9 = XMIS.

if (statu9 eq 3 ) then tmp9 = pz9.

M3pz = mean( tmp0, tmp1, tmp2, tmp3, tmp4, tmp5, tmp6, tmp7, tmp8, tmp9).

D13pz = M1pz - M3pz. tmp0 = XMIS.

if (statu0 eq 1 ) then tmp0 = pz_r0. tmp1 = XMIS.

if (statu1 eq 1 ) then tmp1 = pz_r1. tmp2 = XMIS.

if (statu2 eq 1 ) then tmp2 = pz_r2. tmp3 = XMIS.

if (statu3 eq 1 ) then tmp3 = pz_r3. tmp4 = XMIS.

if (statu4 eq 1 ) then tmp4 = pz_r4. tmp5 = XMIS.

if (statu5 eq 1 ) then tmp5 = pz_r5. tmp6 = XMIS.

if (statu6 eq 1 ) then tmp6 = pz_r6. tmp7 = XMIS.

if (statu7 eq 1 ) then tmp7 = pz_r7. tmp8 = XMIS.

if (statu8 eq 1 ) then tmp8 = pz_r8. tmp9 = XMIS.

if (statu9 eq 1 ) then tmp9 = pz_r9.

M1pz_r = mean( tmp0, tmp1, tmp2, tmp3, tmp4, tmp5, tmp6, tmp7, tmp8,

tmp9). tmp0 = XMIS. if (statu0 eq 3 ) then tmp0 = pz_r0.

tmp1 = XMIS. if (statu1 eq 3 ) then tmp1 = pz_r1. tmp2 = XMIS.

if (statu2 eq 3 ) then tmp2 = pz_r2. tmp3 = XMIS.

if (statu3 eq 3 ) then tmp3 = pz_r3. tmp4 = XMIS.

if (statu4 eq 3 ) then tmp4 = pz_r4. tmp5 = XMIS.

if (statu5 eq 3 ) then tmp5 = pz_r5. tmp6 = XMIS.

if (statu6 eq 3 ) then tmp6 = pz_r6. tmp7 = XMIS.

if (statu7 eq 3 ) then tmp7 = pz_r7. tmp8 = XMIS.

if (statu8 eq 3 ) then tmp8 = pz_r8. tmp9 = XMIS.

if (statu9 eq 3 ) then tmp9 = pz_r9.

M3pz_r = mean( tmp0, tmp1, tmp2, tmp3, tmp4, tmp5, tmp6, tmp7, tmp8,

tmp9). D13pz_r = M1pz_r - M3pz_r.

/hist group = Pferd. var = D13pz, D13pz_r.

/print level = min. case = 0.

/end/

NUMBER OF CASES READ. . . . . . . . . . . . . . 28

CASES WITH USE SET TO ZERO . . . . . . . . . 8

REMAINING NUMBER OF CASES . . . . . . . . 20

DESCRIPTIVE STATISTICS OF DATA

----------- ---------- -- ----

VARIABLE TOTAL STANDARD ST.ERR COEFF SMALLEST LARGEST

NO. NAME FREQ. MEAN DEV. OF MEAN OF VAR VALUE VALUE RANGE

2 pferd 20 12.400 4.4651 .99842 .36009 2.0000 18.000 16.000

6 znr 20 8.5000 1.1471 .25649 .13495 7.0000 10.000 3.0000

11 pz0 19 27.856 12.064 2.7676 .43307 10.680 57.830 47.150

38 pz1 19 76.448 40.825 9.3659 .53402 30.350 165.71 135.36

65 pz2 19 104.01 59.192 13.580 .56912 33.970 201.47 167.50

92 pz3 9 62.862 29.859 9.9528 .47498 36.950 133.22 96.270

119 pz4 9 107.63 49.818 16.606 .46284 43.970 180.19 136.22

146 pz5 5 133.81 61.615 27.555 .46047 67.240 206.04 138.80

173 pz6 2 144.55 8.2095 5.8050 .05679 138.75 150.36 11.610

200 pz7 0

227 pz8 0

254 pz9 20 143.61 33.160 7.4148 .23090 58.230 185.52 127.29

25 pz_r0 18 5.1635 2.0452 .48205 .39608 1.8497 9.2853 7.4356

52 pz_r1 19 13.779 9.3213 2.1384 .67648 4.8429 43.549 38.707

79 pz_r2 19 17.055 10.365 2.3778 .60773 5.3265 44.553 39.227

106 pz_r3 9 10.176 5.1604 1.7201 .50713 6.5927 22.622 16.029

133 pz_r4 9 15.560 6.2185 2.0728 .39964 7.8924 25.340 17.448

160 pz_r5 5 18.340 7.1584 3.2013 .39031 11.479 26.208 14.729

187 pz_r6 2 19.686 1.0292 .72779 .05228 18.958 20.414 1.4556

214 pz_r7 0

241 pz_r8 0

268 pz_r9 20 21.576 4.5885 1.0260 .21267 12.054 35.276 23.222

289 M1pz 20 43.847 11.759 2.6294 .26818 23.980 71.495 47.515

290 M3pz 20 148.80 32.527 7.2733 .21860 58.230 185.48 127.25

291 D13pz 20 -104.95 38.560 8.6223 -.36741 -151.77 13.265 165.04

292 M1pz_r 19 7.8440 2.3470 .53843 .29921 4.4662 13.273 8.8068

293 M3pz_r 20 22.298 5.3232 1.1903 .23873 12.054 39.914 27.861

294 D13pz_r 19 -14.566 6.5074 1.4929 -.44676 -33.056 1.2192 34.275

************ ************

HISTOGRAM OF * D13pz * ( 291) GROUPED BY * pferd * ( 2)

************ ************

CASES WITH

UNUSED

VALUES FOR

*9 *15 *18 pferd

MIDPOINTS.............................+.............................+.............................+.............................+

30.000)

15.000) *

0.000)

-15.000)

-30.000)

-45.000)

-60.000) *

-75.000) *

-90.000)* M* M*

-105.000) ***

-120.000)N * **

-135.000) M **

-150.000)* **

LEGEND FOR GROUP MEANS: M - MEAN COINCIDES WITH AN ASTERISK

N - MEAN DOES NOT COINCIDE WITH ANY ASTERISK

MEAN -123.660 -139.724 -92.840 -92.259

STD.DEV. 37.265 12.890 1.414 41.119

S. E. M. 26.351 6.445 1.000 11.870

MAXIMUM -97.309 -122.125 -91.840 13.265

MINIMUM -150.010 -151.770 -93.840 -137.100

CASES EXCL. ( 0) ( 0) ( 0) ( 0)

CASES INCL. 2 4 2 12

---------------------------------------------------------------------------- ALL GROUPS COMBINED

| ANALYSIS OF VARIANCE TABLE FOR MEANS TAIL | (EXCEPT CASES WITH UNUSED

| SOURCE SUM OF SQUARES DF MEAN SQUARE F VALUE PROBABILITY | VALUES FOR VARIABLE pferd )

| -------- -------------- ---- ----------- ------- ----------- | MEAN -1.2E+2

| pferd 2931.0676 2 1465.5338 3.88 0.0962 | STD. DEV. 26.241

| ERROR 1889.1409 5 377.8282 | S. E. M. 9.278

|--------------------------------------------------------------------------| MAXIMUM -91.840

| EQUALITY OF MEANS TESTS; VARIANCES ARE NOT ASSUMED TO BE EQUAL | MINIMUM -1.5E+2

| WELCH 2, 2 19.92 0.0478 | CASES EXCLUDED ( 12)

| BROWN-FORSYTHE 2, 1 2.60 0.4014 | CASES INCLUDED 8

|--------------------------------------------------------------------------|

| LEVENE'S TEST FOR VARIANCES 2, 5 11.11 0.0145 | ROBUST S.D. 30.426

----------------------------------------------------------------------------

************ ************

HISTOGRAM OF * D13pz_r * ( 294) GROUPED BY * pferd * ( 2)

************ ************

CASES WITH

UNUSED

VALUES FOR

*9 *15 *18 pferd

MIDPOINTS.............................+.............................+.............................+.............................+

3.0000)

0.0000) *

-3.0000)

-6.0000)

-9.0000) *

-12.0000) M* ****

-15.0000)* * M***

-18.0000)N M**

-21.0000)*

-24.0000)

-27.0000)

-30.0000)

-33.0000) *

LEGEND FOR GROUP MEANS: M - MEAN COINCIDES WITH AN ASTERISK

N - MEAN DOES NOT COINCIDE WITH ANY ASTERISK

MEAN -16.682 -18.062 -10.696 -13.613

STD.DEV. 4.402 1.319 0.211 7.999

S. E. M. 3.113 0.660 0.149 2.412

MAXIMUM -13.569 -16.355 -10.546 1.219

MINIMUM -19.795 -19.387 -10.845 -33.056

CASES EXCL. ( 0) ( 0) ( 0) ( 1)

CASES INCL. 2 4 2 11

---------------------------------------------------------------------------- ALL GROUPS COMBINED

| ANALYSIS OF VARIANCE TABLE FOR MEANS TAIL | (EXCEPT CASES WITH UNUSED

| SOURCE SUM OF SQUARES DF MEAN SQUARE F VALUE PROBABILITY | VALUES FOR VARIABLE pferd )

| -------- -------------- ---- ----------- ------- ----------- | MEAN -15.876

| pferd 74.0870 2 37.0435 7.52 0.0311 | STD. DEV. 3.756

| ERROR 24.6460 5 4.9292 | S. E. M. 1.328

|--------------------------------------------------------------------------| MAXIMUM -10.546

| EQUALITY OF MEANS TESTS; VARIANCES ARE NOT ASSUMED TO BE EQUAL | MINIMUM -19.795

| WELCH 2, 2 46.00 0.0213 | CASES EXCLUDED ( 12)

| BROWN-FORSYTHE 2, 1 4.80 0.3072 | CASES INCLUDED 8

|--------------------------------------------------------------------------|

| LEVENE'S TEST FOR VARIANCES 2, 5 19.90 0.0042 | ROBUST S.D. 4.243

----------------------------------------------------------------------------

/ FINISH

PROGRAM TERMINATED

BMDP7D - ONE- AND TWO-WAY ANALYSIS OF VARIANCE WITH DATA SCREENING

Copyright 1977, 1979, 1981, 1982, 1983, 1985, 1987, 1988, 1990, 1993

by BMDP Statistical Software, Inc.

Statistical Solutions Ltd. | Statistical Solutions

Unit 1A, South Ring Business Park | Stonehill Corporate Center, Suite 104

Kinsale Road, Cork, Ireland | 999 Broadway, Saugus, MA 01906, USA

Phone: + 353 21 4319629 | Phone: 781.231.7680

Fax: + 353 21 4319630 | Fax: 781.231.7684

e-mail: sales@statsol.ie | e-mail: info@statsolusa.com

Website: http://www.statsol.ie | Website: http://www.statsolusa.com

Release: 8.1 (Windows 9x, 2000, Me, Xp) Date: 06/07/17 at 11:52:21

Manual: BMDP Manual Volumes 1, 2, and 3.

Digest: BMDP User's Digest.

IBM PC: BMDP PC Supplement -- Installation and Special Features.

PROGRAM INSTRUCTIONS

/prob title = 'Herr Lauritz Englisch: D7D2.inp *** Quantitative Studien

zum Schmelzgehalt in Pferdebackenzaehnen.

- 1 Fall = 1 Zahn = 10 Lokalisationen = 10 Zeilen

- Hier: Oberkiefer, Zahn 7 - 10

- Vergleich der Mittelwerte des Zahnzements

bzgl. der Lokalisation im Zahnfach

- Umrechnung der relativen Werte in Prozentwerte

- Berechnung der Mittelwerte "M" ueber die Schnittebenen

getrennt fuer innerhalb und ausserhalb des Zahnfachs

- Berechnung der Differenz der Mittelwerte "M" innerhalb jedes Zahnes

- einfaktorielle Varianzanalyse

***'.

/inp var = 89.

file = d. ## analog zu Datensatz B.Dat

format = '16f10,8(/70x,9f10), /40x,1f10'. ## Mit Abstandsangabe für die Ebene 9

/var names = zahnid,pferd,rasse,zp,qu,znr,za,

for lo = 0 to 8.% ## Lokalisation

for va = lok,statu,ps,pz,id,ges,s_rel,z_rel,d_rel.%

|va||lo|,%%

abst99. ## Mit Abstandsangabe für die Ebene 99

use = pferd,znr,

for va = pz,z_rel.%

for lo = 0 to 8.% ## Lokalisation

|va||lo|,%%

for va = pz,z_rel.%

M1|va|,M3|va|,D13|va|,%.

/group codes(pferd) = 7 to 10. ## diese Pferde haben mehr als 1 Zahn in der Au

/trans use = ((znr ge 7) AND (znr le 10)).

# Umrechnung der relativen Werte in Prozentwerte

for lo = 0 to 8.%

z_rel|lo| = z_rel|lo| * 100.%

# Selektion der Schnittebenen nach Position bzgl. des Zahnfachs

for va = pz,z_rel.%

# status = 1

for lo = 0 to 8.%

tmp|lo| = XMIS. ## vorbesetzen

if (statu|lo| eq 1 ) then tmp|lo| = |va||lo|.

%

M1|va| = mean(for lo = 0 to 8.% tmp|lo|,%).

# status = 3

for lo = 0 to 8.%

tmp|lo| = XMIS. ## vorbesetzen

if (statu|lo| eq 3 ) then tmp|lo| = |va||lo|.

%

M3|va| = mean(for lo = 0 to 8.% tmp|lo|,%).

# Berechnung der Differenz

D13|va| = M1|va| - M3|va|.

%

/hist group = Pferd.

var = for va = pz,z_rel.%

D13|va|,%.

/print level = min.

case = 0.

/end

--- PROGRAM INSTRUCTIONS AFTER "FOR %" EXPANSION ---

/prob title = 'Herr Lauritz Englisch: D7D2.inp *** Quantitative Studien zum

Schmelzgehalt in Pferdebackenzaehnen.

- 1 Fall = 1 Zahn = 10 Lokalisationen =

10 Zeilen - Hier: Oberkiefer,

Zahn 7 - 10 - Vergleich der Mittelwerte des Zahnzements bzgl.

der Lokalisation im Zahnfach - Umrechnung der relativen Werte in

Prozentwerte - Berechnung der Mittelwerte "M" ueber die

Schnittebenen getrennt fuer innerhalb und ausserhalb des Zahnfachs -

Berechnung der Differenz der Mittelwerte "M" innerhalb jedes Zahnes

- einfaktorielle Varianzanalyse ***'.

/inp var = 89. file = d. format = '16f10,8(/70x,9f10),

/40x,1f10'.

/var names = zahnid,pferd,rasse,zp,qu,znr,za, lok0, statu0, ps0, pz0, id0,

ges0, s_rel0, z_rel0, d_rel0, lok1, statu1, ps1, pz1, id1, ges1,

s_rel1, z_rel1, d_rel1, lok2, statu2, ps2, pz2, id2, ges2, s_rel2,

z_rel2, d_rel2, lok3, statu3, ps3, pz3, id3, ges3, s_rel3, z_rel3,

d_rel3, lok4, statu4, ps4, pz4, id4, ges4, s_rel4, z_rel4, d_rel4,

lok5, statu5, ps5, pz5, id5, ges5, s_rel5, z_rel5, d_rel5, lok6,

statu6, ps6, pz6, id6, ges6, s_rel6, z_rel6, d_rel6, lok7, statu7,

ps7, pz7, id7, ges7, s_rel7, z_rel7, d_rel7, lok8, statu8, ps8,

pz8, id8, ges8, s_rel8, z_rel8, d_rel8, abst99.

use = pferd,znr, pz0, pz1, pz2, pz3, pz4, pz5, pz6, pz7, pz8,

z_rel0, z_rel1, z_rel2, z_rel3, z_rel4, z_rel5, z_rel6,

z_rel7, z_rel8, M1pz,M3pz,D13pz, M1z_rel,M3z_rel,D13z_rel.

/group codes(pferd) = 7 to 10.

/trans use = ((znr ge 7) AND (znr le 10)). z_rel0 = z_rel0 * 100.

z_rel1 = z_rel1 * 100. z_rel2 = z_rel2 * 100. z_rel3 = z_rel3 * 100.

z_rel4 = z_rel4 * 100. z_rel5 = z_rel5 * 100. z_rel6 = z_rel6 * 100.

z_rel7 = z_rel7 * 100. z_rel8 = z_rel8 * 100. tmp0 = XMIS.

if (statu0 eq 1 ) then tmp0 = pz0. tmp1 = XMIS.

if (statu1 eq 1 ) then tmp1 = pz1. tmp2 = XMIS.

if (statu2 eq 1 ) then tmp2 = pz2. tmp3 = XMIS.

if (statu3 eq 1 ) then tmp3 = pz3. tmp4 = XMIS.

if (statu4 eq 1 ) then tmp4 = pz4. tmp5 = XMIS.

if (statu5 eq 1 ) then tmp5 = pz5. tmp6 = XMIS.

if (statu6 eq 1 ) then tmp6 = pz6. tmp7 = XMIS.

if (statu7 eq 1 ) then tmp7 = pz7. tmp8 = XMIS.

if (statu8 eq 1 ) then tmp8 = pz8.

M1pz = mean( tmp0, tmp1, tmp2, tmp3, tmp4, tmp5, tmp6, tmp7, tmp8).

tmp0 = XMIS. if (statu0 eq 3 ) then tmp0 = pz0. tmp1 = XMIS.

if (statu1 eq 3 ) then tmp1 = pz1. tmp2 = XMIS.

if (statu2 eq 3 ) then tmp2 = pz2. tmp3 = XMIS.

if (statu3 eq 3 ) then tmp3 = pz3. tmp4 = XMIS.

if (statu4 eq 3 ) then tmp4 = pz4. tmp5 = XMIS.

if (statu5 eq 3 ) then tmp5 = pz5. tmp6 = XMIS.

if (statu6 eq 3 ) then tmp6 = pz6. tmp7 = XMIS.

if (statu7 eq 3 ) then tmp7 = pz7. tmp8 = XMIS.

if (statu8 eq 3 ) then tmp8 = pz8.

M3pz = mean( tmp0, tmp1, tmp2, tmp3, tmp4, tmp5, tmp6, tmp7, tmp8).

D13pz = M1pz - M3pz. tmp0 = XMIS.

if (statu0 eq 1 ) then tmp0 = z_rel0. tmp1 = XMIS.

if (statu1 eq 1 ) then tmp1 = z_rel1. tmp2 = XMIS.

if (statu2 eq 1 ) then tmp2 = z_rel2. tmp3 = XMIS.

if (statu3 eq 1 ) then tmp3 = z_rel3. tmp4 = XMIS.

if (statu4 eq 1 ) then tmp4 = z_rel4. tmp5 = XMIS.

if (statu5 eq 1 ) then tmp5 = z_rel5. tmp6 = XMIS.

if (statu6 eq 1 ) then tmp6 = z_rel6. tmp7 = XMIS.

if (statu7 eq 1 ) then tmp7 = z_rel7. tmp8 = XMIS.

if (statu8 eq 1 ) then tmp8 = z_rel8.

M1z_rel = mean( tmp0, tmp1, tmp2, tmp3, tmp4, tmp5, tmp6, tmp7, tmp8).

tmp0 = XMIS. if (statu0 eq 3 ) then tmp0 = z_rel0. tmp1 = XMIS.

if (statu1 eq 3 ) then tmp1 = z_rel1. tmp2 = XMIS.

if (statu2 eq 3 ) then tmp2 = z_rel2. tmp3 = XMIS.

if (statu3 eq 3 ) then tmp3 = z_rel3. tmp4 = XMIS.

if (statu4 eq 3 ) then tmp4 = z_rel4. tmp5 = XMIS.

if (statu5 eq 3 ) then tmp5 = z_rel5. tmp6 = XMIS.

if (statu6 eq 3 ) then tmp6 = z_rel6. tmp7 = XMIS.

if (statu7 eq 3 ) then tmp7 = z_rel7. tmp8 = XMIS.

if (statu8 eq 3 ) then tmp8 = z_rel8.

M3z_rel = mean( tmp0, tmp1, tmp2, tmp3, tmp4, tmp5, tmp6, tmp7, tmp8).

D13z_rel = M1z_rel - M3z_rel.

/hist group = Pferd. var = D13pz, D13z_rel.

/print level = min. case = 0.

/end/

NUMBER OF CASES READ. . . . . . . . . . . . . . 26

CASES WITH USE SET TO ZERO . . . . . . . . . 10

REMAINING NUMBER OF CASES . . . . . . . . 16

DESCRIPTIVE STATISTICS OF DATA

----------- ---------- -- ----

VARIABLE TOTAL STANDARD ST.ERR COEFF SMALLEST LARGEST

NO. NAME FREQ. MEAN DEV. OF MEAN OF VAR VALUE VALUE RANGE

2 pferd 16 7.0625 2.9090 .72726 .41190 1.0000 10.000 9.0000

6 znr 16 8.0000 1.1547 .28868 .14434 7.0000 10.000 3.0000

11 pz0 16 50.933 35.626 8.9066 .69947 16.270 123.60 107.33

20 pz1 16 109.45 64.351 16.088 .58795 32.440 208.98 176.54

29 pz2 16 159.88 83.986 20.996 .52530 40.650 292.54 251.89

38 pz3 9 142.12 84.246 28.082 .59277 51.940 279.64 227.70

47 pz4 5 96.750 35.789 16.006 .36992 55.760 137.65 81.890

56 pz5 5 162.12 89.864 40.188 .55431 65.850 267.20 201.35

65 pz6 4 208.12 27.576 13.788 .13250 187.00 247.63 60.630

74 pz7 1 253.18 0.0000 0.0000 0.0000 253.18 253.18 0.0000

83 pz8 16 165.91 42.773 10.693 .25781 92.850 257.61 164.76

15 z_rel0 16 14.681 8.4979 2.1245 .57883 4.9000 27.000 22.100

24 z_rel1 16 25.912 11.793 2.9481 .45509 10.600 42.300 31.700

33 z_rel2 16 33.500 13.764 3.4410 .41086 12.600 49.900 37.300

42 z_rel3 9 28.722 11.480 3.8266 .39969 15.600 43.000 27.400

51 z_rel4 5 22.640 5.3984 2.4142 .23845 16.200 29.200 13.000

60 z_rel5 5 32.080 10.560 4.7224 .32917 19.500 43.000 23.500

69 z_rel6 4 40.475 5.0408 2.5204 .12454 36.200 47.600 11.400

78 z_rel7 1 43.700 0.0000 0.0000 0.0000 43.700 43.700 0.0000

87 z_rel8 16 35.956 4.2417 1.0604 .11797 26.600 46.800 20.200

99 M1pz 16 71.677 23.757 5.9392 .33144 38.650 126.72 88.070

100 M3pz 16 192.95 38.738 9.6845 .20077 129.62 275.08 145.46

101 D13pz 16 -121.27 32.439 8.1096 -.26749 -200.72 -66.540 134.18

102 M1z_rel 16 19.361 4.1758 1.0440 .21568 13.567 27.000 13.433

103 M3z_rel 16 39.456 3.2734 .81836 .08296 36.000 48.350 12.350

104 D13z_rel 16 -20.095 5.0483 1.2621 -.25122 -27.570 -10.000 17.570

************ ************

HISTOGRAM OF * D13pz * ( 101) GROUPED BY * pferd * ( 2)

************ ************

CASES WITH

UNUSED

VALUES FOR

*7 *8 *9 *10 pferd

MIDPOINTS.......................+.......................+.......................+.......................+.......................+

-45.000)

-60.000) *

-75.000) *

-90.000) M

-105.000) * M*

-120.000)* ** **

-135.000)M N

-150.000) * **

-165.000)

-180.000) N

-195.000) *

-210.000)

LEGEND FOR GROUP MEANS: M - MEAN COINCIDES WITH AN ASTERISK

N - MEAN DOES NOT COINCIDE WITH ANY ASTERISK

MEAN -128.712 -173.600 -135.056 -87.683 -106.487

STD.DEV. 5.123 38.346 21.161 19.708 18.318

S. E. M. 3.622 27.115 10.581 11.379 8.192

MAXIMUM -125.090 -146.485 -115.033 -66.540 -78.580

MINIMUM -132.335 -200.715 -154.740 -105.545 -125.605

CASES EXCL. ( 0) ( 0) ( 0) ( 0) ( 0)

CASES INCL. 2 2 4 3 5

---------------------------------------------------------------------------- ALL GROUPS COMBINED

| ANALYSIS OF VARIANCE TABLE FOR MEANS TAIL | (EXCEPT CASES WITH UNUSED

| SOURCE SUM OF SQUARES DF MEAN SQUARE F VALUE PROBABILITY | VALUES FOR VARIABLE pferd )

| -------- -------------- ---- ----------- ------- ----------- | MEAN -1.3E+2

| pferd 9235.1738 3 3078.3914 5.96 0.0243 | STD. DEV. 35.850

| ERROR 3616.9421 7 516.7060 | S. E. M. 10.809

|--------------------------------------------------------------------------| MAXIMUM -66.540

| EQUALITY OF MEANS TESTS; VARIANCES ARE NOT ASSUMED TO BE EQUAL | MINIMUM -2.0E+2

| WELCH 3, 3 3.59 0.1606 | CASES EXCLUDED ( 5)

| BROWN-FORSYTHE 3, 2 5.15 0.1668 | CASES INCLUDED 11

|--------------------------------------------------------------------------|

| LEVENE'S TEST FOR VARIANCES 3, 7 7.14 0.0155 | ROBUST S.D. 34.942

----------------------------------------------------------------------------

************ ************

HISTOGRAM OF * D13z_rel * ( 104) GROUPED BY * pferd * ( 2)

************ ************

CASES WITH

UNUSED

VALUES FOR

*7 *8 *9 *10 pferd

MIDPOINTS.......................+.......................+.......................+.......................+.......................+

-9.0000)

-10.8000) *

-12.6000) **

-14.4000)

-16.2000)* N

-18.0000) N **

-19.8000)N *

-21.6000) * * * *

-23.4000)* N N *

-25.2000) *

-27.0000) * *

-28.8000)

LEGEND FOR GROUP MEANS: M - MEAN COINCIDES WITH AN ASTERISK

N - MEAN DOES NOT COINCIDE WITH ANY ASTERISK

MEAN -20.025 -23.925 -23.821 -18.483 -16.578

STD.DEV. 4.207 4.561 3.325 7.405 3.407

S. E. M. 2.975 3.225 1.663 4.275 1.524

MAXIMUM -17.050 -20.700 -19.930 -10.000 -12.800

MINIMUM -23.000 -27.150 -27.570 -23.650 -20.800

CASES EXCL. ( 0) ( 0) ( 0) ( 0) ( 0)

CASES INCL. 2 2 4 3 5

---------------------------------------------------------------------------- ALL GROUPS COMBINED

| ANALYSIS OF VARIANCE TABLE FOR MEANS TAIL | (EXCEPT CASES WITH UNUSED

| SOURCE SUM OF SQUARES DF MEAN SQUARE F VALUE PROBABILITY | VALUES FOR VARIABLE pferd )

| -------- -------------- ---- ----------- ------- ----------- | MEAN -21.694

| pferd 64.5447 3 21.5149 0.83 0.5180 | STD. DEV. 4.959

| ERROR 181.3358 7 25.9051 | S. E. M. 1.495

|--------------------------------------------------------------------------| MAXIMUM -10.000

| EQUALITY OF MEANS TESTS; VARIANCES ARE NOT ASSUMED TO BE EQUAL | MINIMUM -27.570

| WELCH 3, 3 0.53 0.6925 | CASES EXCLUDED ( 5)

| BROWN-FORSYTHE 3, 5 0.82 0.5348 | CASES INCLUDED 11

|--------------------------------------------------------------------------|

| LEVENE'S TEST FOR VARIANCES 3, 7 2.10 0.1882 | ROBUST S.D. 4.564

----------------------------------------------------------------------------

/ FINISH

PROGRAM TERMINATED

BMDP7D - ONE- AND TWO-WAY ANALYSIS OF VARIANCE WITH DATA SCREENING

Copyright 1977, 1979, 1981, 1982, 1983, 1985, 1987, 1988, 1990, 1993

by BMDP Statistical Software, Inc.

Statistical Solutions Ltd. | Statistical Solutions

Unit 1A, South Ring Business Park | Stonehill Corporate Center, Suite 104

Kinsale Road, Cork, Ireland | 999 Broadway, Saugus, MA 01906, USA

Phone: + 353 21 4319629 | Phone: 781.231.7680

Fax: + 353 21 4319630 | Fax: 781.231.7684

e-mail: sales@statsol.ie | e-mail: info@statsolusa.com

Website: http://www.statsol.ie | Website: http://www.statsolusa.com

Release: 8.1 (Windows 9x, 2000, Me, Xp) Date: 06/07/17 at 11:52:21

Manual: BMDP Manual Volumes 1, 2, and 3.

Digest: BMDP User's Digest.

IBM PC: BMDP PC Supplement -- Installation and Special Features.

PROGRAM INSTRUCTIONS

/prob title = 'Herr Lauritz Englisch: D7D2.inp *** Quantitative Studien

zum Schmelzgehalt in Pferdebackenzaehnen.

- 1 Fall = 1 Zahn = 10 Lokalisationen = 10 Zeilen

- Hier: Oberkiefer, Zahn 7 - 10

- Vergleich der Mittelwerte des Zahnzements

bzgl. der Lokalisation im Zahnfach

- Umrechnung der relativen Werte in Prozentwerte

- Berechnung der Mittelwerte "M" ueber die Schnittebenen

getrennt fuer innerhalb und ausserhalb des Zahnfachs

- Berechnung der Differenz der Mittelwerte "M" innerhalb jedes Zahnes

- einfaktorielle Varianzanalyse

***'.

/inp var = 89.

file = d. ## analog zu Datensatz B.Dat

format = '16f10,8(/70x,9f10), /40x,1f10'. ## Mit Abstandsangabe für die Ebene 9

/var names = zahnid,pferd,rasse,zp,qu,znr,za,

for lo = 0 to 8.% ## Lokalisation

for va = lok,statu,ps,pz,id,ges,s_rel,z_rel,d_rel.%

|va||lo|,%%

abst99. ## Mit Abstandsangabe für die Ebene 99

use = pferd,znr,

for va = pz,z_rel.%

for lo = 0 to 8.% ## Lokalisation

|va||lo|,%%

for va = pz,z_rel.%

M1|va|,M3|va|,D13|va|,%.

/group codes(pferd) = 7 to 10. ## diese Pferde haben mehr als 1 Zahn in der Au

/trans use = ((znr ge 7) AND (znr le 10)).

# Umrechnung der relativen Werte in Prozentwerte

for lo = 0 to 8.%

z_rel|lo| = z_rel|lo| * 100.%

# Selektion der Schnittebenen nach Position bzgl. des Zahnfachs

for va = pz,z_rel.%

# status = 1

for lo = 0 to 8.%

tmp|lo| = XMIS. ## vorbesetzen

if (statu|lo| eq 1 ) then tmp|lo| = |va||lo|.

%

M1|va| = mean(for lo = 0 to 8.% tmp|lo|,%).

# status = 3

for lo = 0 to 8.%

tmp|lo| = XMIS. ## vorbesetzen

if (statu|lo| eq 3 ) then tmp|lo| = |va||lo|.

%

M3|va| = mean(for lo = 0 to 8.% tmp|lo|,%).

# Berechnung der Differenz

D13|va| = M1|va| - M3|va|.

%

/hist group = Pferd.

var = for va = pz,z_rel.%

D13|va|,%.

/print level = min.

case = 0.

/end

--- PROGRAM INSTRUCTIONS AFTER "FOR %" EXPANSION ---

/prob title = 'Herr Lauritz Englisch: D7D2.inp *** Quantitative Studien zum

Schmelzgehalt in Pferdebackenzaehnen.

- 1 Fall = 1 Zahn = 10 Lokalisationen =

10 Zeilen - Hier: Oberkiefer,

Zahn 7 - 10 - Vergleich der Mittelwerte des Zahnzements bzgl.

der Lokalisation im Zahnfach - Umrechnung der relativen Werte in

Prozentwerte - Berechnung der Mittelwerte "M" ueber die

Schnittebenen getrennt fuer innerhalb und ausserhalb des Zahnfachs -

Berechnung der Differenz der Mittelwerte "M" innerhalb jedes Zahnes

- einfaktorielle Varianzanalyse ***'.

/inp var = 89. file = d. format = '16f10,8(/70x,9f10),

/40x,1f10'.

/var names = zahnid,pferd,rasse,zp,qu,znr,za, lok0, statu0, ps0, pz0, id0,

ges0, s_rel0, z_rel0, d_rel0, lok1, statu1, ps1, pz1, id1, ges1,

s_rel1, z_rel1, d_rel1, lok2, statu2, ps2, pz2, id2, ges2, s_rel2,

z_rel2, d_rel2, lok3, statu3, ps3, pz3, id3, ges3, s_rel3, z_rel3,

d_rel3, lok4, statu4, ps4, pz4, id4, ges4, s_rel4, z_rel4, d_rel4,

lok5, statu5, ps5, pz5, id5, ges5, s_rel5, z_rel5, d_rel5, lok6,

statu6, ps6, pz6, id6, ges6, s_rel6, z_rel6, d_rel6, lok7, statu7,

ps7, pz7, id7, ges7, s_rel7, z_rel7, d_rel7, lok8, statu8, ps8,

pz8, id8, ges8, s_rel8, z_rel8, d_rel8, abst99.

use = pferd,znr, pz0, pz1, pz2, pz3, pz4, pz5, pz6, pz7, pz8,

z_rel0, z_rel1, z_rel2, z_rel3, z_rel4, z_rel5, z_rel6,

z_rel7, z_rel8, M1pz,M3pz,D13pz, M1z_rel,M3z_rel,D13z_rel.

/group codes(pferd) = 7 to 10.

/trans use = ((znr ge 7) AND (znr le 10)). z_rel0 = z_rel0 * 100.

z_rel1 = z_rel1 * 100. z_rel2 = z_rel2 * 100. z_rel3 = z_rel3 * 100.

z_rel4 = z_rel4 * 100. z_rel5 = z_rel5 * 100. z_rel6 = z_rel6 * 100.

z_rel7 = z_rel7 * 100. z_rel8 = z_rel8 * 100. tmp0 = XMIS.

if (statu0 eq 1 ) then tmp0 = pz0. tmp1 = XMIS.

if (statu1 eq 1 ) then tmp1 = pz1. tmp2 = XMIS.

if (statu2 eq 1 ) then tmp2 = pz2. tmp3 = XMIS.

if (statu3 eq 1 ) then tmp3 = pz3. tmp4 = XMIS.

if (statu4 eq 1 ) then tmp4 = pz4. tmp5 = XMIS.

if (statu5 eq 1 ) then tmp5 = pz5. tmp6 = XMIS.

if (statu6 eq 1 ) then tmp6 = pz6. tmp7 = XMIS.

if (statu7 eq 1 ) then tmp7 = pz7. tmp8 = XMIS.

if (statu8 eq 1 ) then tmp8 = pz8.

M1pz = mean( tmp0, tmp1, tmp2, tmp3, tmp4, tmp5, tmp6, tmp7, tmp8).

tmp0 = XMIS. if (statu0 eq 3 ) then tmp0 = pz0. tmp1 = XMIS.

if (statu1 eq 3 ) then tmp1 = pz1. tmp2 = XMIS.

if (statu2 eq 3 ) then tmp2 = pz2. tmp3 = XMIS.

if (statu3 eq 3 ) then tmp3 = pz3. tmp4 = XMIS.

if (statu4 eq 3 ) then tmp4 = pz4. tmp5 = XMIS.

if (statu5 eq 3 ) then tmp5 = pz5. tmp6 = XMIS.

if (statu6 eq 3 ) then tmp6 = pz6. tmp7 = XMIS.

if (statu7 eq 3 ) then tmp7 = pz7. tmp8 = XMIS.

if (statu8 eq 3 ) then tmp8 = pz8.

M3pz = mean( tmp0, tmp1, tmp2, tmp3, tmp4, tmp5, tmp6, tmp7, tmp8).

D13pz = M1pz - M3pz. tmp0 = XMIS.

if (statu0 eq 1 ) then tmp0 = z_rel0. tmp1 = XMIS.

if (statu1 eq 1 ) then tmp1 = z_rel1. tmp2 = XMIS.

if (statu2 eq 1 ) then tmp2 = z_rel2. tmp3 = XMIS.

if (statu3 eq 1 ) then tmp3 = z_rel3. tmp4 = XMIS.

if (statu4 eq 1 ) then tmp4 = z_rel4. tmp5 = XMIS.

if (statu5 eq 1 ) then tmp5 = z_rel5. tmp6 = XMIS.

if (statu6 eq 1 ) then tmp6 = z_rel6. tmp7 = XMIS.

if (statu7 eq 1 ) then tmp7 = z_rel7. tmp8 = XMIS.

if (statu8 eq 1 ) then tmp8 = z_rel8.

M1z_rel = mean( tmp0, tmp1, tmp2, tmp3, tmp4, tmp5, tmp6, tmp7, tmp8).

tmp0 = XMIS. if (statu0 eq 3 ) then tmp0 = z_rel0. tmp1 = XMIS.

if (statu1 eq 3 ) then tmp1 = z_rel1. tmp2 = XMIS.

if (statu2 eq 3 ) then tmp2 = z_rel2. tmp3 = XMIS.

if (statu3 eq 3 ) then tmp3 = z_rel3. tmp4 = XMIS.

if (statu4 eq 3 ) then tmp4 = z_rel4. tmp5 = XMIS.

if (statu5 eq 3 ) then tmp5 = z_rel5. tmp6 = XMIS.

if (statu6 eq 3 ) then tmp6 = z_rel6. tmp7 = XMIS.

if (statu7 eq 3 ) then tmp7 = z_rel7. tmp8 = XMIS.

if (statu8 eq 3 ) then tmp8 = z_rel8.

M3z_rel = mean( tmp0, tmp1, tmp2, tmp3, tmp4, tmp5, tmp6, tmp7, tmp8).

D13z_rel = M1z_rel - M3z_rel.

/hist group = Pferd. var = D13pz, D13z_rel.

/print level = min. case = 0.

/end/

NUMBER OF CASES READ. . . . . . . . . . . . . . 26

CASES WITH USE SET TO ZERO . . . . . . . . . 10

REMAINING NUMBER OF CASES . . . . . . . . 16

DESCRIPTIVE STATISTICS OF DATA

----------- ---------- -- ----

VARIABLE TOTAL STANDARD ST.ERR COEFF SMALLEST LARGEST

NO. NAME FREQ. MEAN DEV. OF MEAN OF VAR VALUE VALUE RANGE

2 pferd 16 7.0625 2.9090 .72726 .41190 1.0000 10.000 9.0000

6 znr 16 8.0000 1.1547 .28868 .14434 7.0000 10.000 3.0000

11 pz0 16 50.933 35.626 8.9066 .69947 16.270 123.60 107.33

20 pz1 16 109.45 64.351 16.088 .58795 32.440 208.98 176.54

29 pz2 16 159.88 83.986 20.996 .52530 40.650 292.54 251.89

38 pz3 9 142.12 84.246 28.082 .59277 51.940 279.64 227.70

47 pz4 5 96.750 35.789 16.006 .36992 55.760 137.65 81.890

56 pz5 5 162.12 89.864 40.188 .55431 65.850 267.20 201.35

65 pz6 4 208.12 27.576 13.788 .13250 187.00 247.63 60.630

74 pz7 1 253.18 0.0000 0.0000 0.0000 253.18 253.18 0.0000

83 pz8 16 165.91 42.773 10.693 .25781 92.850 257.61 164.76

15 z_rel0 16 14.681 8.4979 2.1245 .57883 4.9000 27.000 22.100

24 z_rel1 16 25.912 11.793 2.9481 .45509 10.600 42.300 31.700

33 z_rel2 16 33.500 13.764 3.4410 .41086 12.600 49.900 37.300

42 z_rel3 9 28.722 11.480 3.8266 .39969 15.600 43.000 27.400

51 z_rel4 5 22.640 5.3984 2.4142 .23845 16.200 29.200 13.000

60 z_rel5 5 32.080 10.560 4.7224 .32917 19.500 43.000 23.500

69 z_rel6 4 40.475 5.0408 2.5204 .12454 36.200 47.600 11.400

78 z_rel7 1 43.700 0.0000 0.0000 0.0000 43.700 43.700 0.0000

87 z_rel8 16 35.956 4.2417 1.0604 .11797 26.600 46.800 20.200

99 M1pz 16 71.677 23.757 5.9392 .33144 38.650 126.72 88.070

100 M3pz 16 192.95 38.738 9.6845 .20077 129.62 275.08 145.46

101 D13pz 16 -121.27 32.439 8.1096 -.26749 -200.72 -66.540 134.18

102 M1z_rel 16 19.361 4.1758 1.0440 .21568 13.567 27.000 13.433

103 M3z_rel 16 39.456 3.2734 .81836 .08296 36.000 48.350 12.350

104 D13z_rel 16 -20.095 5.0483 1.2621 -.25122 -27.570 -10.000 17.570

************ ************

HISTOGRAM OF * D13pz * ( 101) GROUPED BY * pferd * ( 2)

************ ************

CASES WITH

UNUSED

VALUES FOR

*7 *8 *9 *10 pferd

MIDPOINTS.......................+.......................+.......................+.......................+.......................+

-45.000)

-60.000) *

-75.000) *

-90.000) M

-105.000) * M*

-120.000)* ** **

-135.000)M N

-150.000) * **

-165.000)

-180.000) N

-195.000) *

-210.000)

LEGEND FOR GROUP MEANS: M - MEAN COINCIDES WITH AN ASTERISK

N - MEAN DOES NOT COINCIDE WITH ANY ASTERISK

MEAN -128.712 -173.600 -135.056 -87.683 -106.487

STD.DEV. 5.123 38.346 21.161 19.708 18.318

S. E. M. 3.622 27.115 10.581 11.379 8.192

MAXIMUM -125.090 -146.485 -115.033 -66.540 -78.580

MINIMUM -132.335 -200.715 -154.740 -105.545 -125.605

CASES EXCL. ( 0) ( 0) ( 0) ( 0) ( 0)

CASES INCL. 2 2 4 3 5

---------------------------------------------------------------------------- ALL GROUPS COMBINED

| ANALYSIS OF VARIANCE TABLE FOR MEANS TAIL | (EXCEPT CASES WITH UNUSED

| SOURCE SUM OF SQUARES DF MEAN SQUARE F VALUE PROBABILITY | VALUES FOR VARIABLE pferd )

| -------- -------------- ---- ----------- ------- ----------- | MEAN -1.3E+2

| pferd 9235.1738 3 3078.3914 5.96 0.0243 | STD. DEV. 35.850

| ERROR 3616.9421 7 516.7060 | S. E. M. 10.809

|--------------------------------------------------------------------------| MAXIMUM -66.540

| EQUALITY OF MEANS TESTS; VARIANCES ARE NOT ASSUMED TO BE EQUAL | MINIMUM -2.0E+2

| WELCH 3, 3 3.59 0.1606 | CASES EXCLUDED ( 5)

| BROWN-FORSYTHE 3, 2 5.15 0.1668 | CASES INCLUDED 11

|--------------------------------------------------------------------------|

| LEVENE'S TEST FOR VARIANCES 3, 7 7.14 0.0155 | ROBUST S.D. 34.942

----------------------------------------------------------------------------

************ ************

HISTOGRAM OF * D13z_rel * ( 104) GROUPED BY * pferd * ( 2)

************ ************

CASES WITH

UNUSED

VALUES FOR

*7 *8 *9 *10 pferd

MIDPOINTS.......................+.......................+.......................+.......................+.......................+

-9.0000)

-10.8000) *

-12.6000) **

-14.4000)

-16.2000)* N

-18.0000) N **

-19.8000)N *

-21.6000) * * * *

-23.4000)* N N *

-25.2000) *

-27.0000) * *

-28.8000)

LEGEND FOR GROUP MEANS: M - MEAN COINCIDES WITH AN ASTERISK

N - MEAN DOES NOT COINCIDE WITH ANY ASTERISK

MEAN -20.025 -23.925 -23.821 -18.483 -16.578

STD.DEV. 4.207 4.561 3.325 7.405 3.407

S. E. M. 2.975 3.225 1.663 4.275 1.524

MAXIMUM -17.050 -20.700 -19.930 -10.000 -12.800

MINIMUM -23.000 -27.150 -27.570 -23.650 -20.800

CASES EXCL. ( 0) ( 0) ( 0) ( 0) ( 0)

CASES INCL. 2 2 4 3 5

---------------------------------------------------------------------------- ALL GROUPS COMBINED

| ANALYSIS OF VARIANCE TABLE FOR MEANS TAIL | (EXCEPT CASES WITH UNUSED

| SOURCE SUM OF SQUARES DF MEAN SQUARE F VALUE PROBABILITY | VALUES FOR VARIABLE pferd )

| -------- -------------- ---- ----------- ------- ----------- | MEAN -21.694

| pferd 64.5447 3 21.5149 0.83 0.5180 | STD. DEV. 4.959

| ERROR 181.3358 7 25.9051 | S. E. M. 1.495

|--------------------------------------------------------------------------| MAXIMUM -10.000

| EQUALITY OF MEANS TESTS; VARIANCES ARE NOT ASSUMED TO BE EQUAL | MINIMUM -27.570

| WELCH 3, 3 0.53 0.6925 | CASES EXCLUDED ( 5)

| BROWN-FORSYTHE 3, 5 0.82 0.5348 | CASES INCLUDED 11

|--------------------------------------------------------------------------|

| LEVENE'S TEST FOR VARIANCES 3, 7 2.10 0.1882 | ROBUST S.D. 4.564

----------------------------------------------------------------------------

/ FINISH

PROGRAM TERMINATED
